# Supplementary material for: Single-Molecule Real-Time (SMRT) Full-Length RNA-Sequencing Reveals Novel and Distinct mRNA Isoforms in Human Bone Marrow Cell Subpopulations
Source: Genes (Basel). 2019 Mar 27;10(4):253. doi: 10.3390/genes10040253 (PMC6523297; doi:10.3390/genes10040253)
Supplement: Supplementary file 1 [file genes-10-00253-s001.zip › genes-458537-supplementary/genes-458537-File S1. Supplemental Experimental Procedures.pdf]

SUPPLEMENTAL DATA

Single molecule real time (SMRT) full length RNA-sequencing reveals novel and distinct mRNA isoforms in human bone marrow cell subpopulations

Anne Deslattes Mays[1], Marcel O. Schmidt[1], Garrett T. Graham[1], Elizabeth Tseng[2], Primo Baybayan[2], Robert Sebra[3], Miloslav Sanda[1], Jean-Baptiste Mazarati[1,4], Anna T. Riegel[1], and Anton Wellstein[1]*

Contents:

- Supplemental Experimental and Computational Procedures
- Supplemental References
- Supplemental Figures S1 to Fig S6

- A separate file contains Tables S1 to S12: "Deslattes Genes Tables S1 to S12 March 18 2019.xlsx"
Table S1: Top five transcript isoform counts for genes with 2 to 16 exons (Fig. 5b)
Table S2: Transcript ID, gene names, detection of peptides by mass spectrometry (Fig. 5b and Table S1).
Table S3: Number of exons, transcript isoform numbers (Trans #) for genes in Fig. 5a.
Table S4: Number of exons, transcript isoform numbers (Trans #) for genes in Fig. 5c.
Table S5: Distribution of exon counts and transcript isoform frequency.
Table S6: Validation of the transcript isoforms.
Table S7: MatchAnnot analysis of isoform alignments.
Table S8: Supporting evidence for novel transcript isoforms of EEF1A1 using SQANTI2 and CAGE.
Table S9: Supporting evidence for novel transcript isoforms of ANXA1 using SQANTI2 and CAGE.
Table S10: Supporting evidence for exon-exon junctions in EEF1A1 with public RNAseq data.
Table S11: Supporting evidence for exon-exon junctions in ANXA1 with public RNAseq data.
Table S12: NCBI accession numbers

Supplemental Experimental and Computational Procedures

SQANTI2 is a private github repository of Elizabeth Tseng (co-author of this manuscript) and is a modification of the original SQANTI by the Ana Conesa Lab (1).

CAGE Peak data is from Fantom5 (2).

Intropolis is a list of exon-exon junctions across 21,5004 human RNA-seq from the Sequence Read Archive from spliced read alignment to hg19 with Rail-RNA (3).

Scripts:

Transcriptome Alignment and Assembly from Illumina Data.
The specific qsub command for the total bone marrow (T) alignment was:

```
#!/bin/bash
#$ -cwd
#$ -pe orte 32
#$ -N "th.fr.unstr.tot"
#$ -V
export PATH="/mnt/myenv2/bin:/mydata/cufflinks-2.2.1.Linux_x86_64;/mydata/bowtie2-2.2.5:/mydata/samtools-1.2:$PATH"
/mydata/tophat-2.0.14.Linux_x86_64/tophat2 -p 32 -G /results/Homo_sapiens/UCSC/hg19/Annotation/Genes/genes.gtf \
                      -r200 \
                      --library-type fr-unstranded \
                      -o /trimmed_fastq/transcriptome/ill.bm.total/tophat.2.0.14.fr-unstranded.gtf \
                         /results/Homo_sapiens/UCSC/hg19/Sequence/Bowtie2Index/genome \
                         /trimmed_fastq/trimmed.fasta.total.bm.non.ss/bm.trimmed.reads.1.fa \
                         /trimmed_fastq/trimmed.fasta.total.bm.non.ss/bm.trimmed.reads.2.fa
```

The specific command for cufflinks 2 assembly for total bone marrow (T) was:

```
#!/bin/bash
#$ -cwd
#$ -pe orte 32
#$ -N "cf2.tot.fr.un.gtf"
#$ -V
export PATH="/mnt/myenv2/bin:/mydata/cufflinks-2.2.1.Linux_x86_64;/mydata/bowtie2-2.21:/mydata/samtools-0.1.19:$PATH"
/mydata/cufflinks-2.2.1.Linux_x86_64/cufflinks -b /results/Homo_sapiens/UCSC/hg19/Sequence/WholeGenomeFasta/genome.fa \
  -p 32 \
  -g /results/Homo_sapiens/UCSC/hg19/Annotation/Genes/genes.gtf \
  -L tot.fr.unstranded.gtf \
  -o /trimmed_fastq/transcriptome/ill.bm.total/cufflinks2.2.1.tophat.2.0.14.fr.unstranded.gtf.5.19 \
  /trimmed_fastq/transcriptome/ill.bm.total/tophat.2.0.14.fr-unstranded.gtf/accepted_hits.bam
```

The specific qsub command for the lineage-negative (N) alignment was:

```
#!/bin/bash
```

```
#$ -cwd
#$ -pe orte 32
#$ -N "th.neg.gtf"
#$ -V
export PATH="/mnt/myenv2/bin:/mydata/cufflinks-2.2.1.Linux_x86_64;/mydata/bowtie2-2.2.5:/mydata/samtools-1.2:$PATH"
/usr/local/bin/tophat -p 32 -G /results/Homo_sapiens/UCSC/hg19/Annotation/Genes/genes.gtf \
                      --library-type fr-firststrand \
                      -r200 \
                      -o /trimmed_fastq/transcriptome/ill.lin.neg/tophat.2.0.14.fr.firststrand.no.mm.gtf \
                         /results/Homo_sapiens/UCSC/hg19/Sequence/Bowtie2Index/genome \
                         /trimmed_fastq/trimmed.fasta.lin.neg.ss/lin.neg.trimmed.reads.1.fa \
                         /trimmed_fastq/trimmed.fasta.lin.neg.ss/lin.neg.trimmed.reads.2.fa
```

The specific command for cufflinks 2 assembly for lineage-negative (N) was:

```
#!/bin/bash
#$ -cwd
#$ -pe orte 32
#$ -N "cf2.ill.lin.neg.gtf"
#$ -V
export PATH="/mnt/myenv2/bin:/mydata/cufflinks-2.2.1.Linux_x86_64;/mydata/bowtie2-2.21:/mydata/samtools-0.1.19:$PATH"
/mydata/cufflinks-2.2.1.Linux_x86_64/cufflinks -b /results/Homo_sapiens/UCSC/hg19/Sequence/WholeGenomeFasta/genome.fa \
  -p 32 \
  -g /results/Homo_sapiens/UCSC/hg19/Annotation/Genes/genes.gtf \
  -L ill.lin.neg.nm.th \
  -o /trimmed_fastq/transcriptome/ill.lin.neg/cufflinks2.2.1.no.mm.no.u.gtf \
   /trimmed_fastq/transcriptome/ill.lin.neg/tophat.2.0.14.fr.firststrand.no.mm/accepted_hits.bam
```

Analysis of full length transcript by ToFU:

All processing was done on an amazon cloud instant on which the SMRTAnalysis software was first installed followed by installing the then referred to cDNA_primer prebam version of ToFU.

Before running the tofu_wrap.py software, these files needed to be prepared.

1. CircularConsensus.sh to obtain circular consensus reads (ccs) from movie files
2. Classify to obtain Full length non-chimeric reads (FL) and non-full length (nFL) ccs reads

The ccs reads now classified into FL and nFL are then converted from fastq to fasta files.

So that prior to running tofu_wrap.py, one has:

1. Isoseq_nfl.fasta (nFL) ccs reads
2. Isoseq_flnc.fasta (FL)
3. Input.fofn (quality information from the movie files)
4. Reads_of_insert.fofn (the ccs reads listed as file names)
5. Reference database (hg19)

Then, the ToFU step of final assembly is accomplished with:

```
tofu_wrap.py --nfl_fa isoseq_nfl.fasta \
--ccs_fofn reads_of_insert.fofn --bas_fofn input.fofn \
-d clusterOut --quiver --use_sge --max_sge_jobs 120 \
--gmap_db /home/UNIXHOME/etseng/share/gmap_db_new/ \
--gmap_name hg19 \
--output_seqid_prefix tissue1
isoseq_flnc.fasta final.consensus.fa
```

Confirmation of novel transcript isoforms with blast

To confirm novel transcripts isoforms, two blastable databases were prepared separately for the total bone marrow (total.bm.non.ss) and lineage-negative short read RNA sequences (lin.neg.ss) using the example script below. The full length RNA-seq reads for each gene was split into single sequence files then batch blasted in a script such as this used for *ANXA1*:

```
#!/bin/bash
endings="aa ab ac ad ae af ag ah ai aj ak al am an ao ap aq ar as at au av aw ax ay az
        ba bb bc bd be bf bg bh bi bj bk bl bm bn bo bp bq br bs bt bu bv bw bx by bz
        ca cb cc cd cd cf cg ch ci cj ck cl cm cn co"
#rm ANXA1.lin.neg.coverage.txt
#rm ANXA1.total.coverage.txt
for end in $endings;
do
   echo ANXA1.split."$end"
   blastn -db ../lin.neg.ss/lin.neg.ss -perc_identity 99 -ungapped -num_threads 32  -max_target_seqs 10000 -query
ANXA1.split."$end" -outfmt "6 qseqid qstart qend \
qseq sseq sseqid sstart send" -out ANXA1.split."$end".lin.neg.out
   blastn -db ../../total.bm.non.ss/total.bm.non.ss -perc_identity 99 -ungapped -num_threads 32 -max_target_seqs 10000 -
query ANXA1.split."$end" -outfmt "6 qseqid \
qstart qend qseq sseq sseqid sstart send" -out ANXA1.split."$end".total.out
done
```

Using this output file, a *summarize* script provides the coverage.

```
#!/bin/bash
endings ="aa ab ac ad ae af ag ah ai aj ak al am an ao ap aq ar as at au av
ba bb bc bd be bf bg bh bi bj bk bl bm bn bo bp bq br bs bt bu bv
ca cb cc cd cd cf cg ch ci cj ck cl cm cn co"
for end in endings
do
echo ANXA1.split."$end"
var=$(grep ">" ANXA1.split."$end"
var2=${var:1}
echo "$var2"
grep "$var2" ANXA1.split."$end".lin.neg.out | cut -c -20 > ANXA1.split."$end".lin.neg.num.txt
sort -n -k 2,3 ANXA1.split."$end".lin.neg.num.txt > ANXA1."$end" > . "$var2".lin.neg.txt
```

```
sort -n -k 2,3 ANXA1.split."$end".lin.neg.out > ANXA1."$end". "$var2".lin.neg.fa.txt
cat ANXA1."$end" . "$var2".lin.neg.txt >> ANXA1.lin.neg.coverage.txt
grep "$var2" ANXA1.split."$end".total.out | cut -c -20 >ANXA1.split."$end".total.num.txt
sort -n -k 2,3 ANXA1.split."$end".total.num.txt >ANXA1."$end". "$var2".total.txts
sort -n -k 2,3 ANXA1.split."$end".total.out > ANXA1."$end"."$var2".total.fa.txt
cat ANXA1."$end"."$var2".total.txt >> ANXA1.total.coverage.txt
done
```

Summarize finds the matches and prepares the coverage file. Next, a series of *awk* scripts find the gaps in coverage as a result of the blast and limits the hits to 100 bp.
The output of the short reads that match each of the novel isoforms were then searched for the full length reads. All genes followed the same stepwise procedure.
step 1 - Simplify the blast output capturing the ends of the hit, and the length of the overlap.

```
cat ANXA1.total.coverage.txt | awk '{print $1 "\t" $2 "\t" $3 "\t" $3-$2+1}' >ANXA1.total.coverage.maths.txt
```

Ensure the hits are at least 100 bp

```
grep "100" ANXA1.total.coverage.maths.txt > ANXA1.total.100.coverage.maths.txt
```

step 2 - preserving the end of the previous line, this awk script identifies gaps.

```
cat ANXA1.total.100.coverage.maths.txt | awk '{print var "\t" $1 "\t" $2 "\t" $3 "\t" $4 "\t" var-$2;var=$3}' >
ANXA1.total.100.coverage.all.txt
```

step 3 - use the end of the match from the previous line to determine if there is a gap

```
grep "\-" ANXA1.total.coverage.all.txt
```

The specific command for the Sqanti2 package was:

```
python ~/GitHub/SQANTI2/sqanti_qc2.py -t 30 53A_negative.good.5merge.collapsed.longest_rep.fa
gencode.v29.annotation.gtf /pbi/dept/bifx/etseng/genomes/hg38/hg38_noalt.fa --cage_peak
~/share/FANTOM/hg38.cage_peak_phase1and2combined_coord.bed -c
"Public_Intronpolis/*count_10.*modified" --polyA_motif_list polyA.list
```
"polyA.list"
```
aataaa
attaaa
agtaaa
tataaa
cataaa
gataaa
aatata
aataca
aataga
aaaaag
actaaa
aagaaa
aatgaa
tttaaa
aaaaca
ggggct
```

Supplemental References

1. Tardaguila, M., de la Fuente, L., Marti C., Pereira, C., Pardo-Palacios, F.J., Del Risco, H., Ferrell, M., Mellado, M., Macchietto, M., Verheggen, K., Edelmann, M., Ezkurdia, I., Vazquez, J., Tress, M., Mortazavi, A., Martens, L., Rodriguez-Navarro, S., Moreno-Manzano, V., Conesa, A. (2018) SQANTI: extensive characterization of long-read transcript sequences for quality control in full-length transcriptome identification and quantification. *Genome Res.* **28**, 396-411
2. Lizio, M, et al. (2015) Gateways to the FANTOM5 promoter level mammalian expression atlas. Genome Biology **16**, 22
3. Nellore, A., et al. (2016) Human splicing diversity and the extent of unannotated splice junctions across human RNA-seq samples on the Sequence Read Archive. Genome Biology **17**, 266
4. Shilov, I. V., Seymour, S. L., Patel, A. A., Loboda, A., Tang, W. H., Keating, S. P., Hunter, C. L., Nuwaysir, L. M., and Schaeffer, D. A. (2007) The Paragon Algorithm, a Next Generation Search Engine That Uses Sequence Temperature Values and Feature Probabilities to Identify Peptides from Tandem Mass Spectra. *Molecular & Cellular Proteomics*. **6**, 1638–1655
5. Tang, W. H., Shilov, I. V., and Seymour, S. L. (2008) Nonlinear fitting method for determining local false discovery rates from decoy database searches. *J Proteome Res*. **7**, 3661–3667

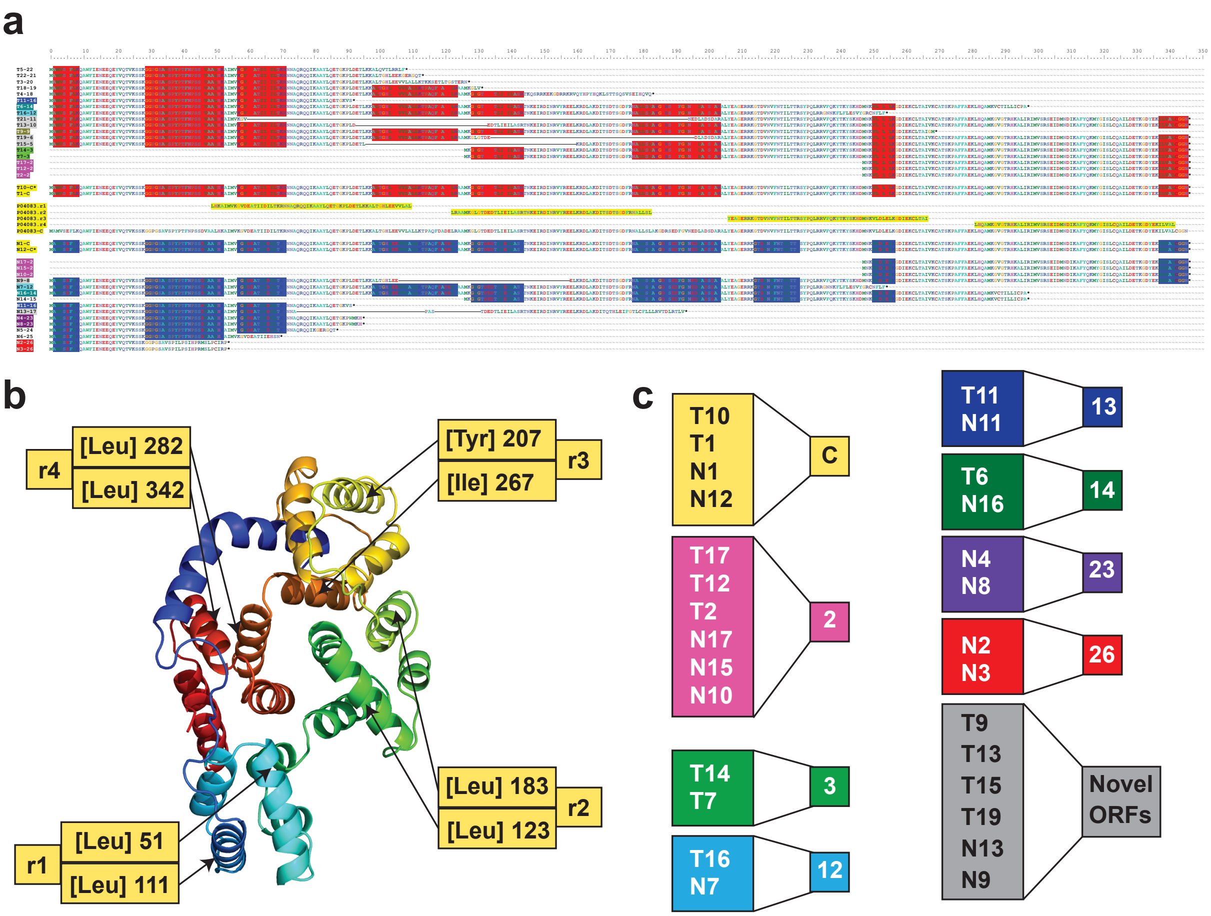

Figure S1. Amino acid sequence alignment and proteins predicted from the transcript isoforms identified for *ANXA1* (see **Fig. 4c,d**). (**a**) Amino acid sequence alignments including the isoform identifiers. The canonical protein and conserved repeat domains r1 - r4 are highlighted in yellow. ORFs coding for the same protein are shown in matching colors. Note: Solid lines connecting protein fragments indicate contiguous amino acid sequences predicted from the respective transcript isoform. (**b**) Predicted protein structure of the canonical *ANXA1* protein P04083 with repeat domains r1 to r4 indicated. (**c**) Common predicted proteins for groups of transcript isoforms, ORFs 2, 3, 12, 13, 14, 23, 26 and the canonical ORF (C) are listed and shown with the respective color from panel (**a**). Novel ORFs are indicated.

(A magnified portion of Figure S1a is on the next page.)

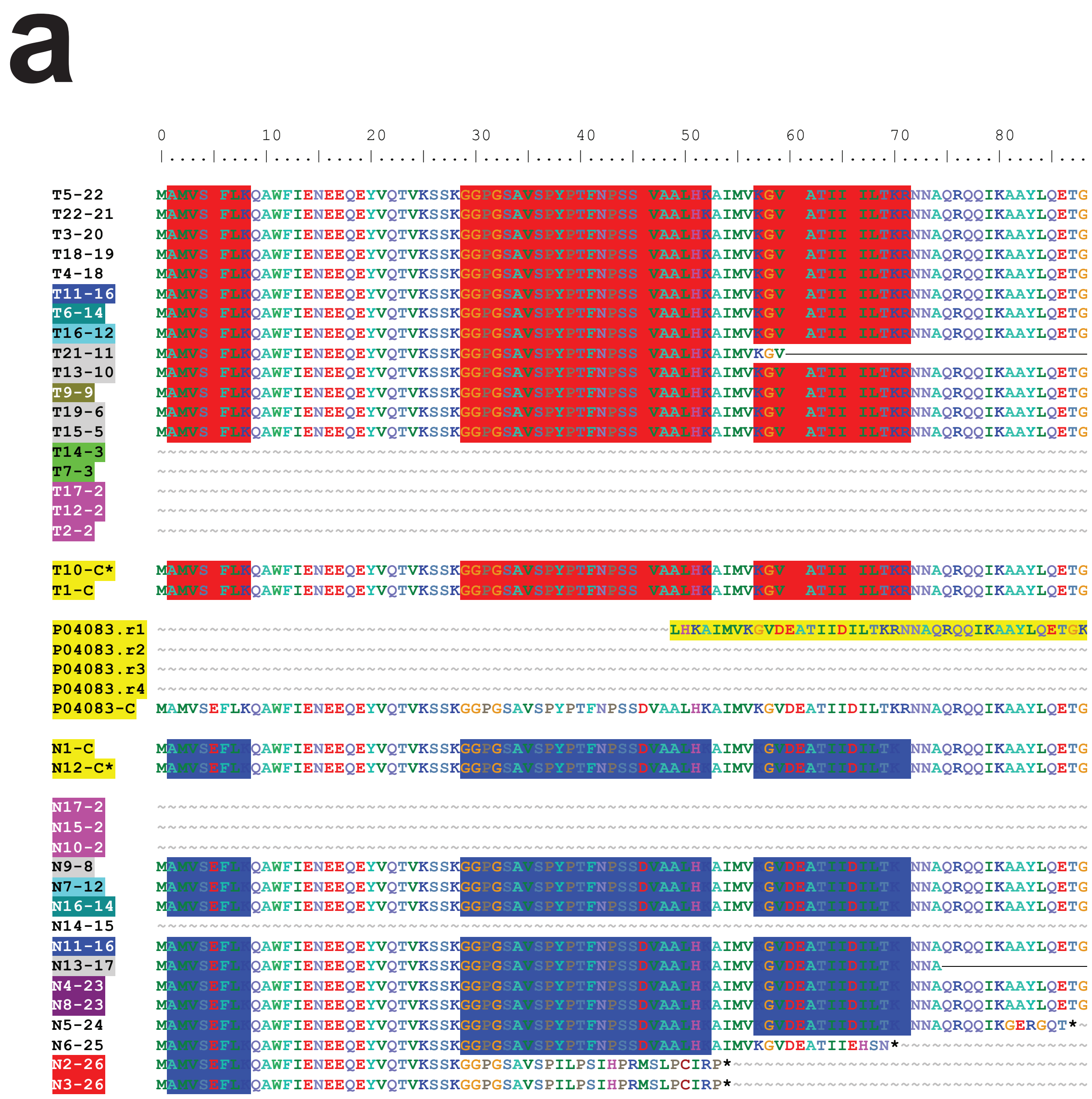

Figure S1a magnified for better readibility.

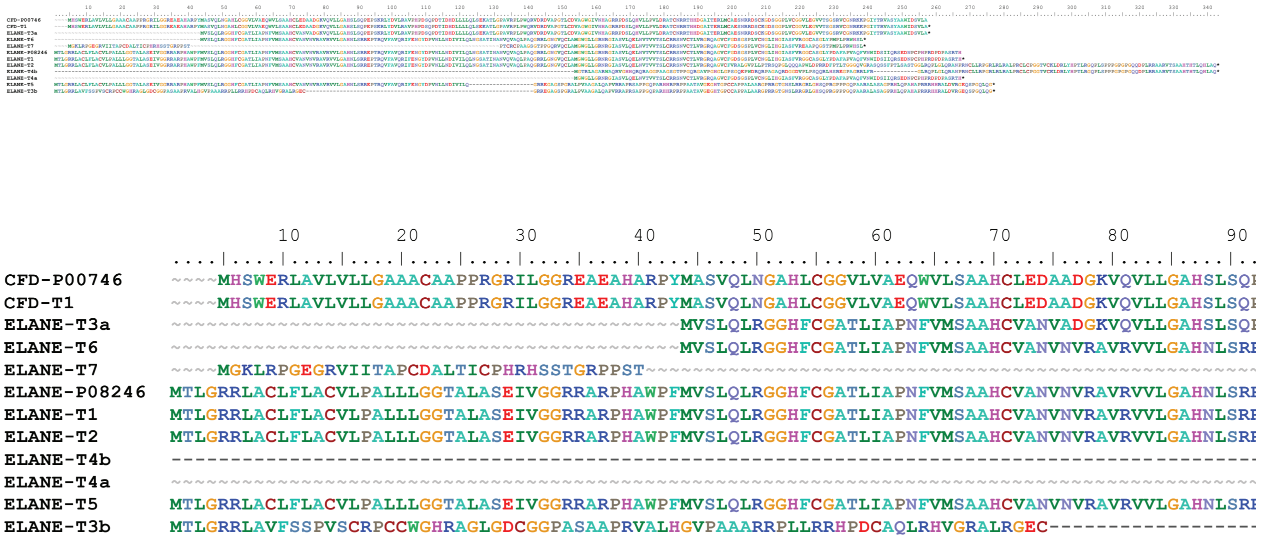

Figure S2. Multiple amino acid sequence alignment predicted from transcript isoforms for *ELANE* and *CFD* The alignment is shown in the top panel. The identifiers of the transcript isoforms are included.

For better readibility a magnified portion of the figure is shown as the bottom panel.

a

b

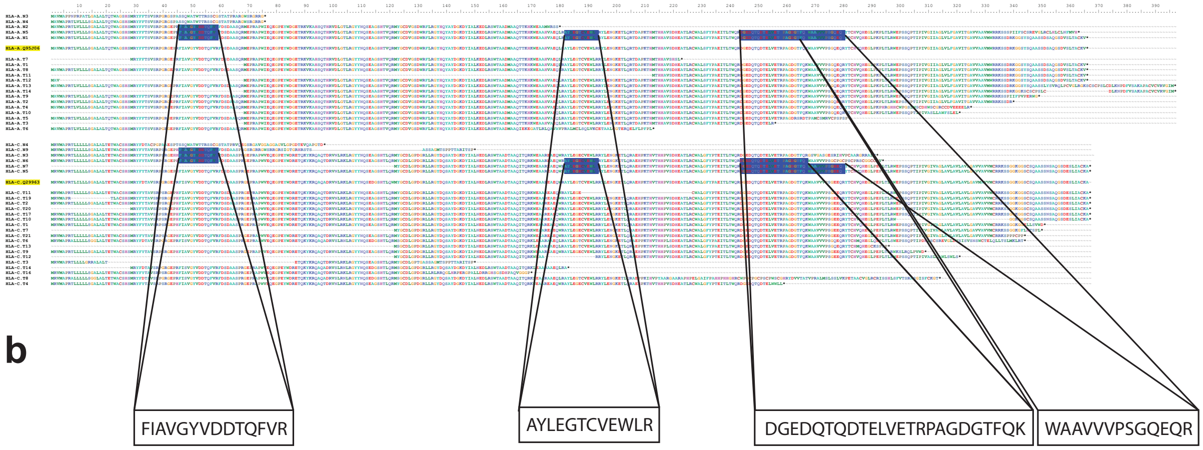

a

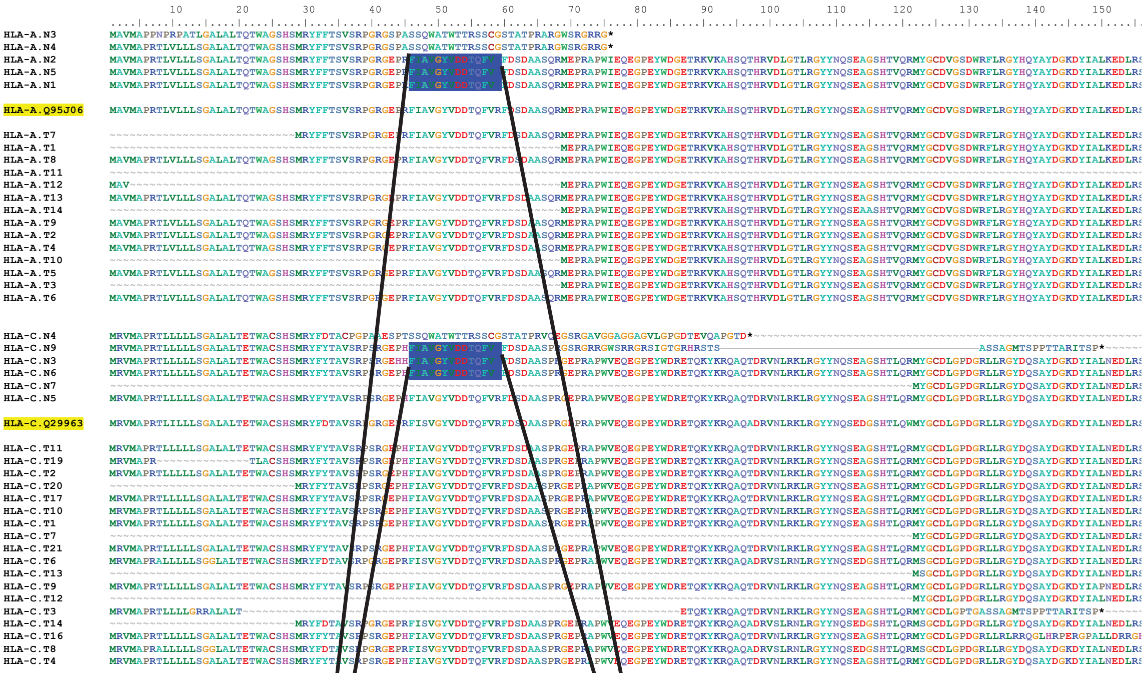

Figure S3. Multiple amino acid sequence alignment **(a)** and mass spectrometry detected peptides (**b**) for *HLA-A*, *-B* and *-C* transcripts. **(a)** Sequences predicted from transcript isoforms of *HLA-A, HLA-B* and *HLA-C*. The identifiers of the transcript isoforms are included. Canonical amino acid sequences are highlighted in yellow. **(b)** Peptide fragments identified by mass spectrometry analysis of tryptic fragments of proteins extracted from lin-neg bone marrow cells. The spectra are shown in **Fig. 5d-f.**

For better readibility a magnified portion of Fig S3a is shown in the bottom panel

a lineage-negative bone marrow cells

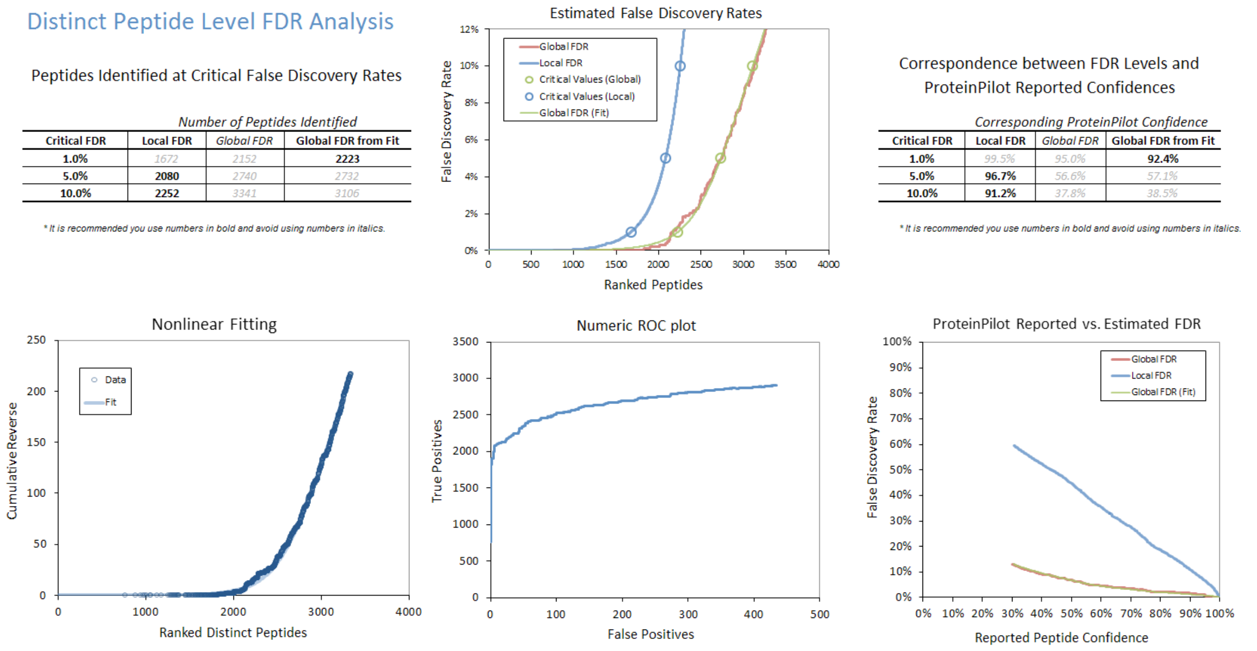

Number of Peptides Identified

Critical FDR	Local FDR	*Global FDR*	Global FDR from Fit
**1.0%**	*1672*	*2152*	**2223**
**5.0%**	**2080**	*2740*	*2732*
**10.0%**	**2252**	*3341*	*3106*

* It is recommended you use numbers in bold and avoid using numbers in italics.

Corresponding ProteinPilot Confidence

Critical FDR	Local FDR	*Global FDR*	Global FDR from Fit
**1.0%**	*99.5%*	*95.0%*	**92.4%**
**5.0%**	**96.7%**	*56.6%*	*57.1%*
**10.0%**	**91.2%**	*37.8%*	*38.5%*

* It is recommended you use numbers in bold and avoid using numbers in italics.

b lineage-positive bone marrow cells

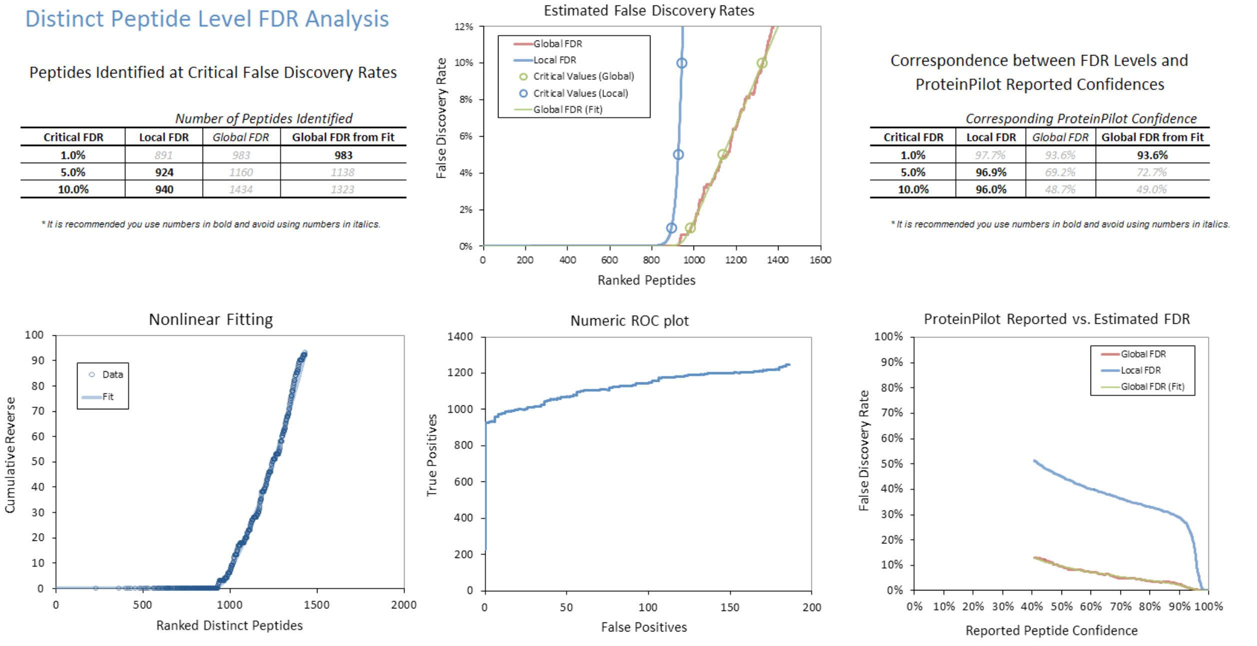

Number of Peptides Identified

Critical FDR	Local FDR	*Global FDR*	Global FDR from Fit
**1.0%**	*891*	*983*	**983**
**5.0%**	**924**	*1160*	*1138*
**10.0%**	**940**	*1434*	*1323*

* It is recommended you use numbers in bold and avoid using numbers in italics.

Corresponding ProteinPilot Confidence

Critical FDR	Local FDR	*Global FDR*	Global FDR from Fit
**1.0%**	*97.7%*	*93.6%*	**93.6%**
**5.0%**	**96.9%**	*69.2%*	*72.7%*
**10.0%**	**96.0%**	*48.7%*	*49.0%*

* It is recommended you use numbers in bold and avoid using numbers in italics.

Figure S4. Summary of the false discovery rate (FDRs) of the shotgun mass spectrometry of proteins extracted from bone marrow-derived cells. FDR analysis was performed on the ProteinPilot platform (4, 5). The cutoff for positive peptide identification was set at an FDR < 1%. The graphs and tables represent the overview of the proteomics analysis of lineage-negative **(a)** and lineage-positive **(b)** bone marrow cells.

Figure S5. Blast alignment of EEF1A1 novel transcripts with NCBI EST repository copied below

Figure S6. Blast alignment of ANXA1 novel transcripts with NCBI EST repository copied further below

BLASTN 2.9.0+
Reference: Zheng Zhang, Scott Schwartz, Lukas Wagner, and
Webb Miller (2000), "A greedy algorithm for aligning DNA
sequences", J Comput Biol 2000; 7(1-2):203-14.

RID: 8PV46HTU015

Database: Database of GenBank+EMBL+DDBJ sequences from EST Divisions
77,566,966 sequences; 43,200,890,307 total letters
Query= T1-33 [organism=Homo sapiens] eef1a1

Length=1638

```
                                                                  Score        E     Max
Sequences producing significant alignments:                      (Bits)     Value  ident

BX421307.2 BX421307 Homo sapiens B CELLS (RAMOS CELL LINE) Hom...  1622       0.0    100%
BX443032.2 BX443032 Homo sapiens B CELLS (RAMOS CELL LINE) Hom...  1618       0.0    100%
BY794942.2 BY794942 Homo sapiens eye Homo sapiens cDNA clone H...  1616       0.0    100%
BX417098.2 BX417098 Homo sapiens PLACENTA Homo sapiens cDNA cl...  1615       0.0    100%
DC527884.1 DC527884 chimpanzee skin cDNA library PstA Pan trog...  1611       0.0    100%
BX441194.2 BX441194 Homo sapiens FETAL BRAIN Homo sapiens cDNA...  1611       0.0    100%
AL541984.3 AL541984 Homo sapiens PLACENTA Homo sapiens cDNA cl...  1609       0.0    100%
BQ049312.1 AGENCOURT_6795094 NIH_MGC_85 Homo sapiens cDNA clon...  1607       0.0    100%
BX417528.2 BX417528 Homo sapiens PLACENTA Homo sapiens cDNA cl...  1604       0.0    100%
BM477966.1 AGENCOURT_6482916 NIH_MGC_85 Homo sapiens cDNA clon...  1602       0.0    100%

ALIGNMENTS

Query       3     TTTTTCGCAACGGGTTTGCCGCCAGAACACAGGTGTCGTGAAAACTACCCCTAAAAGCCAAAATGGGAAAGGAAAAGACTCATATCAACATTGTCGTCATTGGACACGTAGATTCGGGCAAGTCCACCACTACTGGCCATCTGATCTATA  152
BY794942.2  25                       ...................................................................................................................................  155
DC527884.1  9     ......................................................................................................................................................  158

Query       153   AATGCGGTGGCATCGACAAAAGAACCATTGAAAAATTTGAGAAGGAGGCTGCTGAGATGGGAAAGGGCTCCTTCAAGTATGCCTGGGTCTTGGATAAACTGAAAGCTGAGCGTGAACGTGGTATCACCATTGATATCTCCTTGTGGAAAT  302
BY794942.2  156   ......................................................................................................................................................  305
DC527884.1  159   ............................................................................................................................T.........................  308

Query       303   TTGAGACCAGCAAGTACTATGTGACTATCATTGATGCCCCAGGACACAGAGACTTTATCAAAAACATGATTACAGGGACATCTCAGGCTGACTGTGCTGTCCTGATTGTTGCTGCTGGTGTTGGTGAATTTGAAGCTGGTATCTCCAAGA  452
BY794942.2  306   ......................................................................................................................................................  455
DC527884.1  309   .......................................................C..............................................................................................  458

Query       453   ATGGGCAGACCCGAGAGCATGCCCTTCTGGCTTACACACTGGGTGTGAAACAACTAATTGTCGGTGTTAACAAAATGGATTCCACTGAGCCACCCTACAGCCAGAAGAGATATGAGGAAATTGTTAAGGAAGTCAGCACTTACATTAAGA  602
BY794942.2  456   ......................................................................................................................................................  605
DC527884.1  459   ......................................................................................................................................................  608

Query       603   AAATTGGCTACAACCCCGACACAGTAGCATTTGTGCCAATTTCTGGTTGGAATGGTGACAACATGCTGGAGCCAAGTGCTAACATGCCTTGGTTCAAGGGATGGAAAGTCACCCGTAAGGATGGCAATGCCAGTGGAACCACGCTGCTTG  752
BY794942.2  606   ......................................................................................................................................................  755
DC527884.1  609   ......................................................................................................................................................  758
AL541984.3  1                                                                                                                                   ........................  24

Query       753   AGGCTCTGGACTGGTGTTCTCAAACCCGGTATGGTGGTCACCTTTGCTCCAGTCAACGTTACAACGGAAGTAAAATCTGTCGAAATGCACCATGAAGCTTTGAGTGAAGCTCTTCCTGGGGACAATGTGGGCTTCAATGTCAAGAATGTG  902
BX421307.2  27            ..............................................................................................................................................  168
BX443032.2  53            ...............................................................M..............................................................................  194
BY794942.2  756   .............                                                                                                                                           768
BY794942.2  871           ..............................................................................................................................................  1012
BX417098.2  16            ..............................................................................................................................................  157
DC527884.1  874           ..............................................................................................................................................  1015
DC527884.1  759   .............                                                                                                                                           771
BX441194.2  65            ..........................W...................................................................................................................  206
AL541984.3  140           ..............................................................................................................................................  281
AL541984.3  25    .............                                                                                                                                           37
BQ049312.1  25            ..............................................................................................................................................  166
BX417528.2  32            ..............................................................................................................................................  173
BM477966.1  9             ..............................................................................................................................................  150

Query       903   TCTGTCAAGGATGTTCGTCGTGGCAACGTTGCTGGTGACAGCAAAAATGACCCACCAATGGAAGCAGCTGGCTTCACTGCTCAGGTGATTATCCTGAACCATCCAGGCCAAATAAGCGCCGGCTATGCCCCTGTATTGGATTGCCACACG  1052
BX421307.2  169   ......................................................................................................................................................  318
BX443032.2  195   ......................................................................................................................................................  344
BY794942.2  1013  ......................................................................................................................................................  1163
                                                                                                                        \
                                                                                                                        |
                                                                                                                        G
BX417098.2  158   ......................................................................................................................................................  307
DC527884.1  1016  ......................................................................................................................................................  1165
BX441194.2  207   ......................................................................................................................................................  356
AL541984.3  282   ......................................................................................................................................................  431
BQ049312.1  167   ......................................................................................................................................................  316
BX417528.2  174   ......................................................................................................................................................  323
BM477966.1  151   ......................................................................................................................................................  300
```

```
Query       1053  GCTCACATTGCATGCAAGTTTGCTGAGCTGAAGGAAAAGATTGATCGCCGTTCTGGTAAAAAGCTGGAAGATGGCCCTAAATTCTTGAAGTCTGGTGATGCTGCCATTGTTGATATGGTTCCTGGCAAGCCCATGTGTGTTGAGAGCTTC  1202
BX421307.2  319   ......................................................................................................................................................  468
BX443032.2  345   ......................................................................................................................................................  494
BY794942.2  1164  ......................................................................................................................................................  1313
BX417098.2  308   ......................................................................................................................................................  457
DC527884.1  1166  ......................................................................................................................................................  1315
BX441194.2  357   ......................................................................................................................................................  506
AL541984.3  432   ......................................................................................................................................................  581
BQ049312.1  317   ......................................................................................................................................................  466
BX417528.2  324   ......................................................................................................................................................  473
BM477966.1  301   ......................................................................................................................................................  450

Query       1203  TCAGACTATCCACCTTTGGGTCGCTTTGCTGTTCGTGATATGAGACAGACAGTTGCGGTGGGTGTCATCAAAGCAGTGGACAAGAAGGCTGCTGGAGCTGGCAAGGTCACCAAGTCTGCCCAGAAAGCTCAGAAGGCTAAATGAATATTA  1352
BX421307.2  469   ......................................................................................................................................................  618
BX443032.2  495   ......................................................................................................................................................  644
BY794942.2  1314  ......................................................................................................................................................  1463
BX417098.2  458   ......................................................................................................................................................  607
DC527884.1  1316  ...............................................................A......................................................................................  1465
BX441194.2  507   ......................................................................................................................................................  656
AL541984.3  582   ......................................................................................................................................................  731
BQ049312.1  467   ......................................................................................................................................................  616
BX417528.2  474   ......................................................................................................................................................  623
BM477966.1  451   ......................................................................................................................................................  600

Query       1353  TCCCTAATACCTGCCACCCCACTCTTAATCAGTGGTGGAAGAACGGTCTCAGAACTGTTTGTTTCAATTGGCCATTTAAGTTTAGTAGTAAAAGACTGGTTAATGATAACAATGCATCGTAAAACCTTCAGAAGGAAAGGAGAATgtttt  1502
BX421307.2  619   ......................................................................................................................................................  768
BX443032.2  645   ......................................................................................................................................................  794
BY794942.2  1464  ......................................................................................................................................................  1613
BX417098.2  608   ......................................................................................................................................................  757
DC527884.1  1466  .........................C............................................................................................................................  1615
BX441194.2  657   ......................................................................................................................................................  806
AL541984.3  732   ......................................................................................................................................................  881
BQ049312.1  617   ......................................................................................................................................................  766
BX417528.2  624   ..................................................................................................................................T...................  773
BM477966.1  601   ......................................................................................................................................................  750

Query       1503  gtggaccactttggttttcttttttgcgtgtggcagttttaagttattagtttttaaaatcagtactttttaATGGAAACAACTTGACCAAAAATTTGTCACAGAATTTTGAGACCCATTAAAAAAGTTAAATGAG  1638
BX421307.2  769   ........................................................................................................................................  904
BX443032.2  795   ........................................................................................................................................  930
BY794942.2  1614  ........................................................................................................................................  1749
BX417098.2  758   ..................................................................................................................-.....................  892
DC527884.1  1616  ........................................................................................................................................  1751
BX441194.2  807   ..................................................................................................................-.....................  941
AL541984.3  882   ...............................K.S......................................................................................................  1018
                                                                                                                      \
                                                                                                                      |
                                                                                                                      M
BQ049312.1  767   .................................................................................................................................T....   901
                                                                                                                                 \
                                                                                                                                 |
                                                                                                                                 A
BX417528.2  774   ....................................................................................................................-........-..........  907
BM477966.1  751   .......................................................-.................................................................N...A.......   883

Query= T2-C* [organism=Homo sapiens] eef1a1

Length=1742


                                                                      Score        E     Max
Sequences producing significant alignments:                          (Bits)     Value  ident

DC527884.1 DC527884 chimpanzee skin cDNA library PstA Pan trog...  3193       0.0    100%
BY794942.2 BY794942 Homo sapiens eye Homo sapiens cDNA clone H...  3175       0.0    100%
CF111220.1 Shultzomica04471 Rat lung airway and parenchyma cDN...  2361       0.0    91%
BX426149.2 BX426149 Homo sapiens NEUROBLASTOMA Homo sapiens cD...  2146       0.0    99%
CO001006.1 OC028 pre-oestrum adult sheep ovary library Ovis ar...  2050       0.0    93%
BX418564.2 BX418564 Homo sapiens FETAL BRAIN Homo sapiens cDNA...  2047       0.0    97%
BX426150.2 BX426150 Homo sapiens NEUROBLASTOMA Homo sapiens cD...  2037       0.0    99%
AL567209.3 AL567209 Homo sapiens FETAL BRAIN Homo sapiens cDNA...  2001       0.0    98%
BX440470.1 BX440470 Homo sapiens FETAL BRAIN Homo sapiens cDNA...  1999       0.0    98%
BU902062.1 AGENCOURT_10127553 NIH_MGC_71 Homo sapiens cDNA clo...  1999       0.0    98%

ALIGNMENTS

Query       2     TTTTTCGCAACGGGTTTGCCGCCAGAACACAGGTGTCGTGAAAACTACCCCTAAAAGCCAAAATGGGAAAGGAAAAGACTCATATCAACATTGTCGTCATTGGACACGTAGATTCGGGCAAGTCCACCACTACTGGCCATCTGATCTATA  151
DC527884.1  9     ......................................................................................................................................................  158
BY794942.2  25                       .......................................................................................................................................  155
CF111220.1  7              .........-..T......G.......T......G.C...G........A.......................C........C.....A.................C..............A..C.....C........C.  151
                              \                                       \
                              |                                       |
```

```
                              A                                     ATTC
BX426150.2  1                           ..T...........................................................................................................................  126
BU902062.1  1                 .........................................................................................................................................  137

Query       152   AATGCGGTGGCATCGACAAAAGAACCATTGAAAAATTTGAGAAGGAGGCTGCTGAGATGGGAAAGGGCTCCTTCAAGTATGCCTGGGTCTTGGATAAACTGAAAGCTGAGCGTGAACGTGGTATCACCATTGATATCTCCTTGTGGAAAT  301
DC527884.1  159   ............................................................................................................................T.........................  308
BY794942.2  156   ......................................................................................................................................................  305
CF111220.1  152   ....T.....A.......................G.................G...........A........................C....C....................G...........T.....C......C.......G.  301
BX426150.2  127   ......................................................................................................................................................  276
BU902062.1  138   ......................................................................................................................................................  287

Query       302   TTGAGACCAGCAAGTACTATGTGACTATCATTGATGCCCCAGGACACAGAGACTTTATCAAAAACATGATTACAGGGACATCTCAGGCTGACTGTGCTGTCCTGATTGTTGCTGCTGGTGTTGGTGAATTTGAAGCTGGTATCTCCAAGA  451
DC527884.1  309   .......................................................C..............................................................................................  458
BY794942.2  306   ......................................................................................................................................................  455
CF111220.1  302   .......................................................C.....G...........G..C.................C.............................G.........................  451
CO001006.1  1464    ....................T..C.............................C....................C.....C.....................................................C.............  1317
BX426150.2  277   ......................................................................................................................................................  426
BU902062.1  288   ......................................................................................................................................................  437

Query       452   ATGGGCAGACCCGAGAGCATGCCCTTCTGGCTTACACACTGGGTGTGAAACAACTAATTGTCGGTGTTAACAAAATGGATTCCACTGAGCCACCCTACAGCCAGAAGAGATATGAGGAAATTGTTAAGGAAGTCAGCACTTACATTAAGA  601
DC527884.1  459   ......................................................................................................................................................  608
BY794942.2  456   ......................................................................................................................................................  605
CF111220.1  452   .C...........T........T..............TT.............G........T.....C.................C........A.....T...........C........C.................C..........  601
BX426149.2  1196                                                                          ......-..A....-.A...............G.............................................  1121
CO001006.1  1316  .C...........T.......................C.......................T..C.................T..............T..............C..........................C..........  1167
BX418564.2  1196                                                                     ....-....-......-...-...-...YH.VS..-.R......-W.-..G..W....-.R......M.....K....-.R.-  1125
BX426150.2  427   ......................................................................................................................................................  576
AL567209.3  1145                                                                                              .......S.....-...W.-..........-K.W........R.....-....A.M.C  1091
                                                                                                                                                               \
                                                                                                                                                               |
                                                                                                                                                               M
BX440470.1  1128                                                                                                                                                 ....-  1125
BU902062.1  438   ......................................................................................................................................................  587

Query       602   AAATTGGCTACAACCCCGACACAGTAGCATTTGTGCCAATTTCTGGTTGGAATGGTGACAACATGCTGGAGCCAAGTGCTAACATGCCTTGGTTCAAGGGATGGAAAGTCACCCGTAAGGATGGCAATGCCAGTGGAACCACGCTGCTTG  751
DC527884.1  609   ......................................................................................................................................................  758
BY794942.2  606   ......................................................................................................................................................  755
CF111220.1  602   ................T.................................................................T.....G..........................C..A.......G.........C...........G.  751
BX426149.2  1120  ......................................................................................................................................................  971
CO001006.1  1166  ..............................................C...................................T.....A................................C....................C.......  1017
BX418564.2  1124  ......S....-..........-R.....-.................-.....-..........................C......................-..............................................  981
BX426150.2  577   ......................................................................................................................................................  726
AL567209.3  1090  ............C.........................................................................................................................................  941
BX440470.1  1124  .....-..-W.-.............-...-....-S.M...Y..-.K......-..............-.....................K...........................................................  983
                     \
                     |
                     C
BU902062.1  588   ......................................................................................................................................................  737

Query       752   AGGCTCTGGACTGCATCCTACCACCAACTCGTCCAACTGACAAGCCCTTGCGCCTGCCTCTCCAGGATGTCTACAAAATTGGTGGTATTGGTACTGTTCCTGTTGGCCGAGTGGAGACTGGTGTTCTCAAACCCGGTATGGTGGTCACCT  901
DC527884.1  759   ......................................................................................................................................................  908
BY794942.2  756   ......................................................................................................................................................  905
CF111220.1  752   .A...T..........T..G..G.......................TC....A.....C..............T........C..C.....C.....C.....G...........A.................T...........T....  901
BX426149.2  970   ......................................................................................................................................................  821
CO001006.1  1016  .A........T........G...........C...........A........TT...................T.......................A.....G..T..T.......................T..C.............  867
BX418564.2  980   ...............................................C......................................................................................................  831
BX426150.2  727   ......................................................................................................................................................  876
AL567209.3  940   ........K.............................................................................................................................................  791
BX440470.1  982   ........R.............................................................................................................................................  833
BU902062.1  738   ..................................................................................................................................C..G................  887

Query       902   TTGCTCCAGTCAACGTTACAACGGAAGTAAAATCTGTCGAAATGCACCATGAAGCTTTGAGTGAAGCTCTTCCTGGGGACAATGTGGGCTTCAATGTCAAGAATGTGTCTGTCAAGGATGTTCGTCGTGGCAACGTTGCTGGTGACAGCA  1051
DC527884.1  909   ......................................................................................................................................................  1058
BY794942.2  906   ......................................................................................................................................................  1055
CF111220.1  902   .............T..A.....T.....C..G.....G................................G..............A........C..A.....C...........A..C..A.A.........T........G.......  1051
BX426149.2  820   ......................................................................................................................................................  671
CO001006.1  866   ....C........T..A.....T..G..G..G..C..A.............................C...................................C...........A.....C...........T..G.............  717
BX418564.2  830   ...............................................................................................................C......................................  681
BX426150.2  877   ...................................................................................................................................-...............-..  1025
                                                                                            \
                                                                                            |
                                                                                            G
AL567209.3  790   ......................................................................................................................................................  641
BX440470.1  832   ......................................................................................................................................................  683
BU902062.1  888   ............................-..................................................AC...........C...C..........................C..C.......................  1037
                                                                                                                                                  \
                                                                                                                                                  |
                                                                                                                                                  G

Query       1052  AAAATGACCCACCAATGGAAGCAGCTGGCTTCACTGCTCAGGTGATTATCCTGAACCATCCAGGCCAAATAAGCGCCGGCTATGCCCCTGTATTGGATTGCCACACGGCTCACATTGCATGCAAGTTTGCTGAGCTGAAGGAAAAGATTG  1201
DC527884.1  1059  ......................................................................................................................................................  1208
BY794942.2  1056  ......................................................................................................................................................  1206
```

```
                                                                             \
                                                                             |
                                                                             G
CF111220.1  1052  ...................................................................G..C..T..T..............TC....C...........C.....A..............C.....T..A..G.....C.  1201
BX426149.2  670   ......................................................................................................................................................  521
CO001006.1  716   .......T.....C........T......C....A..............T....................C..T.GT..A.....A.....GC.......T.....A...........T.......................G.......  567
BX418564.2  680   ................................................................................................................................................M.....  531
BX426150.2  1026  .........-.........-.........-...................-..................-.......-.......-................-......T.-.............                            1140
AL567209.3  640   ..-...................................................................................................................................................  492
BX440470.1  682   ......................................................................................................................................................  532
                                                                                                                                                                    \
                                                                                                                                                                    |
                                                                                                                                                                    C
BU902062.1  1038  .......................C.G......-.........-.........................T....-.....G.......-..A...........C..G..T...-..............                         1163
                                                       \                          \                \              \
                                                       |                          |                |              |
                                                       C                          G                T              A

Query       1202  ATCGCCGTTCTGGTAAAAAGCTGGAAGATGGCCCTAAATTCTTGAAGTCTGGTGATGCTGCCATTGTTGATATGGTTCCTGGCAAGCCCATGTGTGTTGAGAGCTTCTCAGACTATCCACCTTTGGGTCGCTTTGCTGTTCGTGATATGA  1351
DC527884.1  1209  ......................................................................................................................................................  1358
BY794942.2  1207  ......................................................................................................................................................  1356
CF111220.1  1202  ....T...........G.................C...................................C.....C.......................A........T.....C..T..AC.T.....T..............C....  1351
BX426149.2  520   ......................................................................................................................................................  371
CO001006.1  566   ....T........G................................A........C........C.......................T........C........T..T..T.....T..C.....C..T........G.....C....  417
BX418564.2  530   ......................................................................................................................................................  381
AL567209.3  491   ............................................................................K.........................................................................  342
BX440470.1  531   ......................................................................................................................................................  382

Query       1352  GACAGACAGTTGCGGTGGGTGTCATCAAAGCAGTGGACAAGAAGGCTGCTGGAGCTGGCAAGGTCACCAAGTCTGCCCAGAAAGCTCAGAAGGCTAAATGAATATTATCCCTAATACCTGCCACCCCACTCTTAATCAGTGGTGGAAGAA  1501
DC527884.1  1359  ....................A...............................................................................................................C.................  1508
BY794942.2  1357  ......................................................................................................................................................  1506
CF111220.1  1352  .G...........T.................C.................A...........A....................................................C.............G.....................  1501
BX426149.2  370   ......................................................................................................................................................  221
CO001006.1  416   ..........C..T................................A................................................................C................G.....................  267
BX418564.2  380   ......................................................................................................................................................  231
AL567209.3  341   .......................................................................................M......Y.......................................................  191
                                                                                                                                                               \
                                                                                                                                                               |
                                                                                                                                                               A
BX440470.1  381   ......................................................................................................................................................  232

Query       1502  CGGTCTCAGAACTGTTTGTTTCAATTGGCCATTTAAGTTTAGTAGTAAAAGACTGGTTAATGATAACAATGCATCGTAAAACCTTCAGAAGGAAAGGAGAATgttttgtggaccactttggttttcttttttgcgtgtggcagttttaag  1651
DC527884.1  1509  ......................................................................................................................................................  1658
BY794942.2  1507  ......................................................................................................................................................  1656
CF111220.1  1502  ...................C.....................A....................................................---..................-...--....-..G.----................  1640
BX426149.2  220   ......................................................................................................................................................  71
CO001006.1  266   ...................C.....................A.........................................................................-.A..-....G.-----.--........-......  126
                                                                                                                             \
                                                                                                                             |
                                                                                                                             T
BX418564.2  230   ......................................................................................................................................................  81
AL567209.3  190   ......................................................................................................................................................  40
                                                                                                                                   \
                                                                                                                                   |
                                                                                                                                   G
BX440470.1  231   ......................................................................................................................................................  82

Query       1652  ttattagtttttaaaatcagtactttttaATGGAAACAACTTGACCAAAAATTTGTCACAGAATTTTGAGACCCATTAAAAAAGTTAAATG  1742
DC527884.1  1659  ...........................................................................................  1749
BY794942.2  1657  ...........................................................................................  1747
CF111220.1  1641  ...........C........................................C....................-............T....  1731
                                                                                                   \
                                                                                                   |
                                                                                                   C
BX426149.2  70    ................................-.......-.....G......G.-..G...........T..                    1
CO001006.1  125   ................................A...................C.............-...................T....  35
                                                                                                     \
                                                                                                     |
                                                                                                     A
BX418564.2  80    ..................................................................M-.............            1
AL567209.3  39    ...........................-....-..-...-...                                                  1
BX440470.1  81    .................................................................................            1
```

Query= T3-23 [organism=Homo sapiens] eef1a1

Length=1643

```
                                                                 Score        E     Max
Sequences producing significant alignments:                      (Bits)     Value  ident

BY794942.2 BY794942 Homo sapiens eye Homo sapiens cDNA clone H...  2468       0.0    100%
```

```
DC527884.1 DC527884 chimpanzee skin cDNA library PstA Pan trog...  2462       0.0    100%
BX426149.2 BX426149 Homo sapiens NEUROBLASTOMA Homo sapiens cD...  2146       0.0    99%
BX418564.2 BX418564 Homo sapiens FETAL BRAIN Homo sapiens cDNA...  2047       0.0    97%
AL567209.3 AL567209 Homo sapiens FETAL BRAIN Homo sapiens cDNA...  2001       0.0    98%
BX440470.1 BX440470 Homo sapiens FETAL BRAIN Homo sapiens cDNA...  1999       0.0    98%
BX420340.1 BX420340 Homo sapiens FETAL BRAIN Homo sapiens cDNA...  1997       0.0    99%
AL518238.2 AL518238 Homo sapiens NEUROBLASTOMA Homo sapiens cD...  1993       0.0    98%
BX459625.2 BX459625 Homo sapiens FETAL BRAIN Homo sapiens cDNA...  1993       0.0    99%
BX418589.2 BX418589 Homo sapiens FETAL BRAIN Homo sapiens cDNA...  1980       0.0    98%

ALIGNMENTS

Query       2     TTTTTCGCAACGGGTTTGCCGCCAGAACACAGGTGTCGTGAAAACTACCCCTAAAAGCCAAAATGGGAAAGGAAAAGACTCATATCAACATTGTCGTCATTGGACACGTAGATTCGGGCAAGTCCACCACTACTGGCCATCTGATCTATA  151
BY794942.2  25                       ...................................................................................................................................  155
DC527884.1  9     ......................................................................................................................................................  158

Query       152   AATGCGGTGGCATCGACAAAAGTAGCCTGGCTTGGATAAACTGAAAGCGAGCGTGAACGTGGTATCACCATTGATATCTCCTTGTGGAAATTTGAGACCAGCAGTACTATGTGACTATCATTGATGCCCCAGGACACAGAGACTTATCAA  301
BY794942.2  156   ......................                                                                                                                                  177
BY794942.2  230                      ...................................................................................................................................  366
                                      \   \ \                 \                                                     \                                          \
                                      |   | |                 |                                                     |                                          |
                                      T   G T                 T                                                     A                                          T
DC527884.1  159   ......................                                                                                                                                  180
DC527884.1  233                      ..............................................T....................................................................................  369
                                      \   \ \                 \                                                     \                                          \
                                      |   | |                 |                                                     |                                          |
                                      T   G T                 T                                                     A                                          C

Query       302   ACATGATTGTTGCTGCTGGTGTTGGTGAATTTGAAGCTGGTATCTCCAAGAATGGGCAGACCCGAGAGCATGCCCTTCTGGCTTACACACTGGGTGTGAAACAACTAATTGTCGGTGTTAACAAAATGGATTCCACTGAGCCACCCTACA  451
BY794942.2  408      ...................................................................................................................................................  554
BY794942.2  367   ........                                                                                                                                                376
                   \
                   |
                  AA
DC527884.1  411      ...................................................................................................................................................  557
DC527884.1  370   ........                                                                                                                                                379
                   \
                   |
                  AA
BX426149.2  1196                                                                                                                              ......-..A....-.A.........  1172
BX418564.2  1196                                                                                                                        ....-....-......-...-...-...YH.V  1170
AL567209.3  1145                                                                                                                                                 .......  1139

Query       452   GCCAGAAGAGATATGAGGAAATTGTTAAGGAAGTCAGCACTTACATTAAGAAAATTGGCTACAACCCCGACACAGTAGCATTTGTGCCAATTTCTGGTTGGAATGGTGACAACATGCTGGAGCCAAGTGCTAACATGCCTTGGTTCAAGG  601
BY794942.2  555   ......................................................................................................................................................  704
DC527884.1  558   ......................................................................................................................................................  707
BX426149.2  1171  .....G................................................................................................................................................  1022
BX418564.2  1169  S..-.R......-W.-..G..W....-.R......M.....K....-.R.-......S....-..........-R.....-.................-.....-..........................C.................  1031
AL567209.3  1138  S.....-...W.-..........-K.W........R.....-....A.M.C...........C.......................................................................................  992
                                                              \
                                                              |
                                                              M
BX440470.1  1128                                                ....-.....-..-W.-............-...-....-S.M...Y..-.K......-..............-....................K........  1034
                                                                                     \
                                                                                     |
                                                                                     C
BX420340.1  1117                                                         ...M..M..................K.-..M.W...YT...D....K.-...........T..........Y...................  1027
AL518238.2  1148                         .....R..-....-.M.....-..........-..W...M....V.M........................-K.........A...........A............................  1028
BX459625.2  1118                                                   ........C.MCM....C.....K....W...-KS.......-.K......W......................................K.A.....  1021
                                                                                                                              \
                                                                                                                              |
                                                                                                                              T
BX418589.2  1130                                             ....-........-.K..-W.-M....C..M..K.-...-....S....-....-........-..............-......-KK............K.C.....  1033

Query       602   GATGGAAAGTCACCCGTAAGGATGGCAATGCCAGTGGAACCACGCTGCTTGAGGCTCTGGACTGCATCCTACCACCAACTCGTCCAACTGACAAGCCCTTGCGCCTGCCTCTCCAGGATGTCTACAAAATTGGTGGTATTGGTACTGTTC  751
BY794942.2  705   ......................................................................................................................................................  854
DC527884.1  708   ......................................................................................................................................................  857
BX426149.2  1021  ......................................................................................................................................................  872
BX418564.2  1030  ....-.........................................................................................C.......................................................  882
AL567209.3  991   ..........................................................K...........................................................................................  842
BX440470.1  1033  ..........................................................R...........................................................................................  884
BX420340.1  1026  ..........................................................A..............................R............................................................  877
AL518238.2  1027  ......W...........................R...................................................................................................................  877
                                     \
                                     |
                                     A
BX459625.2  1020  ......................................................................................................................................................  871
BX418589.2  1032  ......................................................................................................................................................  883

Query       752   CTGTTGGCCGAGTGGAGACTGGTGTTCTCAAACCCGGTATGGTGGTCACCTTTGCTCCAGTCAACGTTACAACGGAAGTAAAATCTGTCGAAATGCACCATGAAGCTTTGAGTGAAGCTCTTCCTGGGGACAATGTGGGCTTCAATGTCA  901
BY794942.2  855   ......................................................................................................................................................  1004
DC527884.1  858   ......................................................................................................................................................  1007
```

```
BX426149.2  871   ......................................................................................................................................................  722
BX418564.2  881   ......................................................................................................................................................  732
AL567209.3  841   ......................................................................................................................................................  692
BX440470.1  883   ......................................................................................................................................................  734
BX420340.1  876   ......................................................................................................................................................  727
AL518238.2  876   ................W...................................B.............................W...................................................................  727
BX459625.2  870   ......................................................................................................................................................  721
BX418589.2  882   ......................................................................................................................................................  733

Query       902   AGAATGTGTCTGTCAAGGATGTTCGTCGTGGCAACGTTGCTGGTGACAGCAAAAATGACCCACCAATGGAAGCAGCTGGCTTCACTGCTCAGGTGATTATCCTGAACCATCCAGGCCAAATAAGCGCCGGCTATGCCCCTGTATTGGATT  1051
BY794942.2  1005  ......................................................................................................................................................  1155
                                                                                                                                  \
                                                                                                                                  |
                                                                                                                                  G
DC527884.1  1008  ......................................................................................................................................................  1157
BX426149.2  721   ......................................................................................................................................................  572
BX418564.2  731   ............C.........................................................................................................................................  582
AL567209.3  691   ......................................................-...............................................................................................  543
BX440470.1  733   ......................................................................................................................................................  584
BX420340.1  726   ......................................................................................................................................................  577
AL518238.2  726   ............................................A............................G............................................................................  577
BX459625.2  720   ......................................................................................................................................................  571
BX418589.2  732   ......................................................................................................................................................  583

Query       1052  GCCACACGGCTCACATTGCATGCAAGTTTGCTGAGCTGAAGGAAAAGATTGATCGCCGTTCTGGTAAAAAGCTGGAAGATGGCCCTAAATTCTTGAAGTCTGGTGATGCTGCCATTGTTGATATGGTTCCTGGCAAGCCCATGTGTGTTG  1201
BY794942.2  1156  ......................................................................................................................................................  1305
DC527884.1  1158  ......................................................................................................................................................  1307
BX426149.2  571   ......................................................................................................................................................  422
BX418564.2  581   .............................................M........................................................................................................  432
AL567209.3  542   ...............................................................................................................................K......................  393
BX440470.1  583   ......................................................................................................................................................  433
                                                                 \
                                                                 |
                                                                 C
BX420340.1  576   ......................................................................................................................................................  427
AL518238.2  576   ................Y.....................................................................................................................................  427
BX459625.2  570   ......................................................................................................................................................  421
BX418589.2  582   ....................................................Y.S...............................................................................................  433

Query       1202  AGAGCTTCTCAGACTATCCACCTTTGGGTCGCTTTGCTGTTCGTGATATGAGACAGACAGTTGCGGTGGGTGTCATCAAAGCAGTGGACAAGAAGGCTGCTGGAGCTGGCAAGGTCACCAAGTCTGCCCAGAAAGCTCAGAAGGCTAAAT  1351
BY794942.2  1306  ......................................................................................................................................................  1455
DC527884.1  1308  .......................................................................A..............................................................................  1457
BX426149.2  421   ......................................................................................................................................................  272
BX418564.2  431   ......................................................................................................................................................  282
AL567209.3  392   ..........................................................................................................................................M......Y....  243
BX440470.1  432   ......................................................................................................................................................  283
BX420340.1  426   ......................................................................................................................................................  277
AL518238.2  426   .......................................................................................................G..............................................  277
BX459625.2  420   ......................................................................................................................................................  271
BX418589.2  432   ......................................................................................................................................................  283

Query       1352  GAATATTATCCCTAATACCTGCCACCCCACTCTTAATCAGTGGTGGAAGAACGGTCTCAGAACTGTTTGTTTCAATTGGCCATTTAAGTTTAGTAGTAAAAGACTGGTTAATGATACAATGCATCGTAAAACCTTCAGAAGGAAAGGAG  1501
BY794942.2  1456  ......................................................................................................................................................  1605
DC527884.1  1458  .................................C....................................................................................................................  1607
BX426149.2  271   ......................................................................................................................................................  122
BX418564.2  281   ......................................................................................................................................................  132
AL567209.3  242   ......................................................................................................................................................  92
                                                            \
                                                            |
                                                            A
BX440470.1  282   ......................................................................................................................................................  133
BX420340.1  276   ......................................................................................................................................................  127
AL518238.2  276   ............................................................G.........................................................................................  127
BX459625.2  270   ......................................................................................................................................................  121
BX418589.2  282   ......................................................................................................................................................  133

Query       1502  AATgttttgtggaccactttggttttctttttttgcgtgtggcagttttaagttattagtttttaaaatcagtactttttaATGGAAACAACTTGACCAAAAATTTGTCACAGAATTTTGAGACCCATTAAAAAAGTTAAATG  1643
BY794942.2  1606  ..............................................................................................................................................  1747
DC527884.1  1608  ..............................................................................................................................................  1749
BX426149.2  121   ....................................................................................-.......-.....G......G.-..G..........T..                    1
BX418564.2  131   .....................................................................................................................M-.............            1
AL567209.3  91    ...............................................................................-....-..-...-...                                                1
                                \
                                |
                                G
BX440470.1  132   ....................................................................................................................................            1
BX420340.1  126   ........................................................................................................................R.....                  1
AL518238.2  126   .........................................................................................N.............N.....................                 1
                                                                                                                             \
                                                                                                                             |
                                                                                                                             N
BX459625.2  120   ........................................................................................................................                        1
```

```
BX418589.2  132   .....................................................................................................................D.............           1

Query= T4-32 [organism=Homo sapiens] eef1a1

Length=1203


                                                                  Score        E     Max
Sequences producing significant alignments:                      (Bits)     Value  ident

BY794942.2 BY794942 Homo sapiens eye Homo sapiens cDNA clone H...  1757       0.0    100%
DC527884.1 DC527884 chimpanzee skin cDNA library PstA Pan trog...  1749       0.0    100%
AL541984.3 AL541984 Homo sapiens PLACENTA Homo sapiens cDNA cl...  1749       0.0    100%
BX440470.1 BX440470 Homo sapiens FETAL BRAIN Homo sapiens cDNA...  1738       0.0    100%
BX420402.2 BX420402 Homo sapiens FETAL BRAIN Homo sapiens cDNA...  1736       0.0    100%
BX440308.2 BX440308 Homo sapiens FETAL BRAIN Homo sapiens cDNA...  1735       0.0    100%
BX440436.2 BX440436 Homo sapiens FETAL BRAIN Homo sapiens cDNA...  1735       0.0    100%
BX325185.1 BX325185 Homo sapiens T CELLS (JURKAT CELL LINE) CO...  1733       0.0    100%
BX440303.2 BX440303 Homo sapiens FETAL BRAIN Homo sapiens cDNA...  1731       0.0    100%
BX418589.2 BX418589 Homo sapiens FETAL BRAIN Homo sapiens cDNA...  1731       0.0    100%

ALIGNMENTS

Query       2     TTTTTCGCAACGGGTTTGCCGCCAGAACACAGGTGTCGTGAAAACTACCCCTAAAAGCCAAAATGGGAAAGGAAAAGACTCATATCAACATTGTCGTCATTGGACACGTAGATTCGGGCAAGTCCACCACTACTGGCCATCTGATCTATA  151
BY794942.2  25                       ...................................................................................................................................  155
DC527884.1  9     ......................................................................................................................................................  158

Query       152   AATGCGGTGGCATCGACAAAAGAACCATTGAAAAATTTGAGAAGGAGGCTGCTGAGATGGGAAAGGGCTCCTTCAAGTATGCCTGGGTCTTGGATAAACTGACAAGCCCTTGCGCCTGCCTCTCCAGGATGTCTACAAAATTGGTGGTAT  301
BY794942.2  790                                                                                                   ......................................................  843
BY794942.2  156   ......................................................................................................-....                                             261
DC527884.1  793                                                                                                   ......................................................  846
DC527884.1  159   ......................................................................................................-....                                             264
AL541984.3  59                                                                                                    ......................................................  112
BX440470.1  948                                                                                                   ......................................................  895
BX420402.2  944                                                                                                   ......................................................  891
BX440308.2  947                                                                                                   ......................................................  894
BX440436.2  950                                                                                                   .......R..............................................  897
BX325185.1  946                                                                                                   ....R.................................................  893
BX440303.2  946                                                                                                   ......................................................  893
BX418589.2  947                                                                                                   ......................................................  894

Query       302   TGGTACTGTTCCTGTTGGCCGAGTGGAGACTGGTGTTCTCAAACCCGGTATGGTGGTCACCTTTGCTCCAGTCAACGTTACAACGGAAGTAAAATCTGTCGAAATGCACCATGAAGCTTTGAGTGAAGCTCTTCCTGGGGACAATGTGGG  451
BY794942.2  844   ......................................................................................................................................................  993
DC527884.1  847   ......................................................................................................................................................  996
AL541984.3  113   ......................................................................................................................................................  262
BX440470.1  894   ......................................................................................................................................................  745
BX420402.2  890   ......................................................................................................................................................  741
BX440308.2  893   ......................................................................................................................................................  744
BX440436.2  896   ......................................................................................................................................................  747
BX325185.1  892   ......................................................................................................................................................  743
BX440303.2  892   ......................................................................................................................................................  743
BX418589.2  893   ......................................................................................................................................................  744

Query       452   CTTCAATGTCAAGAATGTGTCTGTCAAGGATGTTCGTCGTGGCAACGTTGCTGGTGACAGCAAAAATGACCCACCAATGGAAGCAGCTGGCTTCACTGCTCAGGTGATTATCCTGAACCATCCAGGCCAAATAAGCGCCGGCTATGCCCC  601
BY794942.2  994   ......................................................................................................................................................  1144
                                                                                                                           \
                                                                                                                           |
                                                                                                                           G
DC527884.1  997   ......................................................................................................................................................  1146
AL541984.3  263   ......................................................................................................................................................  412
BX440470.1  744   ......................................................................................................................................................  595
BX420402.2  740   ......................................................................................................................................................  591
BX440308.2  743   ..................................................................................................................................M...................  594
BX440436.2  746   ......................................................................................................................................................  597
BX325185.1  742   ......................................................................................................................................................  593
BX440303.2  742   ..........................................................C...........................................................................................  593
BX418589.2  743   ......................................................................................................................................................  594

Query       602   TGTATTGGATTGCCACACGGCTCACATTGCATGCAAGTTTGCTGAGCTGAAGGAAAAGATTGATCGCCGTTCTGGTAAAAAGCTGGAAGATGGCCCTAAATTCTTGAAGTCTGGTGATGCTGCCATTGTTGATATGGTTCCTGGCAAGCC  751
BY794942.2  1145  ......................................................................................................................................................  1294
DC527884.1  1147  ......................................................................................................................................................  1296
AL541984.3  413   ......................................................................................................................................................  562
BX440470.1  594   ......................................................................................................................................................  444
                                                            \
                                                            |
                                                            C
BX420402.2  590   ......................................................................................................................................................  441
BX440308.2  593   ......................................................................................................................................................  444
BX440436.2  596   ......................................................................................................................................................  447
BX325185.1  592   ...........................Y..........................................................................................................................  443
BX440303.2  592   ......................................................................................................................................................  443
BX418589.2  593   ...............................................................Y.S....................................................................................  444
```

```
Query       752   CATGTGTGTTGAGAGCTTCTCAGACTATCCACCTTTGGGTCGCTTTGCTGTTCGTGATATGAGACAGACAGTTGCGGTGGGTGTCATCAAAGCAGTGGACAAGAAGGCTGCTGGAGCTGGCAAGGTCACCAAGTCTGCCCAGAAAGCTCA  901
BY794942.2  1295  ......................................................................................................................................................  1444
DC527884.1  1297  ..................................................................................A...................................................................  1446
AL541984.3  563   ......................................................................................................................................................  712
BX440470.1  443   ......................................................................................................................................................  294
BX420402.2  440   ......................................................................................................................................................  291
BX440308.2  443   ......................................................................................................................................................  294
BX440436.2  446   ......................................................................................................................................................  297
BX325185.1  442   ......................................................................................................................................................  293
BX440303.2  442   ......................................................................................................................................................  293
BX418589.2  443   ......................................................................................................................................................  294

Query       902   GAAGGCTAAATGAATATTATCCCTAATACCTGCCACCCCACTCTTAATCAGTGGTGGAAGAACGGTCTCAGAACTGTTTGTTTCAATTGGCCATTTAAGTTTAGTAGTAAAAGACTGGTTAATGATAACAATGCATCGTAAAACCTTCAG  1051
BY794942.2  1445  ......................................................................................................................................................  1594
DC527884.1  1447  ............................................C.........................................................................................................  1596
AL541984.3  713   ......................................................................................................................................................  862
BX440470.1  293   ......................................................................................................................................................  144
BX420402.2  290   ......................................................................................................................................................  141
BX440308.2  293   ......................................................................................................................................................  144
BX440436.2  296   ......................................................................................................................................................  147
BX325185.1  292   ......................................................................................................................................................  143
BX440303.2  292   ......................................................................................................................................................  143
BX418589.2  293   ......................................................................................................................................................  144

Query       1052  AAGGAAAGGAGAATGTTTTGTGGACCACtttggttttctttttgcgtgtggcagttttaagttattagtttttaaaatcagtactttttaATGGAAACAACTTGACCAAAAATTTGTCACAGAATTTTGAGACCCATTAAAAAAGTTAAA  1201
BY794942.2  1595  ......................................................................................................................................................  1745
                                                        \
                                                        |
                                                        T
DC527884.1  1597  ......................................................................................................................................................  1747
                                                        \
                                                        |
                                                        T
AL541984.3  863   .................................................K.S..................................................................................................  1014
                                                        \                                                                                 \
                                                        |                                                                                 |
                                                        T                                                                                 M
BX440470.1  143   ..............................................................................................................................................          1
                                                             \
                                                             |
                                                             T
BX420402.2  140   ...........................................................................................................................................             1
                                                             \
                                                             |
                                                             T
BX440308.2  143   ................................................................................................................................-..............         1
                                                             \
                                                             |
                                                             T
BX440436.2  146   .....................................................................................................................N..........................        1
                                                             \                                                            \
                                                             |                                                            |
                                                             T                                                            N
BX325185.1  142   .............................................................................................................................................           1
                                                             \
                                                             |
                                                             T
BX440303.2  142   ................................................................................................................................-.............          1
                                                             \
                                                             |
                                                             T
BX418589.2  143   ................................................................................................................................D.............          1
                                                             \
                                                             |
                                                             T

Query       1202  TG  1203
BY794942.2  1746  ..  1747
DC527884.1  1748  ..  1749
AL541984.3  1015  ..  1016
```

Query= T5-24 [organism=Homo sapiens] eef1a1

Length=822

```
                                                                  Score        E     Max
Sequences producing significant alignments:                      (Bits)     Value  ident

CR979370.1 CR979370 RZPD no.9017 Homo sapiens cDNA clone RZPDp...  905        0.0    100%
CD522692.1 AGENCOURT_14354560 NIH_MGC_191 Homo sapiens cDNA cl...  905        0.0    100%
```

```
BU581210.1 in37g01.x1 Human Fetal Pancreas 1B Homo sapiens cDN...  905        0.0    100%
BU532443.1 AGENCOURT_10200888 NIH_MGC_126 Homo sapiens cDNA cl...  905        0.0    100%
BU158477.1 AGENCOURT_7937274 NIH_MGC_92 Homo sapiens cDNA clon...  905        0.0    100%
BM993568.1 UI-H-DT0-avj-h-01-0-UI.s1 NCI_CGAP_DT0 Homo sapiens...  905        0.0    100%
AU147847.1 AU147847 MAMMA1 Homo sapiens cDNA clone MAMMA100186...  905        0.0    100%
BE677612.1 7d88f01.x1 Lupski_dorsal_root_ganglion Homo sapiens...  905        0.0    100%
CN389382.1 17000600087635 GRN_PRENEU Homo sapiens cDNA 5', mRN...  904        0.0    100%
CX868104.1 HESC4_26_D10.b1_A037 NIH_MGC_262 Homo sapiens cDNA ...  902        0.0    100%

ALIGNMENTS

Query       333  TGATGCTGCCATTGTTGATATGGTTCCTGGCAAGCCCATGTGTGTTGAGAGCTTCTCAGACTATCCACCTTTGGGTCGCTTTGCTGTTCGTGATATGAGACAGACAGTTGCGGTGGGTGTCATCAAAGCAGTGGACAAGAAGGCTGCTGG  482
CR979370.1  235  ......................................................................................................................................................  384
CD522692.1  74   ......................................................................................................................................................  223
BU581210.1  492  ......................................................................................................................................................  343
BU532443.1  85   ......................................................................................................................................................  234
BU158477.1  256  ......................................................................................................................................................  405
BM993568.1  508  ......................................................................................................................................................  359
AU147847.1  492  ......................................................................................................................................................  343
BE677612.1  501  ......................................................................................................................................................  352
CN389382.1  145  ......................................................................................................................................................  294
CX868104.1  515  ......................................................................................................................................................  366

Query       483  AGCTGGCAAGGTCACCAAGTCTGCCCAGAAAGCTCAGAAGGCTAAATGAATATTATCCCTAATACCTGCCACCCCACTCTTAATCAGTGGTGGAAGAACGGTCTCAGAACTGTTTGTTTCAATTGGCCATTTAAGTTTAGTAGTAAAAGA  632
CR979370.1  385  ......................................................................................................................................................  534
CD522692.1  224  ......................................................................................................................................................  373
BU581210.1  342  ......................................................................................................................................................  193
BU532443.1  235  ......................................................................................................................................................  384
BU158477.1  406  ......................................................................................................................................................  555
BM993568.1  358  ......................................................................................................................................................  209
AU147847.1  342  ......................................................................................................................................................  193
BE677612.1  351  ......................................................................................................................................................  202
CN389382.1  295  ......................................................................................................................................................  444
CX868104.1  365  ......................................................................................................................................................  216

Query       633  CTGGTTAATGATAACAATGCATCGTAAAACCTTCAGAAGGAAAGGAGAATgttttgtggaccactttggttttctttttttgcgtgtggcagttttaagttattagtttttaaaatcagtactttttaATGGAAACAACTTGACCAAAAAT  782
CR979370.1  535  ......................................................................................................................................................  684
CD522692.1  374  ......................................................................................................................................................  523
BU581210.1  192  ......................................................................................................................................................  43
BU532443.1  385  ......................................................................................................................................................  534
BU158477.1  556  ......................................................................................................................................................  705
BM993568.1  208  ......................................................................................................................................................  59
AU147847.1  192  ......................................................................................................................................................  43
BE677612.1  201  ......................................................................................................................................................  52
CN389382.1  445  ......................................................................................................................................................  594
CX868104.1  215  ......................................................................................................................................................  66

Query       783  TTGTCACAGAATTTTGAGACCCATTaaaaaaaGTTAAATG  822
CR979370.1  685  ........................................  724
CD522692.1  524  ........................................  563
BU581210.1  42   ........................................  3
BU532443.1  535  ........................................  574
BU158477.1  706  ........................................  745
BM993568.1  58   ........................................  19
AU147847.1  42   ........................................  3
BE677612.1  51   ........................................  12
CN389382.1  595  .......................................   633
CX868104.1  65   .....................................N..  26

Query= T6-31 [organism=Homo sapiens] eef1a1

Length=1168


                                                                   Score        E     Max
Sequences producing significant alignments:                       (Bits)     Value  ident

BY794942.2 BY794942 Homo sapiens eye Homo sapiens cDNA clone H...  1855       0.0    99%
DC527884.1 DC527884 chimpanzee skin cDNA library PstA Pan trog...  1847       0.0    99%
BX440470.1 BX440470 Homo sapiens FETAL BRAIN Homo sapiens cDNA...  1832       0.0    99%
AL541984.3 AL541984 Homo sapiens PLACENTA Homo sapiens cDNA cl...  1831       0.0    99%
BX418589.2 BX418589 Homo sapiens FETAL BRAIN Homo sapiens cDNA...  1829       0.0    99%
BX442182.2 BX442182 Homo sapiens FETAL BRAIN Homo sapiens cDNA...  1823       0.0    99%
BX459625.2 BX459625 Homo sapiens FETAL BRAIN Homo sapiens cDNA...  1818       0.0    100%
BX420340.1 BX420340 Homo sapiens FETAL BRAIN Homo sapiens cDNA...  1816       0.0    99%
BX418564.2 BX418564 Homo sapiens FETAL BRAIN Homo sapiens cDNA...  1814       0.0    99%
BX325185.1 BX325185 Homo sapiens T CELLS (JURKAT CELL LINE) CO...  1810       0.0    99%

ALIGNMENTS

Query       1     TGGTGTTGGTGAATTTGAAGCTGGTATCTCCAAGAATGGGCAGACCCGAGAGCATGCCCTTCTGGCTTACACACTGGGTGTGAAACAACTAATTGTCGGTGTTAACAAAATGGATTCCACTGAGCCACCCTACAGCCAGAAGAGATATGA  150
BY794942.2  723                                                                                                                                                        .  723
```

```
BY794942.2  421   ......................................................................................................................................................  570
DC527884.1  726                                                                                                                                                        .  726
DC527884.1  424   ......................................................................................................................................................  573
BX440470.1  1015                                                                                                                                                       .  1015
BX418589.2  1014                                                                                                                                                       .  1014
BX442182.2  1007                                                                                                                                                       .  1007
BX459625.2  1002                                                                                                                                                       .  1002
BX420340.1  1008                                                                                                                                                       .  1008
BX418564.2  1013                                                                                                                                                       .  1013
BX325185.1  1013                                                                                                                                                       .  1013

Query       151   GGATGGCAATGCCAGTGGAACCACGCTGCTTGAGGCTCTGGACTGCATCCTACACCAACTCGTCCAACTGACAAGCCCTTGCGCCTGCCTCTCCAGGATGTCTACAAAATTGGTGGTATTGGTACTGTTCCTGTTGGCCGAGTGGAGACT  300
BY794942.2  724   ......................................................................................................................................................  874
                                                                      \
                                                                      |
                                                                      C
BY794942.2  571   ...                                                                                                                                                     573
DC527884.1  727   ......................................................................................................................................................  877
                                                                      \
                                                                      |
                                                                      C
DC527884.1  574   ...                                                                                                                                                     576
BX440470.1  1014  ........................................R.............................................................................................................  864
                                                                       \
                                                                       |
                                                                       C
AL541984.3  1             ..............................................................................................................................................  143
                                                                      \
                                                                      |
                                                                      C
BX418589.2  1013  ......................................................................................................................................................  863
                                                                       \
                                                                       |
                                                                       C
BX442182.2  1006  ..................................................................................................................................................R...  856
                                                                       \
                                                                       |
                                                                       C
BX459625.2  1001  ......................................................................................................................................................  851
                                                                       \
                                                                       |
                                                                       C
BX420340.1  1007  ........................................A............................R................................................................................  857
                                                                       \
                                                                       |
                                                                       C
BX418564.2  1012  ..............................................................................C.......................................................................  862
                                                                       \
                                                                       |
                                                                       C
BX325185.1  1012  ...............K.R.M..........K.........A............................R................................................................................  862
                                                                       \
                                                                       |
                                                                       C

Query       301   GGTGTTCTCAAACCGGTATGGTGGTCACCTTTGCTCCAGTCAACGTTACAACGAAGTAAAATCTGTCGAAATGCACCATGAAGCTTTGAGTGAAGCTCTTCCTGGGGACAATGTGGGCTTCAATGTCAAGAATGTGTCTGTCAAGGATGT  450
BY794942.2  875   ......................................................................................................................................................  1026
                              \                                       \
                              |                                       |
                              C                                       G
DC527884.1  878   ......................................................................................................................................................  1029
                              \                                       \
                              |                                       |
                              C                                       G
BX440470.1  863   ......................................................................................................................................................  712
                                \                                      \
                                |                                      |
                                C                                      G
AL541984.3  144   ......................................................................................................................................................  295
                              \                                       \
                              |                                       |
                              C                                       G
BX418589.2  862   ......................................................................................................................................................  711
                                \                                      \
                                |                                      |
                                C                                      G
BX442182.2  855   ......................................................................................................................................................  704
                                \                                      \
                                |                                      |
                                C                                      G
BX459625.2  850   ......................................................................................................................................................  699
                                \                                      \
```

```
                                |                                      |
                                C                                      G
BX420340.1  856   ......................................................................................................................................................  705
                                \                                      \
                                |                                      |
                                C                                      G
BX418564.2  861   .............................................................................................................................................C........  710
                                \                                      \
                                |                                      |
                                C                                      G
BX325185.1  861   ......................................................................................................................................................  710
                                \                                      \
                                |                                      |
                                C                                      G

Query       451   TCGTCGTGGCAACGTTGCTGGTGACAGCAAAAATGACCACCAATGGAAGCAGCTGGCTTCACTGCTCAGGTGATTATCCTGAACCATCCAGGCCAAATAAGCGCCGGCTATGCCCTGTATTGGATTGCCACACGGCTCACATTGCATGCA  600
BY794942.2  1027  ......................................................................................................................................................  1179
                                                      \                                                  \                        \
                                                      |                                                  |                        |
                                                      C                                                  G                        C
DC527884.1  1030  ......................................................................................................................................................  1181
                                                      \                                                                           \
                                                      |                                                                           |
                                                      C                                                                           C
BX440470.1  711   ......................................................................................................................................................  560
                                                        \                                                                            \
                                                        |                                                                            |
                                                        C                                                                            C
AL541984.3  296   ......................................................................................................................................................  447
                                                      \                                                                           \
                                                      |                                                                           |
                                                      C                                                                           C
BX418589.2  710   ......................................................................................................................................................  559
                                                        \                                                                            \
                                                        |                                                                            |
                                                        C                                                                            C
BX442182.2  703   ......................................................................................................................................................  552
                                                        \                                                                            \
                                                        |                                                                            |
                                                        C                                                                            C
BX459625.2  698   ......................................................................................................................................................  547
                                                        \                                                                            \
                                                        |                                                                            |
                                                        C                                                                            C
BX420340.1  704   ......................................................................................................................................................  553
                                                        \                                                                            \
                                                        |                                                                            |
                                                        C                                                                            C
BX418564.2  709   ......................................................................................................................................................  558
                                                        \                                                                            \
                                                        |                                                                            |
                                                        C                                                                            C
BX325185.1  709   ...............................................................................................................................................Y......  558
                                                        \                                                                            \
                                                        |                                                                            |
                                                        C                                                                            C

Query       601   AGTTTGCTGAGCTGAAGGAAAAGATTGATCGCCGTTCTGGTAAAAAGCTGGAAGATGGCCCTAAATTCTTGAAGTCTGGTGATGCTGCCATTGTTGATATGGTTCCTGGCAAGCCCATGTGTGTTGAGAGCTTCTCAGACTATCCACCTT  750
BY794942.2  1180  ......................................................................................................................................................  1329
DC527884.1  1182  ......................................................................................................................................................  1331
BX440470.1  559   ......................................................................................................................................................  409
                                         \
                                         |
                                         C
AL541984.3  448   ......................................................................................................................................................  597
BX418589.2  558   ............................Y.S.......................................................................................................................  409
BX442182.2  551   ......................................................................................................................................................  402
BX459625.2  546   ......................................................................................................................................................  397
BX420340.1  552   ......................................................................................................................................................  403
BX418564.2  557   .....................M................................................................................................................................  408
BX325185.1  557   ......................................................................................................................................................  408

Query       751   TGGGTCGCTTTGCTGTTCGTGATATGAGACAGACAGTTGCGGTGGGTGTCATCAAAGCAGTGGACAAGAAGGCTGCTGGAGCTGGCAAGGTCACCAAGTCTGCCCAGAAAGCTCAGAAGGCTAAATGAATATTATCCCTAATACCTGCCA  900
BY794942.2  1330  ......................................................................................................................................................  1479
DC527884.1  1332  ...............................................A......................................................................................................  1481
BX440470.1  408   ......................................................................................................................................................  259
AL541984.3  598   ......................................................................................................................................................  747
BX418589.2  408   ......................................................................................................................................................  259
BX442182.2  401   ......................................................................................................................................................  252
BX459625.2  396   ......................................................................................................................................................  247
BX420340.1  402   ......................................................................................................................................................  253
BX418564.2  407   ......................................................................................................................................................  258
```

```
BX325185.1  407   ......................................................................................................................................................  258

Query       901   CCCCACTCTTAATCAGTGGTGGAAGAACGGTCTCAGAACTGTTTGTTTCAATTGGCCATTTAAGTTTAGTAGTAAAAGACTGGTTAATGATAACAATGCATCGTAAAACCTTCAGAAGGAAAGGAGAATgttttgtggaccactttggtt  1050
BY794942.2  1480  ......................................................................................................................................................  1629
DC527884.1  1482  .........C............................................................................................................................................  1631
BX440470.1  258   ......................................................................................................................................................  109
AL541984.3  748   ......................................................................................................................................................  897
BX418589.2  258   ......................................................................................................................................................  109
BX442182.2  251   ......................................................................................................................................................  102
BX459625.2  246   ......................................................................................................................................................  97
BX420340.1  252   ......................................................................................................................................................  103
BX418564.2  257   ......................................................................................................................................................  108
BX325185.1  257   ......................................................................................................................................................  108

Query       1051  ttctttttgcgtgtggcagttttaagttattagtttttaaaatcagtactttttaATGGAAACAACTTGACCAAAAATTTGTCACAGAATTTTGAGACCCATTAAAAAAGTTAAATG  1168
BY794942.2  1630  ......................................................................................................................  1747
DC527884.1  1632  ......................................................................................................................  1749
BX440470.1  108   ............................................................................................................            1
AL541984.3  898   ...............K.S....................................................................................................  1016
                                                                                                        \
                                                                                                        |
                                                                                                        M
BX418589.2  108   .............................................................................................D..............            1
BX442182.2  101   .....................................................................................................                   1
BX459625.2  96    ................................................................................................                        1
BX420340.1  102   ................................................................................................R.....                  1
BX418564.2  107   .............................................................................................M-............             1
BX325185.1  107   ...........................................................................................................             1
```

Query= T7-25 [organism=Homo sapiens] eef1a1

Length=1553

```
                                                                    Score        E     Max
Sequences producing significant alignments:                        (Bits)     Value  ident

BY794942.2 BY794942 Homo sapiens eye Homo sapiens cDNA clone H...  2710       0.0    100%
DC527884.1 DC527884 chimpanzee skin cDNA library PstA Pan trog...  2693       0.0    100%
BX426149.2 BX426149 Homo sapiens NEUROBLASTOMA Homo sapiens cD...  2146       0.0    99%
CO001006.1 OC028 pre-oestrum adult sheep ovary library Ovis ar...  2050       0.0    93%
BX418564.2 BX418564 Homo sapiens FETAL BRAIN Homo sapiens cDNA...  2047       0.0    97%
CF111220.1 Shultzomica04471 Rat lung airway and parenchyma cDN...  2034       0.0    92%
AL567209.3 AL567209 Homo sapiens FETAL BRAIN Homo sapiens cDNA...  2001       0.0    98%
BX440470.1 BX440470 Homo sapiens FETAL BRAIN Homo sapiens cDNA...  1999       0.0    98%
BX420340.1 BX420340 Homo sapiens FETAL BRAIN Homo sapiens cDNA...  1997       0.0    99%
AL518238.2 AL518238 Homo sapiens NEUROBLASTOMA Homo sapiens cD...  1993       0.0    98%
```

ALIGNMENTS

```
Query       2     TTTTTCGCAACGGGTTTGCCGCCAGAACACAGGTGTCGTGAAAACTACCCCTAAAAGCCAAAATGGGAAAGGAAAAGACTCATATCACCATTGATATCTCCTTGTGGAAATTTGAGACCAGCAAGTACTATGTGACTATCATTGATGCCC  151
BY794942.2  277                                                                                     ....................................................................  344
BY794942.2  25                       ....................................................................A.....                                                         98
DC527884.1  280                                                                                     ...T................................................................  347
DC527884.1  9     .......................................................................................A.....                                                         101
CO001006.1  1464                                                                                                                   .....................T..C............  1428
CF111220.1  273                                                                                     ......T.....C......C.......G........................................  340

Query       152   CAGGACACAGAGACTTTATCAAAAACATGATTACAGGGACATCTCAGGCTGACTGTGCTGTCCTGATTGTTGCTGCTGGTGTTGGTGAATTTGAAGCTGGTATCTCCAAGAATGGGCAGACCCGAGAGCATGCCCTTCTGGCTTACACAC  301
BY794942.2  345   ......................................................................................................................................................  494
DC527884.1  348   ................C.....................................................................................................................................  497
CO001006.1  1427  ................C....................C.....C.....................................................C..............C...........T.......................C.  1278
CF111220.1  341   ................C....G............G..C.................C.............................G..........................C...........T........T..............TT  490

Query       302   TGGGTGTGAAACAACTAATTGTCGGTGTTAACAAAATGGATTCCACTGAGCCACCCTACAGCCAGAAGAGATATGAGGAAATTGTTAAGGAAGTCAGCACTTACATTAAGAAAATTGGCTACAACCCCGACACAGTAGCATTTGTGCCAA  451
BY794942.2  495   ......................................................................................................................................................  644
DC527884.1  498   ......................................................................................................................................................  647
BX426149.2  1196                                   ......-..A....-.A..............G.....................................................................................  1082
CO001006.1  1277  .....................T..C.................T..............T..............C..........................C..................................................  1128
BX418564.2  1196                              ....-....-......-...-...-...YH.VS..-.R......-W.-..G..W....-.R......M.....K....-.R.-......S....-..........-R.....-.........  1089
CF111220.1  491   ............G........T.....C.................C........A.....T...........C........C.................C..........................T.......................  640
AL567209.3  1145                                                       ......S.....-...W.-..........-K.W........R.....-....A.M.C............C...........................  1052
                                                                                                                          \
                                                                                                                          |
                                                                                                                          M
BX440470.1  1128                                                                                                              ....-.....-..-W.-.............-...-....-S.M.  1091
                                                                                                                                                  \
                                                                                                                                                  |
                                                                                                                                                  C
BX420340.1  1117                                                                                                                          ...M..M..................K.-..M.  1086
AL518238.2  1148                                                                                      .....R..-....-.M.....-..........-..W...M....V.M...................  1087
```

```
Query       452   TTTCTGGTTGGAATGGTGACAACATGCTGGAGCCAAGTGCTAACATGCCTTGGTTCAAGGGATGGAAAGTCACCCGTAAGGATGGCAATGCCAGTGGAACCACGCTGCTTGAGGCTCTGGACTGCATCCTACCACCAACTCGTCCAACTG  601
BY794942.2  645   ......................................................................................................................................................  794
DC527884.1  648   ......................................................................................................................................................  797
BX426149.2  1081  ......................................................................................................................................................  932
CO001006.1  1127  .......C...................................T.....A................................C....................C........A........T........G...........C.......  978
BX418564.2  1088  ........-.....-..........................C......................-.....................................................................................  942
CF111220.1  641   ...........................................T.....G..........................C..A.......G.........C...........G..A...T..........T..G..G................  790
AL567209.3  1051  .......................................................................................................................K..............................  902
BX440470.1  1090  ..Y.-.K.......-..............-.....................K...................................................................R..............................  944
BX420340.1  1085  W...YT..D...K.-.............T..........Y...............................................................................A.............................R  937
AL518238.2  1086  ......-K.........A...........A....................................W.............................R.....................................................  937
                                                                                                 \
                                                                                                 |
                                                                                                 A

Query       602   ACAAGCCCTTGCGCCTGCCTCTCCAGGATGTCTACAAAATTGGTGGTATTGGTACTGTTCCTGTTGGCCGAGTGGAGACTGGTGTTCTCAAACCCGGTATGGTGGTCACCTTTGCTCCAGTCAACGTTACAACGGAAGTAAAATCTGTCG  751
BY794942.2  795   ......................................................................................................................................................  944
DC527884.1  798   ......................................................................................................................................................  947
BX426149.2  931   ......................................................................................................................................................  782
CO001006.1  977   ....A........TT...................T.......................A.....G..T..T.......................T..C.................C........T..A.....T..G..G..G..C..A.  828
BX418564.2  941   ........C.............................................................................................................................................  792
CF111220.1  791   .......TC....A.....C..............T........C..C.....C.....G.....G...........A.................T...........T.................T..A.....T.....C..G.....G.  940
AL567209.3  901   ......................................................................................................................................................  752
BX440470.1  943   ......................................................................................................................................................  794
BX420340.1  936   ......................................................................................................................................................  787
AL518238.2  936   ............................................................................W...................................B.............................W.......  787

Query       752   AAATGCACCATGAAGCTTTGAGTGAAGCTCTTCCTGGGGACAATGTGGGCTTCAATGTCAAGAATGTGTCTGTCAAGGATGTTCGTCGTGGCAACGTTGCTGGTGACAGCAAAAATGACCCACCAATGGAAGCAGCTGGCTTCACTGCTC  901
BY794942.2  945   ......................................................................................................................................................  1094
DC527884.1  948   ......................................................................................................................................................  1097
BX426149.2  781   ......................................................................................................................................................  632
CO001006.1  827   ............................C...................................C...........A.....C...........T..G....................T.....C........T......C....A....  678
BX418564.2  791   ........................................................................C.............................................................................  642
CF111220.1  941   ...............................G..............A........C..A.....C...........A..C..A..A........T........G..............................................  1090
AL567209.3  751   ..................................................................................................................-...................................  603
BX440470.1  793   ......................................................................................................................................................  644
BX420340.1  786   ......................................................................................................................................................  637
AL518238.2  786   ........................................................................................................A............................G................  637

Query       902   AGGTGATTATCCTGAACCATCCAGGCCAAATAAGCGCCGGCTATGCCCCTGTATTGGATTGCCACACGGCTCACATTGCATGCAAGTTTGCTGAGCTGAAGGAAAAGATTGATCGCCGTTCTGGTAAAAAGCTGGAAGATGGCCCTAAAT  1051
BY794942.2  1095  ......................................................................................................................................................  1245
                                      \
                                      |
                                      G
DC527884.1  1098  ......................................................................................................................................................  1247
BX426149.2  631   ......................................................................................................................................................  482
CO001006.1  677   ..........T....................C..T.GT..A.....A.....GC.......T.....A...........T.......................G...........T........G.........................  528
BX418564.2  641   .........................................................................................................M............................................  492
CF111220.1  1091  ............................G..C..T..T..............TC....C...........C.....A..............C.....T..A..G.....C.....T...........G.................C....  1240
AL567209.3  602   ......................................................................................................................................................  453
BX440470.1  643   ......................................................................................................................................................  493
                                                                                                                             \
                                                                                                                             |
                                                                                                                             C
BX420340.1  636   ......................................................................................................................................................  487
AL518238.2  636   ............................................................................Y.........................................................................  487

Query       1052  TCTTGAAGTCTGGTGATGCTGCCATTGTTGATATGGTTCCTGGCAAGCCCATGTGTGTTGAGAGCTTCTCAGACTATCCACCTTTGGGTCGCTTTGCTGTTCGTGATATGAGACAGACAGTTGCGGTGGGTGTCATCAAAGCAGTGGACA  1201
BY794942.2  1246  ......................................................................................................................................................  1395
DC527884.1  1248  ...................................................................................................................................A..................  1397
BX426149.2  481   ......................................................................................................................................................  332
CO001006.1  527   .......A........C........C.......................T........C........T..T..T.....T..C.....C..T........G.....C..............C..T.........................  378
BX418564.2  491   ......................................................................................................................................................  342
CF111220.1  1241  ...............................C.....C.......................A........T.....C..T..AC.T.....T..............C.....G...........T.................C.......  1390
AL567209.3  452   .....................................K................................................................................................................  303
BX440470.1  492   ......................................................................................................................................................  343
BX420340.1  486   ......................................................................................................................................................  337
AL518238.2  486   ......................................................................................................................................................  337

Query       1202  AGAAGGCTGCTGGAGCTGGCAAGGTCACCAAGTCTGCCCAGAAAGCTCAGAAGGCTAAATGAATATTATCCCTAATACCTGCCACCCCACTCTTAATCAGTGGTGGAAGAACGGTCTCAGAACTGTTTGTTTCAATTGGCCATTTAAGTT  1351
BY794942.2  1396  ......................................................................................................................................................  1545
DC527884.1  1398  .............................................................................................C........................................................  1547
BX426149.2  331   ......................................................................................................................................................  182
CO001006.1  377   .......A................................................................C................G........................................C...................  228
BX418564.2  341   ......................................................................................................................................................  192
CF111220.1  1391  ..........A...........A....................................................C.............G........................................C...................  1540
AL567209.3  302   ................................................M......Y..............................................................................................  152
                                                                                                                        \
                                                                                                                        |
                                                                                                                        A
BX440470.1  342   ......................................................................................................................................................  193
BX420340.1  336   ......................................................................................................................................................  187
```

```
AL518238.2  336   .............G..........................................................................................................G.............................  187

Query       1352  TAGTAGTAAAAGACTGGTTAATGATAACAATGCATCGTAAAACCTTCAGAAGGAAAGGAGAATgttttgtggaccactttggttttcttttttgcgtgtggcagttttaagttattagtttttaaaatcagtacttttttaATGGAAACAA  1501
BY794942.2  1546  ......................................................................................................................................................  1695
DC527884.1  1548  ......................................................................................................................................................  1697
BX426149.2  181   ...............................................................................................................................................-......  33
CO001006.1  227   ..A.........................................................................-.A..-....G.-----.--........-......................................A......  87
                                                                                      \
                                                                                      |
                                                                                      T
BX418564.2  191   ......................................................................................................................................................  42
CF111220.1  1541  ..A....................................................---..................-...--....-..G.----...........................C...........................  1679
AL567209.3  151   ..........................................................................................................................................-....-..-...  4
                                                                                            \
                                                                                            |
                                                                                            G
BX440470.1  192   ......................................................................................................................................................  43
BX420340.1  186   ......................................................................................................................................................  37
AL518238.2  186   ....................................................................................................................................................N.  37

Query       1502  CTTGACCAAAAATTTGTCACAGAATTTTGAGACCCATTAAAAAAGTTAAATG  1553
BY794942.2  1696  ....................................................  1747
DC527884.1  1698  ....................................................  1749
BX426149.2  32    .-.....G......G.-..G...........T..                    1
CO001006.1  86    .............C.............-...................T....  35
                                                              \
                                                              |
                                                              A
BX418564.2  41    ...........................M-.............            1
CF111220.1  1680  .............C....................-............T....  1731
                                                            \
                                                            |
                                                            C
AL567209.3  3     -...                                                  1
BX440470.1  42    ..........................................            1
BX420340.1  36    ..............................R.....                  1
AL518238.2  36    ............N.....................                    1
                                    \
                                    |
                                    N

Query= T8-26 [organism=Homo sapiens] eef1a1

Length=1631


                                                                   Score        E     Max
Sequences producing significant alignments:                       (Bits)     Value   ident

DC527884.1 DC527884 chimpanzee skin cDNA library PstA Pan trog...  1724       0.0    100%
AL541952.3 AL541952 Homo sapiens PLACENTA Homo sapiens cDNA cl...  1716       0.0    100%
AL559705.3 AL559705 Homo sapiens B CELLS (RAMOS CELL LINE) Hom...  1711       0.0    100%
AL557904.3 AL557904 Homo sapiens T CELLS (JURKAT CELL LINE) CO...  1711       0.0    100%
AL536241.3 AL536241 Homo sapiens FETAL BRAIN Homo sapiens cDNA...  1711       0.0    100%
BX459223.2 BX459223 Homo sapiens PLACENTA Homo sapiens cDNA cl...  1711       0.0    100%
BX459080.2 BX459080 Homo sapiens PLACENTA Homo sapiens cDNA cl...  1711       0.0    100%
BX417904.2 BX417904 Homo sapiens PLACENTA Homo sapiens cDNA cl...  1711       0.0    100%
BX417296.2 BX417296 Homo sapiens PLACENTA Homo sapiens cDNA cl...  1711       0.0    100%
BX440304.2 BX440304 Homo sapiens FETAL BRAIN Homo sapiens cDNA...  1707       0.0    100%

ALIGNMENTS

Query       2     TTTTTCGCAACGGGTTTGCCGCCAGAACACAGGTGTCGTGAAAACTACCCCTAAAAAGCCAAAATGGGAAAGGAAAAGACTCATATCAACATTGTCGTCATTGGACACGTAGATTCGGGCAAGTCCACCACTACTGGCCATCTGATCTAT  151
DC527884.1  9     ....................................................-.................................................................................................  157
AL541952.3  1               ....A.....................................-.................................................................................................  139
AL559705.3  1               ....A.....................................-.................................................................................................  139
AL557904.3  1               ....A.....................................-.................................................................................................  139
AL536241.3  1               ....A.....................................-.................................................................................................  139
BX459223.2  1               ....A.....................................-.................................................................................................  139
BX459080.2  1               ....A.....................................-.................................................................................................  139
BX417904.2  1               ....A.....................................-.................................................................................................  139
BX417296.2  1               ....A.....................................-.................................................................................................  139
BX440304.2  1               ....A.....................................-.................................................................................................  139

Query       152   AAATGCGGTGGCATCGACAAAAGAACCATTGAAAAATTTGAGAAGGAGGCTGCTGAGATGGGAAAGGGCTCCTTCAAGTATGCCTGGGTCTTGGATAAACTGAAAGCTGAGCGTGAACGTGGTATCACCATTGATATCTCCTTGTGGAAA  301
DC527884.1  158   .............................................................................................................................T........................  307
AL541952.3  140   ......................................................................................................................................................  289
AL559705.3  140   ......................................................................................................................................................  289
AL557904.3  140   ......................................................................................................................................................  289
AL536241.3  140   ......................................................................................................................................................  289
BX459223.2  140   ......................................................................................................................................................  289
BX459080.2  140   ......................................................................................................................................................  289
```

```
BX417904.2  140   ......................................................................................................................................................  289
BX417296.2  140   ......................................................................................................................................................  289
BX440304.2  140   ......................................................................................................................................................  289

Query       302   TTTGAGACCAGCAAGTACTATGTGACTATCATTGATGCCCCAGGACACAGAGACTTTATCAAAAACATGATTACAGGGACATCTCAGGCTGACTGTGCTGTCCTGATTGTTGCTGCTGGTGTTGGTGAATTTGAAGCTGGTATCTCCAAG  451
DC527884.1  308   ........................................................C.............................................................................................  457
AL541952.3  290   ......................................................................................................................................................  439
AL559705.3  290   ......................................................................................................................................................  439
AL557904.3  290   ......................................................................................................................................................  439
AL536241.3  290   ......................................................................................................................................................  439
BX459223.2  290   ......................................................................................................................................................  439
BX459080.2  290   ......................................................................................................................................................  439
BX417904.2  290   ......................................................................................................................................................  439
BX417296.2  290   ......................................................................................................................................................  439
BX440304.2  290   ......................................................................................................................................................  439

Query       452   AATGGGCAGACCCGAGAGCATGCCCTTCTGGCTTACACACTGGGTGTGAAACAACTAATTGTCGGTGTTAACAAAATGGATTCCACTGAGCCACCCTACAGCCAGAAGAGATATGAGGAAATTGTTAAGGAAGTCAGCACTTACATTAAG  601
DC527884.1  458   ......................................................................................................................................................  607
AL541952.3  440   ......................................................................................................................................................  589
AL559705.3  440   ......................................................................................................................................................  589
AL557904.3  440   ......................................................................................................................................................  589
AL536241.3  440   ......................................................................................................................................................  589
BX459223.2  440   ......................................................................................................................................................  589
BX459080.2  440   ......................................................................................................................................................  589
BX417904.2  440   ......................................................................................................................................................  589
BX417296.2  440   ......................................................................................................................................................  589
BX440304.2  440   ......................................................................................................................................................  589

Query       602   AAAATTGGCTACAACCCCGACACAGTAGCATTTGTGCCAATTTCTGGTTGGAATGGTGACAACATGCTGGAGCCAAGTGCTAACATGCCTTGGTTCAAGGGATGGAAAGTCACCCGTAAGGATGGCAATGCCAGTGGAACCACGCTGCTT  751
DC527884.1  608   ......................................................................................................................................................  757
AL541952.3  590   ......................................................................................................................................................  739
AL559705.3  590   ......................................................................................................................................................  739
AL557904.3  590   ......................................................................................................................................................  739
AL536241.3  590   ......................................................................................................................................................  739
BX459223.2  590   ......................................................................................................................................................  739
BX459080.2  590   ......................................................................................................................................................  739
BX417904.2  590   ......................................................................................................................................................  739
BX417296.2  590   ......................................................................................................................................................  739
BX440304.2  590   ......................................................................................................................................................  740
                                                                                                                                                \
                                                                                                                                                |
                                                                                                                                                T

Query       752   GAGGCTCTGGACTGCATCCTACCACCAACTCGTCCAACTGACAAGCCCTTGCGCCTGCCTCTCCAGGATGTCTACAAAATTGGTGGTATTGGTACTGTTCCTGTTGGCCGAGTGGAGACTGGTGTTCTCAAACCCGGTATGGTGGTCACC  901
DC527884.1  758   ......................................................................................................................................................  907
AL541952.3  740   ......................................................................................................................................................  889
AL559705.3  740   ......................................................................................................................................................  889
AL557904.3  740   ......................................................................................................................................................  889
AL536241.3  740   ......................................................................................................................................................  889
BX459223.2  740   ......................................................................................................................................................  889
BX459080.2  740   ......................................................................................................................................................  889
BX417904.2  740   ......................................................................................................................................................  889
BX417296.2  740   ......................................................................................................................................................  889
BX440304.2  741   ......................................................................................................................................................  890

Query       902   TTTGCTCCAGTCAACGTTACAACGGAAGTAAAATCTGTCGAAAATGACCCACCAATGGAAGCAGCTGGCTTCACTGCTCAGGTGATTATCCTGAACCATCCAGGCCAAATAAGCGCCGGCTATGCCCCTGTATTGGATTGCCACACGGCT  1051
DC527884.1  908   ...........................................                                                                                                             950
DC527884.1  1059                                          ..............................................................................................................  1168
AL541952.3  890   ..............................................                                                                                                          935
AL559705.3  890   ...........................................                                                                                                             932
AL557904.3  890   ...........................................                                                                                                             932
AL536241.3  890   ...........................................                                                                                                             932
BX459223.2  890   ...........................................                                                                                                             932
BX459080.2  890   ...........................................                                                                                                             932
BX417904.2  890   ...........................................                                                                                                             932
BX417296.2  890   ...........................................                                                                                                             932
BX440304.2  891   ...........................................                                                                                                             933

Query       1052  CACATTGCATGCAAGTTTGCTGAGCTGAAGGAAAAGATTGATCGCCGTTCTGGTAAAAAGCTGGAAGATGGCCCTAAATTCTTGAAGTCTGGTGATGCTGCCATTGTTGATATGGTTCCTGGCAAGCCCATGTGTGTTGAGAGCTTCTCA  1201
DC527884.1  1169  ......................................................................................................................................................  1318

Query       1202  GACTATCCACCTTTGGGTCGCTTTGCTGTTCGTGATATGAGACAGACAGTTGCGGTGGGTGTCATCAAAGCAGTGGACAAGAAGGCTGCTGGAGCTGGCAAGGTCACCAAGTCTGCCCAGAAAGCTCAGAAGGCTAAATGAATATTATCC  1351
DC527884.1  1319  ............................................................A.........................................................................................  1468

Query       1352  CTAATACCTGCCACCCCACTCTTAATCAGTGGTGGAAGAACGGTCTCAGAACTGTTTGTTTCAATTGGCCATTTAAGTTTAGTAGTAAAAGACTGGTTAATGATAACAATGCATCGTAAAACCTTCAGAAGGAAAGGAGAATgttttgtg  1501
DC527884.1  1469  ......................C...............................................................................................................................  1618

Query       1502  gaccactttggttttctttttttgcgtgtggcagttttaagttattagtttttaaaatcagtactttttaATGGAAACAACTTGACCAAAAATTTGTCACAGAATTTTGAGACCCATTAAAAAAGTTAAAT  1631
DC527884.1  1619  ..................................................................................................................................  1748

Query= T9-28 [organism=Homo sapiens] eef1a1
```

```
Length=1433


                                                                  Score        E     Max
Sequences producing significant alignments:                      (Bits)     Value  ident

DC527884.1 DC527884 chimpanzee skin cDNA library PstA Pan trog...  1969       0.0    100%
BY794942.2 BY794942 Homo sapiens eye Homo sapiens cDNA clone H...  1945       0.0    100%
DC629953.1 DC629953 macaque bone marrow cDNA library QbmA Maca...  1941       0.0    96%
BX426072.2 BX426072 Homo sapiens NEUROBLASTOMA Homo sapiens cD...  1910       0.0    100%
FS580102.1 FS580102 macaque heart cDNA library QhtB Macaca fas...  1906       0.0    99%
BX440468.2 BX440468 Homo sapiens FETAL BRAIN Homo sapiens cDNA...  1905       0.0    99%
BX440573.2 BX440573 Homo sapiens FETAL BRAIN Homo sapiens cDNA...  1903       0.0    100%
BX426150.2 BX426150 Homo sapiens NEUROBLASTOMA Homo sapiens cD...  1899       0.0    99%
BX402793.2 BX402793 Homo sapiens T CELLS (JURKAT CELL LINE) CO...  1899       0.0    99%
AL528506.3 AL528506 Homo sapiens NEUROBLASTOMA COT 25-NORMALIZ...  1897       0.0    99%

ALIGNMENTS

Query       1     CTTTTTCGCAACGGGTTTGCCGCCAGAACACAGGTGTCGTGAAAACTACCCCTAAAAAGCCAAAATGGGAAAGGAAAAGACTCATATCAACATTGTCGTCATTGGACACGTAGATTCGGGCAAGTCCACCACTACTGGCCATCTGATCTA  150
DC527884.1  9      ....................................................-................................................................................................  156
BY794942.2  25                        .................................-................................................................................................  153
DC629953.1  1     .....................................................-................................................................................................  149
BX426072.2  1                                   .......................-................................................................................................  119
FS580102.1  1     .....................................................-................................................................................................  149
BX440468.2  1                          ................................-................................................................................................  128
BX440573.2  1                                   .......................-................................................................................................  119
BX426150.2  1                              ..T.........................-................................................................................................  124
BX402793.2  6                      ....................................-................................................................................................  137
AL528506.3  1                                   .......................-................................................................................................  119

Query       151   TAAATGCGGTGGCATCGACAAAAGAACCATTGAAAAATTTGAGAAGGAGGCTGCTGAGATGGGAAAGGGCTCCTTCAAGTATGCCTGGGTCTTGGATAAACTGAAAGCTGAGCGTGAACGTGGTATCACCATTGATATCTCCTTGTGGAA  300
DC527884.1  157   ..............................................................................................................................T.......................  306
BY794942.2  154   ......................................................................................................................................................  303
DC629953.1  150   C.................................................................................................................G...................................  299
BX426072.2  120   ......................................................................................................................................................  269
FS580102.1  150   C.................................................................................................................G...................................  299
BX440468.2  129   ......................................................................................................................................................  278
BX440573.2  120   ......................................................................................................................................................  269
BX426150.2  125   ......................................................................................................................................................  274
BX402793.2  138   ......................................................................................................................................................  287
AL528506.3  120   ......................................................................................................................................................  269

Query       301   ATTTGAGACCAGCAAGTACTATGTGACTATCATTGATGCCCCAGGACACAGAGACTTTATCAAAAACATGATTACAGGGACATCTCAGGCTGACTGTGCTGTCCTGATTGTTGCTGCTGGTGTTGGTGAATTTGAAGCTGGTATCTCCAA  450
DC527884.1  307   .........................................................C............................................................................................  456
BY794942.2  304   ......................................................................................................................................................  453
DC629953.1  300   ..................T......................................C............................................................................................  449
BX426072.2  270   ......................................................................................................................................................  419
FS580102.1  300   ..................T......................................C............................................................................................  449
BX440468.2  279   ......................................................................................................................................................  428
BX440573.2  270   ......................................................................................................................................................  419
BX426150.2  275   ......................................................................................................................................................  424
BX402793.2  288   ......................................................................................................................................................  437
AL528506.3  270   ......................................................................................................................................................  419

Query       451   GAATGGGCAGACCCGAGAGCATGCCCTTCTGGCTTACACACTGGGTGTGAAACAACTAATTGTCGGTGTTAACAAAATGGATTCCACTGAGCCACCCTACAGCCAGAAGAGATATGAGGAAATTGTTAAGGAAGTCAGCACTTACATTAA  600
DC527884.1  457   ......................................................................................................................................................  606
BY794942.2  454   ......................................................................................................................................................  603
DC629953.1  450   ........................T......................................T..................................................C...............................C...  599
BX426072.2  420   ......................................................................................................................................................  569
FS580102.1  450   ........................T......................................T..................................................C...................................  599
BX440468.2  429   ......................................................................................................................................................  578
BX440573.2  420   ......................................................................................................................................................  569
BX426150.2  425   ......................................................................................................................................................  574
BX402793.2  438   ......................................................................................................................................................  587
AL528506.3  420   ......................................................................................................................................................  569

Query       601   GAAAATTGGCTACAACCCCGACACAGTAGCATTTGTGCCAATTTCTGGTTGGAATGGTGACAACATGCTGGAGCCAAGTGCTAACATGCCTTGGTTCAAGGGATGGAAAGTCACCCGTAAGGATGGCAATGCCAGTGGAACCACGCTGCT  750
DC527884.1  607   ......................................................................................................................................................  756
BY794942.2  604   ......................................................................................................................................................  753
DC629953.1  600   ............................................................................................................G.........................................  749
BX426072.2  570   ......................................................................................................................................................  719
FS580102.1  600   ............................................................................................................G.........................................  749
BX440468.2  579   ........................................................................................................................-.............................  727
BX440573.2  570   ......................................................................................................................................................  719
BX426150.2  575   ......................................................................................................................................................  724
BX402793.2  588   ......................................................................................................................................................  737
AL528506.3  570   ......................................................................................................................................................  719

Query       751   TGAGGCTCTGGACTGCATCCTACCACCAACTCGTCCAACTGACAAGCCCTTGCGCCTGCCTCTCCAGGATGTCTACAAAATTGGTGGTATTGGTACTGTTCCTGTTGGCCGAGTGGAGACTGGTGTTCTCAAACCCGGTATGGTGGTCAC  900
DC527884.1  757   ......................................................................................................................................................  906
BY794942.2  754   ......................................................................................................................................................  903
```

```
DC629953.1  750   ......................................................................................................................................................  899
BX426072.2  720   ......................................................................................................................................................  869
FS580102.1  750   ......................................................................................................................................................  899
BX440468.2  728   ......................................................................................................................................................  877
BX440573.2  720   ......................................................................................................................................................  869
BX426150.2  725   ......................................................................................................................................................  874
BX402793.2  738   ......................................................................................................................................................  887
AL528506.3  720   ......................................................................................................................................................  869

Query       901   CTTTGCTCCAGTCAACGTTACAACGGAAGTAAAATCTGTCGAAATGCACCATGAAGCTTTGAGTGAAGCTCTTCCTGGGGACAATGTGGGCTTCAATGTCAAGAATGTGTCTGTCAAGGATGTTCGTCGTGGCAACGTTGCTGGTGACAG  1050
DC527884.1  907   ......................................................................................................................................................  1056
BY794942.2  904   ......................................................................................................................................................  1053
DC629953.1  900   ........................T..........................................................-..........-............................-.........-................  1045
BX426072.2  870   ......................................................................................................................................-...............  1020
                                                                                                                                                                \  \
                                                                                                                                                                |  |
                                                                                                                                                                T  G
FS580102.1  900   ........................T.........................................................................................................-...................  1048
BX440468.2  878   ................................................................................................W....................R................M-.............S  1026
BX440573.2  870   .....................................................................................................................................-...............-  1017
BX426150.2  875   .....................................................................................................................................-...............-  1023
                                                                                              \
                                                                                              |
                                                                                              G
BX402793.2  888   ..............................S..................................-.................................TM.A..............................................S  1036
AL528506.3  870   .....................................................................................................................................-...............S  1018

Query       1051  CAAAAATGACCCACCAATGGAAGCAGTGGACAAGAAGGCTGCTGGAGCTGGCAAGGTCACCAAGTCTGCCCAGAAAGCTCAGAAGGCTAAATGAATATTATCCCTAATACCTGCCACCCCACTCTTAATCAGTGGTGGAAGAACGGTCTC  1200
DC527884.1  1057  .............................                                                                                                           1086
                                            \
                                            |
                                            C
DC527884.1  1386                      .........................................................................................................C........................  1515
BY794942.2  1054  .............................                                                                                                           1083
                                            \
                                            |
                                            C
BY794942.2  1384                      ..................................................................................................................................  1513
DC629953.1  1046  ....C--.......-.-..-.-......-...-..-.-......-........-.-.....-.............-.......-.-..T.-.....-......-..-...-................-...-.......-....C...    1169
                                                                                                                                        \
                                                                                                                                        |
                                                                                                                                        C
BX426072.2  1021  .............................                                                                                                           1050
                                            \
                                            |
                                            C
FS580102.1  1049  .....C-.........-.........                                                                                                              1072
BX440468.2  1027  -...........M................                                                                                                           1055
                                            \
                                            |
                                            C
BX440573.2  1018  ..............-.........                                                                                                                1040
BX426150.2  1024  ...........-.........-.......                                                                                                           1051
                                            \
                                            |
                                            C
BX402793.2  1037  .....WKR...M-................                                                                                                           1065
                                            \
                                            |
                                            C
AL528506.3  1019  M....-........-..........                                                                                                               1041

Query       1201  AGAACTGTTTGTTTCAATTGGCCATTTAAGTTTAGTAGTAAAAGACTGGTTAATGATAACAATGCATCGTAAAACCTTCAGAAGGAAAGGAGAATgttttgtggaccactttggttttctttttttgcgtgtggcagttttaagttattag  1350
DC527884.1  1516  ......................................................................................................................................................  1665
BY794942.2  1514  ......................................................................................................................................................  1663

Query       1351  tttttaaaatcagtactttttaATGGAAACAACTTGACCAAAAATTTGTCACAGAATTTTGAGACCCATTAAAAAAGTTAAAT  1433
DC527884.1  1666  ...................................................................................  1748
BY794942.2  1664  ...................................................................................  1746

Query= T10-2 [organism=Homo sapiens] eef1a1

Length=1660


                                                                 Score        E     Max
Sequences producing significant alignments:                     (Bits)     Value  ident

BY794942.2 BY794942 Homo sapiens eye Homo sapiens cDNA clone H...  1901       0.0    100%
DC527884.1 DC527884 chimpanzee skin cDNA library PstA Pan trog...  1893       0.0    100%
BX440470.1 BX440470 Homo sapiens FETAL BRAIN Homo sapiens cDNA...  1881       0.0    100%
BX418589.2 BX418589 Homo sapiens FETAL BRAIN Homo sapiens cDNA...  1877       0.0    100%
```

```
BX442182.2 BX442182 Homo sapiens FETAL BRAIN Homo sapiens cDNA...  1871       0.0    100%
BX459625.2 BX459625 Homo sapiens FETAL BRAIN Homo sapiens cDNA...  1866       0.0    100%
BX420340.1 BX420340 Homo sapiens FETAL BRAIN Homo sapiens cDNA...  1864       0.0    100%
BX418564.2 BX418564 Homo sapiens FETAL BRAIN Homo sapiens cDNA...  1862       0.0    99%
BX440436.2 BX440436 Homo sapiens FETAL BRAIN Homo sapiens cDNA...  1857       0.0    99%
AL541984.3 AL541984 Homo sapiens PLACENTA Homo sapiens cDNA cl...  1855       0.0    100%

ALIGNMENTS

Query       2     TTTTTCGCAACGGGTTTGCCGCCAGAACACAGGTGTCGTGAAAACTACCCCTAAAAGCCAAAATGGGAAAGGAAAAGACTCATATCAACATTGTCGTCATTGGACACGTAGATTCGGGCAAGTCCACCACTACTGGCCATCTGATCTATA  151
BY794942.2  25                       ...................................................................................................................................  155
DC527884.1  9     ......................................................................................................................................................  158

Query       152   AATGCGGTGGCATCGACAAAAGAACCATTGAAAAATTTGAGAAGGAGGCTGCTGAGATGGGAAAGGGCTCCTTCAAGTATGCCTGGTCTTGGATAAACTGAAAGCTGAGCGTGAACGTGGTATCACCATTGATATCTCCTTGTGGAAATT  301
BY794942.2  156   ......................................................................................................................................................  306
                                                                                                      \
                                                                                                      |
                                                                                                      G
DC527884.1  159   ...........................................................................................................................T..........................  309
                                                                                                      \
                                                                                                      |
                                                                                                      G

Query       302   TGAGACAGCAAGTACTATGTGACTATCATTGATGCCCAGGACACAGAGACTTTATCAAAAACATGATTACAGGGACATCTCAGGCTGACTGTGCTGTCCTGATTGTTGCTGCTGGTGTTGGTGAATTTGAAGCTGGTATCTCCAAGAATG  451
BY794942.2  307   ......................................................................................................................................................  458
                       \                            \
                       |                            |
                       C                            C
DC527884.1  310   ....................................................C.................................................................................................  461
                       \                            \
                       |                            |
                       C                            C

Query       452   GGCAGACCCGAGAGCATGCCCTTCTGGCTTACACACTGGGTGTGAAACAACTAATTGTCGGTGTTAACAAAATGGATTCCACTGAGCCACCCTACAGCCAGAAGAGATATGAGGAAATTGTTAAGGAAGTCaaaaaaaaaaaaaaaaaa  601
BY794942.2  459   ....................................................................................................................................                    590
DC527884.1  462   ....................................................................................................................................                    593

Query       602   aaaaaaaaaaaaaaaTTGTTAAAGGAAGTCACCCGTAAGGATGGCAATGCCAGTGGAACCACGCTGCTTGAGGCTCTGGACTGCATCCTACCACCAACTCGTCCAACTGACAAGCCCTTGCGCCTGCCTCTCCAGGATGTCTACAAAATT  751
BY794942.2  711                            .............................................................................................................................  835
DC527884.1  714                            .............................................................................................................................  838
BX440470.1  1027                           .....................................................R.......................................................................  903
BX418589.2  1026                           .............................................................................................................................  902
BX442182.2  1019                           .............................................................................................................................  895
BX459625.2  1014                           .............................................................................................................................  890
BX420340.1  1020                           .....................................................A.............................R.........................................  896
BX418564.2  1025                           ............................................................................................C................................  901
BX440436.2  1026                           ..................-...................-YK.....-........................................R.....................................  905
AL541984.3  1                                                                  ........................................................................................................  104

Query       752   GGTGGTATTGGTACTGTTCCTGTTGGCCGAGTGGAGACTGGTGTTCTCAAACCCGGTATGGTGGTCACCTTTGCTCCAGTCAACGTTACAACGGAAGTAAAATCTGTCGAAATGCACCATGAAGCTTTGAGTGAAGCTCTTCCTGGGGAC  901
BY794942.2  836   ......................................................................................................................................................  985
DC527884.1  839   ......................................................................................................................................................  988
BX440470.1  902   ......................................................................................................................................................  753
BX418589.2  901   ......................................................................................................................................................  752
BX442182.2  894   ...................................R..................................................................................................................  745
BX459625.2  889   ......................................................................................................................................................  740
BX420340.1  895   ......................................................................................................................................................  746
BX418564.2  900   ......................................................................................................................................................  751
BX440436.2  904   ......................................................................................................................................................  755
AL541984.3  105   ......................................................................................................................................................  254

Query       902   AATGTGGGCTTCAATGTCAAGAATGTGTCTGTCAAGGATGTTCGTCGTGGCAACGTTGCTGGTGACAGCAAAAATGACCCACCAATGGAAGCAGCTGGCTTCACTGCTCAGGTGATTATCCTGAACCATCCAGGCCAAATAAGCGCCGGC  1051
BY794942.2  986   ......................................................................................................................................................  1136
                                                                                                                                                   \
                                                                                                                                                   |
                                                                                                                                                   G
DC527884.1  989   ......................................................................................................................................................  1138
BX440470.1  752   ......................................................................................................................................................  603
BX418589.2  751   ......................................................................................................................................................  602
BX442182.2  744   ......................................................................................................................................................  595
BX459625.2  739   ......................................................................................................................................................  590
BX420340.1  745   ......................................................................................................................................................  596
BX418564.2  750   ...............................C......................................................................................................................  601
BX440436.2  754   ......................................................................................................................................................  605
AL541984.3  255   ......................................................................................................................................................  404

Query       1052  TATGCCCCTGTATTGGATTGCCACACGGCTCACATTGCATGCAAGTTTGCTGAGCTGAAGGAAAAGATTGATCGCCGTTCTGGTAAAAAGCTGGAAGATGGCCCTAAATTCTTGAAGTCTGGTGATGCTGCCATTGTTGATATGGTTCCT  1201
BY794942.2  1137  ......................................................................................................................................................  1286
DC527884.1  1139  ......................................................................................................................................................  1288
BX440470.1  602   ......................................................................................................................................................  452
                                                                                    \
                                                                                    |
```

```
                                                                                    C
BX418589.2  601   .......................................................................Y.S............................................................................  452
BX442182.2  594   ......................................................................................................................................................  445
BX459625.2  589   ......................................................................................................................................................  440
BX420340.1  595   ......................................................................................................................................................  446
BX418564.2  600   ................................................................M.....................................................................................  451
BX440436.2  604   ......................................................................................................................................................  455
AL541984.3  405   ......................................................................................................................................................  554

Query       1202  GGCAAGCCCATGTGTGTTGAGAGCTTCTCAGACTATCCACCTTTGGGTCGCTTTGCTGTTCGTGATATGAGACAGACAGTTGCGGTGGGTGTCATCAAAGCAGTGGACAAGAAGGCTGCTGGAGCTGGCAAGGTCACCAAGTCTGCCCAG  1351
BY794942.2  1287  ......................................................................................................................................................  1436
DC527884.1  1289  ..........................................................................................A...........................................................  1438
BX440470.1  451   ......................................................................................................................................................  302
BX418589.2  451   ......................................................................................................................................................  302
BX442182.2  444   ......................................................................................................................................................  295
BX459625.2  439   ......................................................................................................................................................  290
BX420340.1  445   ......................................................................................................................................................  296
BX418564.2  450   ......................................................................................................................................................  301
BX440436.2  454   ......................................................................................................................................................  305
AL541984.3  555   ......................................................................................................................................................  704

Query       1352  AAAGCTCAGAAGGCTAAATGAATATTATCCCTAATACCTGCCACCCCACTCTTAATCAGTGGTGGAAGAACGGTCTCAGAACTGTTTGTTTCAATTGGCCATTTAAGTTTAGTAGTAAAAGACTGGTTAATGATAACAATGCATCGTAAA  1501
BY794942.2  1437  ......................................................................................................................................................  1586
DC527884.1  1439  ....................................................C.................................................................................................  1588
BX440470.1  301   ......................................................................................................................................................  152
BX418589.2  301   ......................................................................................................................................................  152
BX442182.2  294   ......................................................................................................................................................  145
BX459625.2  289   ......................................................................................................................................................  140
BX420340.1  295   ......................................................................................................................................................  146
BX418564.2  300   ......................................................................................................................................................  151
BX440436.2  304   ......................................................................................................................................................  155
AL541984.3  705   ......................................................................................................................................................  854

Query       1502  ACCTTCAGAAGGAAAGGAGAATgttttgtggaccactttggttttctttttttgcgtgtggcagttttaagttattagtttttaaaatcagtactttttaATGGAAACAACTTGACCAAAAATTTGTACAGAATTTTGAGACCCATTAAAA  1651
BY794942.2  1587  ......................................................................................................................................................  1737
                                                                                                                                                               \
                                                                                                                                                               |
                                                                                                                                                               C
DC527884.1  1589  ......................................................................................................................................................  1739
                                                                                                                                                               \
                                                                                                                                                               |
                                                                                                                                                               C
BX440470.1  151   ......................................................................................................................................................  1
                                                                                                                                                               \
                                                                                                                                                               |
                                                                                                                                                               C
BX418589.2  151   .......................................................................................................................................D..............  1
                                                                                                                                                               \
                                                                                                                                                               |
                                                                                                                                                               C
BX442182.2  144   ..............................................................................................................................................          1
                                                                                                                                                               \
                                                                                                                                                               |
                                                                                                                                                               C
BX459625.2  139   .........................................................................................................................................               1
                                                                                                                                                               \
                                                                                                                                                               |
                                                                                                                                                               C
BX420340.1  145   .........................................................................................................................................R.....         1
                                                                                                                                                               \
                                                                                                                                                               |
                                                                                                                                                               C
BX418564.2  150   .......................................................................................................................................M-.............  1
                                                                                                                                                               \
                                                                                                                                                               |
                                                                                                                                                               C
BX440436.2  154   ......................................................................................................................................................  3
                                                                                                                                                               \ \
                                                                                                                                                               | |
                                                                                                                                                               N N
AL541984.3  855   ..........................................................K.S.........................................................................................  1006
                                                                                                                                                               \ \
                                                                                                                                                               | |
                                                                                                                                                               C M

Query       1652  AAGTTAAAT  1660
BY794942.2  1738  .........  1746
DC527884.1  1740  .........  1748
BX440436.2  2     ..         1
AL541984.3  1007  .........  1015
```

Query= T11-30 [organism=Homo sapiens] eef1a1

```
Length=1164


                                                                      Score        E     Max
Sequences producing significant alignments:                          (Bits)     Value  ident

AU120002.1 AU120002 HEMBA1 Homo sapiens cDNA clone HEMBA100713...  1356       0.0    99%
CJ434202.1 CJ434202 macaque brain cDNA library QccE Macaca fas...  1216       0.0    96%
CR976939.1 CR976939 RZPD no.9016 Homo sapiens cDNA clone RZPDp...  1149       0.0    99%
CB138394.1 K-EST0191255 L15CKK1 Homo sapiens cDNA clone L15CKK...  1064       0.0    97%
GR891610.1 CANFLECK_JINDO_BR_55_A03.ab1 Jindo brain cDNA libra...  1035       0.0    92%
DC519934.1 DC519934 chimpanzee brain cDNA library PccB Pan tro...  996        0.0    97%
AU296783.1 AU296783 female adult cerebellum, full-length enric...  996        0.0    97%
CD466199.1 LeukoN2_2_E07.g1_A024 Unstimulated peripheral blood...  955        0.0    91%
AU144980.1 AU144980 HEMBA1 Homo sapiens cDNA clone HEMBA100353...  935        0.0    95%
DA429743.1 DA429743 COLON2 Homo sapiens cDNA clone COLON200583...  926        0.0    97%

ALIGNMENTS

Query       3     TTTTTCGCAACGGGTTTGCCGCCAGAACACAGGTGTCGTGAAAACTACCCCTAAAAGCCAAAATGGGAAAGGAAAAGACTCATATCAACATTGTCGTCATTGGACACGTAGATTCGGGCAAGTCCACCACTACTGGCCATCTGATCTATA  152
AU120002.1  2     ......................................................................................................................................................  151
CJ434202.1  30    ........-................-..........................................................................................................................C.  177
CR976939.1  1                                                                                                                            .................................  33
CB138394.1  1                                                                                                                                                         ....  4
GR891610.1  1               ...................................C...G......A................G........C........C.................T..............T.......................C.  144
                                                                         \\
                                                                         ||
                                                                       CCC|
                                                                          |
                                                                          T
DC519934.1  2     ...................................N..................................................................................................................  151
AU296783.1  2     ...................................N..................................................................................................................  151
CD466199.1  2     .............................................C....AA...............G.......................C...........G.....C....................G........C........C.  155
                                                                 \    \
                                                                 |    |
                                                                 G  TCT
DA429743.1  2     ......................................................................................................................................................  151

Query       153   AATGCGGTGGCATCGACAAAAGAACCATTGAAAAATTTGAGAAGGAGGCTGCTGAGATGGGAAAGGGCTCCTTCAAGTATGCCTGGGTCTTGGATAAACTGAAAGCTGAGCGTGAACGTGGTATCACCATTGATATCTCCTTGTGGAAAT  302
AU120002.1  152   ........................................................G.............................................................................................  301
CJ434202.1  178   ......................................................T.........................................................G.....................................  327
CR976939.1  34    ......................................................................................................................................................  183
CB138394.1  5     ......................................................................................................................................................  154
GR891610.1  145   ....T.....G..............T..C...................................A............................................A..............................C.........  294
DC519934.1  152   ............................................................................................................................T.........................  301
AU296783.1  152   ............................................................................................................................T.........................  301
CD466199.1  156   .G........G.................C..G.....C.......................................................................A.....G........................C.........  305
DA429743.1  152   ......................................................................................................................................................  302
                       \
                       |
                       N

Query       303   TTGAGACCAGCAAGTACTATGTGACTATCATTGATGCCCCAGGACACAGAGACTTTATCAAAAACATGATTACAGGGACATCTCAGGCTGACTGTGCTGTCCTGATTGTTGCTGCTGGTGTTGGTGTTCTCAAACCCGGTATGGTGGTCA  452
AU120002.1  302   ...................................................................................................................------.............................  445
CJ434202.1  328   ................T......................................C...........................................................------.............................  471
CR976939.1  184   ...................................................................................................................------.............................  327
CB138394.1  155   ......................................................................................................................................................  319
                                                                                                                                         \
                                                                                                                                         |
                                                                                                                           TGGTGAATTTGAAGC
GR891610.1  295   .C..............T..C.....C..................................................C......................................C......C-.-.----..G..T.............  438
DC519934.1  302   .......................................................C..............................................................................................  464
                                                                                                                                         \
                                                                                                                                         |
                                                                                                                             TGGTGTTGGAGAC
AU296783.1  302   .......................................................C..............................................................................................  464
                                                                                                                                         \
                                                                                                                                         |
                                                                                                                             TGGTGTTGGAGAC
CD466199.1  306   .C..............T........C..............C..............C....................C..G.......................C.....C...A........T-..-----.....T..C........T.  449
DA429743.1  303   ......................................................................................................................................................  467
                                                                                                                                  \\ \\  \
                                                                                                                                  || ||  |
                                                                                                                                 TT| TT GGAGAC
                                                                                                                                   |  |
                                                                                                                                   C GCCG

Query       453   CCTTTGCTCCAGTCAACGTTACAACGGAAGTAAAATCTGTCGAAATGCACCATGAAGCTTTGAGTGAAGCTCTTCCTGGGGACAATGTGGGCTTCAATGTCAAGAATGTGTCTGTCAAGGATGTTCGTCGTGGCAACGTTGCTGGTGACA  602
AU120002.1  446   ......................................................................................................................................................  595
CJ434202.1  472   .........................T............................................................................................................................  621
CR976939.1  328   ......................................................................................................................................................  477
```

```
CB138394.1  320   ......................................................................................................................................................  469
GR891610.1  439   ................T........T........G.....T..........................G......................................C..A........A.................T..G..........  588
DC519934.1  465   ..................................................................................................................................                      594
AU296783.1  465   ..................................................................................................................................                      594
CD466199.1  450   ................T........T........G.....T........T..............C....................C....................C.....C.....A.........C.......T..G..........  599
AU144980.1  594                                                                                                                                      ................C..  576
DA429743.1  468   ...........................................................................................                                                             558

Query       603   GCAAAAATGACCCACCAATGGAAGCAGCTGGCTTCACTGCTCAGGTGATTATCCTGAACCATCCAGGCCAAATAAGCGCCGGCTATGCCCCTGTATTGGATTGCCACACGGCTCACATTGCATGCAAGTTTGCTGAGCTGAAGGAAAAGA  752
AU120002.1  596   ......................................................................................................................................................  745
CJ434202.1  622   .......C.......................G................A..T.............-..........T..T.............GG..............AAT......TG............................A.  772
                                                                         \      \
                                                                         |      |
                                                                         T      A
CR976939.1  478   ......................................................................................................................................................  627
CB138394.1  470   ......................................................................................................................................................  619
GR891610.1  589   .....................................A...................................C..T..T..A.....A.....GC....A..T.....A...........CC.............A...........    736
CD466199.1  600   ..........T..........................A...............................-...C..T..T..G.....A.....GC.......T.....A...........T...                         724
                                                                                  \
                                                                                  |
                                                                                  C
AU144980.1  575   .........N..N.......A..N....N..T.......G.....G...........C......N......T.......G..T............................T......................................  419
                          \       \                         \            \     \                             \                   \
                          |       |                         |            |     |                             |                   |
                          T       A                         C            T     T                             C                   G

Query       753   TTGATCGCTGTTCGTGATATGAGACAGACAGTTGCGGTGGGTGTCATCAAAGCAGTGGACAAGAAGGCTGCTGGAGCTGGCAAGGTCACCAAGTCTGCCCAGAAAGCTCAGAAGGCTAAATGAATATTATCCCTAATACCTGCCACCCCA  902
AU120002.1  746   ........                                                                                                                                                753
CJ434202.1  773   .-......                                                                                                                                                779
CR976939.1  628   ........C....                                                                                                                                           640
CB138394.1  620   ........C....                                                                                                                                           632
AU144980.1  418   ......................................................................................................................................................  264
                           \
                           |
                       TTGCT

Query       903   CTCTTAATCAGTGGTGGAAGAACGGTCTCAGAACTGTTTGTTTCAATTGGCCATTTAAGTTTAGTAGTAAAAGACTGGTTAATGATAACAATGCATCGTAAAACCTTCAGAAGGAAAGGAGAATgttttgtggaccactttggttttctt  1052
AU144980.1  263   ..N..................................................................................C................................................................  114

Query       1053  ttttgcgtgtggcagttttaagttattagtttttaaaatcagtactttttaATGGAAACAACTTGACCAAAAATTTGTCACAGAATTTTGAGACCCATTAAAAAAGTTAAAT  1164
AU144980.1  113   ................................................................................................................  2

Query= T12-29 [organism=Homo sapiens] eef1a1

Length=1240


                                                                    Score        E     Max
Sequences producing significant alignments:                        (Bits)     Value  ident

BY794942.2 BY794942 Homo sapiens eye Homo sapiens cDNA clone H...  2141       0.0    100%
DC527884.1 DC527884 chimpanzee skin cDNA library PstA Pan trog...  2135       0.0    100%
BX426149.2 BX426149 Homo sapiens NEUROBLASTOMA Homo sapiens cD...  2091       0.0    99%
BX418564.2 BX418564 Homo sapiens FETAL BRAIN Homo sapiens cDNA...  2025       0.0    98%
BX459625.2 BX459625 Homo sapiens FETAL BRAIN Homo sapiens cDNA...  1988       0.0    98%
BX420340.1 BX420340 Homo sapiens FETAL BRAIN Homo sapiens cDNA...  1984       0.0    99%
BX440470.1 BX440470 Homo sapiens FETAL BRAIN Homo sapiens cDNA...  1980       0.0    98%
AL518238.2 AL518238 Homo sapiens NEUROBLASTOMA Homo sapiens cD...  1978       0.0    97%
AL567209.3 AL567209 Homo sapiens FETAL BRAIN Homo sapiens cDNA...  1975       0.0    98%
BX418589.2 BX418589 Homo sapiens FETAL BRAIN Homo sapiens cDNA...  1962       0.0    98%

ALIGNMENTS

Query       2     TTTTTCGCAACGGGTTTGCCGCCAGAACACAGGTGTCGTGAAAACTACCCCTAAAAGCCAAAATGGGAAAGGAAATTGTTAAGGAAGTCAGCACTTACATTAAGAAAATTGGCTACAACCCCGACACAGTAGCATTTGTGCCAATTTCTG  151
BY794942.2  570                                                                        .................................................................................  650
BY794942.2  25                       ........................................................                                                                           80
DC527884.1  573                                                                        .................................................................................  653
DC527884.1  9     ...........................................................................                                                                           83
BX426149.2  1156                                                                       .................................................................................  1076
BX418564.2  1153                                                                           ..W....-.R......M.....K....-.R.-......S....-..........-R.....-...............  1083
BX459625.2  1118                                                                                                       ........C.MCM....C.....K....W....-KS......-.K  1076
BX420340.1  1117                                                                                                             ...M..M...................K.-..M.W...YT  1080
BX440470.1  1128                                                                                                  ....-.....-..-W.-.............-...-....-S.M...Y..-  1086
                                                                                                                              \
                                                                                                                              |
                                                                                                                              C
AL518238.2  1148                                                                                .....R..-....-.M.....-..........-..W...M....V.M.........................  1081
AL567209.3  1125                                                                       ........-K.W........R.....-....A.M.C............C................................  1046
                                                                                                    \
                                                                                                    |
                                                                                                    M
BX418589.2  1130                                                                                             ....-........-.K..-W.-M....C..M..K.-...-....S....-....-  1084
```

```
Query       152   GTTGGAATGGTGACAACATGCTGGAGCCAAGTGCTAACATGCCTTGGTTCAAGGGATGGAAAGTCACCCGTAAGGATGGCAATGCCAGTGGAACCACGCTGCTTGAGGCTCTGGACTGCATCCTACCACCAACTCGTCCAACTGACAAGC  301
BY794942.2  651   ......................................................................................................................................................  800
DC527884.1  654   ......................................................................................................................................................  803
BX426149.2  1075  ......................................................................................................................................................  926
BX418564.2  1082  ..-.....-..........................C......................-...........................................................................................  936
BX459625.2  1075  ......W.......................................K.A.....................................................................................................  925
                                \
                                |
                                T
BX420340.1  1079  ...D....K.-...........T..........Y...............................................................................A.............................R......  931
BX440470.1  1085  .K......-..............-.....................K...................................................................R....................................  938
AL518238.2  1080  -K.........A...........A....................................W.............................R...........................................................  931
                                                                                         \
                                                                                         |
                                                                                         A
AL567209.3  1045  .................................................................................................................K....................................  896
BX418589.2  1083  ........-..............-......-KK.............K.C.....................................................................................................  937

Query       302   CCTTGCGCCTGCCTCTCCAGGATGTCTACAAAATTGGTGGTATTGGTACTGTTCCTGTTGGCCGAGTGGAGACTGGTGTTCTCAAACCCGGTATGGTGGTCACCTTTGCTCCAGTCAACGTTACAACGGAAGTAAAATCTGTCGAAATGC  451
BY794942.2  801   ......................................................................................................................................................  950
DC527884.1  804   ......................................................................................................................................................  953
BX426149.2  925   ......................................................................................................................................................  776
BX418564.2  935   ..C...................................................................................................................................................  786
BX459625.2  924   ......................................................................................................................................................  775
BX420340.1  930   ......................................................................................................................................................  781
BX440470.1  937   ......................................................................................................................................................  788
AL518238.2  930   ......................................................................W...................................B.............................W.............  781
AL567209.3  895   ......................................................................................................................................................  746
BX418589.2  936   ......................................................................................................................................................  787

Query       452   ACCATGAAGCTTTGAGTGAAGCTCTTCCTGGGGACAATGTGGGCTTCAATGTCAAGAATGTGTCTGTCAAGGATGTTCGTCGTGGCAACGTTGCTGGTGACAGCAAAAATGACCCACCAATGGAAGCAGCTGGCTTCACTGCTCAGGTGA  601
BY794942.2  951   ......................................................................................................................................................  1100
DC527884.1  954   ......................................................................................................................................................  1103
BX426149.2  775   ......................................................................................................................................................  626
BX418564.2  785   ..................................................................C...................................................................................  636
BX459625.2  774   ......................................................................................................................................................  625
BX420340.1  780   ......................................................................................................................................................  631
BX440470.1  787   ......................................................................................................................................................  638
AL518238.2  780   ..................................................................................................A............................G......................  631
AL567209.3  745   ............................................................................................................-.........................................  597
BX418589.2  786   ......................................................................................................................................................  637

Query       602   TTATCCTGAACCATCCAGGCCAAATAAGCGCCGGCTATGCCCCTGTATTGGATTGCCACACGGCTCACATTGCATGCAAGTTTGCTGAGCTGAAGGAAAAGATTGATCGCCGTTCTGGTAAAAAGCTGGAAGATGGCCCTAAATTCTTGA  751
BY794942.2  1101  ......................................................................................................................................................  1251
                                \
                                |
                                G
DC527884.1  1104  ......................................................................................................................................................  1253
BX426149.2  625   ......................................................................................................................................................  476
BX418564.2  635   ...................................................................................................M..................................................  486
BX459625.2  624   ......................................................................................................................................................  475
BX420340.1  630   ......................................................................................................................................................  481
BX440470.1  637   ......................................................................................................................................................  487
                                                                                                                       \
                                                                                                                       |
                                                                                                                       C
AL518238.2  630   ......................................................................Y...............................................................................  481
AL567209.3  596   ......................................................................................................................................................  447
BX418589.2  636   ..........................................................................................................Y.S.........................................  487

Query       752   AGTCTGGTGATGCTGCCATTGTTGATATGGTTCCTGGCAAGCCCATGTGTGTTGAGAGCTTCTCAGACTATCCACCTTTGGGTCGCTTTGCTGTTCGTGATATGAGACAGACAGTTGCGGTGGTTGTCATCAAAGCAGTGGACAAGAAGG  901
BY794942.2  1252  ...........................................................................................................................G..........................  1401
DC527884.1  1254  ...........................................................................................................................G.A........................  1403
BX426149.2  475   ...........................................................................................................................G..........................  326
BX418564.2  485   ...........................................................................................................................G..........................  336
BX459625.2  474   ...........................................................................................................................G..........................  325
BX420340.1  480   ...........................................................................................................................G..........................  331
BX440470.1  486   ...........................................................................................................................G..........................  337
AL518238.2  480   ...........................................................................................................................G..........................  331
AL567209.3  446   ...............................K...........................................................................................G..........................  297
BX418589.2  486   ...........................................................................................................................G..........................  337

Query       902   CTGCTGGAGCTGGCAAGGTCACCAAGTCTGCCCAGAAAGCTCAGAAGGCTAAATGAATATTATCCCTAATACCTGCCACCCCACTCTTAATCAGTGGTGGAAGAACGGTCTCAGAACTGTTTGTTTCAATTGGCCATTTAAGTTTAGTAG  1051
BY794942.2  1402  ......................................................................................................................................................  1551
DC527884.1  1404  .......................................................................................C..............................................................  1553
BX426149.2  325   ......................................................................................................................................................  176
BX418564.2  335   ......................................................................................................................................................  186
BX459625.2  324   ......................................................................................................................................................  175
BX420340.1  330   ......................................................................................................................................................  181
BX440470.1  336   ......................................................................................................................................................  187
AL518238.2  330   .......G..................................................................................................G...................................  181
```

```
AL567209.3  296   ..........................................M......Y....................................................................................................  146
                                                                                                                  \
                                                                                                                  |
                                                                                                                  A
BX418589.2  336   ......................................................................................................................................................  187

Query       1052  TAAAAGACTGGTTAATGATAACAATGCATCGTAAAACCTTCAGAAGGAAAGGAGAATgttttgtggaccactttggttttctttttttgcgtgtggcagttttaagttattagtttttaaaatcagtacttttttaATGGAAACAACTTGAC  1201
BY794942.2  1552  ......................................................................................................................................................  1701
DC527884.1  1554  ......................................................................................................................................................  1703
BX426149.2  175   .........................................................................................................................................-.......-....  28
BX418564.2  185   ......................................................................................................................................................  36
BX459625.2  174   ......................................................................................................................................................  25
BX420340.1  180   ......................................................................................................................................................  31
BX440470.1  186   ......................................................................................................................................................  37
AL518238.2  180   ..............................................................................................................................................N.......  31
AL567209.3  145   ....................................................................................................................................-....-..-...-...    1
                                                                                      \
                                                                                      |
                                                                                      G
BX418589.2  186   ......................................................................................................................................................  37

Query       1202  CAAAAATTTGTCACAGAATTTTGACCCATTAAAAAAGTT  1240
BY794942.2  1702  .......................................  1742
                                        \
                                        |
                                       GA
DC527884.1  1704  .......................................  1744
                                        \
                                        |
                                       GA
BX426149.2  27    .G......G.-..G..........                 5
BX418564.2  35    .....................M............       1
                                        \
                                        |
                                        A
BX459625.2  24    ........................                 1
BX420340.1  30    ........................                 7
BX440470.1  36    ..................................       1
                                          \
                                          |
                                         GA
AL518238.2  30    ......N.................                 6
                              \
                              |
                              N
BX418589.2  36    .....................D............       1
                                          \
                                          |
                                         GA
```

Query= T13-27 [organism=Homo sapiens] eef1a1

Length=1489

Sequences producing significant alignments:	Score (Bits)	E Value	Max ident
DC527884.1 DC527884 chimpanzee skin cDNA library PstA Pan trog...	2163	0.0	100%
BY794942.2 BY794942 Homo sapiens eye Homo sapiens cDNA clone H...	2134	0.0	100%
BX426150.2 BX426150 Homo sapiens NEUROBLASTOMA Homo sapiens cD...	2037	0.0	99%
BU902062.1 AGENCOURT_10127553 NIH_MGC_71 Homo sapiens cDNA clo...	1997	0.0	98%
BX402801.2 BX402801 Homo sapiens T CELLS (JURKAT CELL LINE) CO...	1988	0.0	98%
BX402793.2 BX402793 Homo sapiens T CELLS (JURKAT CELL LINE) CO...	1973	0.0	98%
BX440468.2 BX440468 Homo sapiens FETAL BRAIN Homo sapiens cDNA...	1971	0.0	98%
AL513590.3 AL513590 Homo sapiens PLACENTA Homo sapiens cDNA cl...	1967	0.0	97%
AL513664.3 AL513664 Homo sapiens NEUROBLASTOMA Homo sapiens cD...	1962	0.0	98%
AL560156.3 AL560156 Homo sapiens B CELLS (RAMOS CELL LINE) Hom...	1960	0.0	98%

ALIGNMENTS

```
Query       2     TTTTTCGCAACGGGTTTGCCGCCAGAACACAGGTGTCGTGAAAACTACCCCTAAAAGCCAAAATGGGAAAGGAAAAGACTCATATCAACATTGTCGTCATTGGACACGTAGATTCGGGCAAGTCCACCACTACTGGCCATCTGATCTATA  151
DC527884.1  9     ......................................................................................................................................................  158
BY794942.2  25                       ...................................................................................................................................  155
BX426150.2  1                             ..T...........................................................................................................................  126
BU902062.1  1                  .........................................................................................................................................  137
BX402801.2  1               ....A.........................................................................................................................................  140
BX402793.2  6                     ......................................................................................................................................  139
BX440468.2  1                         ..................................................................................................................................  130
AL513590.3  2                                  .........................................................................................................................  123
                                                                                               \
                                                                                               |
```

```
                                                                                             C
AL513664.3 1                        ..................................................................................................................................  130
AL560156.3 1                                          ................................................................................................................  112

Query      152  AATGCGGTGGCATCGACAAAAGAACCATTGAAAAATTTGAGAAGGAGGCTGCTGAGATGGGAAAGGGCTCCTTCAAGTATGCCTGGGTCTTGGATAAACTGAAAGCTGAGCGTGAACGTGGTATCACCATTGATATCTCCTTGTGGAAAT  301
DC527884.1 159  ............................................................................................................................T.........................  308
BY794942.2 156  ......................................................................................................................................................  305
BX426150.2 127  ......................................................................................................................................................  276
BU902062.1 138  ......................................................................................................................................................  287
BX402801.2 141  ......................................................................................................................................................  290
BX402793.2 140  ......................................................................................................................................................  289
BX440468.2 131  ......................................................................................................................................................  280
AL513590.3 124  ..........................................................................................................B...........................................  273
AL513664.3 131  ............................................................................................................................Y.........................  280
AL560156.3 113  ......................................................................................................................................................  262

Query      302  TTGAGACCAGCAAGTACTATGTGACTATCATTGATGCCCCAGGACACAGAGACTTTATCAAAAACATGATTACAGGGACATCTCAGGCTGACTGTGCTGTCCTGATTGTTGCTGCTGGTGTTGGTGAATTTGAAGCTGGTATCTCCAAGA  451
DC527884.1 309  .......................................................C..............................................................................................  458
BY794942.2 306  ......................................................................................................................................................  455
BX426150.2 277  ......................................................................................................................................................  426
BU902062.1 288  ......................................................................................................................................................  437
BX402801.2 291  ......................................................................................................................................................  440
BX402793.2 290  ......................................................................................................................................................  439
BX440468.2 281  ......................................................................................................................................................  430
AL513590.3 274  .............................................................................................................K..B..B.....K............................  423
AL513664.3 281  ...................................................................................................................Y..................................  430
AL560156.3 263  ......................................................................................................................................................  412

Query      452  ATGGGCAGACCCGAGAGCATGCCCTTCTGGCTTACACACTGGGTGTGAAACAACTAATTGTCGGTGTTAACAAAATGGATTCCACTGAGCCACCCTACAGCCAGAAGAGATATGAGGAAATTGTTAAGGAAGTCAGCACTTACATTAAGA  601
DC527884.1 459  ......................................................................................................................................................  608
BY794942.2 456  ......................................................................................................................................................  605
BX426150.2 427  ......................................................................................................................................................  576
BU902062.1 438  ......................................................................................................................................................  587
BX402801.2 441  ......................................................................................................................................................  590
BX402793.2 440  ......................................................................................................................................................  589
BX440468.2 431  ......................................................................................................................................................  580
AL513590.3 424  ......................................................................................................................................................  573
AL513664.3 431  ......................................................................................................................................................  580
AL560156.3 413  ......................................................................................................................................................  562

Query      602  AAATTGGCTACAACCCCGACACAGTAGCATTTGTGCCAATTTCTGGTTGGAATGGTGACAACATGCTGGAGCCAAGTGCTAACATGCCTTGGTTCAAGGGATGGAAAGTCACCCGTAAGGATGGCAATGCCAGTGGAACCACGCTGCTTG  751
DC527884.1 609  ......................................................................................................................................................  758
BY794942.2 606  ......................................................................................................................................................  755
BX426150.2 577  ......................................................................................................................................................  726
BU902062.1 588  ......................................................................................................................................................  737
BX402801.2 591  ......................................................................................................................................................  740
BX402793.2 590  ......................................................................................................................................................  739
BX440468.2 581  ......................................................................................................................-...............................  729
AL513590.3 574  ..................................................................Y............Y......................................-...............T...............  722
AL513664.3 581  ......................................................................................................................................K...............  730
AL560156.3 563  ......................................................................................................................................................  712

Query      752  AGGCTCTGGACTGCATCCTACCACCAACTCGTCCAACTGACAAGCCCTTGCGCCTGCCTCTCCAGGATGTCTACAAAATTGGTGGTATTGGTACTGTTCCTGTTGGCCGAGTGGAGACTGGTGTTCTCAAACCCGGTATGGTGGTCACCT  901
DC527884.1 759  ......................................................................................................................................................  908
BY794942.2 756  ......................................................................................................................................................  905
BX426150.2 727  ......................................................................................................................................................  876
BU902062.1 738  ..................................................................................................................................C..G................  887
BX402801.2 741  ......................................................................................................................................................  890
BX402793.2 740  ......................................................................................................................................................  889
BX440468.2 730  ......................................................................................................................................................  879
AL513590.3 723  .......................................................................................................Y..............................................  872
AL513664.3 731  ......................................................................................................................................................  880
AL560156.3 713  ......................................................................................................................................................  862

Query      902  TTGCTCCAGTCAACGTTACAACGGAAGTAAAATCTGTCGAAATGCACCATGAAGCTTTGAGTGAAGCTCTTCCTGGGGACAATGTGGGCTTCAATGTCAAGAATGTGTCTGTCAAGGATGTTCGTCGTGGCAACGTTGCTGGTGACAGCA  1051
DC527884.1 909  ......................................................................................................................................................  1058
BY794942.2 906  ......................................................................................................................................................  1055
BX426150.2 877  ...................................................................................................................................-...............-..  1025
                                                                                          \
                                                                                          |
                                                                                          G
BU902062.1 888  ............................-..................................................AC...........C...C..........................C..C.......................  1037
                                                                                                                                                \
                                                                                                                                                |
                                                                                                                                                G
BX402801.2 891  ............................W......................................Y.......................Y.....T............K.T..A................M.K............S..  1042
                                                                                                     \                                          \
                                                                                                     |                                          |
                                                                                                     G                                          K
BX402793.2 890  ............................S..................................-.................................TM.A..............................................S..  1038
BX440468.2 880  ..............................................................................................W....................R...............M-..............S-.  1027
AL513590.3 873  ..........T.................T........Y........................R..................................M............T....................-...............C..  1021
```

```
AL513664.3  881   ..........T.................W.................................K..................................A............K.T........G........GC...............S.-  1030
                                                                                                                                                           \
                                                                                                                                                           |
                                                                                                                                                           G
AL560156.3  863   ....................................................................................................................................-..............S..  1013
                                                                                           \                                                             \
                                                                                           |                                                             |
                                                                                           T                                                             T

Query       1052  AAAATGACCCACCAATGGAAGCAGCTGGCTTCACTGCTCAGGTGATTATCCTGAACCATCCAGGCCAAATAAGCGCCGGCTATGCCCCTGTATTGGATTGCCACACGGCTCACATTGCATGCAAGTCTGCCCAGAAAGCTCAGAAGGCTA  1201
DC527884.1  1059  ..............................................................................................................................T...                    1188
DC527884.1  1426                                                                                                                           .............................  1454
BY794942.2  1056  ..............................................................................................................................T...                    1186
                                                                             \
                                                                             |
                                                                             G
BY794942.2  1424                                                                                                                           .............................  1452
BX426150.2  1026  .........-.........-.........-...................-..................-.......-.......-................-......T.-.............                          1140
BU902062.1  1038  .......................C.G......-.........-.........................T....-.....G.......-..A...........C..G..T...-.............                        1162
                                                       \                          \                \              \
                                                       |                          |                |              |
                                                       C                          G                T              A
BX402801.2  1043  ...W-..A.................K..Y...............T..W......................W.....S.S-....-.........K.W.......                                              1144
                                                                                                                     \
                                                                                                                     |
                                                                                                                     M
BX402793.2  1039  ...WKR...M-.....................M....Y.........-......M.M-...M...M-................S...                                                               1121
BX440468.2  1028  ..........M.....................M......M...........-....MWC..R.-.....A..M-..S..-.......Y-.........                                                    1120
AL513590.3  1022  .........................Y.........K.C.........W......M.M.-................................T.......T...M.Y..S.Y.A.............T...                    1151
                                                                                  \
                                                                                  |
                                                                                  C
AL513664.3  1031  .........................G...........Y.........W.M...................WR.....S.SY-......-...T.......                                                    1127
AL560156.3  1014  ................K.R............Y............W.-.....K..M.M..........W-..........W-.....Y-.....                                                        1103

Query       1202  AATGAATATTATCCCTAATACCTGCCACCCCACTCTTAATCAGTGGTGGAAGAACGGTCTCAGAACTGTTTGTTTCAATTGGCCATTTAAGTTTAGTAGTAAAAGACTGGTTAATGATAACAATGCATCGTAAAACCTTCAGAAGGAAAG  1351
DC527884.1  1455  ....................................C.....................................................................................................................  1604
BY794942.2  1453  ......................................................................................................................................................  1602

Query       1352  GAGAATgttttgtggaccactttggttttctttttttgcgtgtggcagttttaagttattagtttttaaaatcagtactttttaATGGAAACAACTTGACCAAAAATTTGTCACAGAATTTTGAGACCCATTAAAAAAG  1489
DC527884.1  1605  ..........................................................................................................................................  1742
BY794942.2  1603  ..........................................................................................................................................  1740

Query= T14-34 [organism=Homo sapiens] eef1a1

Length=1467


                                                                  Score        E     Max
Sequences producing significant alignments:                      (Bits)     Value  ident

BX440539.2 BX440539 Homo sapiens FETAL BRAIN Homo sapiens cDNA...  1465       0.0    100%
DR763375.1 HESC4_151_G10.g1_A037 NIH_MGC_262 Homo sapiens cDNA...  1463       0.0    100%
DR763172.1 HESC4_149_E01.g1_A037 NIH_MGC_262 Homo sapiens cDNA...  1463       0.0    100%
DR762061.1 HESC4_136_A03.g1_A037 NIH_MGC_262 Homo sapiens cDNA...  1463       0.0    100%
DR158661.1 HESC2_92_H12.g1_A035 NIH_MGC_258 Homo sapiens cDNA ...  1463       0.0    100%
CX870789.1 HESC4_49_D11.g1_A037 NIH_MGC_262 Homo sapiens cDNA ...  1463       0.0    100%
BU846433.1 AGENCOURT_10413493 NIH_MGC_109 Homo sapiens cDNA cl...  1463       0.0    100%
AU119155.1 AU119155 HEMBA1 Homo sapiens cDNA clone HEMBA100515...  1463       0.0    100%
DA738169.1 DA738169 NT2RP4 Homo sapiens cDNA clone NT2RP400261...  1461       0.0    100%
DA418936.1 DA418936 CD34C1 Homo sapiens cDNA clone CD34C100001...  1461       0.0    100%

ALIGNMENTS

Query       1    CTTTTTCGCAACGGGTTTGCCGCCAGAACACAGGTGTCGTGAAAACTACCCCTAAAAGCCAAAATGGGAAAGGAAAAGACTCATATCAACATTGTCGTCATTGGACACGTAGATTCGGGCAAGTCCACCACTACTGGCCATCTGATCTAT  150
BX440539.2  40   ......................................................................................................................................................  189
DR763375.1  6     .....................................................................................................................................................  154
DR763172.1  3     .....................................................................................................................................................  151
DR762061.1  19    .....................................................................................................................................................  167
DR158661.1  11    .....................................................................................................................................................  159
CX870789.1  7     .....................................................................................................................................................  155
BU846433.1  20    .....................................................................................................................................................  168
AU119155.1  7     .....................................................................................................................................................  155
DA738169.1  4    ......................................................................................................................................................  153
DA418936.1  1    ......................................................................................................................................................  150

Query       151  AAATGCGGTGGCATCGACAAAAGAACCATTGAAAAATTTGAGAAGGAGGCTGCTGAGATGGGAAAGGGCTCCTTCAAGTATGCCTGGGTCTTGGATAAACTGAAAGCTGAGCGTGAACGTGGTATCACCATTGATATCTCCTTGTGGAAA  300
BX440539.2  190  ......................................................................................................................................................  339
DR763375.1  155  ......................................................................................................................................................  304
DR763172.1  152  ......................................................................................................................................................  301
DR762061.1  168  ......................................................................................................................................................  317
DR158661.1  160  ......................................................................................................................................................  309
```

```
CX870789.1  156  ......................................................................................................................................................  305
BU846433.1  169  ......................................................................................................................................................  318
AU119155.1  156  ......................................................................................................................................................  305
DA738169.1  154  ......................................................................................................................................................  303
DA418936.1  151  ......................................................................................................................................................  300

Query       301  TTTGAGACCAGCAAGTACTATGTGACTATCATTGATGCCCCAGGACACAGAGACTTTATCAAAAACATGATTACAGGGACATCTCAGGCTGACTGTGCTGTCCTGATTGTTGCTGCTGGTGTTGGTGAATTTGAAGCTGGTATCTCCAAG  450
BX440539.2  340  ......................................................................................................................................................  489
DR763375.1  305  ......................................................................................................................................................  454
DR763172.1  302  ......................................................................................................................................................  451
DR762061.1  318  ......................................................................................................................................................  467
DR158661.1  310  ......................................................................................................................................................  459
CX870789.1  306  ......................................................................................................................................................  455
BU846433.1  319  ......................................................................................................................................................  468
AU119155.1  306  ......................................................................................................................................................  455
DA738169.1  304  ......................................................................................................................................................  453
DA418936.1  301  ......................................................................................................................................................  450

Query       451  AATGGGCAGACCCGAGAGCATGCCCTTCTGGCTTACACACTGGGTGTGAAACAACTAATTGTCGGTGTTAACAAAATGGATTCCACTGAGCCACCCTACAGCCAGAAGAGATATGAGGAAATTGTTAAGGAAGTCAGCACTTACATTAAG  600
BX440539.2  490  ......................................................................................................................................................  639
DR763375.1  455  ......................................................................................................................................................  604
DR763172.1  452  ......................................................................................................................................................  601
DR762061.1  468  ......................................................................................................................................................  617
DR158661.1  460  ......................................................................................................................................................  609
CX870789.1  456  ......................................................................................................................................................  605
BU846433.1  469  ......................................................................................................................................................  618
AU119155.1  456  ......................................................................................................................................................  605
DA738169.1  454  ......................................................................................................................................................  603
DA418936.1  451  ......................................................................................................................................................  600

Query       601  AAAATTGGCTACAACCCCGACACAGTAGCATTTGTGCCAATTTCTGGTTGGAATGGTGACAAACATGCTGGAGCCAAGTGCTAACATGCCTTGGTTCAAGGGATGGAAAGTCACCCGTAAGGATGGCAATGCCAGTGGAACCACGCTGCT  750
BX440539.2  640  ............................................................-.........................................................................................  788
DR763375.1  605  ............................................................-.........................................................................................  753
DR763172.1  602  ............................................................-.........................................................................................  750
DR762061.1  618  ............................................................-.........................................................................................  766
DR158661.1  610  ............................................................-.........................................................................................  758
CX870789.1  606  ............................................................-.........................................................................................  754
BU846433.1  619  ............................................................-.........................................................................................  767
AU119155.1  606  ............................................................-.........................................................................................  754
DA738169.1  604  ............................................................-.........................................................................................  753
                                                                                                                                  \
                                                                                                                                  |
                                                                                                                                  C
DA418936.1  601  ............................................................-.........................................................................................  749

Query       751  TGAGGCTCTGGACTGCATCCTACCACCAACTCGTCCAACTGACAAGC  797
BX440539.2  789  ...............................................  835
DR763375.1  754  ...............................................  800
DR763172.1  751  ...............................................  797
DR762061.1  767  ...............................................  813
DR158661.1  759  ...............................................  805
CX870789.1  755  ...............................................  801
BU846433.1  768  ...............................................  814
AU119155.1  755  ...............................................  801
DA738169.1  754  ...............................................  800
DA418936.1  750  ..................N............................  796

  Database: Database of GenBank+EMBL+DDBJ sequences from EST Divisions
    Posted date:  Feb 24, 2019  1:46 AM
  Number of letters in database: 43,200,890,307
  Number of sequences in database:  77,566,966

Lambda      K        H
    1.33    0.621     1.12
Gapped
Lambda      K        H
    1.28    0.460    0.850
Matrix: blastn matrix:1 -2
Gap Penalties: Existence: 0, Extension: 0
Number of Sequences: 77566966
Number of Hits to DB: 11823009
Number of extensions: 1013502
Number of successful extensions: 1013502
Number of sequences better than 10: 34556
Number of HSP's better than 10 without gapping: 0
Number of HSP's gapped: 528608
Number of HSP's successfully gapped: 518797
Length of database: 43200890307
A: 0
X1: 13 (25.0 bits)
X2: 32 (59.1 bits)
X3: 54 (99.7 bits)
```

```
S1: 13 (25.1 bits)

BLASTN 2.9.0+
Reference: Zheng Zhang, Scott Schwartz, Lukas Wagner, and
Webb Miller (2000), "A greedy algorithm for aligning DNA
sequences", J Comput Biol 2000; 7(1-2):203-14.


RID: 8PVG24DZ015


Database: Database of GenBank+EMBL+DDBJ sequences from EST Divisions
           77,566,966 sequences; 43,200,890,307 total letters
Query= N1-4 [organism=Homo sapiens] eef1a1

Length=1109


                                                                  Score        E     Max
Sequences producing significant alignments:                       (Bits)     Value  ident

CR982215.1 CR982215 RZPD no.9016 Homo sapiens cDNA clone RZPDp...  1456       0.0    100%
BX443032.2 BX443032 Homo sapiens B CELLS (RAMOS CELL LINE) Hom...  1456       0.0    100%
BX421307.2 BX421307 Homo sapiens B CELLS (RAMOS CELL LINE) Hom...  1456       0.0    100%
BX353545.2 BX353545 Homo sapiens NEUROBLASTOMA COT 25-NORMALIZ...  1456       0.0    100%
CX167214.1 HESC2_49_G11.g1_A035 NIH_MGC_258 Homo sapiens cDNA ...  1456       0.0    100%
CA771972.1 io93g08.x1 HR85 islet Homo sapiens cDNA clone IMAGE...  1456       0.0    100%
BU844361.1 AGENCOURT_10414634 NIH_MGC_109 Homo sapiens cDNA cl...  1456       0.0    100%
BX453401.2 BX453401 Homo sapiens T CELLS (JURKAT CELL LINE) Ho...  1454       0.0    100%
CR996534.1 CR996534 RZPD no.9017 Homo sapiens cDNA clone RZPDp...  1452       0.0    100%
BU845975.1 AGENCOURT_10413042 NIH_MGC_109 Homo sapiens cDNA cl...  1452       0.0    100%

ALIGNMENTS

Query       322   TTTGAGTGAAGCTCTTCCTGGGGACAATGTGGGCTTCAATGTCAAGAATGTGTCTGTCAAGGATGTTCGTCGTGGCAACGTTGCTGGTGACAGCAAAAATGACCCACCAATGGAAGCAGCTGGCTTCACTGCTCAGGTGATTATCCTGAA  471
CR982215.1  6     ......................................................................................................................................................  155
BX443032.2  143   ......................................................................................................................................................  292
BX421307.2  117   ......................................................................................................................................................  266
BX353545.2  22    ......................................................................................................................................................  171
CX167214.1  788   ......................................................................................................................................................  639
CA771972.1  788   ......................................................................................................................................................  639
BU844361.1  795   ......................................................................................................................................................  646
BX453401.2  17    ......................................................................................................................................................  166
CR996534.1  63    ......................................................................................................................................................  212
BU845975.1  786   ......................................................................................................................................................  637

Query       472   CCATCCAGGCCAAATAAGCGCCGGCTATGCCCCTGTATTGGATTGCCACACGGCTCACATTGCATGCAAGTTTGCTGAGCTGAAGGAAAAGATTGATCGCCGTTCTGGTAAAAAGCTGGAAGATGGCCCTAAATTCTTGAAGTCTGGTGA  621
CR982215.1  156   ......................................................................................................................................................  305
BX443032.2  293   ......................................................................................................................................................  442
BX421307.2  267   ......................................................................................................................................................  416
BX353545.2  172   ......................................................................................................................................................  321
CX167214.1  638   ......................................................................................................................................................  489
CA771972.1  638   ......................................................................................................................................................  489
BU844361.1  645   ......................................................................................................................................................  496
BX453401.2  167   ......................................................................................................................................................  316
CR996534.1  213   ......................................................................................................................................................  362
BU845975.1  636   ......................................................................................................................................................  487

Query       622   TGCTGCCATTGTTGATATGGTTCCTGGCAAGCCCATGTGTGTTGAGAGCTTCTCAGACTATCCACCTTTGGGTCGCTTTGCTGTTCGTGATATGAGACAGACAGTTGCGGTGGGTGTCATCAAAGCAGTGGACAAGAAGGCTGCTGGAGC  771
CR982215.1  306   ......................................................................................................................................................  455
BX443032.2  443   ......................................................................................................................................................  592
BX421307.2  417   ......................................................................................................................................................  566
BX353545.2  322   ......................................................................................................................................................  471
CX167214.1  488   ......................................................................................................................................................  339
CA771972.1  488   ......................................................................................................................................................  339
BU844361.1  495   ......................................................................................................................................................  346
BX453401.2  317   ......................................................................................................................................................  466
CR996534.1  363   ......................................................................................................................................................  512
BU845975.1  486   ......................................................................................................................................................  337

Query       772   TGGCAAGGTCACCAAGTCTGCCCAGAAAGCTCAGAAGGCTAAATGAATATTATCCCTAATACCTGCCACCCCACTCTTAATCAGTGGTGGAAGAACGGTCTCAGAACTGTTTGTTTCAATTGGCCATTTAAGTTTAGTAGTAAAAGACTG  921
CR982215.1  456   ......................................................................................................................................................  605
BX443032.2  593   ......................................................................................................................................................  742
BX421307.2  567   ......................................................................................................................................................  716
BX353545.2  472   ......................................................................................................................................................  621
CX167214.1  338   ......................................................................................................................................................  189
CA771972.1  338   ......................................................................................................................................................  189
BU844361.1  345   ......................................................................................................................................................  196
BX453401.2  467   ......................................................................................................................................................  616
CR996534.1  513   ......................................................................................................................................................  662
BU845975.1  336   ......................................................................................................................................................  187
```

```
Query       922   GTTAATGATAACAATGCATCGTAAAACCTTCAGAAGGAAAGGAGAATgttttgtggaccactttggttttctttttgcgtgtggcagttttaagttattagtttttaaaatcagtactttttaATGGAAACAACTTGACCAAAAATTTG  1071
CR982215.1  606   ......................................................................................................................................................  755
BX443032.2  743   ......................................................................................................................................................  892
BX421307.2  717   ......................................................................................................................................................  866
BX353545.2  622   ......................................................................................................................................................  771
CX167214.1  188   ......................................................................................................................................................  39
CA771972.1  188   ......................................................................................................................................................  39
BU844361.1  195   ......................................................................................................................................................  46
BX453401.2  617   ......................................................................................................................................................  766
CR996534.1  663   ......................................................................................................................................................  812
BU845975.1  186   ......................................................................................................................................................  37

Query       1072  TCACAGAATTTTGAGACCCATTAAAAAAGTTAAATGAG  1109
CR982215.1  756   ......................................  793
BX443032.2  893   ......................................  930
BX421307.2  867   ......................................  904
BX353545.2  772   ......................................  809
CX167214.1  38    ......................................  1
CA771972.1  38    ......................................  1
BU844361.1  45    ......................................  8
BX453401.2  767   .....................................   803
CR996534.1  813   ....................................    848
BU845975.1  36    ....................................    1

Query= N2-14 [organism=Homo sapiens] eef1a1

Length=987


                                                                  Score        E     Max
Sequences producing significant alignments:                      (Bits)     Value  ident

CN389362.1 17000599951108 GRN_PRENEU Homo sapiens cDNA 5', mRN...  952        0.0    100%
CK843266.1 UI-R-AA1-aad-f-05-0-UI.s10 UI-R-AA1 Rattus norvegic...  948        0.0    100%
CK843262.1 UI-R-AA1-aad-c-09-0-UI.s10 UI-R-AA1 Rattus norvegic...  948        0.0    100%
CK843256.1 UI-R-AA1-aac-g-06-0-UI.s10 UI-R-AA1 Rattus norvegic...  948        0.0    100%
CB373762.1 TgESTzyg83g02.x1 TgME49 3 day invitro bradyzoite To...  948        0.0    100%
BQ883373.1 AGENCOURT_7982297 Lupski_dorsal_root_ganglion Homo ...  948        0.0    100%
BQ788143.1 il48f10.x1 HR85 islet Homo sapiens cDNA clone IMAGE...  948        0.0    100%
BQ787972.1 il45h02.x1 HR85 islet Homo sapiens cDNA clone IMAGE...  948        0.0    100%
BQ787616.1 im13c06.x1 Human insulinoma Homo sapiens cDNA clone...  948        0.0    100%
BQ787579.1 im12e10.x1 Human insulinoma Homo sapiens cDNA clone...  948        0.0    100%

ALIGNMENTS

Query       467  GAGAGCATGCCCTAAATTCTTGAAGTCTGGTGATGCTGCCATTGTTGATATGGTTCCTGGCAAGCCCATGTGTGTTGAGAGCTTCTCAGACTATCCACCTTTGGGTCGCTTTGCTGTTCGTGATATGAGACAGACAGTTGCGGTGGGTGT  616
CN389362.1  26   .....-................................................................................................................................................  175
                         \
                         |
                         G
CK843266.1  529          ..............................................................................................................................................  388
CK843262.1  529          ..............................................................................................................................................  388
CK843256.1  529          ..............................................................................................................................................  388
CB373762.1  525          ..............................................................................................................................................  384
BQ883373.1  60           ..............................................................................................................................................  201
BQ788143.1  514          ..............................................................................................................................................  373
BQ787972.1  517          ..............................................................................................................................................  376
BQ787616.1  532          ..............................................................................................................................................  391
BQ787579.1  528          ..............................................................................................................................................  387

Query       617  CATCAAAGCAGTGGACAAGAAGGCTGCTGGAGCTGGCAAGGTCACCAAGTCTGCCCAGAAAGCTCAGAAGGCTAAATGAATATTATCCCTAATACCTGCCACCCCACTCTTAATCAGTGGTGGAAGAACGGTCTCAGAACTGTTTGTTTC  766
CN389362.1  176  ......................................................................................................................................................  325
CK843266.1  387  ......................................................................................................................................................  238
CK843262.1  387  ......................................................................................................................................................  238
CK843256.1  387  ......................................................................................................................................................  238
CB373762.1  383  ......................................................................................................................................................  234
BQ883373.1  202  ......................................................................................................................................................  351
BQ788143.1  372  ......................................................................................................................................................  223
BQ787972.1  375  ......................................................................................................................................................  226
BQ787616.1  390  ......................................................................................................................................................  241
BQ787579.1  386  ......................................................................................................................................................  237

Query       767  AATTGGCCATTTAAGTTTAGTAGTAAAAGACTGGTTAATGATAACAATGCATCGTAAAACCTTCAGAAGGAAAGGAGAATgttttgtggaccactttggttttctttttgcgtgtggcagttttaagttattagtttttaaaatcagta  916
CN389362.1  326  ......................................................................................................................................................  475
CK843266.1  237  ......................................................................................................................................................  88
CK843262.1  237  ......................................................................................................................................................  88
CK843256.1  237  ......................................................................................................................................................  88
CB373762.1  233  ......................................................................................................................................................  84
BQ883373.1  352  ......................................................................................................................................................  501
BQ788143.1  222  ......................................................................................................................................................  73
BQ787972.1  225  ......................................................................................................................................................  76
BQ787616.1  240  ......................................................................................................................................................  91
```

```
BQ787579.1  236  ......................................................................................................................................................  87

Query       917  ctttttaATGGAAACAACTTGACCAAAAATTTGTCACAGAATTTTGAGACCCATTAAAAAGTTAAATGAG  987
CN389362.1  476  ......................................................................  546
CK843266.1  87   ......................................................................  17
CK843262.1  87   ......................................................................  17
CK843256.1  87   ......................................................................  17
CB373762.1  83   ......................................................................  13
BQ883373.1  502  ......................................................................  572
BQ788143.1  72   ......................................................................  2
BQ787972.1  75   ......................................................................  5
BQ787616.1  90   ......................................................................  20
BQ787579.1  86   ......................................................................  16

Query= N3-8 [organism=Homo sapiens] eef1a1

Length=926


                                                                   Score        E     Max
Sequences producing significant alignments:                       (Bits)     Value  ident

DC635691.1 DC635691 macaque bone marrow cDNA library QbmA Maca...  1504       0.0    96%
AI679280.1 tu62f06.x1 NCI_CGAP_Gas4 Homo sapiens cDNA clone IM...  1077       0.0    99%
CA431918.1 UI-H-FT0-bhl-c-18-0-UI.s1 NCI_CGAP_FT0 Homo sapiens...  1075       0.0    99%
BU070293.1 im11h09.x1 Human insulinoma Homo sapiens cDNA clone...  1075       0.0    99%
AI588866.1 tq28e03.x1 NCI_CGAP_Ut1 Homo sapiens cDNA clone IMA...  1075       0.0    99%
AI554314.1 tq05f11.x1 NCI_CGAP_Ut3 Homo sapiens cDNA clone IMA...  1075       0.0    99%
AI336170.1 qt44b04.x1 Soares_fetal_lung_NbHL19W Homo sapiens c...  1075       0.0    99%
CT005089.1 CT005089 RZPD no.9017 Homo sapiens cDNA clone RZPDp...  1074       0.0    100%
CR999820.1 CR999820 RZPD no.9017 Homo sapiens cDNA clone RZPDp...  1074       0.0    100%
CR999128.1 CR999128 RZPD no.9017 Homo sapiens cDNA clone RZPDp...  1074       0.0    100%

ALIGNMENTS

Query       1    CTTTTTCGCAACGGGTTTGCCGCCAGAACACAGGTGTCGTGAAAACTACCCCTAAAAGCCAAAATGGGAAAGGAAAAGACTCATATCAACATTGTCGTCATTGGACACGTAGATTCGGGCAAGTCCACCACTACTGGCCACCTGATCTAT  150
DC635691.1  1    .........-....................................................................................................................................T........C  149

Query       151  AAATGCGGTGGCATCGACAAAAGAACCATTGAAAAATTTGAGAAGGAGGCTGCTGAGATGGGAAAGGGCTCCTTCAAGTATTCCTGGGTCTTGGATAAACTGAAAGCTGAGCGTGAACGTGGTATCACCATTGATATCTCCTTGTGGAAA  300
DC635691.1  150  .................................................................................G...............................G....................................  299

Query       301  TTTGAGACCAGCAAGTACTATGTGACTATCATTGATGCCCCAGGACACATTGCATGCAAGTTTGCTGAGCTGAAGGAAAAGATTGATCGCCGTTCTGGTAAAAAGCTGGAAGATGGCCCTAAATTCTTGAAGTCTGGTGATGCTGCCATT  450
DC635691.1  300  .................T..........................T...........................................................A.............................................  467
                                                          \\\\
                                                          ||||
                                                   TGTGTTGG|||
                                                           |||
                                                          TT||
                                                            ||
                                                      CCACACA|
                                                             |
                                                             C
AI679280.1  604                                ..............T.........................................................................................................  481
                                                  \      \ \ \
                                                  |      | | |
                                                  G      A C C
CA431918.1  617                                .........A....T.........................................................................................................  495
                                                  \        \ \
                                                  |        | |
                                                  G        C C
BU070293.1  616                                .........A....T.........................................................................................................  494
                                                    \      \ \
                                                    |      | |
                                                    T      C C
AI588866.1  599                                   ...........T.........................................................................................................  480
                                                         \ \ \
                                                         | | |
                                                         A C C
AI554314.1  599                                .........A....T.........................................................................................................  477
                                                  \        \ \
                                                  |        | |
                                                  G        C C
AI336170.1  604                                .........A....T.........................................................................................................  482
                                                  \        \ \
                                                  |        | |
                                                  G        C C
CT005089.1  94                                              .........................................................................................................  198
CR999820.1  52                                              .........................................................................................................  156
CR999128.1  166                                             .........................................................................................................  270

Query       451  GTTGATATGGTTCCTGGCAAGCCCATGTGTGTTGAGAGCTTCTCAGACTATCCACCTTTGGGTCGCTTTGCTGTTCGTGATATGAGACAGACAGTTGCGGTGGGTGTCATCAAAGCAGTGGACAAGAAGGCTGCTGGAGCTGGCAAGGTC  600
DC635691.1  468  .......................T..............................................................G...............................................................  617
```

```
AI679280.1  480  ......................................................................................................................................................  331
CA431918.1  494  ......................................................................................................................................................  345
BU070293.1  493  ......................................................................................................................................................  344
AI588866.1  479  ......................................................................................................................................................  330
AI554314.1  476  ......................................................................................................................................................  327
AI336170.1  481  ......................................................................................................................................................  332
CT005089.1  199  ......................................................................................................................................................  348
CR999820.1  157  ......................................................................................................................................................  306
CR999128.1  271  ......................................................................................................................................................  420

Query       601  ACCAAGTCTGCCCAGAAAGCTCAGAAGGCTAAATGAATATTATCCCTAATACCTGCCACCCCACTCTTAATCAGTGGTGGAAGAACGGTCTCAGAACTGTTTGTTTCAATTGGCCATTTAAGTTTAGTAGTAAAAGACTGGTTAATGATA  750
DC635691.1  618  ......................................................................................................................................................  767
AI679280.1  330  ......................................................................................................................................................  181
CA431918.1  344  ......................................................................................................................................................  195
BU070293.1  343  ......................................................................................................................................................  194
AI588866.1  329  ......................................................................................................................................................  180
AI554314.1  326  ......................................................................................................................................................  177
AI336170.1  331  ......................................................................................................................................................  182
CT005089.1  349  ......................................................................................................................................................  498
CR999820.1  307  ......................................................................................................................................................  456
CR999128.1  421  ......................................................................................................................................................  570

Query       751  ACAATGCATCGTAAAACCTTCAGAAGGAAAGGAGAATgttttgtggaccactttggttttctttttttgcgtgtggcagttttaagttattagtttttaaaatcagtactttttaATGGAAACAACTTGACCAAAAATTTGTCACAGAATT  900
DC635691.1  768  .........................-.....................................................-.................--.....................................-...........-..  911
AI679280.1  180  ......................................................................................................................................................  31
CA431918.1  194  ......................................................................................................................................................  45
BU070293.1  193  ......................................................................................................................................................  44
AI588866.1  179  ......................................................................................................................................................  30
AI554314.1  176  ......................................................................................................................................................  27
AI336170.1  181  ......................................................................................................................................................  32
CT005089.1  499  ......................................................................................................................................................  648
CR999820.1  457  ......................................................................................................................................................  606
CR999128.1  571  ......................................................................................................................................................  720

Query       901  TTGAGACCCATTAAAAAAGTTAAATG  926
DC635691.1  912  .-.........-.....-...       929
AI679280.1  30   ..........................  5
CA431918.1  44   ..........................  19
BU070293.1  43   ..........................  18
AI588866.1  29   ..........................  4
AI554314.1  26   ..........................  1
AI336170.1  31   ..........................  6
CT005089.1  649  ..........................  674
CR999820.1  607  ..........................  632
CR999128.1  721  ..........................  746
```

Query= N4-1 [organism=Homo sapiens] eef1a1

Length=1918

Sequences producing significant alignments:	Score (Bits)	E Value	Max ident
DC527884.1 DC527884 chimpanzee skin cDNA library PstA Pan trog...	3184	0.0	100%
BY794942.2 BY794942 Homo sapiens eye Homo sapiens cDNA clone H...	3166	0.0	100%
CF111220.1 Shultzomica04471 Rat lung airway and parenchyma cDN...	2357	0.0	91%
BX426149.2 BX426149 Homo sapiens NEUROBLASTOMA Homo sapiens cD...	2146	0.0	99%
CO001006.1 OC028 pre-oestrum adult sheep ovary library Ovis ar...	2047	0.0	93%
BX418564.2 BX418564 Homo sapiens FETAL BRAIN Homo sapiens cDNA...	2047	0.0	97%
BX426150.2 BX426150 Homo sapiens NEUROBLASTOMA Homo sapiens cD...	2037	0.0	99%
AL567209.3 AL567209 Homo sapiens FETAL BRAIN Homo sapiens cDNA...	2001	0.0	98%
BX440470.1 BX440470 Homo sapiens FETAL BRAIN Homo sapiens cDNA...	1999	0.0	98%
BU902062.1 AGENCOURT_10127553 NIH_MGC_71 Homo sapiens cDNA clo...	1999	0.0	98%

ALIGNMENTS

```
Query       180   TTCTTTTTCGCAACGGGTTTGCCGCCAGAACACAGGTGTCGTGAAAACTACCCCTAAAAGCCAAAATGGGAAAGGAAAAGACTCATATCAACATTGTCGTCATTGGACACGTAGATTCGGGCAAGTCCACCACTACTGGCCATCTGATCT  329
DC527884.1  6     ..T...................................................................................................................................................  155
BY794942.2  25                          ..................................................................................................................................  152
CF111220.1  7                 .........-..T......G.......T......G.C...G........A......................C........C.....A................C..............A..C.....C.......  148
                                 \                                        \
                                 |                                        |
                                 A                                       ATTC
BX426150.2  1                                .T.......................................................................................................................  123
BU902062.1  1                     .....................................................................................................................................  134

Query       330   ATAAATGCGGTGGCATCGACAAAAGAACCATTGAAAAATTTGAGAAGGAGGCTGCTGAGATGGGAAAGGGCTCCTTCAAGTATGCCTGGGTCTTGGATAAACTGAAAGCTGAGCGTGAACGTGGTATCACCATTGATATCTCCTTGTGGA  479
DC527884.1  156   ...............................................................................................................................T......................  305
BY794942.2  153   ......................................................................................................................................................  302
CF111220.1  149   .C.....T.....A......................G................G..........A.......................C...C...................G..........T.....C......C.....  298
BX426150.2  124   ......................................................................................................................................................  273
```

```
BU902062.1  135    ......................................................................................................................................................  284

Query       480    AATTTGAGACCAGCAAGTACTATGTGACTATCATTGATGCCCCAGGACACAGAGACTTTATCAAAAACATGATTACAGGGACATCTCAGGCTGACTGTGCTGTCCTGATTGTTGCTGCTGGTGTTGGTGAATTTGAAGCTGGTATCTCCA  629
DC527884.1  306    ..........................................................C...........................................................................................  455
BY794942.2  303    ......................................................................................................................................................  452
CF111220.1  299    .G........................................................C.....G...........G..C.................C.............................G......................  448
CO001006.1  1464        ....................T..C.............................C....................C.....C.....................................................C..........  1320
BX426150.2  274    ......................................................................................................................................................  423
BU902062.1  285    ......................................................................................................................................................  434

Query       630    AGAATGGGCAGACCCGAGAGCATGCCCTTCTGGCTTACACACTGGGTGTGAAACAACTAATTGTCGGTGTTAACAAAATGGATTCCACTGAGCCACCCTACAGCCAGAAGAGATATGAGGAAATTGTTAAGGAAGTCAGCACTTACATTA  779
DC527884.1  456    ......................................................................................................................................................  605
BY794942.2  453    ......................................................................................................................................................  602
CF111220.1  449    ....C...........T........T..............TT.............G........T.....C.................C........A.....T...........C........C.................C.......  598
BX426149.2  1196                                                                              ......-..A....-.A...............G..........................................  1124
CO001006.1  1319   ....C...........T.......................C.......................T..C.................T..............T..............C..........................C.......  1170
BX418564.2  1196                                                                         ....-....-......-...-...-...YH.VS..-.R......-W.-..G..W....-.R......M.....K....-.  1127
BX426150.2  424    ......................................................................................................................................................  573
AL567209.3  1145                                                                                                  .......S.....-...W.-..........-K.W........R.....-....A.  1094
                                                                                                                                                                     \
                                                                                                                                                                     |
                                                                                                                                                                     M
BX440470.1  1128                                                                                                                                                          ..  1127
BU902062.1  435    ......................................................................................................................................................  584

Query       780    AGAAAATTGGCTACAACCCCGACACAGTAGCATTTGTGCCAATTTCTGGTTGGAATGGTGACAACATGCTGGAGCCAAGTGCTAACATGCCTTGGTTCAAGGGATGGAAAGTCACCCGTAAGGATGGCAATGCCAGTGGAACCACGCTGC  929
DC527884.1  606    ......................................................................................................................................................  755
BY794942.2  603    ......................................................................................................................................................  752
CF111220.1  599    ...................T.................................................................T.....G..........................C..A.......G.........C..........  748
BX426149.2  1123   ......................................................................................................................................................  974
CO001006.1  1169   .................................................C...................................T.....A................................C....................C....  1020
BX418564.2  1126   R.-......S....-..........-R.....-.................-.....-..........................C......................-...........................................  984
BX426150.2  574    ......................................................................................................................................................  723
AL567209.3  1093   M.C............C......................................................................................................................................  944
BX440470.1  1126   ..-.....-..-W.-.............-...-....-S.M...Y..-.K......-..............-.....................K........................................................  986
                                         \
                                         |
                                         C
BU902062.1  585    ......................................................................................................................................................  734

Query       930    TTGAGGCTCTGGACTGCATCCTACCACCAACTCGTCCAACTGACAAGCCCTTGCGCCTGCCTCTCCAGGATGTCTACAAAATTGGTGGTATTGGTACTGTTCCTGTTGGCCGAGTGGAGACTGGTGTTCTCAAACCCGGTATGGTGGTCA  1079
DC527884.1  756    ......................................................................................................................................................  905
BY794942.2  753    ......................................................................................................................................................  902
CF111220.1  749    .G..A...T..........T..G..G......................TC.....A.....C..............T........C..C.....C.....C.....G...........A.................T...........T.  898
BX426149.2  973    ......................................................................................................................................................  824
CO001006.1  1019   ....A........T........G...........C...........A.........TT..................T.......................A.....G..T..T.......................T..C..........  870
BX418564.2  983    ..................................................C...................................................................................................  834
BX426150.2  724    ......................................................................................................................................................  873
AL567209.3  943    ...........K..........................................................................................................................................  794
BX440470.1  985    ...........R..........................................................................................................................................  836
BU902062.1  735    .....................................................................................................................................C..G.............  884

Query       1080   CCTTTGCTCCAGTCAACGTTACAACGGAAGTAAAATCTGTCGAAATGCACCATGAAGCTTTGAGTGAAGCTCTTCCTGGGGACAATGTGGGCTTCAATGTCAAGAATGTGTCTGTCAAGGATGTTCGTCGTGGCAACGTTGCTGGTGACA  1229
DC527884.1  906    ......................................................................................................................................................  1055
BY794942.2  903    ......................................................................................................................................................  1052
CF111220.1  899    ................T..A.....T.....C..G.....G................................G..............A........C..A.....C...........A..C...A.A........T........G....  1048
BX426149.2  823    ......................................................................................................................................................  674
CO001006.1  869    .......C........T..A.....T..G..G..G..C..A.............................C...................................C...........A.....C...........T..G..........  720
BX418564.2  833    ..................................................................................................................C...................................  684
BX426150.2  874    ......................................................................................................................................-...............  1023
                                                                                                 \
                                                                                                 |
                                                                                                 G
AL567209.3  793    ......................................................................................................................................................  644
BX440470.1  835    ......................................................................................................................................................  686
BU902062.1  885    ...............................-..................................................AC...........C...C..........................C..C....................  1034
                                                                                                                                                      \
                                                                                                                                                      |
                                                                                                                                                      G

Query       1230   GCAAAAATGACCCACCAATGGAAGCAGCTGGCTTCACTGCTCAGGTGATTATCCTGAACCATCCAGGCCAAATAAGCGCCGGCTATGCCCCTGTATTGGATTGCCACACGGCTCACATTGCATGCAAGTTTGCTGAGCTGAAGGAAAAGA  1379
DC527884.1  1056   ......................................................................................................................................................  1205
BY794942.2  1053   ......................................................................................................................................................  1203
                                                                                 \
                                                                                 |
                                                                                 G
CF111220.1  1049   ......................................................................G..C..T..T..............TC....C...........C.....A..............C.....T..A..G....  1198
BX426149.2  673    ......................................................................................................................................................  524
CO001006.1  719    ..........T.....C........T......C....A..............T....................C..TG....A.....A.....GC.......T.....A...........T.......................G....  570
BX418564.2  683    ...................................................................................................................................................M..  534
BX426150.2  1024   -...........-.........-.........-...................-..................-.......-.......-................-......T.-.............                         1140
AL567209.3  643    ......-...............................................................................................................................................  495
```

```
BX440470.1  685   ......................................................................................................................................................  535
                                                                                                                                                                       \
                                                                                                                                                                       |
                                                                                                                                                                       C
BU902062.1  1035  ..........................C.G......-.........-.........................T....-.....G.......-..A...........C..G..T...-..............                      1163
                                                          \                          \                \              \
                                                          |                          |                |              |
                                                          C                          G                T              A

Query       1380  TTGATCGCCGTTCTGGTAAAAAGCTGGAAGATGGCCCTAAATTCTTGAAGTCTGGTGATGCTGCCATTGTTGATATGGTTCCTGGCAAGCCCATGTGTGTTGAGAGCTTCTCAGACTATCCACCTTTGGGTCGCTTTGCTGTTCGTGATA  1529
DC527884.1  1206  ......................................................................................................................................................  1355
BY794942.2  1204  ......................................................................................................................................................  1353
CF111220.1  1199  .C.....T...........G.................C...................................C.....C.......................A........T.....C..T..AC.T.....T..............C.  1348
BX426149.2  523   ......................................................................................................................................................  374
CO001006.1  569   .......T........G................................A........C........C.......................T........C........T..T..T.....T..C.....C..T........G.....C.  420
BX418564.2  533   ......................................................................................................................................................  384
AL567209.3  494   ...............................................................................K......................................................................  345
BX440470.1  534   ......................................................................................................................................................  385

Query       1530  TGAGACAGACAGTTGCGGTGGGTGTCATCAAAGCAGTGGACAAGAAGGCTGCTGGAGCTGGCAAGGTCACCAAGTCTGCCCAGAAAGCTCAGAAGGCTAAATGAATATTATCCCTAATACCTGCCACCCCACTCTTAATCAGTGGTGGAA  1679
DC527884.1  1356  .......................A...............................................................................................................C..............  1505
BY794942.2  1354  ......................................................................................................................................................  1503
CF111220.1  1349  ....G...........T.................C.................A...........A....................................................C.............G..................  1498
BX426149.2  373   ......................................................................................................................................................  224
CO001006.1  419   .............C..T................................A................................................................C................G..................  270
BX418564.2  383   ......................................................................................................................................................  234
AL567209.3  344   ..........................................................................................M......Y....................................................  194
                                                                                                                                                                  \
                                                                                                                                                                  |
                                                                                                                                                                  A
BX440470.1  384   ......................................................................................................................................................  235

Query       1680  GAACGGTCTCAGAACTGTTTGTTTCAATTGGCCATTTAAGTTTAGTAGTAAAAGACTGGTTAATGATAACAATGCATCGTAAAACCTTCAGAAGGAAAGGAGAATgttttgtggaccactttggttttctttttttgcgtgtggcagtttt  1829
DC527884.1  1506  ......................................................................................................................................................  1655
BY794942.2  1504  ......................................................................................................................................................  1653
CF111220.1  1499  ......................C.....................A....................................................---..................-..--...-..G.----...............  1637
BX426149.2  223   ......................................................................................................................................................  74
CO001006.1  269   ......................C.....................A.........................................................................-.A..-....G.-----.--........-...  129
                                                                                                                                \
                                                                                                                                |
                                                                                                                                T
BX418564.2  233   ......................................................................................................................................................  84
AL567209.3  193   ......................................................................................................................................................  43
                                                                                                                                      \
                                                                                                                                      |
                                                                                                                                      G
BX440470.1  234   ......................................................................................................................................................  85

Query       1830  aagttattagtttttaaaatcagtactttttaATGGAAACAACTTGACCAAAAATTTGTCACAGAATTTTGAGACCCATTAAAAAAGTT  1918
DC527884.1  1656  .........................................................................................  1744
BY794942.2  1654  .........................................................................................  1742
CF111220.1  1638  ..............C........................................C....................-............  1726
                                                                                                      \
                                                                                                      |
                                                                                                      C
BX426149.2  73    ...................................-.......-.....G......G.-..G...........T..                1
CO001006.1  128   ...................................A...................C.............-...................  40
                                                                                                        \
                                                                                                        |
                                                                                                        A
BX418564.2  83    .....................................................................M-.............       1
AL567209.3  42    ..............................-....-..-...-...                                             1
BX440470.1  84    ....................................................................................       1
```

Query= N5-2 [organism=Homo sapiens] eef1a1

Length=1718

```
                                                                   Score        E     Max
Sequences producing significant alignments:                       (Bits)     Value  ident

DC527884.1 DC527884 chimpanzee skin cDNA library PstA Pan trog...  3011       0.0    98%
BY794942.2 BY794942 Homo sapiens eye Homo sapiens cDNA clone H...  2992       0.0    98%
CF111220.1 Shultzomica04471 Rat lung airway and parenchyma cDN...  2206       0.0    90%
BX426149.2 BX426149 Homo sapiens NEUROBLASTOMA Homo sapiens cD...  2146       0.0    99%
BX418564.2 BX418564 Homo sapiens FETAL BRAIN Homo sapiens cDNA...  2047       0.0    97%
AL567209.3 AL567209 Homo sapiens FETAL BRAIN Homo sapiens cDNA...  2001       0.0    98%
BX440470.1 BX440470 Homo sapiens FETAL BRAIN Homo sapiens cDNA...  1999       0.0    98%
BX420340.1 BX420340 Homo sapiens FETAL BRAIN Homo sapiens cDNA...  1997       0.0    99%
AL518238.2 AL518238 Homo sapiens NEUROBLASTOMA Homo sapiens cD...  1993       0.0    98%
BX459625.2 BX459625 Homo sapiens FETAL BRAIN Homo sapiens cDNA...  1993       0.0    99%
```

ALIGNMENTS

```
Query       2     TTTTTTCGCAACGGGTTTGCCGCCAGAACACAGGTGTCGTGAAAAACTACCCCTAAAAAGCCAAAATGGGAAAGGAAAAGACTCATATCAACATTGTCGTCATTGGACACGTAGATTCGGGCAAGTCCACCACTACTGGCCATCTGATCT  151
DC527884.1  8     .............................................-........-...............................................................................................  155
BY794942.2  25                        .........................-........-...............................................................................................  152
CF111220.1  7               .........-..T......G.......T......-G.C...G.........A.......................C........C.....A.................C..............A..C.....C.......  148
                               \                                          \
                               |                                          |
                               A                                        TTC

Query       152   ATAAATGCGGTGGCATCGACAAAAGAACCATTGAAAAATTTGAGAAGGAGGCTGCTGAGATGGGAAAGGGCTCCTTCAAGTATGCCTGGGTCTGGATAAACTGAAAGCTGAGCGTGAACGTGTATCACATTGATATCTCCTTGTGGAAAT  301
DC527884.1  156   .............................................................................................................................T........................  308
                                                                                                              \                             \    \
                                                                                                              |                             |    |
                                                                                                              T                             G    C
BY794942.2  153   ......................................................................................................................................................  305
                                                                                                              \                             \    \
                                                                                                              |                             |    |
                                                                                                              T                             G    C
CF111220.1  149   .C.....T.....A.......................G.................G...........A............................C....................G...............C......C.......G.  301
                                                                                                             \                              \     \
                                                                                                             |                              |     |
                                                                                                             C                              G     T

Query       302   TTGAGACCAGCAAGTACTATGTGACTATCATTGATGCCCAGGAACAGAGACTTTATCAAAAACATGATACAGGGACATCTCAGCTGACTGTGCTGTCCTGATTGTGCTGTGTTGTGAATTTGAGTGGTCCAAGAACCCGAGAGCATGCCC  451
DC527884.1  309   .....................................................C................................................................................................  482
                                                         \   \                       \              \                      \  \    \        \ \  \  \ \\\
                                                         |   |                       |              |                      |  |    |        | |  |  | |||
                                                         C   C                       T              G                   TGCT  G    G        A C TATC| TGG
                                                                                                                                                    |  ||
                                                                                                                                                  AAG  C|
                                                                                                                                                        |
                                                                                                                                                        G
BY794942.2  306   ......................................................................................................................................................  479
                                                         \   \                       \              \                      \  \    \        \ \  \  \ \\\
                                                         |   |                       |              |                      |  |    |        | |  |  | |||
                                                         C   C                       T              G                   TGCT  G    G        A C TATC| TGG
                                                                                                                                                    |  ||
                                                                                                                                                  AAG  C|
                                                                                                                                                        |
                                                                                                                                                        G
CF111220.1  302   .....................................................C.....G..........G..C................C.......................G........................T........T.  475
                                                         \   \                        \             \                      \  \    \        \ \  \  \ \\\
                                                         |   |                        |             |                      |  |    |        | |  |  | |||
                                                         C   C                        T             G                   TGCT  G    G        A C TATC| CGG
                                                                                                                                                    |  ||
                                                                                                                                                  AAG  C|
                                                                                                                                                        |
                                                                                                                                                        G

Query       452   TTCTGGCTTACACACTGGGTGTGAAACAACTAATTGTCGGTGTTAACAAAATGGATTCCACTGAGCCACCCTACAGCCAGAAGAGATATGAGGAAATTGTTAAGGAAGTCAGCACTTACATTAAGAAAATTGGCTACAACCCCGACACAG  601
DC527884.1  483   ......................................................................................................................................................  632
BY794942.2  480   ......................................................................................................................................................  629
CF111220.1  476   ............TT..............G........T.....C.................C........A.....T...........C........C.................C..........................T.......  625
BX426149.2  1196                                                  ......-..A....-.A...............G.....................................................................  1097
BX418564.2  1196                                             ....-....-......-...-...-...YH.VS..-.R......-W.-..G..W....-.R......M.....K....-.R.-......S....-..........-R  1103
AL567209.3  1145                                                                      .......S.....-...W.-..........-K.W........R.....-....A.M.C............C...........  1067
                                                                                                                                                     \
                                                                                                                                                     |
                                                                                                                                                     M
BX440470.1  1128                                                                                                                       ....-.....-..-W.-............  1103
                                                                                                                                                               \
                                                                                                                                                               |
                                                                                                                                                               C
BX420340.1  1117                                                                                                                                  ...M..M...........  1100
BX418238.2  1148                                                                                                                 .....R..-....-.M.....-..........-..W...M....V.M....  1102
BX459625.2  1118                                                                                                                            ........C.MCM....C.....K  1095

Query       602   TAGCATTTGTGCCAATTTCTGGTTGGAATGGTGACAACATGCTGGAGCCAAGTGCTAACATGCCTTGGTTCAAGGGATGGAAAGTCACCCGTAAGGATGGCAATGCCAGTGGAACCACGCTGCTTGAGGCTCTGGACTGCATCCTACCAC  751
DC527884.1  633   ......................................................................................................................................................  782
BY794942.2  630   ......................................................................................................................................................  779
CF111220.1  626   ..........................................................T.....G..........................C..A.......G.........C...........G..A...T..........T..G..G.  775
BX426149.2  1096  ......................................................................................................................................................  947
BX418564.2  1102  .....-.................-.....-..........................C......................-......................................................................  957
AL567209.3  1066  ......................................................................................................................................K...............  917
BX440470.1  1102  .-...-....-S.M...Y..-.K......-..............-.....................K...................................................................R...............  959
BX420340.1  1099  ........K.-..M.W...YT...D....K.-...........T..........Y...............................................................................A...............  952
AL518238.2  1101  .....................-K.........A...........A....................................W.............................R......................................  952
                                                                                                                \
                                                                                                                |
                                                                                                                A
```

```
BX459625.2  1094  ....W...-KS.......-.K......W.......................................K.A................................................................................  946
                                                     \
                                                     |
                                                     T

Query       752   CAACTCGTCCAACTGACAAGCCCTTGCGCCTGCCTCTCCAGGATGTCTACAAAATTGGTGGTATTGGTACTGTTCCTGTTGGCCGAGTGGAGACTGGTGTTCTCAAACCCGGTATGGTGGTCACCTTTGCTCCAGTCAACGTTACAACGG  901
DC527884.1  783   ......................................................................................................................................................  932
BY794942.2  780   ......................................................................................................................................................  929
CF111220.1  776   ......................TC....A.....C..............T........C..C.....C.....C.....G...........A.................T...........T................T..A......T.  925
BX426149.2  946   ......................................................................................................................................................  797
BX418564.2  956   .......................C..............................................................................................................................  807
AL567209.3  916   ......................................................................................................................................................  767
BX440470.1  958   ......................................................................................................................................................  809
BX420340.1  951   ..............R.......................................................................................................................................  802
AL518238.2  951   ...........................................................................................W..................................B.......................  802
BX459625.2  945   ......................................................................................................................................................  796

Query       902   AAGTAAAATCTGTCGAAATGCACCATGAAGCTTTGAGTGAAGCTCTTCCTGGGGACAATGTGGGCTTCAATGTCAAGAATGTGTCTGTCAAGGATGTTCGTCGTGGCAACGTTGCTGGTGACAGCAAAAATGACCCACCAATGGAAGCAG  1051
DC527884.1  933   ......................................................................................................................................................  1082
BY794942.2  930   ......................................................................................................................................................  1079
CF111220.1  926   ....C..G.....G................................G..............A........C..A.....C...........A..C...A.A........T........G...............................  1075
BX426149.2  796   ......................................................................................................................................................  647
BX418564.2  806   .......................................................................................C..............................................................  657
AL567209.3  766   ................................................................................................................................-.....................  618
BX440470.1  808   ......................................................................................................................................................  659
BX420340.1  801   ......................................................................................................................................................  652
AL518238.2  801   .......W...............................................................................................................A............................G.  652
BX459625.2  795   ......................................................................................................................................................  646

Query       1052  CTGGCTTCACTGCTCAGGTGATTATCCTGAACCATCCAGGCCAAATAAGCGCCGGCTATGCCCCTGTATTGGATTGCCACACGGCTCACATTGCATGCAAGTTTGCTGAGCTGAAGGAAAAGATTGATCGCCGTTCTGGTAAAAAGCTGG  1201
DC527884.1  1083  ......................................................................................................................................................  1232
BY794942.2  1080  ......................................................................................................................................................  1230
                                                     \
                                                     |
                                                     G

CF111220.1  1076  ...........................................G..C..T..T..............TC....C...........C.....A..............C.....T..A..G.....C.....T..........G........  1225
BX426149.2  646   ......................................................................................................................................................  497
BX418564.2  656   .......................................................................................................................M..............................  507
AL567209.3  617   ......................................................................................................................................................  468
BX440470.1  658   ......................................................................................................................................................  508
                                                                                                                                            \
                                                                                                                                            |
                                                                                                                                            C

BX420340.1  651   ......................................................................................................................................................  502
AL518238.2  651   ...........................................................................................Y..........................................................  502
BX459625.2  645   ......................................................................................................................................................  496

Query       1202  AAGATGGCCCTAAATTCTTGAAGTCTGGTGATGCTGCCATTGTTGATATGGTTCCTGGCAAGCCCATGTGTGTTGAGAGCTTCTCAGACTATCCACCTTTGGGTCGCTTTGCTGTTCGTGATATGAGACAGACAGTTGCGGTGGGTGTCA  1351
DC527884.1  1233  ..................................................................................................................................................A...  1382
BY794942.2  1231  ......................................................................................................................................................  1380
CF111220.1  1226  ..........C...................................C.....C.......................A........T.....C..T..AC.T.....T..............C....G...........T...........  1375
BX426149.2  496   ......................................................................................................................................................  347
BX418564.2  506   ......................................................................................................................................................  357
AL567209.3  467   ....................................................K.................................................................................................  318
BX440470.1  507   ......................................................................................................................................................  358
BX420340.1  501   ......................................................................................................................................................  352
AL518238.2  501   ......................................................................................................................................................  352
BX459625.2  495   ......................................................................................................................................................  346

Query       1352  TCAAAGCAGTGGACAAGAAGGCTGCTGGAGCTGGCAAGGTCACCAAGTCTGCCCAGAAAGCTCAGAAGGCTAAATGAATATTATCCCTAATACCTGCCACCCCACTCTTAATCAGTGGTGGAAGAACGGTCTCAGAACTGTTTGTTTCAA  1501
DC527884.1  1383  ............................................................................................................C.........................................  1532
BY794942.2  1381  ......................................................................................................................................................  1530
CF111220.1  1376  .......C.................A...........A....................................................C.............G........................................C....  1525
BX426149.2  346   ......................................................................................................................................................  197
BX418564.2  356   ......................................................................................................................................................  207
AL567209.3  317   ...............................................................M......Y...............................................................................  167
                                                                                                                                       \
                                                                                                                                       |
                                                                                                                                       A

BX440470.1  357   ......................................................................................................................................................  208
BX420340.1  351   ......................................................................................................................................................  202
AL518238.2  351   ............................G.....................................................................................................G...............  202
BX459625.2  345   ......................................................................................................................................................  196

Query       1502  TTGGCCATTTAAGTTTAGTAGTAAAAGACTGGTTAATGATAACAATGCATCGTAAAACCTTCAGAAGGAAAGGAGAATgttttgtggaccactttggttttctttttttgcgtgtggcagttttaagttattagtttttaaaatcagtact  1651
DC527884.1  1533  ......................................................................................................................................................  1682
BY794942.2  1531  ......................................................................................................................................................  1680
CF111220.1  1526  .................A....................................................---..................-...--....-..G.----...........................C............  1664
BX426149.2  196   ......................................................................................................................................................  47
BX418564.2  206   ......................................................................................................................................................  57
AL567209.3  166   ......................................................................................................................................................  16
                                                                                                           \
```

```
                                                                                                           |
                                                                                                           G
BX440470.1  207   ......................................................................................................................................................  58
BX420340.1  201   ......................................................................................................................................................  52
AL518238.2  201   ......................................................................................................................................................  52
BX459625.2  195   ......................................................................................................................................................  46

Query       1652  ttttaATGGAAACAACTTGACCAAAAATTTGTCACAGAATTTTGAGACCCATTAAAAAAGTTAAATG  1718
DC527884.1  1683  ...................................................................  1749
BY794942.2  1681  ...................................................................  1747
CF111220.1  1665  ............................C....................-............T....  1731
                                                                           \
                                                                           |
                                                                           C

BX426149.2  46    ........-.......-.....G......G.-..G...........T..                    1
BX418564.2  56    ..........................................M-.............             1
AL567209.3  15    ...-....-..-...-...                                                  1
BX440470.1  57    .........................................................            1
BX420340.1  51    .............................................R.....                  1
AL518238.2  51    .............N.............N......................                    1
                                                   \
                                                   |
                                                   N
BX459625.2  45    .............................................                        1

Query= N6-15 [organism=Homo sapiens] eef1a1

Length=1144


                                                                   Score        E     Max
Sequences producing significant alignments:                       (Bits)     Value  ident

CR996534.1 CR996534 RZPD no.9017 Homo sapiens cDNA clone RZPDp...  1437       0.0    100%
CR982215.1 CR982215 RZPD no.9016 Homo sapiens cDNA clone RZPDp...  1437       0.0    100%
BX453401.2 BX453401 Homo sapiens T CELLS (JURKAT CELL LINE) Ho...  1437       0.0    100%
BX443032.2 BX443032 Homo sapiens B CELLS (RAMOS CELL LINE) Hom...  1437       0.0    100%
BX421307.2 BX421307 Homo sapiens B CELLS (RAMOS CELL LINE) Hom...  1437       0.0    100%
BX353545.2 BX353545 Homo sapiens NEUROBLASTOMA COT 25-NORMALIZ...  1437       0.0    100%
CX167214.1 HESC2_49_G11.g1_A035 NIH_MGC_258 Homo sapiens cDNA ...  1437       0.0    100%
CA771972.1 io93g08.x1 HR85 islet Homo sapiens cDNA clone IMAGE...  1437       0.0    100%
BU845975.1 AGENCOURT_10413042 NIH_MGC_109 Homo sapiens cDNA cl...  1437       0.0    100%
BU844361.1 AGENCOURT_10414634 NIH_MGC_109 Homo sapiens cDNA cl...  1437       0.0    100%

ALIGNMENTS

Query       357   CTTTAGAGTGAAGCTCTTCCTGGGGACAATGTGGGCTTCAATGTCAAGAATGTGTCTGTCAAGGATGTTCGTCGTGGCAACGTTGCTGGTGACAGCAAAAATGACCCACCAATGGAAGCAGCTGGCTTCACTGCTCAGGTGATTATCCTG  506
CR996534.1  62    ....-.............................................................................................................................................  210
CR982215.1  5     ....-.............................................................................................................................................  153
BX453401.2  16    ....-.............................................................................................................................................  164
BX443032.2  142   ....-.............................................................................................................................................  290
BX421307.2  116   ....-.............................................................................................................................................  264
BX353545.2  21    ....-.............................................................................................................................................  169
CX167214.1  789   ....-.............................................................................................................................................  641
CA771972.1  789   ....-.............................................................................................................................................  641
BU845975.1  787   ....-.............................................................................................................................................  639
BU844361.1  796   ....-.............................................................................................................................................  648

Query       507   AACCATCCAGGCCAAATAAGCGCCGGCTATGCCCTGTATTGGATTGCCACACGGCTCACATTGCATGCAAGTTTGCTGAGCTGAAGGAAAAGATTGATCGCCGTTCTGGTAAAAAGCTGGAAGATGGCCCTAAATTCTTGAAGTCTGGTG  656
CR996534.1  211   ......................................................................................................................................................  361
                                                 \
                                                 |
                                                 C
CR982215.1  154   ......................................................................................................................................................  304
                                                 \
                                                 |
                                                 C
BX453401.2  165   ......................................................................................................................................................  315
                                                 \
                                                 |
                                                 C
BX443032.2  291   ......................................................................................................................................................  441
                                                 \
                                                 |
                                                 C
BX421307.2  265   ......................................................................................................................................................  415
                                                 \
                                                 |
                                                 C
BX353545.2  170   ......................................................................................................................................................  320
                                                 \
                                                 |
```

```
                                                 C
CX167214.1  640   ......................................................................................................................................................  490
                                                    \
                                                    |
                                                    C
CA771972.1  640   ......................................................................................................................................................  490
                                                    \
                                                    |
                                                    C
BU845975.1  638   ......................................................................................................................................................  488
                                                    \
                                                    |
                                                    C
BU844361.1  647   ......................................................................................................................................................  497
                                                    \
                                                    |
                                                    C

Query       657   ATGCTGCCATTGTTGATATGGTTCCTGGCAAGCCCATGTGTGTTGAGAGCTTCTCAGACTATCCACCTTTGGGTCGCTTTGCTGTTCGTGATATGAGACAGACAGTTGCGGTGGGTGTCATCAAAGCAGTGGACAAGAAGGCTGCTGGAG  806
CR996534.1  362   ......................................................................................................................................................  511
CR982215.1  305   ......................................................................................................................................................  454
BX453401.2  316   ......................................................................................................................................................  465
BX443032.2  442   ......................................................................................................................................................  591
BX421307.2  416   ......................................................................................................................................................  565
BX353545.2  321   ......................................................................................................................................................  470
CX167214.1  489   ......................................................................................................................................................  340
CA771972.1  489   ......................................................................................................................................................  340
BU845975.1  487   ......................................................................................................................................................  338
BU844361.1  496   ......................................................................................................................................................  347

Query       807   CTGGCAAGGTCACCAAGTCTGCCCAGAAAGCTCAGAAGGCTAAATGAATATTATCCCTAATACCTGCCACCCCACTCTTAATCAGTGGTGGAAGAACGGTCTCAGAACTGTTTGTTTCAATTGGCCATTTAAGTTTAGTAGTAAAAGACT  956
CR996534.1  512   ......................................................................................................................................................  661
CR982215.1  455   ......................................................................................................................................................  604
BX453401.2  466   ......................................................................................................................................................  615
BX443032.2  592   ......................................................................................................................................................  741
BX421307.2  566   ......................................................................................................................................................  715
BX353545.2  471   ......................................................................................................................................................  620
CX167214.1  339   ......................................................................................................................................................  190
CA771972.1  339   ......................................................................................................................................................  190
BU845975.1  337   ......................................................................................................................................................  188
BU844361.1  346   ......................................................................................................................................................  197

Query       957   GGTTAATGATAACAATGCATCGTAAAACCTTCAGAAGGAAAGGAGAATgttttgtggaccactttggttttcttttttgcgtgtggcagttttaagttattagtttttaaaatcagtactttttaATGGAAACAACTTGACCAAAAATTT  1106
CR996534.1  662   ......................................................................................................................................................  811
CR982215.1  605   ......................................................................................................................................................  754
BX453401.2  616   ......................................................................................................................................................  765
BX443032.2  742   ......................................................................................................................................................  891
BX421307.2  716   ......................................................................................................................................................  865
BX353545.2  621   ......................................................................................................................................................  770
CX167214.1  189   ......................................................................................................................................................  40
CA771972.1  189   ......................................................................................................................................................  40
BU845975.1  187   ......................................................................................................................................................  38
BU844361.1  196   ......................................................................................................................................................  47

Query       1107  GTCACCAGAATTTTGAGACCCATTAAAAAAGTTAAATG  1144
CR996534.1  812   ....-.................................  848
CR982215.1  755   ....-.................................  791
BX453401.2  766   ....-.................................  802
BX443032.2  892   ....-.................................  928
BX421307.2  866   ....-.................................  902
BX353545.2  771   ....-.................................  807
CX167214.1  39    .....-................................  3
CA771972.1  39    .....-................................  3
BU845975.1  37    .....-................................  1
BU844361.1  46    .....-................................  10

Query= N7-11 [organism=Homo sapiens] eef1a1

Length=972


                                                                  Score        E     Max
Sequences producing significant alignments:                      (Bits)     Value  ident

CT005089.1 CT005089 RZPD no.9017 Homo sapiens cDNA clone RZPDp...  1201       0.0    100%
CR999128.1 CR999128 RZPD no.9017 Homo sapiens cDNA clone RZPDp...  1201       0.0    100%
CR996969.1 CR996969 RZPD no.9017 Homo sapiens cDNA clone RZPDp...  1201       0.0    100%
CR996534.1 CR996534 RZPD no.9017 Homo sapiens cDNA clone RZPDp...  1201       0.0    100%
CR982215.1 CR982215 RZPD no.9016 Homo sapiens cDNA clone RZPDp...  1201       0.0    100%
CR977084.1 CR977084 RZPD no.9016 Homo sapiens cDNA clone RZPDp...  1201       0.0    100%
CR975903.1 CR975903 RZPD no.9016 Homo sapiens cDNA clone RZPDp...  1201       0.0    100%
BQ776023.1 UI-H-FH0-bck-l-16-0-UI.s1 NCI_CGAP_FH0 Homo sapiens...  1201       0.0    100%
```

```
BQ775888.1 UI-H-FH0-bcg-o-05-0-UI.s1 NCI_CGAP_FH0 Homo sapiens...  1201       0.0    100%
BQ775886.1 UI-H-FH0-bcg-o-01-0-UI.s1 NCI_CGAP_FH0 Homo sapiens...  1201       0.0    100%

ALIGNMENTS

Query       323  GTGATTATCCTGAACCATCCAGGCCAAATAAGCGCCGGCTATGCCCCTGTATTGGATTGCCACACGGCTCACATTGCATGCAAGTTTGCTGAGCTGAAGGAAAAGATTGATCGCCGTTCTGGTAAAAAGCTGGAAGATGGCCCTAAATTC  472
CT005089.1  25   ......................................................................................................................................................  174
CR999128.1  97   ......................................................................................................................................................  246
CR996969.1  130  ......................................................................................................................................................  279
CR996534.1  199  ......................................................................................................................................................  348
CR982215.1  142  ......................................................................................................................................................  291
CR977084.1  88   ......................................................................................................................................................  237
CR975903.1  19   ......................................................................................................................................................  168
BQ776023.1  668  ......................................................................................................................................................  519
BQ775888.1  668  ......................................................................................................................................................  519
BQ775886.1  668  ......................................................................................................................................................  519

Query       473  TTGAAGTCTGGTGATGCTGCCATTGTTGATATGGTTCCTGGCAAGCCCATGTGTGTTGAGAGCTTCTCAGACTATCCACCTTTGGGTCGCTTTGCTGTTCGTGATATGAGACAGACAGTTGCGGTGGGTGTCATCAAAGCAGTGGACAAG  622
CT005089.1  175  ......................................................................................................................................................  324
CR999128.1  247  ......................................................................................................................................................  396
CR996969.1  280  ......................................................................................................................................................  429
CR996534.1  349  ......................................................................................................................................................  498
CR982215.1  292  ......................................................................................................................................................  441
CR977084.1  238  ......................................................................................................................................................  387
CR975903.1  169  ......................................................................................................................................................  318
BQ776023.1  518  ......................................................................................................................................................  369
BQ775888.1  518  ......................................................................................................................................................  369
BQ775886.1  518  ......................................................................................................................................................  369

Query       623  AAGGCTGCTGGAGCTGGCAAGGTCACCAAGTCTGCCCAGAAAGCTCAGAAGGCTAAATGAATATTATCCCTAATACCTGCCACCCCACTCTTAATCAGTGGTGGAAGAACGGTCTCAGAACTGTTTGTTTCAATTGGCCATTTAAGTTTA  772
CT005089.1  325  ......................................................................................................................................................  474
CR999128.1  397  ......................................................................................................................................................  546
CR996969.1  430  ......................................................................................................................................................  579
CR996534.1  499  ......................................................................................................................................................  648
CR982215.1  442  ......................................................................................................................................................  591
CR977084.1  388  ......................................................................................................................................................  537
CR975903.1  319  ......................................................................................................................................................  468
BQ776023.1  368  ......................................................................................................................................................  219
BQ775888.1  368  ......................................................................................................................................................  219
BQ775886.1  368  ......................................................................................................................................................  219

Query       773  GTAGTAAAAGACTGGTTAATGATAACAATGCATCGTAAAACCTTCAGAAGGAAAGGAGAATgttttgtggaccactttggttttctttttttgcgtgtggcagttttaagttattagtttttaaaatcagtactttttaATGGAAACAACT  922
CT005089.1  475  ......................................................................................................................................................  624
CR999128.1  547  ......................................................................................................................................................  696
CR996969.1  580  ......................................................................................................................................................  729
CR996534.1  649  ......................................................................................................................................................  798
CR982215.1  592  ......................................................................................................................................................  741
CR977084.1  538  ......................................................................................................................................................  687
CR975903.1  469  ......................................................................................................................................................  618
BQ776023.1  218  ......................................................................................................................................................  69
BQ775888.1  218  ......................................................................................................................................................  69
BQ775886.1  218  ......................................................................................................................................................  69

Query       923  TGACCAAAAATTTGTCACAGAATTTTGAGACCCATTAAAAAAGTTAAATG  972
CT005089.1  625  ..................................................  674
CR999128.1  697  ..................................................  746
CR996969.1  730  ..................................................  779
CR996534.1  799  ..................................................  848
CR982215.1  742  ..................................................  791
CR977084.1  688  ..................................................  737
CR975903.1  619  ..................................................  668
BQ776023.1  68   ..................................................  19
BQ775888.1  68   ..................................................  19
BQ775886.1  68   ..................................................  19

Query= N8-C* [organism=Homo sapiens] eef1a1

Length=1830


                                                                  Score        E     Max
Sequences producing significant alignments:                       (Bits)     Value  ident

BY794942.2 BY794942 Homo sapiens eye Homo sapiens cDNA clone H...  3155       0.0    100%
DC527884.1 DC527884 chimpanzee skin cDNA library PstA Pan trog...  3138       0.0    100%
CF111220.1 Shultzomica04471 Rat lung airway and parenchyma cDN...  2344       0.0    91%
BX426149.2 BX426149 Homo sapiens NEUROBLASTOMA Homo sapiens cD...  2146       0.0    99%
CO001006.1 OC028 pre-oestrum adult sheep ovary library Ovis ar...  2050       0.0    93%
BX418564.2 BX418564 Homo sapiens FETAL BRAIN Homo sapiens cDNA...  2047       0.0    97%
BX426150.2 BX426150 Homo sapiens NEUROBLASTOMA Homo sapiens cD...  2032       0.0    99%
AL567209.3 AL567209 Homo sapiens FETAL BRAIN Homo sapiens cDNA...  2001       0.0    98%
BX440470.1 BX440470 Homo sapiens FETAL BRAIN Homo sapiens cDNA...  1999       0.0    98%
```

```
BX420340.1 BX420340 Homo sapiens FETAL BRAIN Homo sapiens cDNA...  1997       0.0    99%

ALIGNMENTS

Query       117   CGAAGGTGTCGTGAAAACTACCCCTAAAAGCCAAAATGGGAAAGGAAAAGACTCATATCAACATTGTCGTCATTGGACACGTAGATTCGGGCAAGTCCACCACTACTGGCCATCTGATCTATAAATGCGGTGGCATCGACAAAAGAACCA 266
BY794942.2  36       ................................................................................................................................................... 182
DC527884.1  39       ................................................................................................................................................... 185
CF111220.1  25    ..C......T......G.C...G........A.......................C........C.....A.................C..............A..C.....C........C.....T.....A................ 178
                                            \
                                            |
                                         ATTC
BX426150.2  7        ................................................................................................................................................... 153

Query       267   TTGAAAAATTTGAGAAGGAGGCTGCTGAGATGGGAAAGGGCTCCTTCAAGTATGCCTGGGTCTTGGATAAACTGAAAGCTGAGCGTGAACGTGGTATCACCATTGATATCTCCTTGTGGAAATTTGAGACCAGCAAGTACTATGTGACTA 416
BY794942.2  183   ...................................................................................................................................................... 332
DC527884.1  186   .................................................................................................T.................................................... 335
CF111220.1  179   .......G.................G...........A........................C....C....................G...........T.....C......C.......G............................ 328
CO001006.1  1464                                                                                                                               ....................T..C. 1440
BX426150.2  154   ...................................................................................................................................................... 303

Query       417   TCATTGATGCCCCAGGACACAGAGACTTTATCAAAAACATGATTACAGGGACATCTCAGGCTGACTGTGCTGTCCTGATTGTTGCTGCTGGTGTTGGTGAATTTGAAGCTGGTATCTCCAAGAATGGGCAGACCCGAGAGCATGCCCTTC 566
BY794942.2  333   ...................................................................................................................................................... 482
DC527884.1  336   ............................C......................................................................................................................... 485
CF111220.1  329   ............................C.....G...........G..C.................C.............................G..........................C...........T........T.... 478
CO001006.1  1439  ............................C....................C.....C.....................................................C..............C...........T............. 1290
BX426150.2  304   ...................................................................................................................................................... 453

Query       567   TGGCTTACACACTGGGTGTGAAACAACTAATTGTCGGTGTTAACAAAATGGATTCCACTGAGCCACCCTACAGCCAGAAGAGATATGAGGAAATTGTTAAGGAAGTCAGCACTTACATTAAGAAAATTGGCTACAACCCCGACACAGTAG 716
BY794942.2  483   ...................................................................................................................................................... 632
DC527884.1  486   ...................................................................................................................................................... 635
CF111220.1  479   ..........TT.............G........T.....C.................C........A.....T...........C........C.................C..........................T.......... 628
BX426149.2  1196                                                ......-..A....-.A...............G........................................................................ 1094
CO001006.1  1289  ..........C.......................T..C.................T..............T..............C..........................C..................................... 1140
BX418564.2  1196                                          ....-....-......-...-...-...YH.VS..-.R......-W.-..G..W....-.R......M.....K...-.R.-.......S...-...........-R... 1100
BX426150.2  454   ...................................................................................................................................................... 603
AL567209.3  1145                                                                   .......S.....-...W.-..........-K.W........R.....-....A.M.C............C.............. 1064
                                                                                                                                  \
                                                                                                                                  |
                                                                                                                                  M
BX440470.1  1128                                                                                                                        ....-.....-..-W.-.............-. 1101
                                                                                                                                                                \
                                                                                                                                                                |
                                                                                                                                                                C
BX420340.1  1117                                                                                                                                       ...M..M.............. 1097

Query       717   CATTTGTGCCAATTTCTGGTTGGAATGGTGACAACATGCTGGAGCCAAGTGCTAACATGCCTTGGTTCAAGGGATGGAAAGTCACCCGTAAGGATGGCAATGCCAGTGGAACCACGCTGCTTGAGGCTCTGGACTGCATCCTACCACCAA 866
BY794942.2  633   ...................................................................................................................................................... 782
DC527884.1  636   ...................................................................................................................................................... 785
CF111220.1  629   .......................................................T.....G..........................C..A.......G.........C...........G..A...T..........T..G..G.... 778
BX426149.2  1093  ...................................................................................................................................................... 944
CO001006.1  1139  ...................C...................................T.....A................................C....................C........A........T........G....... 990
BX418564.2  1099  ..-.................-.....-..........................C......................-......................................................................... 954
BX426150.2  604   ...................................................................................................................................................... 753
AL567209.3  1063  ...................................................................................................................................K.................. 914
BX440470.1  1100  ..-....-S.M..Y..-.K.......-..............-.....................K...................................................................R.................. 956
BX420340.1  1096  .....K.-..M.W...YT...D....K.-...........T..........Y...............................................................................A.................. 949

Query       867   CTCGTCCAACTGACAAGCCCTTGCGCCTGCCTCTCCAGGATGTCTACAAAATTGGTGGTATTGGTACTGTTCCTGTTGGCCGAGTGGAGACTGGTGTTCTCAAACCCGGTATGGTGGTCACCTTTGCTCCAGTCAACGTTACAACGGAAG 1016
BY794942.2  783   ...................................................................................................................................................... 932
DC527884.1  786   ...................................................................................................................................................... 935
CF111220.1  779   ..................TC.....A.....C..............T........C..C.....C.....C.....G...........A.................T...........T.................T..A.....T.... 928
BX426149.2  943   ...................................................................................................................................................... 794
CO001006.1  989   ....C...........A........TT...................T.......................A.....G..T..T.......................T..C.................C........T..A.....T..G. 840
BX418564.2  953   ....................C..................................................................................................................................... 804
BX426150.2  754   ...................................................................................................................................................... 903
AL567209.3  913   ...................................................................................................................................................... 764
BX440470.1  955   ...................................................................................................................................................... 806
BX420340.1  948   ...........R.......................................................................................................................................... 799

Query       1017  TAAAATCTGTCGAAATGCACCATGAAGCTTTGAGTGAAGCTCTTCCTGGGGACAATGTGGGCTTCAATGTCAAGAATGTGTCTGTCAAGGATGTTCGTCGTGGCAACGTTGCTGGTGACAGCAAAAATGACCCACCAATGGAAGCAGCTG 1166
BY794942.2  933   ...................................................................................................................................................... 1082
DC527884.1  936   ...................................................................................................................................................... 1085
CF111220.1  929   .C..G....G................................G..............A.........C.A......C...........A..C..A.A.........T........G.................................. 1078
BX426149.2  793   ...................................................................................................................................................... 644
CO001006.1  839   .G..G..C..A.............................C...................................C...........A.....C...........T..G....................T.....C........T.... 690
BX418564.2  803   ....................................................................................C................................................................. 654
BX426150.2  904   ........................................................................................................-...............-...........-.........-....... 1050
                                                                 \
                                                                 |
                                                                 G
AL567209.3  763   ..............................................................................................................................-....................... 615
BX440470.1  805   ...................................................................................................................................................... 656
```

```
BX420340.1  798   ......................................................................................................................................................  649

Query       1167  GCTTCACTGCTCAGGTGATTATCCTGAACCATCCAGGCCAAATAAGCGCCGGCTATGCCCCTGTATTGGATTGCCACACGGCTCACATTGCATGCAAGTTTGCTGAGCTGAAGGAAAAGATTGATCGCCGTTCTGGTAAAAAGCTGGAAG  1316
BY794942.2  1083  ......................................................................................................................................................  1233
                                                  \
                                                  |
                                                  G
DC527884.1  1086  ......................................................................................................................................................  1235
CF111220.1  1079  ........................................G..C..T..T..............TC....C...........C.....A..............C.....T..A..G.....C.....T..........G...........  1228
BX426149.2  643   ......................................................................................................................................................  494
CO001006.1  689   ..C....A..............T....................C..T.GT..A.....A.....GC......T.....A............T.......................G...........T........G.............  540
BX418564.2  653   ....................................................................................................................M.................................  504
BX426150.2  1051  ..-...................-..................-.......-.......-................-......T.-.............  1140
AL567209.3  614   ......................................................................................................................................................  465
BX440470.1  655   ......................................................................................................................................................  505
                                                                                                                                         \
                                                                                                                                         |
                                                                                                                                         C
BX420340.1  648   ......................................................................................................................................................  499

Query       1317  ATGGCCCTAAATTCTTGAAGTCTGGTGATGCTGCCATTGTTGATATGGTTCCTGGCAAGCCCATGTGTGTTGAGAGCTTCTCAGACTATCCACCTTTGGGTCGCTTTGCTGTTCGTGATATGAGACAGACAGTTGCGGTGGGTGTCATCA  1466
BY794942.2  1234  ......................................................................................................................................................  1383
DC527884.1  1236  ................................................................................................................................................A.....  1385
CF111220.1  1229  .......C...................................C.....C.......................A........T.....C..T..AC.T.....T..............C.....G...........T.............  1378
BX426149.2  493   ......................................................................................................................................................  344
CO001006.1  539   ...................A........C........C.......................T........C........T..T..T.....T..C.....C..T........G.....C..............C..T.............  390
BX418564.2  503   ......................................................................................................................................................  354
AL567209.3  464   .................................................K....................................................................................................  315
BX440470.1  504   ......................................................................................................................................................  355
BX420340.1  498   ......................................................................................................................................................  349

Query       1467  AAGCAGTGGACAAGAAGGCTGCTGGAGCTGGCAAGGTCACCAAGTCTGCCCAGAAAGCTCAGAAGGCTAAATGAATATTATCCCTAATACCTGCCACCCCACTCTTAATCAGTGGTGGAAGAACGGTCTCAGAACTGTTTGTTTCAATTG  1616
BY794942.2  1384  ......................................................................................................................................................  1533
DC527884.1  1386  .........................................................................................................C............................................  1535
CF111220.1  1379  ....C.................A...........A....................................................C.............G........................................C.......  1528
BX426149.2  343   ......................................................................................................................................................  194
CO001006.1  389   ...................A................................................................C................G........................................C.......  240
BX418564.2  353   ......................................................................................................................................................  204
AL567209.3  314   ............................................................M......Y..................................................................................  164
                                                                                                                                    \
                                                                                                                                    |
                                                                                                                                    A
BX440470.1  354   ......................................................................................................................................................  205
BX420340.1  348   ......................................................................................................................................................  199

Query       1617  GCCATTTAAGTTTAGTAGTAAAAGACTGGTTAATGATAACAATGCATCGTAAAACCTTCAGAAGGAAAGGAGAATgttttgtggaccactttggtttctttttttgcgtgtggcagttttaagttattagtttttaaaatcagtactttt  1766
BY794942.2  1534  ......................................................................................................................................................  1683
DC527884.1  1536  ......................................................................................................................................................  1685
CF111220.1  1529  ..............A....................................................---..................-...--....-..G.----...........................C...............  1667
BX426149.2  193   ......................................................................................................................................................  44
CO001006.1  239   ..............A.........................................................................-.A..-....G.-----.--........-.................................  99
                                                                                                  \
                                                                                                  |
                                                                                                  T
BX418564.2  203   ......................................................................................................................................................  54
AL567209.3  163   ......................................................................................................................................................  13
                                                                                                        \
                                                                                                        |
                                                                                                        G
BX440470.1  204   ......................................................................................................................................................  55
BX420340.1  198   ......................................................................................................................................................  49

Query       1767  taATGGAAACAACTTGACCAAAAATTTGTCACAGAATTTTGAGACCCATTAAAAAAGTTAAATG  1830
BY794942.2  1684  ................................................................  1747
DC527884.1  1686  ................................................................  1749
CF111220.1  1668  .........................C....................-............T....  1731
                                                                        \
                                                                        |
                                                                        C
BX426149.2  43    .....-.......-.....G......G.-..G...........T..                    1
CO001006.1  98    .....A...................C.............-...................T....  35
                                                                          \
                                                                          |
                                                                          A
BX418564.2  53    .......................................M-.............            1
AL567209.3  12    -....-..-...-...                                                  1
BX440470.1  54    ......................................................            1
BX420340.1  48    ..........................................R.....                  1
```

Query= N9-C* [organism=Homo sapiens] eef1a1

Length=2090

```
                                                                  Score        E     Max
Sequences producing significant alignments:                      (Bits)     Value  ident

BY794942.2 BY794942 Homo sapiens eye Homo sapiens cDNA clone H...  3155       0.0    100%
DC527884.1 DC527884 chimpanzee skin cDNA library PstA Pan trog...  3138       0.0    100%
CF111220.1 Shultzomica04471 Rat lung airway and parenchyma cDN...  2344       0.0    91%
BX426149.2 BX426149 Homo sapiens NEUROBLASTOMA Homo sapiens cD...  2146       0.0    99%
CO001006.1 OC028 pre-oestrum adult sheep ovary library Ovis ar...  2050       0.0    93%
BX418564.2 BX418564 Homo sapiens FETAL BRAIN Homo sapiens cDNA...  2047       0.0    97%
BX426150.2 BX426150 Homo sapiens NEUROBLASTOMA Homo sapiens cD...  2034       0.0    99%
AL567209.3 AL567209 Homo sapiens FETAL BRAIN Homo sapiens cDNA...  2001       0.0    98%
BX440470.1 BX440470 Homo sapiens FETAL BRAIN Homo sapiens cDNA...  1999       0.0    98%
BX420340.1 BX420340 Homo sapiens FETAL BRAIN Homo sapiens cDNA...  1997       0.0    99%

ALIGNMENTS

Query       376   TCAAAGGTGTCGTGAAAACTACCCCTAAAAGCCAAAATGGGAAAGGAAAAGACTCATATCAACATTGTCGTCATTGGACACGTAGATTCGGGCAAGTCCACCACTACTGGCCATCTGATCTATAAATGCGGTGGCATCGACAAAAGAACC  525
BY794942.2  33     ..C..................................................................................................................................................  181
DC527884.1  36     ..C..................................................................................................................................................  184
CF111220.1  28        ......T......G.C...G........A......................C........C.....A..................C..............A..C....C.........C.....T....A................  177
                                               \
                                               |
                                            ATTC
BX426150.2  3     ...C..................................................................................................................................................  152

Query       526   ATTGAAAAATTTGAGAAGGAGGCTGCTGAGATGGGAAAGGGCTCCTTCAAGTATGCCTGGGTCTTGGATAAACTGAAAGCTGAGCGTGAACGTGGTATCACCATTGATATCTCCTTGTGGAAATTTGAGACCAGCAAGTACTATGTGACT  675
BY794942.2  182   ......................................................................................................................................................  331
DC527884.1  185   ..................................................................................................T...................................................  334
CF111220.1  178   ........G.................G...........A........................C...C.....................G...........T.....C...C..........G...........................  327
CO001006.1  1464                                                                                                                                 ...................T..C  1441
BX426150.2  153   ......................................................................................................................................................  302

Query       676   ATCATTGATGCCCCAGGACACAGAGACTTTATCAAAAACATGATTACAGGGACATCTCAGGCTGACTGTGCTGTCCTGATTGTTGCTGCTGGTGTTGGTGAATTTGAAGCTGGTATCTCCAAGAATGGGCAGACCCGAGAGCATGCCCTT  825
BY794942.2  332   ......................................................................................................................................................  481
DC527884.1  335   .............................C........................................................................................................................  484
CF111220.1  328   .............................C.....G...........G..C.................C.............................G..........................C...........T........T...  477
CO001006.1  1440  .............................C....................C....C......................................................C..............C...........T............  1291
BX426150.2  303   ......................................................................................................................................................  452

Query       826   CTGGCTTACACACTGGGTGTGAAACAACTAATTGTCGGTGTTAACAAAATGGATTCCACTGAGCCACCCTACAGCCAGAAGAGATATGAGGAAATTGTTAAGGAAGTCAGCACTTACATTAAGAAAATTGGCTACAACCCCGACACAGTA  975
BY794942.2  482   ......................................................................................................................................................  631
DC527884.1  485   ......................................................................................................................................................  634
CF111220.1  478   ...........TT.............G........T.....C.................C........A.....T...........C........C.................C..........................T.........  627
BX426149.2  1196                                                ......-..A...-.A................G.......................................................................  1095
CO001006.1  1290  ...........C.......................T..C.................T..............T..............C..........................C....................................  1141
BX418564.2  1196                                            ...-....-......-...-...-...YH.VS..-.R......-W.-..G..W....-.R......M.....K....-.R.-......S....-..........-R..  1101
BX426150.2  453   ......................................................................................................................................................  602
AL567209.3  1145                                                                     ......S.....-...W.-..........-K.W........R.....-....A.M.C............C.............  1065
                                                                                                                                 \
                                                                                                                                 |
                                                                                                                                 M
BX440470.1  1128                                                                                                                                   ....-.....-..-W.-.............-  1102
                                                                                                                                                             \
                                                                                                                                                             |
                                                                                                                                                             C
BX420340.1  1117                                                                                                                                              ...M..M.............  1098

Query       976   GCATTTGTGCCAATTTCTGGTTGGAATGGTGACAACATGCTGGAGCCAAGTGCTAACATGCCTTGGTTCAAGGGATGGAAAGTCACCCGTAAGGATGGCAATGCCAGTGGAACCACGCTGCTTGAGGCTCTGGACTGCATCCTACCACCA  1125
BY794942.2  632   ......................................................................................................................................................  781
DC527884.1  635   ......................................................................................................................................................  784
CF111220.1  628   ........................................................T.....G..........................C..A.......G.........C...........G..A..T...........T..G..G...  777
BX426149.2  1094  ......................................................................................................................................................  945
CO001006.1  1140  ....................C...................................T.....A................................C....................C........A........T........G......  991
BX418564.2  1100  ...-.................-.....-..........................C......................-........................................................................  955
BX426150.2  603   ......................................................................................................................................................  752
AL567209.3  1064  ....................................................................................................................................K.................  915
BX440470.1  1101  ...-....-S.M...Y..-.K......-..............-.....................K...................................................................R.................  957
BX420340.1  1097  ......K.-..M.W...YT...D....K.-...........T..........Y...............................................................................A.................  950

Query       1126  ACTCGTCCAACTGACAAGCCCTTGCGCCTGCCTCTCCAGGATGTCTACAAAATTGGTGGTATTGGTACTGTTCCTGTTGGCCGAGTGGAGACTGGTGTTCTCAAACCCGGTATGGTGGTCACCTTTGCTCCAGTCAACGTTACAACGGAA  1275
BY794942.2  782   ......................................................................................................................................................  931
DC527884.1  785   ......................................................................................................................................................  934
CF111220.1  778   ....................TC....A.....C..............T........C..C.....C.....C.....G...........A.................T...........T.................T..A.....T...  927
BX426149.2  944   ......................................................................................................................................................  795
CO001006.1  990   .....C...........A........TT...................T.......................A.....G..T..T.......................T..C.................C........T..A.....T..G  841
BX418564.2  954   .....................C................................................................................................................................  805
BX426150.2  753   ......................................................................................................................................................  902
AL567209.3  914   ......................................................................................................................................................  765
BX440470.1  956   ......................................................................................................................................................  807
BX420340.1  949   ............R.........................................................................................................................................  800
```

```
Query       1276  GTAAAATCTGTCGAAATGCACCATGAAGCTTTGAGTGAAGCTCTTCCTGGGGACAATGTGGGCTTCAATGTCAAGAATGTGTCTGTCAAGGATGTTCGTCGTGGCAACGTTGCTGGTGACAGCAAAAATGACCCACCAATGGAAGCAGCT  1425
BY794942.2  932   ......................................................................................................................................................  1081
DC527884.1  935   ......................................................................................................................................................  1084
CF111220.1  928   ..C..G....G.................................G..............A........C..A.....C...........A..C...A.A........T........G.................................  1077
BX426149.2  794   ......................................................................................................................................................  645
CO001006.1  840   ..G..G..C..A.............................C...................................C...........A......C..........T..G....................T.....C........T...  691
BX418564.2  804   .....................................................................................C................................................................  655
BX426150.2  903   ..........................................................................................................-..............-...........-.........-......  1049
                                                                  \
                                                                  |
                                                                  G
AL567209.3  764   ...............................................................................................................................-......................  616
BX440470.1  806   ......................................................................................................................................................  657
BX420340.1  799   ......................................................................................................................................................  650

Query       1426  GGCTTCACTGCTCAGGTGATTATCCTGAACCATCCAGGCCAAATAAGCGCCGGCTATGCCCCTGTATTGGATTGCCACACGGCTCACATTGCATGCAAGTTTGCTGAGCTGAAGGAAAAGATTGATCGCCGTTCTGGTAAAAAGCTGGAA  1575
BY794942.2  1082  ......................................................................................................................................................  1232
                                                 \
                                                 |
                                                 G
DC527884.1  1085  ......................................................................................................................................................  1234
CF111220.1  1078  .........................................G..C..T..T..............TC....C...........C.....A..............C.....T..A..G.....C.....T...........G.........  1227
BX426149.2  644   ......................................................................................................................................................  495
CO001006.1  690   ...C....A..............T....................C..T.GT..A.....A.....GC.......T.....A...........T.......................G...........T........G............  541
BX418564.2  654   ......................................................................................................................M...............................  505
BX426150.2  1050  ...-...................-..................-.......-.......-................-......T.-.............  1140
AL567209.3  615   ......................................................................................................................................................  466
BX440470.1  656   ......................................................................................................................................................  506
                                                                                                                                        \
                                                                                                                                        |
                                                                                                                                        C
BX420340.1  649   ......................................................................................................................................................  500

Query       1576  GATGGCCCTAAATTCTTGAAGTCTGGTGATGCTGCCATTGTTGATATGGTTCCTGGCAAGCCCATGTGTGTTGAGAGCTTCTCAGACTATCCACCTTTGGGTCGCTTTGCTGTTCGTGATATGAGACAGACAGTTGCGGTGGGTGTCATC  1725
BY794942.2  1233  ......................................................................................................................................................  1382
DC527884.1  1235  ................................................................................................................................................A.....  1384
CF111220.1  1228  ........C...................................C.....C.......................A........T.....C..T..AC.T.....T..............C.....G...........T............  1377
BX426149.2  494   ......................................................................................................................................................  345
CO001006.1  540   .....................A.......C........C.......................T........C........T..T..T.....T..C.....C..T........G.....C..............C..T............  391
BX418564.2  504   ......................................................................................................................................................  355
AL567209.3  465   ..................................................K...................................................................................................  316
BX440470.1  505   ......................................................................................................................................................  356
BX420340.1  499   ......................................................................................................................................................  350

Query       1726  AAAGCAGTGGACAAGAAGGCTGCTGGAGCTGGCAAGGTCACCAAGTCTGCCCAGAAAGCTCAGAAGGCTAAATGAATATTATCCCTAATACCTGCCACCCCACTCTTAATCAGTGGTGGAAGAACGGTCTCAGAACTGTTTGTTTCAATT  1875
BY794942.2  1383  ......................................................................................................................................................  1532
DC527884.1  1385  ..........................................................................................................C...........................................  1534
CF111220.1  1378  .....C.................A...........A....................................................C.............G........................................C......  1527
BX426149.2  344   ......................................................................................................................................................  195
CO001006.1  390   .....................A...............................................................C................G........................................C......  241
BX418564.2  354   ......................................................................................................................................................  205
AL567209.3  315   .............................................................M......Y.................................................................................  165
                                                                                                                                   \
                                                                                                                                   |
                                                                                                                                   A
BX440470.1  355   ......................................................................................................................................................  206
BX420340.1  349   ......................................................................................................................................................  200

Query       1876  GGCCATTTAAGTTTAGTAGTAAAAGACTGGTTAATGATAACAATGCATCGTAAAACCTTCAGAAGGAAAGGAGAATgttttgtggaccactttggttttctttttttgcgtgtggcagttttaagttattagtttttaaaatcagtacttt  2025
BY794942.2  1533  ......................................................................................................................................................  1682
DC527884.1  1535  ......................................................................................................................................................  1684
CF111220.1  1528  ...............A....................................................---..................-...--....-..G.----...........................C..............  1666
BX426149.2  194   ......................................................................................................................................................  45
CO001006.1  240   ...............A.........................................................................-.A..-....G.-----.--........-................................  100
                                                                                                   \
                                                                                                   |
                                                                                                   T
BX418564.2  204   ......................................................................................................................................................  55
AL567209.3  164   ......................................................................................................................................................  14
                                                                                                         \
                                                                                                         |
                                                                                                         G
BX440470.1  205   ......................................................................................................................................................  56
BX420340.1  199   ......................................................................................................................................................  50

Query       2026  ttaATGGAAACAACTTGACCAAAAATTTGTCACAGAATTTTGAGACCCATTAAAAAAGTTAAATG  2090
BY794942.2  1683  .................................................................  1747
DC527884.1  1685  .................................................................  1749
CF111220.1  1667  ..........................C....................-............T....  1731
                                                                         \
                                                                         |
```

```
                                                                         C
BX426149.2  44    ......-.......-.....G......G.-..G...........T..                    1
CO001006.1  99    ......A...................C.............-...................T....  35
                                                                           \
                                                                           |
                                                                           A
BX418564.2  54    ........................................M-.............            1
AL567209.3  13    .-....-..-...-...                                                  1
BX440470.1  55    .......................................................            1
BX420340.1  49    ...........................................R.....                  1

Query= N10-C* [organism=Homo sapiens] eef1a1

Length=1743


                                                                    Score        E     Max
Sequences producing significant alignments:                        (Bits)     Value  ident

DC527884.1 DC527884 chimpanzee skin cDNA library PstA Pan trog...  3190       0.0    100%
BY794942.2 BY794942 Homo sapiens eye Homo sapiens cDNA clone H...  3171       0.0    100%
CF111220.1 Shultzomica04471 Rat lung airway and parenchyma cDN...  2357       0.0    91%
BX426149.2 BX426149 Homo sapiens NEUROBLASTOMA Homo sapiens cD...  2146       0.0    99%
CO001006.1 OC028 pre-oestrum adult sheep ovary library Ovis ar...  2050       0.0    93%
BX418564.2 BX418564 Homo sapiens FETAL BRAIN Homo sapiens cDNA...  2047       0.0    97%
BX426150.2 BX426150 Homo sapiens NEUROBLASTOMA Homo sapiens cD...  2032       0.0    99%
AL567209.3 AL567209 Homo sapiens FETAL BRAIN Homo sapiens cDNA...  2001       0.0    98%
BX440470.1 BX440470 Homo sapiens FETAL BRAIN Homo sapiens cDNA...  1999       0.0    98%
BX420340.1 BX420340 Homo sapiens FETAL BRAIN Homo sapiens cDNA...  1997       0.0    99%

ALIGNMENTS

Query       1     TTTTTTCGCAACGGGTTTGCCGCCAGAACACAAGGTGTCGTGAAAACTACCCCTAAAAGCCAAAATGGGAAAGGAAAAGACTCATATCAACATTGTCGTCATTGGACACGTAGATTCGGGCAAGTCCACCACTACTGGCCATCTGATCTA  150
DC527884.1  8     ...............................-......................................................................................................................  156
BY794942.2  25                        ...........-......................................................................................................................  153
CF111220.1  7               .........-..T......G.-.......T......G.C...G........A.......................C........C.....A.................C..............A..C.....C........  149
                             \                                           \
                               |                                         |
                               A                                       ATTC
BX426150.2  1                                  ..T...-......................................................................................................................  124

Query       151   TAAATGCGGTGGCATCGACAAAAGAACCATTGAAAAATTTGAGAAGGAGGCTGCTGAGATGGGAAAGGGCTCCTTCAAGTATGCCTGGGTCTTGGATAAACTGAAAGCTGAGCGTGAACGTGGTATCACCATTGATATCTCCTTGTGGAA  300
DC527884.1  157   ..............................................................................................................................T.......................  306
BY794942.2  154   ......................................................................................................................................................  303
CF111220.1  150   C.....T.....A.......................G.................G...........A........................C....C....................G...........T.....C......C.......  299
BX426150.2  125   ......................................................................................................................................................  274

Query       301   ATTTGAGACCAGCAAGTACTATGTGACTATCATTGATGCCCCAGGACACAGAGACTTTATCAAAAACATGATTACAGGGACATCTCAGGCTGACTGTGCTGTCCTGATTGTTGCTGCTGGTGTTGGTGAATTTGAAGCTGGTATCTCCAA  450
DC527884.1  307   .........................................................C............................................................................................  456
BY794942.2  304   ......................................................................................................................................................  453
CF111220.1  300   G........................................................C.....G...........G..C.................C.............................G.......................  449
CO001006.1  1464      ....................T..C.............................C....................C.....C.....................................................C...........  1319
BX426150.2  275   ......................................................................................................................................................  424

Query       451   GAATGGGCAGACCCGAGAGCATGCCCTTCTGGCTTACACACTGGGTGTGAAACAACTAATTGTCGGTGTTAACAAAATGGATTCCACTGAGCCACCCTACAGCCAGAAGAGATATGAGGAAATTGTTAAGGAAGTCAGCACTTACATTAA  600
DC527884.1  457   ......................................................................................................................................................  606
BY794942.2  454   ......................................................................................................................................................  603
CF111220.1  450   ...C...........T........T..............TT.............G........T.....C.................C........A.....T...........C........C.................C........  599
BX426149.2  1196                                                                            ....-..A......-.A...............G...........................................  1123
CO001006.1  1318  ...C...........T.......................C.......................T..C.................T..............T..............C..........................C........  1169
BX418564.2  1196                                                                       ....-....-......-...-...-...YH.VS..-.R......-W.-..G..W....-.R......M.....K....-.R  1126
BX426150.2  425   ......................................................................................................................................................  574
AL567209.3  1145                                                                                                ........S....-....W.-..........-K..W.......R.....-....A.M  1093
                                                                                                                                                               \
                                                                                                                                                               |
                                                                                                                                                               M
BX440470.1  1128                                                                                                                                                     ...  1126

Query       601   GAAAATTGGCTACAACCCCGACACAGTAGCATTTGTGCCAATTTCTGGTTGGAATGGTGACAACATGCTGGAGCCAAGTGCTAACATGCCTTGGTTCAAGGGATGGAAAGTCACCCGTAAGGATGGCAATGCCAGTGGAACCACGCTGCT  750
DC527884.1  607   ......................................................................................................................................................  756
BY794942.2  604   ......................................................................................................................................................  753
CF111220.1  600   ..................T.................................................................T.....G..........................C..A.......G.........C...........  749
BX426149.2  1122  ......................................................................................................................................................  973
CO001006.1  1168  ................................................C...................................T.....A................................C....................C.....  1019
BX418564.2  1125  .-......S....-..........-R.....-.................-.....-..........................C......................-............................................  983
BX426150.2  575   ......................................................................................................................................................  724
AL567209.3  1092  .C............C.......................................................................................................................................  943
BX440470.1  1125  .-.....-..-W.-.............-..-.....-S.M...Y..-.K......-..............-.....................K.........................................................  985
                                     \
                                     |
                                     C
BX420340.1  1117          ...M..M...................K.-..M.W...YT...D....K.-...........T..........Y.....................................................................  978
```

```
Query       751   TGAGGCTCTGGACTGCATCCTACCACCAACTCGTCCAACTGACAAGCCCTTGCGCCTGCCTCTCCAGGATGTCTACAAAATTGGTGGTATTGGTACTGTTCCTGTTGGCCGAGTGGAGACTGGTGTTCTCAAACCCGGTATGGTGGTCAC  900
DC527884.1  757   ......................................................................................................................................................  906
BY794942.2  754   ......................................................................................................................................................  903
CF111220.1  750   G..A...T..........T..G..G.......................TC....A.....C..............T........C..C.....C.....C.....G...........A.................T...........T..  899
BX426149.2  972   ......................................................................................................................................................  823
CO001006.1  1018  ...A........T........G...........C...........A........TT...................T.......................A.....G..T..T.......................T..C...........  869
BX418564.2  982   .................................................C....................................................................................................  833
BX426150.2  725   ......................................................................................................................................................  874
AL567209.3  942   ..........K...........................................................................................................................................  793
BX440470.1  984   ..........R...........................................................................................................................................  835
BX420340.1  977   ..........A.............................R.............................................................................................................  828

Query       901   CTTTGCTCCAGTCAACGTTACAACGGAAGTAAAATCTGTCGAAATGCACCATGAAGCTTTGAGTGAAGCTCTTCCTGGGGACAATGTGGGCTTCAATGTCAAGAATGTGTCTGTCAAGGATGTTCGTCGTGGCAACGTTGCTGGTGACAG  1050
DC527884.1  907   ......................................................................................................................................................  1056
BY794942.2  904   ......................................................................................................................................................  1053
CF111220.1  900   ...............T..A.....T.....C..G.....G................................G..............A........C..A.....C...........A..C.....A..A.....T........G.....  1049
BX426149.2  822   ......................................................................................................................................................  673
CO001006.1  868   ......C........T..A.....T..G..G..G..C..A.............................C...................................C...........A.....C...........T..G...........  719
BX418564.2  832   .................................................................................................................C....................................  683
BX426150.2  875   .....................................................................................................................................-...............-  1023
                                                                                              \
                                                                                              |
                                                                                              G
AL567209.3  792   ......................................................................................................................................................  643
BX440470.1  834   ......................................................................................................................................................  685
BX420340.1  827   ......................................................................................................................................................  678

Query       1051  CAAAAATGACCCACCAATGGAAGCAGCTGGCTTCACTGCTCAGGTGATTATCCTGAACCATCCAGGCCAAATAAGCGCCGGCTATGCCCCTGTATTGGATTGCCACACGGCTCACATTGCATGCAAGTTTGCTGAGCTGAAGGAAAAGAT  1200
DC527884.1  1057  ......................................................................................................................................................  1206
BY794942.2  1054  ......................................................................................................................................................  1204
                                                                               \
                                                                               |
                                                                               G
CF111220.1  1050  .....................................................................G..C..T..T..............TC....C...........C.....A..............C.....T..A..G.....  1199
BX426149.2  672   ......................................................................................................................................................  523
CO001006.1  718   .........T.....C........T......C....A..............T....................C..T.GT..A.....A.....GC.......T.....A...........T.......................G.....  569
BX418564.2  682   ..................................................................................................................................................M...  533
BX426150.2  1024  ...........-.........-.........-...................-..................-.......-.......-................-......T.-.............  1140
AL567209.3  642   .....-................................................................................................................................................  494
BX440470.1  684   ......................................................................................................................................................  534
                                                                                                                                                                      \
                                                                                                                                                                      |
                                                                                                                                                                      C
BX420340.1  677   ......................................................................................................................................................  528

Query       1201  TGATCGCCGTTCTGGTAAAAAGCTGGAAGATGGCCCTAAATTCTTGAAGTCTGGTGATGCTGCCATTGTTGATATGGTTCCTGGCAAGCCCATGTGTGTTGAGAGCTTCTCAGACTATCCACCTTTGGGTCGCTTTGCTGTTCGTGATAT  1350
DC527884.1  1207  ......................................................................................................................................................  1356
BY794942.2  1205  ......................................................................................................................................................  1354
CF111220.1  1200  C.....T...........G.................C...................................C.....C.......................A........T.....C..T..AC.T.....T..............C..  1349
BX426149.2  522   ......................................................................................................................................................  373
CO001006.1  568   ......T........G................................A........C........C.......................T........C........T..T..T.....T..C.....C..T........G.....C..  419
BX418564.2  532   ......................................................................................................................................................  383
AL567209.3  493   ..............................................................................K.......................................................................  344
BX440470.1  533   ......................................................................................................................................................  384
BX420340.1  527   ......................................................................................................................................................  378

Query       1351  GAGACAGACAGTTGCGGTGGGTGTCATCAAAGCAGTGGACAAGAAGGCTGCTGGAGCTGGCAAGGTCACCAAGTCTGCCCAGAAAGCTCAGAAGGCTAAATGAATATTATCCCTAATACCTGCCACCCCACTCTTAATCAGTGGTGGAAG  1500
DC527884.1  1357  ......................A...............................................................................................................C...............  1506
BY794942.2  1355  ......................................................................................................................................................  1504
CF111220.1  1350  ...G...........T.................C.................A...........A....................................................C.............G...................  1499
BX426149.2  372   ......................................................................................................................................................  223
CO001006.1  418   ............C..T................................A................................................................C................G...................  269
BX418564.2  382   ......................................................................................................................................................  233
AL567209.3  343   .........................................................................................M......Y.....................................................  193
                                                                                                                                                                 \
                                                                                                                                                                 |
                                                                                                                                                                 A
BX440470.1  383   ......................................................................................................................................................  234
BX420340.1  377   ......................................................................................................................................................  228

Query       1501  AACGGTCTCAGAACTGTTTGTTTCAATTGGCCATTTAAGTTTAGTAGTAAAAGACTGGTTAATGATAACAATGCATCGTAAAACCTTCAGAAGGAAAGGAGAATgttttgtggaccactttggttttctttttttgcgtgtggcagtttta  1650
DC527884.1  1507  ......................................................................................................................................................  1656
BY794942.2  1505  ......................................................................................................................................................  1654
CF111220.1  1500  .....................C.....................A....................................................---..................-...--....-..G.----..............  1638
BX426149.2  222   ......................................................................................................................................................  73
CO001006.1  268   .....................C.....................A.........................................................................-.A..-....G.-----.--........-....  128
                                                                                                                             \
                                                                                                                             |
                                                                                                                             T
BX418564.2  232   ......................................................................................................................................................  83
AL567209.3  192   ......................................................................................................................................................  42
```

```
                                                                                                                                     \
                                                                                                                                     |
                                                                                                                                     G
BX440470.1  233   ......................................................................................................................................................  84
BX420340.1  227   ......................................................................................................................................................  78

Query       1651  agttattagtttttaaaatcagtactttttaATGGAAACAACTTGACCAAAAATTTGTCACAGAATTTTGAGACCCATTAAAAAAGTTAAATG  1743
DC527884.1  1657  .............................................................................................  1749
BY794942.2  1655  .............................................................................................  1747
CF111220.1  1639  .............C........................................C....................-............T....  1731
                                                                                                     \
                                                                                                     |
                                                                                                     C
BX426149.2  72    ..................................-.......-.....G......G.-..G...........T..                    1
CO001006.1  127   ..................................A...................C.............-...................T....  35
                                                                                                       \
                                                                                                       |
                                                                                                       A
BX418564.2  82    ....................................................................M-.............            1
AL567209.3  41    .............................-....-..-...-...                                                  1
BX440470.1  83    ...................................................................................            1
BX420340.1  77    .......................................................................R....                   1
```

Query= N11-9 [organism=Homo sapiens] eef1a1

Length=1733

Sequences producing significant alignments:	Score (Bits)	E Value	Max ident
DC527884.1 DC527884 chimpanzee skin cDNA library PstA Pan trog...	3134	0.0	99%
BY794942.2 BY794942 Homo sapiens eye Homo sapiens cDNA clone H...	3118	0.0	99%
CF111220.1 Shultzomica04471 Rat lung airway and parenchyma cDN...	2314	0.0	91%
BX426149.2 BX426149 Homo sapiens NEUROBLASTOMA Homo sapiens cD...	2089	0.0	98%
CO001006.1 OC028 pre-oestrum adult sheep ovary library Ovis ar...	1999	0.0	92%
BX418564.2 BX418564 Homo sapiens FETAL BRAIN Homo sapiens cDNA...	1988	0.0	96%
BX426150.2 BX426150 Homo sapiens NEUROBLASTOMA Homo sapiens cD...	1978	0.0	98%
AL567209.3 AL567209 Homo sapiens FETAL BRAIN Homo sapiens cDNA...	1945	0.0	97%
BX440470.1 BX440470 Homo sapiens FETAL BRAIN Homo sapiens cDNA...	1943	0.0	98%
BX420340.1 BX420340 Homo sapiens FETAL BRAIN Homo sapiens cDNA...	1943	0.0	98%

ALIGNMENTS

```
Query       2     TTTTTCGCAACGGGTTTGCCGCCAGAACACAGGTGTCGTGAAAACTACCCCTAAAAGCCAAAATGGGAAAGGAAAAGACTCATATCAACATTGTCGTCATTGGACACGTAGATTCGGGCAAGTCCACCACTACTGGCCATCTGATCTATA  151
DC527884.1  9     ......................................................................................................................................................  158
BY794942.2  25                       ...................................................................................................................................  155
CF111220.1  7               ........-..T......G.......T......G.C...G........A.......................C........C.....A.................C..............A..C.....C........C.  151
                              \                                        \
                              |                                        |
                              A                                     ATTC
BX426150.2  1                             ..T...........................................................................................................................  126

Query       152   AATGCGGTGGCATCGACAAAAGAACCATTGAAAAATTTGAGAAGGAGGCTGCTGAGATGGGAAAGGGCTCCTTCAAGTATGCCTGGGTCTTGGATAAACTGAAAGCTGAGCGTGAACGTGGTATCACCATTGATATCTCCTTGTGGAAAT  301
DC527884.1  159   ............................................................................................................................T.........................  308
BY794942.2  156   ......................................................................................................................................................  305
CF111220.1  152   ....T.....A.......................G.................G...........A........................C....C....................G...........T.....C......C.......G.  301
BX426150.2  127   ......................................................................................................................................................  276

Query       302   TTGAGACCAGCAAGTACTATGTGACTATCATTGATGCCCCAGGACACAGAGACTTTATCAAAAACATGATTACAGGGACATCTCAGGCTGACTGTGCTGTCCTGATTGTTGCTGCTGGTGTTGGTGAATTTGAAGCTGGTATCTCCAAGA  451
DC527884.1  309   .......................................................C..............................................................................................  458
BY794942.2  306   ......................................................................................................................................................  455
CF111220.1  302   .......................................................C.....G...........G..C.................C.............................G.........................  451
CO001006.1  1464    ....................T..C.............................C....................C.....C.....................................................C.............  1317
BX426150.2  277   ......................................................................................................................................................  426

Query       452   ATGGGCAGACCCGAGAGCATGCCCTTCTGGCTTACACACTGGGTGTGAAACAACTAATTGTCGGTGTTAACAAAATGGATTCCACTGAGCCACCCTACAGCCAGAAGAGATATGAGGAAATTGTTAAGGAAGTCAGCACTTACATTAAGA  601
DC527884.1  459   ......................................................................................................................................................  608
BY794942.2  456   ......................................................................................................................................................  605
CF111220.1  452   .C...........T........T..............TT.............G........T.....C.................C........A.....T...........C........C.................C..........  601
BX426149.2  1196                                                                          ......-..A....-.A...............G.............................................  1121
CO001006.1  1316  .C...........T.......................C.......................T..C.................T..............T..............C..........................C..........  1167
BX418564.2  1196                                                                     ....-....-......-...-...-...YH.VS..-.R......-W.-..G..W....-.R......M.....K....-.R.-  1125
BX426150.2  427   ......................................................................................................................................................  576
AL567209.3  1145                                                                                              ......S.....-...W.-.........-K.W........R.....-....A.M.C  1091
                                                                                                                                                                 \
                                                                                                                                                                 |
                                                                                                                                                                 M
BX440470.1  1128                                                                                                                                                   ....-  1125

Query       602   AAATTGGCTACAACCCCGACACAGTAGCATTTGTGCCAATTTCTGGTTGGAATGGTGACAACATGCTGGAGCCAAGTGCTAACATGCCTTGGTTCAAGGGATGGAAAGTCACCCGTAAGGATGGCAATGCCAGTGGAACCACGCTGCTTG  751
DC527884.1  609   ......................................................................................................................................................  758
```

```
BY794942.2  606   ......................................................................................................................................................  755
CF111220.1  602   ................T.................................................................T.....G..........................C..A.......G.........C...........G.  751
BX426149.2  1120  ......................................................................................................................................................  971
CO001006.1  1166  ..............................................C...................................T.....A................................C....................C.......  1017
BX418564.2  1124  ......S....-..........-R.....-.................-.....-..........................C......................-..............................................  981
BX426150.2  577   ......................................................................................................................................................  726
AL567209.3  1090  ............C.........................................................................................................................................  941
BX440470.1  1124  .....-..-W.-.............-...-....-S.M...Y..-.K......-..............-.....................K...........................................................  983
                                     \
                                     |
                                     C
BX420340.1  1117        ...M..M...................K.-..M.W...YT...D....K.-...........T..........Y.......................................................................  976

Query       752   AGGCTCTGACTGCATCCTACACCAACTCGTCAACTGACAACCTTGCGCCTGCCTCTCCAGGATGTCTACAAAATGTGTATTGGTACTGTTCCTGTTGGCCGAGTGAGACTGGTGTTCTCAAACCCGGTATGGTGGTCACCTTTGCTCCAG  901
DC527884.1  759   ......................................................................................................................................................  917
                          \          \          \         \                                 \ \                           \
                          |          |          |         |                                 | |                           |
                          G          C          C        GC                                TG G                           G
BY794942.2  756   ......................................................................................................................................................  914
                          \          \          \         \                                 \ \                           \
                          |          |          |         |                                 | |                           |
                          G          C          C        GC                                TG G                           G
CF111220.1  752   .A...T.........T..C.G..........................A.....C..............T......G.C.....C.....C.....G..........A.................T...........T.............  910
                          \         \            \        \  \                              \\                            \
                          |         |            |        |  |                              ||                            |
                          G         G            C        G  C                             TG|                            G
                                                                                             |
                                                                                             C
BX426149.2  970   ......................................................................................................................................................  812
                          \           \         \         \                                 \ \                            \
                          |           |         |         |                                 | |                            |
                          G           C         C        GC                                TG G                            G
CO001006.1  1016  .A.......T........C..........C.................TT...................T....................A.....G..T..T......................T..C.................C....  858
                          \         \          \        \ \                                 \ \                            \
                          |         |          |        | |                                 | |                            |
                          G         G          C        A C                                TG G                            G
BX418564.2  980   ..........................................C.............................................................................................................  822
                          \           \         \         \                                 \ \                            \
                          |           |         |         |                                 | |                            |
                          G           C         C        GC                                TG G                            G
BX426150.2  727   ......................................................................................................................................................  885
                         \            \          \        \                                 \\\                           \
                         |            |          |        |                                 |||                           |
                         G            C          C       GC                                 T||                           G
                                                                                             ||
                                                                                             G|
                                                                                              |
                                                                                              G
AL567209.3  940   ......................................................................................................................................................  782
                          \          \          \         \                                 \ \                            \
                          |          |          |         |                                 | |                            |
                          K          C          C        GC                                TG G                            G
BX440470.1  982   ......................................................................................................................................................  824
                          \          \          \         \                                 \ \                            \
                          |          |          |         |                                 | |                            |
                          R          C          C        GC                                TG G                            G
BX420340.1  975   ...................................R..................................................................................................................  817
                           \         \          \         \                                 \ \                            \
                           |         |          |         |                                 | |                            |
                           A         C          C        GC                                TG G                            G

Query       902   TCAACGTTACAACGGAAGTAAAATCTGTCGAAATGCACCATGAAGCTTTGAGTGAAGCTCTTCCTGGGGACAATGTGGGCTTCAATGTCAAGAATGTGTCTGTCAAGGATGTTCGTCGTGGCAACGTTGCTGGTGACAGCAAAAATGACC  1051
DC527884.1  918   ......................................................................................................................................................  1067
BY794942.2  915   ......................................................................................................................................................  1064
CF111220.1  911   ....T..A.....T.....C..G.....G................................G..............A........C..A.....C...........A..C...A.A........T........G................  1060
BX426149.2  811   ......................................................................................................................................................  662
CO001006.1  857   ....T..A.....T..G..G..G..C..A.............................C...................................C...........A.....C...........T..G....................T.  708
BX418564.2  821   ......................................................................................................C...............................................  672
BX426150.2  886   ..........................................................................................................................-...............-...........  1034
                                                                                   \
                                                                                   |
                                                                                   G
AL567209.3  781   ................................................................................................................................................-.....  633
BX440470.1  823   ......................................................................................................................................................  674
BX420340.1  816   ......................................................................................................................................................  667

Query       1052  CACCAATGGAAGCAGCTGGCTTCACTGCTCAGGTGATTATCCTGAACCATCCAGGCCAAATAAGCGCCGGCTATGCCCCTGTATTGGATTGCCACACGGCTCACATTGCATGCAAGTTTGCTGAGCTGAAGGAAAAGATTGATCGCCGTT  1201
DC527884.1  1068  ......................................................................................................................................................  1217
BY794942.2  1065  ......................................................................................................................................................  1215
                                                                    \
                                                                    |
                                                                    G
```

```
CF111220.1  1061  ..........................................................G..C..T..T..............TC....C...........C.....A..............C.....T..A..G.....C.....T....  1210
BX426149.2  661   ......................................................................................................................................................  512
CO001006.1  707   ....C........T......C....A..............T....................C..T..GT.A.....A.....GC........T.....A...........T......................G...........T....  558
BX418564.2  671   ........................................................................................................................................M.............  522
BX426150.2  1035  -.........-.........-...................-..................-.......-.......-................-......T.-.............                                   1140
AL567209.3  632   ......................................................................................................................................................  483
BX440470.1  673   ......................................................................................................................................................  523
                                                                                                                                                                 \
                                                                                                                                                                 |
                                                                                                                                                                 C
BX420340.1  666   ......................................................................................................................................................  517

Query       1202  CTGGTAAAAAGCTGGAAGATGGCCCTAAATTCTTGAAGTCTGGTGATGCTGCCATTGTTGATATGGTTCCTGGCAAGCCCATGTGTGTTGAGAGCTTCTCAGACTATCCACCTTTGGGTCGCTTTGCTGTTCGTGATATGAGACAGACAG  1351
DC527884.1  1218  ......................................................................................................................................................  1367
BY794942.2  1216  ......................................................................................................................................................  1365
CF111220.1  1211  .......G.................C...................................C....C........................A........T.....C..T..AC.T.....T...............C....G.......  1360
BX426149.2  511   ......................................................................................................................................................  362
CO001006.1  557   ....G................................A........C........C.......................T........C........T..T..T.....T..C.....C..T........G.....C.............  408
BX418564.2  521   ......................................................................................................................................................  372
AL567209.3  482   ...................................................................K..................................................................................  333
BX440470.1  522   ......................................................................................................................................................  373
BX420340.1  516   ......................................................................................................................................................  367

Query       1352  TTGCGGTGGGTGTCATCAAAGCAGTGGACAAGAAGGCTGCTGGAGCTGGCAAGGTCACCAAGTCTGCCCAGAAAGCTCAGAAGGCTAAATGAATATTATCCCTAATACCTGCCACCCCACTCTTAATCAGTGGTGGAAGAACGGTCTCAG  1501
DC527884.1  1368  ...........A...............................................................................................................C..........................  1517
BY794942.2  1366  ......................................................................................................................................................  1515
CF111220.1  1361  ....T.................C.................A...........A....................................................C.............G..............................  1510
BX426149.2  361   ......................................................................................................................................................  212
CO001006.1  407   .C..T................................A................................................................C................G..............................  258
BX418564.2  371   ......................................................................................................................................................  222
AL567209.3  332   ..............................................................................M......Y................................................................  182
                                                                                                                                                      \
                                                                                                                                                      |
                                                                                                                                                      A
BX440470.1  372   ......................................................................................................................................................  223
BX420340.1  366   ......................................................................................................................................................  217

Query       1502  AACTGTTTGTTTCAATTGGCCATTTAAGTTTAGTAGTAAAAGACTGGTTAATGATAACAATGCATCGTAAAACCTTCAGAAGGAAAGGAGAATgttttgtggaccactttggttttctttttttgcgtgtggcagttttaagttattagtt  1651
DC527884.1  1518  ......................................................................................................................................................  1667
BY794942.2  1516  ......................................................................................................................................................  1665
CF111220.1  1511  ..........C.....................A....................................................---..................-...--....-..G.----.........................  1649
BX426149.2  211   ......................................................................................................................................................  62
CO001006.1  257   ..........C.....................A.........................................................................-.A...-......G.-----.--.....-...............  117
                                                                                                                  \
                                                                                                                  |
                                                                                                                  T
BX418564.2  221   ......................................................................................................................................................  72
AL567209.3  181   ......................................................................................................................................................  31
                                                                                                                        \
                                                                                                                        |
                                                                                                                        G
BX440470.1  222   ......................................................................................................................................................  73
BX420340.1  216   ......................................................................................................................................................  67

Query       1652  tttaaaatcagtactttttaATGGAAACAACTTGACCAAAAATTTGTCACAGAATTTTGAGACCCATTAAAAAAGTTAAATG  1733
DC527884.1  1668  ..................................................................................  1749
BY794942.2  1666  ..................................................................................  1747
CF111220.1  1650  ..C........................................C....................-............T....  1731
                                                                                          \
                                                                                          |
                                                                                          C
BX426149.2  61    .......................-.......-.....G......G.-..G...........T..                    1
CO001006.1  116   .......................A...................C.............-...................T....  35
                                                                                            \
                                                                                            |
                                                                                            A
BX418564.2  71    .........................................................M-.............            1
AL567209.3  30    ..................-....-..-...-...                                                  1
BX440470.1  72    ........................................................................            1
BX420340.1  66    ............................................................R.....                  1
```

Query= N12-7 [organism=Homo sapiens] eef1a1

Length=1279

```
                                                                  Score        E     Max
Sequences producing significant alignments:                      (Bits)     Value  ident

BY794942.2 BY794942 Homo sapiens eye Homo sapiens cDNA clone H...  1905       0.0    99%
DC527884.1 DC527884 chimpanzee skin cDNA library PstA Pan trog...  1897       0.0    99%
BX440470.1 BX440470 Homo sapiens FETAL BRAIN Homo sapiens cDNA...  1879       0.0    99%
```

```
BX442182.2 BX442182 Homo sapiens FETAL BRAIN Homo sapiens cDNA...  1873       0.0    99%
BX418589.2 BX418589 Homo sapiens FETAL BRAIN Homo sapiens cDNA...  1873       0.0    99%
BX418564.2 BX418564 Homo sapiens FETAL BRAIN Homo sapiens cDNA...  1868       0.0    99%
BX420340.1 BX420340 Homo sapiens FETAL BRAIN Homo sapiens cDNA...  1866       0.0    99%
BX459625.2 BX459625 Homo sapiens FETAL BRAIN Homo sapiens cDNA...  1860       0.0    99%
AL517685.3 AL517685 Homo sapiens NEUROBLASTOMA Homo sapiens cD...  1845       0.0    98%
BX440303.2 BX440303 Homo sapiens FETAL BRAIN Homo sapiens cDNA...  1845       0.0    98%

ALIGNMENTS

Query       2     TTTTTCGCAACGGGTTTGCCGCCAGAACACAGGTGTCGTGAAAACTACCCCTAAAAGCCAAAATGGGAAAGGAAAAGACTCATATCAACATTGTCGTCATTGGACACGTAGATTCGGGCAAGTCCACCACTACTGGCCATCTGATCTATA  151
BY794942.2  25                       ...................................................................................................................................  155
DC527884.1  9     ......................................................................................................................................................  158

Query       152   AATGCGGTGGCATCGACAAAAGAACCATTGAAAAATTTGAGAAGGAGCTGCTGAGATGGGAAAGGGCTCCTTCAAGTATGCCTTGGTTCAAGGGATGGAAAGTCACCCGTAAGGATGGCAATGCCAGTGGAACCACGCTGCTTGAGGCTC  301
BY794942.2  689                                                                                .........................................................................  761
BY794942.2  156   ...................................................................................G...                                                                243
                                                                \
                                                                |
                                                                G
DC527884.1  692                                                                                .........................................................................  764
DC527884.1  159   ...................................................................................G...                                                                246
                                                                \
                                                                |
                                                                G
BX440470.1  1049                                                                               .......K.................................................................  977
BX442182.2  1041                                                                               .........................................................................  969
BX418589.2  1048                                                                               ........K.C..............................................................  976
BX418564.2  1046                                                                               ....................-....................................................  975
BX420340.1  1042                                                                               .........................................................................  970
BX459625.2  1036                                                                               ........K.A..............................................................  964
AL517685.3  2                                                                                  .........................................................................  74
BX440303.2  1044                                                                               .....-.K.........R.........M............-.............N............R.....  974

Query       302   TGGACTGCATCCTACCACCAACTCGTCCAACTGACAAGCCCTTGCGCCTGCCTCTCCAGGATGTCTACAAAATTGGTGGTATTGGTACTGTTCCTGTTGGCCGAGTGGAGACTGGTGTTCTCAAACCCGGTATGGTGGTCACCTTTGCTC  451
BY794942.2  762   ......................................................................................................................................................  911
DC527884.1  765   ......................................................................................................................................................  914
BX440470.1  976   ..R...................................................................................................................................................  827
BX442182.2  968   .............................................................................................................R........................................  819
BX418589.2  975   ......................................................................................................................................................  826
BX418564.2  974   .........................................C............................................................................................................  825
BX420340.1  969   ..A.............................R.....................................................................................................................  820
BX459625.2  963   ......................................................................................................................................................  814
AL517685.3  75    ......................................................................................................................................................  224
BX440303.2  973   ..-...................................................................................................................................................  825

Query       452   CAGTCAACGTTACAACGGAAGTAAAATCTGTCGAAATGCACCATGAAGCTTTGAGTGAAGCTCTTCCTGGGGACAATGTGGGCTTCAATGTCAAGAATGTGTCTGTCAAGGATGTTCGTCGTGGCAACGTTGCTGGTGACAGCAAAAATG  601
BY794942.2  912   ......................................................................................................................................................  1061
DC527884.1  915   ......................................................................................................................................................  1064
BX440470.1  826   ......................................................................................................................................................  677
BX442182.2  818   ......................................................................................................................................................  669
BX418589.2  825   ......................................................................................................................................................  676
BX418564.2  824   ..........................................................................................................C...........................................  675
BX420340.1  819   ......................................................................................................................................................  670
BX459625.2  813   ......................................................................................................................................................  664
AL517685.3  225   ......................................................................................................................................................  374
BX440303.2  824   ............................................................................................................................................C.........  675

Query       602   ACCCACCAATGGAAGCAGCTGGCTTCACTGCTCAGGTGATTATCCTGAACCATCCAGGCCAAATAAGCGCCGGCTATGCCCTGTATTGGATTGCCACACGGCTCACATTGCATGCAAGTTTGCTGAGCTGAAGGAAAAGATTGATCGCCG  751
BY794942.2  1062  ......................................................................................................................................................  1213
                                                                       \                        \
                                                                       |                        |
                                                                       G                        C
DC527884.1  1065  ......................................................................................................................................................  1215
                                                                                                \
                                                                                                |
                                                                                                C
BX440470.1  676   ......................................................................................................................................................  525
                                                                                                   \                                                         \
                                                                                                   |                                                         |
                                                                                                   C                                                         C
BX442182.2  668   ......................................................................................................................................................  518
                                                                                                   \
                                                                                                   |
                                                                                                   C
BX418589.2  675   ................................................................................................................................................Y.S...  525
                                                                                                   \
                                                                                                   |
                                                                                                   C
BX418564.2  674   .........................................................................................................................................M............  524
                                                                                                   \
                                                                                                   |
```

```
                                                                                                   C
BX420340.1  669   ......................................................................................................................................................  519
                                                                                                   \
                                                                                                   |
                                                                                                   C
BX459625.2  663   ......................................................................................................................................................  513
                                                                                                   \
                                                                                                   |
                                                                                                   C
AL517685.3  375   ......................................................................................................................................................  525
                                                                                                \
                                                                                                |
                                                                                                C
BX440303.2  674   ......................................................................................................................................................  524
                                                                                                   \
                                                                                                   |
                                                                                                   C

Query       752   TTCTGGTAAAAAGCTGGAAGATGGCCCTAAATTCTTGAAGTCTGGTGATGCTGCCATTGTTGATATGGTTCCTGGCAAGCCATGTGTGTTGAGAGCTTCTCAGACTATCCACCTTTGGGTCGCTTTGCTGTTCGTGATATGAGACAGACA  901
BY794942.2  1214  ......................................................................................................................................................  1364
                                                                                                 \
                                                                                                 |
                                                                                                 C
DC527884.1  1216  ......................................................................................................................................................  1366
                                                                                                 \
                                                                                                 |
                                                                                                 C
BX440470.1  524   ......................................................................................................................................................  374
                                                                                                   \
                                                                                                   |
                                                                                                   C
BX442182.2  517   ......................................................................................................................................................  367
                                                                                                   \
                                                                                                   |
                                                                                                   C
BX418589.2  524   ......................................................................................................................................................  374
                                                                                                   \
                                                                                                   |
                                                                                                   C
BX418564.2  523   ......................................................................................................................................................  373
                                                                                                   \
                                                                                                   |
                                                                                                   C
BX420340.1  518   ......................................................................................................................................................  368
                                                                                                   \
                                                                                                   |
                                                                                                   C
BX459625.2  512   ......................................................................................................................................................  362
                                                                                                   \
                                                                                                   |
                                                                                                   C
AL517685.3  526   ................................................................................................................................................M.....  676
                                                                                                 \
                                                                                                 |
                                                                                                 C
BX440303.2  523   ......................................................................................................................................................  373
                                                                                                   \
                                                                                                   |
                                                                                                   C

Query       902   GTTGCGGTGGGTGTCATCAAAGCAGTGGACAAGAAGGCTGCTGGAGCTGGCAAGGTCACCAAGTCTGCCAGAAAGCTCAGAAGGCTAAATGAATATTATCCCTAATACCTGCCACCCACTCTTAATCAGTGGTGGAAGAACGGTCTCAGA  1051
BY794942.2  1365  ......................................................................................................................................................  1516
                                                                                     \                                              \
                                                                                     |                                              |
                                                                                     C                                              C
DC527884.1  1367  ............A.............................................................................................................C...........................  1518
                                                                                     \                                              \
                                                                                     |                                              |
                                                                                     C                                              C
BX440470.1  373   ......................................................................................................................................................  222
                                                                                       \                                               \
                                                                                       |                                               |
                                                                                       C                                               C
BX442182.2  366   ......................................................................................................................................................  215
                                                                                       \                                               \
                                                                                       |                                               |
                                                                                       C                                               C
BX418589.2  373   ......................................................................................................................................................  222
                                                                                       \                                               \
                                                                                       |                                               |
                                                                                       C                                               C
BX418564.2  372   ......................................................................................................................................................  221
```

```
                                                                                       \                                               \
                                                                                       |                                               |
                                                                                       C                                               C
BX420340.1  367   ......................................................................................................................................................  216
                                                                                       \                                               \
                                                                                       |                                               |
                                                                                       C                                               C
BX459625.2  361   ......................................................................................................................................................  210
                                                                                       \                                               \
                                                                                       |                                               |
                                                                                       C                                               C
AL517685.3  677   ..............Y................................Y......................................................................................................  828
                                                                                     \                                              \
                                                                                     |                                              |
                                                                                     C                                              C
BX440303.2  372   ......................................................................................................................................................  221
                                                                                       \                                               \
                                                                                       |                                               |
                                                                                       C                                               C

Query       1052  ACTGTTTGTTTCAATTGGCCATTTAAGTTTAGTAGTAAAAGACTGGTTAATGATAACAATGCATCGTAAAACCTTCAGAAGGAAAGGAGAATGTTTTGTGGACCACtttggttttctttttgcgtgtggcagttttaagttattagtttt  1201
BY794942.2  1517  ......................................................................................................................................................  1667
                                                                                                                                      \
                                                                                                                                      |
                                                                                                                                      T
DC527884.1  1519  ......................................................................................................................................................  1669
                                                                                                                                      \
                                                                                                                                      |
                                                                                                                                      T
BX440470.1  221   ......................................................................................................................................................  71
                                                                                                                                           \
                                                                                                                                           |
                                                                                                                                           T
BX442182.2  214   ......................................................................................................................................................  64
                                                                                                                                           \
                                                                                                                                           |
                                                                                                                                           T
BX418589.2  221   ......................................................................................................................................................  71
                                                                                                                                           \
                                                                                                                                           |
                                                                                                                                           T
BX418564.2  220   ......................................................................................................................................................  70
                                                                                                                                           \
                                                                                                                                           |
                                                                                                                                           T
BX420340.1  215   ......................................................................................................................................................  65
                                                                                                                                           \
                                                                                                                                           |
                                                                                                                                           T
BX459625.2  209   ......................................................................................................................................................  59
                                                                                                                                           \
                                                                                                                                           |
                                                                                                                                           T
AL517685.3  829   ......................................................................................................-...............................................  979
                                                                                                                                           \    \
                                                                                                                                           |    |
                                                                                                                                           T    T
BX440303.2  220   ......................................................................................................................................................  70
                                                                                                                                           \
                                                                                                                                           |
                                                                                                                                           T

Query       1202  taaaatcagtactttttaATGGAAACAACTTGACCAAAATTTGTCACAGAATTTGAGACCCATTAAAAAAGTTAAATG  1279
BY794942.2  1668  ..............................................................................  1747
                                                     \               \
                                                     |               |
                                                     A               T
DC527884.1  1670  ..............................................................................  1749
                                                     \               \
                                                     |               |
                                                     A               T
BX440470.1  70    ....................................................................            1
                                                         \              \
                                                         |              |
                                                         A              T
BX442182.2  63    .............................................................                   1
                                                         \              \
                                                         |              |
                                                         A              T
BX418589.2  70    ....................................................................            1
                                                         \              \
                                                         |              |
```

```
                                                         A              D
BX418564.2  69    ......................................................M.............            1
                                                         \
                                                         |
                                                         A
BX420340.1  64    .........................................................R.....                 1
                                                         \              \
                                                         |              |
                                                         A              T
BX459625.2  58    ......................................................                          4
                                                         \
                                                         |
                                                         A
AL517685.3  980   ....W-..................M-....K.............Y...............-....D...-..-.....  1052
BX440303.2  69    ......................................................T.............            1
                                                         \
                                                         |
                                                         A

Query= N13-10 [organism=Homo sapiens] eef1a1

Length=1650


                                                                   Score        E     Max
Sequences producing significant alignments:                       (Bits)     Value  ident

DC855699.1 DC855699 macaque spleen cDNA library QspA Macaca fa...  1519       0.0     98%
DC527884.1 DC527884 chimpanzee skin cDNA library PstA Pan trog...  1495       0.0     97%
BY794942.2 BY794942 Homo sapiens eye Homo sapiens cDNA clone H...  1465       0.0     97%
CR996969.1 CR996969 RZPD no.9017 Homo sapiens cDNA clone RZPDp...  1410       0.0    100%
CR996534.1 CR996534 RZPD no.9017 Homo sapiens cDNA clone RZPDp...  1410       0.0    100%
CR982215.1 CR982215 RZPD no.9016 Homo sapiens cDNA clone RZPDp...  1410       0.0    100%
BX453401.2 BX453401 Homo sapiens T CELLS (JURKAT CELL LINE) Ho...  1410       0.0    100%
BX443032.2 BX443032 Homo sapiens B CELLS (RAMOS CELL LINE) Hom...  1410       0.0    100%
BX421307.2 BX421307 Homo sapiens B CELLS (RAMOS CELL LINE) Hom...  1410       0.0    100%
BX353545.2 BX353545 Homo sapiens NEUROBLASTOMA COT 25-NORMALIZ...  1410       0.0    100%

ALIGNMENTS

Query       2     TTTTTCGCAACGGGTTTGCCGCCAGAACACAGGTGTCGTGAAAACTACCCCTAAAAGCCAAAATGGGAAAGGAAAAGACTCATATCAACATTGTCGTCATTGGACACGTAGATTCGGGCAAGTCCACCACTACTGGCCATCTGATCTATA  151
DC855699.1  1        .....-...........................................................................................................................................C.  146
DC527884.1  9     ......................................................................................................................................................  158
BY794942.2  25                       ...................................................................................................................................  155

Query       152   AATGCGGTGGCATCGACAAAAGAACCATTGAAAAATTTGAGAAGGAGGCTGCTGAGATGGGAAAGGGCTCCTTCAAGTATGCCTGGGTCTTGGATAAACTGAAAGCTGAGCGTGAACGTGGTATCACCATTGATATCTCCTTGTGGAAAT  301
DC855699.1  147   ................................................................................................................G.....................................  296
DC527884.1  159   ............................................................................................................................T.........................  308
BY794942.2  156   ......................................................................................................................................................  305

Query       302   TTGAGACCAGCAAGTACTATGTGACTATCAAAAACATGATTACAGGGACATCTCAGGCTGACTGTGCTGTCCTGATTGTTGCTGCTGGTGTTGGTGAATTTGAAGCTGGTATCTCCAAGAATGGGCAGACCCGAGAGCATGCCCTTCTGG  451
DC855699.1  297   ................T..............CCC............................................................................................................T.......  451
                                                \\
                                                ||
                                              TTG|
                                                 |
                                                TG
DC527884.1  309   ......................................................................................................................................................  488
                                            \
                                            |
                  ATCATTGATGCCCCAGGACACAGAGACTTC
BY794942.2  306   ......................................................................................................................................................  485
                                           \
                                           |
                  TATCATTGATGCCCCAGGACACAGAGACTT

Query       452   CTTACACACTGGGTGTGAAACAACTAATTGTCGGTGTTAACAAAATGGATTCCACTGAGCCACCCTACAGCCAGAAGAGATATGAGGAAATTGTTAAGGAAGTCAGCACTTACATTAAGAAAATTGGCTACAACCCCGACACAGTAGCAT  601
DC855699.1  452   ...............................T..................................................C...................................................................  601
DC527884.1  489   ......................................................................................................................................................  638
BY794942.2  486   ......................................................................................................................................................  635

Query       602   TTGTGCCAATTTCTGGTTGGAATGGTGACAACATGCTGGAGCCAAGTGCTAACATGCCTTGGTTCAAGGGATGGAAAGTCACCGTAAGGATGGCAATGCCAGTGGAACCACGCTGCTTGAGGCTCTGGACTGCATCCTACCACCAACTCG  751
DC855699.1  602   ............................................................................G.........................................................................  752
                                                                                                   \
                                                                                                   |
                                                                                                   C
DC527884.1  639   ......................................................................................................................................................  789
                                                                                                   \
                                                                                                   |
                                                                                                   C
BY794942.2  636   ......................................................................................................................................................  786
                                                                                                   \
```

```
                                                                                                   |
                                                                                                   C

Query       752   TCCAACTGACAAGCCCTTGCGCCTGCCTCTCCAGGATGTCTACAAAATTGGTGGTATTGGTACTGTTCCTGTTGGCCGAGTGGAGACTGGTGTTCTCAAACCCGGTATGGTGGTCACCTTTGCTCCAGTCAACGTTACAATGTGGGCTTC  901
DC855699.1  753   ............................................................................................................................  876
DC527884.1  987                                                                                                                                           ..............  1000
DC527884.1  790   ............................................................................................................................................  929
BY794942.2  984                                                                                                                                           ..............  997
BY794942.2  787   ............................................................................................................................................  926
CR996969.1  17                                                                                                                                            ..............  30
CR996534.1  86                                                                                                                                            ..............  99
CR996534.1  1                                                                                                                       ............................  28
CR982215.1  29                                                                                                                                            ..............  42
BX453401.2  40                                                                                                                                            ..............  53
BX443032.2  166                                                                                                                                           ..............  179
BX443032.2  1                                     ............................................................................................................  108
BX421307.2  140                                                                                                                                           ..............  153
BX421307.2  23                                                                                     ............................................................  82
                                                                                                      \
                                                                                                      |
                                                                                                      T
BX353545.2  45                                                                                                                                            ..............  58

Query       902   AATGTCAAGAATGTGTCTGTCAAGGATGTTCGTCGTGGCAACGTTGCTGGTGACAGCAAAAATGACCCACCAATGGAAGCAGCTGGCTTCACTGCTCAGGTGATTATCCTGAACCATCCAGGCCAAATAAGCGCCGGCTATGCCCCTGTA  1051
DC527884.1  1001  ......................................................................................................................................................  1150
BY794942.2  998   ......................................................................................................................................................  1148
                                                                                                                                       \
                                                                                                                                       |
                                                                                                                                       G
CR996969.1  31    ......................................................................................................................................................  180
CR996534.1  100   ......................................................................................................................................................  249
CR982215.1  43    ......................................................................................................................................................  192
BX453401.2  54    ......................................................................................................................................................  203
BX443032.2  180   ......................................................................................................................................................  329
BX421307.2  154   ......................................................................................................................................................  303
BX353545.2  59    ......................................................................................................................................................  208

Query       1052  TTGGATTGCCACACGGCTCACATTGCATGCAAGTTTGCTGAGCTGAAGGAAAAGATTGATCGCCGTTCTGGTAAAAAGCTGGAAGATGGCCCTAAATTCTTGAAGTCTGGTGATGCTGCCATTGTTGATATGGTTCCTGGCAAGCCCATG  1201
DC527884.1  1151  ......................................................................................................................................................  1300
BY794942.2  1149  ......................................................................................................................................................  1298
CR996969.1  181   ......................................................................................................................................................  330
CR996534.1  250   ......................................................................................................................................................  399
CR982215.1  193   ......................................................................................................................................................  342
BX453401.2  204   ......................................................................................................................................................  353
BX443032.2  330   ......................................................................................................................................................  479
BX421307.2  304   ......................................................................................................................................................  453
BX353545.2  209   ......................................................................................................................................................  358

Query       1202  TGTGTTGAGAGCTTCTCAGACTATCCACCTTTGGGTCGCTTTGCTGTTCGTGATATGAGACAGACAGTTGCGGTGGGTGTCATCAAAGCAGTGGACAAGAAGGCTGCTGGAGCTGGCAAGGTCACCAAGTCTGCCCAGAAAGCTCAGAAG  1351
DC527884.1  1301  ..............................................................................A.......................................................................  1450
BY794942.2  1299  ......................................................................................................................................................  1448
CR996969.1  331   ......................................................................................................................................................  480
CR996534.1  400   ......................................................................................................................................................  549
CR982215.1  343   ......................................................................................................................................................  492
BX453401.2  354   ......................................................................................................................................................  503
BX443032.2  480   ......................................................................................................................................................  629
BX421307.2  454   ......................................................................................................................................................  603
BX353545.2  359   ......................................................................................................................................................  508

Query       1352  GCTAAATGAATATTATCCCTAATACCTGCCACCCCACTCTTAATCAGTGGTGGAAGAACGGTCTCAGAACTGTTTGTTTCAATTGGCCATTTAAGTTTAGTAGTAAAAGACTGGTTAATGATAACAATGCATCGTAAAACCTTCAGAAGG  1501
DC527884.1  1451  ........................................C.............................................................................................................  1600
BY794942.2  1449  ......................................................................................................................................................  1598
CR996969.1  481   ......................................................................................................................................................  630
CR996534.1  550   ......................................................................................................................................................  699
CR982215.1  493   ......................................................................................................................................................  642
BX453401.2  504   ......................................................................................................................................................  653
BX443032.2  630   ......................................................................................................................................................  779
BX421307.2  604   ......................................................................................................................................................  753
BX353545.2  509   ......................................................................................................................................................  658

Query       1502  AAAGGAGAATgttttgtggaccactttggttttctttttttgcgtgtggcagttttaagttattagtttttaaaatcagtactttttaATGGAAACAACTTGACCAAAAATTTGTCACAGAATTTTGAGACCCATTAAAAAAGTTAAATG  1650
DC527884.1  1601  .....................................................................................................................................................  1749
BY794942.2  1599  .....................................................................................................................................................  1747
CR996969.1  631   .....................................................................................................................................................  779
CR996534.1  700   .....................................................................................................................................................  848
CR982215.1  643   .....................................................................................................................................................  791
BX453401.2  654   .....................................................................................................................................................  802
BX443032.2  780   .....................................................................................................................................................  928
BX421307.2  754   .....................................................................................................................................................  902
BX353545.2  659   .....................................................................................................................................................  807

Query= N14-17 [organism=Homo sapiens] eef1a1
```

```
Length=1118


                                                                  Score        E     Max
Sequences producing significant alignments:                      (Bits)     Value  ident

BU845975.1 AGENCOURT_10413042 NIH_MGC_109 Homo sapiens cDNA cl...  1592       0.0    100%
BX421307.2 BX421307 Homo sapiens B CELLS (RAMOS CELL LINE) Hom...  1591       0.0    100%
BX443032.2 BX443032 Homo sapiens B CELLS (RAMOS CELL LINE) Hom...  1587       0.0    100%
BY794942.2 BY794942 Homo sapiens eye Homo sapiens cDNA clone H...  1585       0.0    100%
BU192437.1 AGENCOURT_7938567 NIH_MGC_92 Homo sapiens cDNA clon...  1585       0.0    100%
BX417098.2 BX417098 Homo sapiens PLACENTA Homo sapiens cDNA cl...  1583       0.0    100%
DC527884.1 DC527884 chimpanzee skin cDNA library PstA Pan trog...  1580       0.0    100%
BX441194.2 BX441194 Homo sapiens FETAL BRAIN Homo sapiens cDNA...  1580       0.0    100%
BQ049312.1 AGENCOURT_6795094 NIH_MGC_85 Homo sapiens cDNA clon...  1580       0.0    100%
BM477966.1 AGENCOURT_6482916 NIH_MGC_85 Homo sapiens cDNA clon...  1580       0.0    100%

ALIGNMENTS

Query       2     TTTTTCGCAACGGGTTTGCCGCCAGAACACAGGTGTCGTGAAAACTACCCCTAAAAAGCCAAAATGGGAAAGGAAAAGACTCATATCAACATTGTCGTCATTGGACACGTAGATTCGGGCAAGTCCACCACTACTGGCCATCTGATCTAT  151
BY794942.2  25                       .................................-.................................................................................................  154
DC527884.1  9     ....................................................-.................................................................................................  157

Query       152   AAATGCGGTGGCATCGACAAAAGAACCATTGAAAAATTTGAGAAGGAGGCTGCTGAGATGGGAAAGGGCTCCTTCAAGTATGCCTGGGTCTTGGATAAACTCAAAACCCGGTATGGTGGTCACCTTTGCTCCAGTCAACGTTACAACGGA  301
BU845975.1  867                                                                                                      ..........................N........................  817
BX421307.2  37                                                                                                       ...-...............................................  86
BX443032.2  63                                                                                                       ...-...............................................  112
BY794942.2  881                                                                                                      ...-...............................................  930
BY794942.2  155   .....................................................................................................G...                                              259
BU192437.1  8                                                                                                        ...-...............................................  57
BX417098.2  26                                                                                                       ...-...............................................  75
DC527884.1  884                                                                                                      ...-...............................................  933
DC527884.1  158   .....................................................................................................G...                                              262
BX441194.2  75                                                                                                       ...-.............W.................................  124
BQ049312.1  35                                                                                                       ...-...............................................  84
BM477966.1  19                                                                                                       ...-...............................................  68

Query       302   AGTAAAATCTGTCGAAATGCACCATGAAGCTTTGAGTGAAGCTCTTCCTGGGGACAATGTGGGCTTCAATGTCAAGAATGTGTCTGTCAAGGATGTTCGTCGTGGCAACGTTGCTGGTGACAGCAAAAATGACCCACCAATGGAAGCAGC  451
BU845975.1  816   ......................................................................................................................................................  667
BX421307.2  87    ......................................................................................................................................................  236
BX443032.2  113   ...M..................................................................................................................................................  262
BY794942.2  931   ......................................................................................................................................................  1080
BU192437.1  58    ......................................................................................................................................................  207
BX417098.2  76    ......................................................................................................................................................  225
DC527884.1  934   ......................................................................................................................................................  1083
BX441194.2  125   ......................................................................................................................................................  274
BQ049312.1  85    ......................................................................................................................................................  234
BM477966.1  69    ......................................................................................................................................................  218

Query       452   TGGCTTCACTGCTCAGGTGATTATCCTGAACCATCCAGGCCAAATAAGCGCCGGCTATGCCCCTGTATTGGATTGCCACACGGCTCACATTGCATGCAAGTTTGCTGAGCTGAAGGAAAAGATTGATCGCCGTTCTGGTAAAAAGCTGGA  601
BU845975.1  666   ......................................................................................................................................................  517
BX421307.2  237   ......................................................................................................................................................  386
BX443032.2  263   ......................................................................................................................................................  412
BY794942.2  1081  ......................................................................................................................................................  1231
                                                    \
                                                    |
                                                    G
BU192437.1  208   ......................................................................................................................................................  357
BX417098.2  226   ......................................................................................................................................................  375
DC527884.1  1084  ......................................................................................................................................................  1233
BX441194.2  275   ......................................................................................................................................................  424
BQ049312.1  235   ......................................................................................................................................................  384
BM477966.1  219   ......................................................................................................................................................  368

Query       602   AGATGGCCCTAAATTCTTGAAGTCTGGTGATGCTGCCATTGTTGATATGGTTCCTGGCAAGCCCATGTGTGTTGAGAGCTTCTCAGACTATCCACCTTTGGGTCGCTTTGCTGTTCGTGATATGAGACAGACAGTTGCGGTGGGTGTCAT  751
BU845975.1  516   ......................................................................................................................................................  367
BX421307.2  387   ......................................................................................................................................................  536
BX443032.2  413   ......................................................................................................................................................  562
BY794942.2  1232  ......................................................................................................................................................  1381
BU192437.1  358   ......................................................................................................................................................  507
BX417098.2  376   ......................................................................................................................................................  525
DC527884.1  1234  .................................................................................................................................................A....  1383
BX441194.2  425   ......................................................................................................................................................  574
BQ049312.1  385   ......................................................................................................................................................  534
BM477966.1  369   ......................................................................................................................................................  518

Query       752   CAAAGCAGTGGACAAGAAGGCTGCTGGAGCTGGCAAGGTCACCAAGTCTGCCCAGAAAGCTCAGAAGGCTAAATGAATATTATCCCTAATACCTGCCACCCCACTCTTAATCAGTGGTGGAAGAACGGTCTCAGAACTGTTTGTTTCAAT  901
BU845975.1  366   ......................................................................................................................................................  217
BX421307.2  537   ......................................................................................................................................................  686
BX443032.2  563   ......................................................................................................................................................  712
BY794942.2  1382  ......................................................................................................................................................  1531
```

```
BU192437.1  508   ......................................................................................................................................................  657
BX417098.2  526   ......................................................................................................................................................  675
DC527884.1  1384  ...........................................................................................................C..........................................  1533
BX441194.2  575   ......................................................................................................................................................  724
BQ049312.1  535   ......................................................................................................................................................  684
BM477966.1  519   ......................................................................................................................................................  668

Query       902   TGGCCATTTAAGTTTAGTAGTAAAAGACTGGTTAATGATAACAATGCATCGTAAAACCTTCAGAAGGAAAGGAGAATgttttgtggaccactttggttttcttttttgcgtgtggcagttttaagttattagtttttaaaatcagtactt  1051
BU845975.1  216   ......................................................................................................................................................  67
BX421307.2  687   ......................................................................................................................................................  836
BX443032.2  713   ......................................................................................................................................................  862
BY794942.2  1532  ......................................................................................................................................................  1681
BU192437.1  658   ........................................................A.............................................................................................  807
BX417098.2  676   ......................................................................................................................................................  825
DC527884.1  1534  ......................................................................................................................................................  1683
BX441194.2  725   ......................................................................................................................................................  874
BQ049312.1  685   ......................................................................................................................................................  834
BM477966.1  669   .........................................................................................................................................-............  817

Query       1052  tttaATGGAAACAACTTGACCAAAAATTTGTCACAGAATTTTGAGACCCATTaaaaaaaGTTAAATG  1118
BU845975.1  66    ..........................................................-........  1
BX421307.2  837   ..........................................................-........  902
BX443032.2  863   ..........................................................-........  928
BY794942.2  1682  ..........................................................-........  1747
BU192437.1  808   .................................................C.................  874
BX417098.2  826   ................................................-.........-........  890
DC527884.1  1684  ..........................................................-........  1749
BX441194.2  875   ................................................-.........-........  939
BQ049312.1  835   ..........................................................-...T....  901
                                                               \
                                                               |
                                                               A
BM477966.1  818   .....................................................N.....-.......  883

Query= N15-19 [organism=Homo sapiens] eef1a1

Length=933


                                                                 Score        E     Max
Sequences producing significant alignments:                      (Bits)     Value  ident

DC635691.1 DC635691 macaque bone marrow cDNA library QbmA Maca...  1550       0.0    97%
BQ005659.1 UI-H-ED0-ayr-i-05-0-UI.s1 NCI_CGAP_ED0 Homo sapiens...  1153       0.0    100%
BM997474.1 UI-H-DH0-auq-l-16-0-UI.s1 NCI_CGAP_DH0 Homo sapiens...  1153       0.0    100%
CT005089.1 CT005089 RZPD no.9017 Homo sapiens cDNA clone RZPDp...  1151       0.0    100%
CR999820.1 CR999820 RZPD no.9017 Homo sapiens cDNA clone RZPDp...  1151       0.0    100%
CR999128.1 CR999128 RZPD no.9017 Homo sapiens cDNA clone RZPDp...  1151       0.0    100%
CR996969.1 CR996969 RZPD no.9017 Homo sapiens cDNA clone RZPDp...  1151       0.0    100%
CR996534.1 CR996534 RZPD no.9017 Homo sapiens cDNA clone RZPDp...  1151       0.0    100%
CR989971.1 CR989971 RZPD no.9017 Homo sapiens cDNA clone RZPDp...  1151       0.0    100%
CR987376.1 CR987376 RZPD no.9017 Homo sapiens cDNA clone RZPDp...  1151       0.0    100%

ALIGNMENTS

Query       1     CTTTTTCGCAACGGGTTTGCCGCCAGAACACAGGTGTCGTGAAAACTACCCCTAAAAGCCAAAATGGGAAAGGAAAAGACTCATATCAACATTGTCGTCATTGGACACGTAGATTCGGGCAAGTCCACCACTACTGGCCATCTGATCTAT  150
DC635691.1  1     .........-...........................................................................................................................................C  149

Query       151   AAATGCGGTGGCATCGACAAAAGAACCATTGAAAAATTTGAGAAGGAGGCTGCTGAGATGGGAAAGGGCTCCTTCAAGTATGCCTGGGTCTTGGATAAACTGAAAGCTGAGCGTGAACGTGGTATCACCATTGATATCTCCTTGTGGAAA  300
DC635691.1  150   .................................................................................................................G....................................  299

Query       301   TTTGAGACCAAATAAGCGCCTGCTATGCCCCTGTATTGGATTGCCACACGGCTCACATTGCATGCAAGTTTGCTGAGCTGAAGGAAAAGATTGATCGCCGTTCTGGTAAAAAGCTGGAAGATGGCCCTAAATTCTTGAAGTCTGGTGATG  450
DC635691.1  300   ................T.A..T............G..............A..............................................................A.....................................  459
                            \ \ \\     \ \\
                            | | ||     | ||
                           GC G TT     A AT
                                 |        |
                                 T        G
BQ005659.1  648       ..N.............G.................................................................................................................................  503
BM997474.1  648       ..N.............G.................................................................................................................................  503
CT005089.1  45        ..G.............G.................................................................................................................................  190
CR999820.1  3         ..G.............G.................................................................................................................................  148
CR999128.1  117       ..G.............G.................................................................................................................................  262
CR996969.1  150       ..G.............G.................................................................................................................................  295
CR996534.1  219       ..G.............G.................................................................................................................................  364
CR989971.1  75        ..G.............G.................................................................................................................................  220
CR987376.1  12        ..G.............G.................................................................................................................................  157

Query       451   CTGCCATTGTTGATATGGTTCCTGGCAAGCCCATGTGTGTTGAGAGCTTCTCAGACTATCCACCTTTGGGTCGCTTTGCTGTTCGTGATATGAGACAGACAGTTGCGGTGGGTGTCATCAAAGCAGTGGACAAGAAGGCTGCTGGAGCTG  600
DC635691.1  460   ...............................T..............................................................G.......................................................  609
BQ005659.1  502   ......................................................................................................................................................  353
BM997474.1  502   ......................................................................................................................................................  353
```

```
CT005089.1  191  ......................................................................................................................................................  340
CR999820.1  149  ......................................................................................................................................................  298
CR999128.1  263  ......................................................................................................................................................  412
CR996969.1  296  ......................................................................................................................................................  445
CR996534.1  365  ......................................................................................................................................................  514
CR989971.1  221  ......................................................................................................................................................  370
CR987376.1  158  ......................................................................................................................................................  307

Query       601  GCAAGGTCACCAAGTCTGCCCAGAAAGCTCAGAAGGCTAAATGAATATTATCCCTAATACCTGCCACCCCACTCTTAATCAGTGGTGGAAGAACGGTCTCAGAACTGTTTGTTTCAATTGGCCATTTAAGTTTAGTAGTAAAAGACTGGT  750
DC635691.1  610  ......................................................................................................................................................  759
BQ005659.1  352  ......................................................................................................................................................  203
BM997474.1  352  ......................................................................................................................................................  203
CT005089.1  341  ......................................................................................................................................................  490
CR999820.1  299  ......................................................................................................................................................  448
CR999128.1  413  ......................................................................................................................................................  562
CR996969.1  446  ......................................................................................................................................................  595
CR996534.1  515  ......................................................................................................................................................  664
CR989971.1  371  ......................................................................................................................................................  520
CR987376.1  308  ......................................................................................................................................................  457

Query       751  TAATGATAACAATGCATCGTAAAACCTTCAGAAGGAAAGGAGAATgttttgtggaccactttggttttctttttttgcgtgtggcagttttaagttattagtttttaaaatcagtactttttaATGGAAACAACTTGACCAAAAATTTGTC  900
DC635691.1  760  .................................-....................................................-.................--.....................................-......  904
BQ005659.1  202  ......................................................................................................................................................  53
BM997474.1  202  ......................................................................................................................................................  53
CT005089.1  491  ......................................................................................................................................................  640
CR999820.1  449  ......................................................................................................................................................  598
CR999128.1  563  ......................................................................................................................................................  712
CR996969.1  596  ......................................................................................................................................................  745
CR996534.1  665  ......................................................................................................................................................  814
CR989971.1  521  ......................................................................................................................................................  670
CR987376.1  458  ......................................................................................................................................................  607

Query       901  ACAGAATTTTGAGACCCATTAAAAAAGTTAAAT  933
DC635691.1  905  .....-...-.........-.....-...      929
BQ005659.1  52   .................................  20
BM997474.1  52   .................................  20
CT005089.1  641  .................................  673
CR999820.1  599  .................................  631
CR999128.1  713  .................................  745
CR996969.1  746  .................................  778
CR996534.1  815  .................................  847
CR989971.1  671  .................................  703
CR987376.1  608  .................................  640

Query= N16-18 [organism=Homo sapiens] eef1a1

Length=970


                                                                    Score        E     Max
Sequences producing significant alignments:                        (Bits)     Value  ident

CR999128.1 CR999128 RZPD no.9017 Homo sapiens cDNA clone RZPDp...  1247       0.0    100%
CR996969.1 CR996969 RZPD no.9017 Homo sapiens cDNA clone RZPDp...  1247       0.0    100%
CR996534.1 CR996534 RZPD no.9017 Homo sapiens cDNA clone RZPDp...  1247       0.0    100%
CR989971.1 CR989971 RZPD no.9017 Homo sapiens cDNA clone RZPDp...  1247       0.0    100%
CR982215.1 CR982215 RZPD no.9016 Homo sapiens cDNA clone RZPDp...  1247       0.0    100%
CR977084.1 CR977084 RZPD no.9016 Homo sapiens cDNA clone RZPDp...  1247       0.0    100%
BX453401.2 BX453401 Homo sapiens T CELLS (JURKAT CELL LINE) Ho...  1247       0.0    100%
BX453399.2 BX453399 Homo sapiens T CELLS (JURKAT CELL LINE) Ho...  1247       0.0    100%
BX443032.2 BX443032 Homo sapiens B CELLS (RAMOS CELL LINE) Hom...  1247       0.0    100%
BX426531.2 BX426531 Homo sapiens T CELLS (JURKAT CELL LINE) Ho...  1247       0.0    100%

ALIGNMENTS

Query       296  TGGAAGCAGCTGGCTTCACTGCTCAGGTGATTATCCTGAACCATCCAGGCCAAATAAGCGCCGGCTATGCCCCTGTATTGGATTGCCACACGGCTCACATTGCATGCAAGTTTGCTGAGCTGAAGGAAAAGATTGATCGCCGTTCTGGTA  445
CR999128.1  71   ......................................................................................................................................................  220
CR996969.1  104  ......................................................................................................................................................  253
CR996534.1  173  ......................................................................................................................................................  322
CR989971.1  29   ......................................................................................................................................................  178
CR982215.1  116  ......................................................................................................................................................  265
CR977084.1  62   ......................................................................................................................................................  211
BX453401.2  127  ......................................................................................................................................................  276
BX453399.2  681  ......................................................................................................................................................  532
BX443032.2  253  ......................................................................................................................................................  402
BX426531.2  72   ......................................................................................................................................................  221

Query       446  AAAAGCTGGAAGATGGCCCTAAATTCTTGAAGTCTGGTGATGCTGCCATTGTTGATATGGTTCCTGGCAAGCCCATGTGTGTTGAGAGCTTCTCAGACTATCCACCTTTGGGTCGCTTTGCTGTTCGTGATATGAGACAGACAGTTGCGG  595
CR999128.1  221  ......................................................................................................................................................  370
CR996969.1  254  ......................................................................................................................................................  403
CR996534.1  323  ......................................................................................................................................................  472
CR989971.1  179  ......................................................................................................................................................  328
```

```
CR982215.1  266  ......................................................................................................................................................  415
CR977084.1  212  ......................................................................................................................................................  361
BX453401.2  277  ......................................................................................................................................................  426
BX453399.2  531  ......................................................................................................................................................  382
BX443032.2  403  ......................................................................................................................................................  552
BX426531.2  222  ......................................................................................................................................................  371

Query       596  TGGGTGTCATCAAAGCAGTGGACAAGAAGGCTGCTGGAGCTGGCAAGGTCACCAAGTCTGCCCAGAAAGCTCAGAAGGCTAAATGAATATTATCCCTAATACCTGCCACCCCACTCTTAATCAGTGGTGGAAGAACGGTCTCAGAACTGT  745
CR999128.1  371  ......................................................................................................................................................  520
CR996969.1  404  ......................................................................................................................................................  553
CR996534.1  473  ......................................................................................................................................................  622
CR989971.1  329  ......................................................................................................................................................  478
CR982215.1  416  ......................................................................................................................................................  565
CR977084.1  362  ......................................................................................................................................................  511
BX453401.2  427  ......................................................................................................................................................  576
BX453399.2  381  ......................................................................................................................................................  232
BX443032.2  553  ......................................................................................................................................................  702
BX426531.2  372  ......................................................................................................................................................  521

Query       746  TTGTTTCAATTGGCCATTTAAGTTTAGTAGTAAAAGACTGGTTAATGATAACAATGCATCGTAAAACCTTCAGAAGGAAAGGAGAATgttttgtggaccactttggttttctttttttgcgtgtggcagttttaagttattagtttttaaa  895
CR999128.1  521  ......................................................................................................................................................  670
CR996969.1  554  ......................................................................................................................................................  703
CR996534.1  623  ......................................................................................................................................................  772
CR989971.1  479  ......................................................................................................................................................  628
CR982215.1  566  ......................................................................................................................................................  715
CR977084.1  512  ......................................................................................................................................................  661
BX453401.2  577  ......................................................................................................................................................  726
BX453399.2  231  ......................................................................................................................................................  82
BX443032.2  703  ......................................................................................................................................................  852
BX426531.2  522  ......................................................................................................................................................  671

Query       896  atcagtactttttaATGGAAACAACTTGACCAAAAATTTGTCACAGAATTTTGAGACCCATTAAAAAAGTTAAAT  970
CR999128.1  671  ...........................................................................  745
CR996969.1  704  ...........................................................................  778
CR996534.1  773  ...........................................................................  847
CR989971.1  629  ...........................................................................  703
CR982215.1  716  ...........................................................................  790
CR977084.1  662  ...........................................................................  736
BX453401.2  727  ...........................................................................  801
BX453399.2  81   ...........................................................................  7
BX443032.2  853  ...........................................................................  927
BX426531.2  672  ...........................................................................  746

Query= N17-21 [organism=Homo sapiens] eef1a1

Length=1093


                                                                   Score        E     Max
Sequences producing significant alignments:                       (Bits)     Value  ident

CA442866.1 UI-H-DP0-avr-n-20-0-UI.s1 NCI_CGAP_Fs1 Homo sapiens...  1247       0.0    100%
CA417999.1 UI-H-FH0-bcc-k-24-0-UI.s1 NCI_CGAP_FH0 Homo sapiens...  1247       0.0    100%
CA414363.1 UI-H-EZ0-bas-d-03-0-UI.s1 NCI_CGAP_Ch1 Homo sapiens...  1247       0.0    100%
CX758265.1 AGENCOURT_41351531 NIH_MGC_278 Homo sapiens cDNA cl...  1245       0.0    100%
CT005089.1 CT005089 RZPD no.9017 Homo sapiens cDNA clone RZPDp...  1243       0.0    100%
CR999128.1 CR999128 RZPD no.9017 Homo sapiens cDNA clone RZPDp...  1243       0.0    100%
CR996969.1 CR996969 RZPD no.9017 Homo sapiens cDNA clone RZPDp...  1243       0.0    100%
CR996534.1 CR996534 RZPD no.9017 Homo sapiens cDNA clone RZPDp...  1243       0.0    100%
CR989971.1 CR989971 RZPD no.9017 Homo sapiens cDNA clone RZPDp...  1243       0.0    100%
CR982215.1 CR982215 RZPD no.9016 Homo sapiens cDNA clone RZPDp...  1243       0.0    100%

ALIGNMENTS

Query       416   AATTTGAAGCAGCTGGCTTCACTGCTCAGGTGATTATCCTGAACCATCCAGGCCAAATAAGCGCCGGCTATGCCCCTGTATTGGATTGCCACACGGCTCACATTGCATGCAAGTTTGCTGAGCTGAAGGAAAAGATTGATCGCCGTTCTG  565
CA442866.1  697   ....G.................................................................................................................................................  548
CA417999.1  697   ....G.................................................................................................................................................  548
CA414363.1  699   ....G.................................................................................................................................................  550
CX758265.1  704       ..................................................................................................................................................  559
CT005089.1  1          .................................................................................................................................................  145
CR999128.1  73         .................................................................................................................................................  217
CR996969.1  106        .................................................................................................................................................  250
CR996534.1  175        .................................................................................................................................................  319
CR989971.1  31         .................................................................................................................................................  175
CR982215.1  118        .................................................................................................................................................  262

Query       566   GTAAAAAGCTGGAAGATGGCCCTAAATTCTTGAAGTCTGGTGATGCTGCCATTGTTGATATGGTTCCTGGCAAGCCCATGTGTGTTGAGAGCTTCTCAGACTATCCACCTTTGGGTCGCTTTGCTGTTCGTGATATGAGACAGACAGTTG  715
CA442866.1  547   ......................................................................................................................................................  398
CA417999.1  547   ......................................................................................................................................................  398
CA414363.1  549   ......................................................................................................................................................  400
CX758265.1  558   ......................................................................................................................................................  409
CT005089.1  146   ......................................................................................................................................................  295
```

```
CR999128.1  218   ......................................................................................................................................................  367
CR996969.1  251   ......................................................................................................................................................  400
CR996534.1  320   ......................................................................................................................................................  469
CR989971.1  176   ......................................................................................................................................................  325
CR982215.1  263   ......................................................................................................................................................  412

Query       716   CGGTGGGTGTCATCAAAGCAGTGGACAAGAAGGCTGCTGGAGCTGGCAAGGTCACCAAGTCTGCCCAGAAAGCTCAGAAGGCTAAATGAATATTATCCCTAATACCTGCCACCCCACTCTTAATCAGTGGTGGAAGAACGGTCTCAGAAC  865
CA442866.1  397   ......................................................................................................................................................  248
CA417999.1  397   ......................................................................................................................................................  248
CA414363.1  399   ......................................................................................................................................................  250
CX758265.1  408   ......................................................................................................................................................  259
CT005089.1  296   ......................................................................................................................................................  445
CR999128.1  368   ......................................................................................................................................................  517
CR996969.1  401   ......................................................................................................................................................  550
CR996534.1  470   ......................................................................................................................................................  619
CR989971.1  326   ......................................................................................................................................................  475
CR982215.1  413   ......................................................................................................................................................  562

Query       866   TGTTTGTTTCAATTGGCCATTTAAGTTTAGTAGTAAAAGACTGGTTAATGATAACAATGCATCGTAAAACCTTCAGAAGGAAAGGAGAATgttttgtggaccactttggttttctttttttgcgtgtggcagttttaagttattagttttt  1015
CA442866.1  247   ......................................................................................................................................................  98
CA417999.1  247   ......................................................................................................................................................  98
CA414363.1  249   ......................................................................................................................................................  100
CX758265.1  258   ......................................................................................................................................................  109
CT005089.1  446   ......................................................................................................................................................  595
CR999128.1  518   ......................................................................................................................................................  667
CR996969.1  551   ......................................................................................................................................................  700
CR996534.1  620   ......................................................................................................................................................  769
CR989971.1  476   ......................................................................................................................................................  625
CR982215.1  563   ......................................................................................................................................................  712

Query       1016  aaaatcagtactttttaATGGAAACAACTTGACCAAAAATTTGTCACAGAATTTTGAGACCCATTAAAAAAGTTAAAT  1093
CA442866.1  97    ..............................................................................  20
CA417999.1  97    ..............................................................................  20
CA414363.1  99    ..............................................................................  22
CX758265.1  108   ..............................................................................  31
CT005089.1  596   ..............................................................................  673
CR999128.1  668   ..............................................................................  745
CR996969.1  701   ..............................................................................  778
CR996534.1  770   ..............................................................................  847
CR989971.1  626   ..............................................................................  703
CR982215.1  713   ..............................................................................  790

Query= N18-C* [organism=Homo sapiens] eef1a1

Length=1742


                                                                   Score        E     Max
Sequences producing significant alignments:                       (Bits)     Value  ident

DC527884.1 DC527884 chimpanzee skin cDNA library PstA Pan trog...  3169       0.0    100%
BY794942.2 BY794942 Homo sapiens eye Homo sapiens cDNA clone H...  3162       0.0    100%
CF111220.1 Shultzomica04471 Rat lung airway and parenchyma cDN...  2348       0.0    91%
BX426149.2 BX426149 Homo sapiens NEUROBLASTOMA Homo sapiens cD...  2141       0.0    99%
CO001006.1 OC028 pre-oestrum adult sheep ovary library Ovis ar...  2054       0.0    93%
BX418564.2 BX418564 Homo sapiens FETAL BRAIN Homo sapiens cDNA...  2050       0.0    97%
BX426150.2 BX426150 Homo sapiens NEUROBLASTOMA Homo sapiens cD...  2034       0.0    99%
AL567209.3 AL567209 Homo sapiens FETAL BRAIN Homo sapiens cDNA...  2001       0.0    98%
BU902062.1 AGENCOURT_10127553 NIH_MGC_71 Homo sapiens cDNA clo...  1993       0.0    98%
BX440470.1 BX440470 Homo sapiens FETAL BRAIN Homo sapiens cDNA...  1991       0.0    98%

ALIGNMENTS

Query       3     TTTTTCGCACGGGTTTTGCCGCCAGAACACCAGGTGTCGTGAAAACTACCCCTAAAAGCCAAAATGGGAAAGGAAAAGACTCATATCAACATTGTCGTCATTGGACACGTAGATTCGGGCAAGTCCACCACTACTGGCCATCTGATCTAT  152
DC527884.1  9     .............-...............-........................................................................................................................  157
                          \
                          |
                          A
BY794942.2  25                       ..........-........................................................................................................................  154
CF111220.1  5           .....A.G....-..T......-G.......T......G.C...G........A.......................C........C.....A.................C..............A..C.....C........C  150
                                                                        \
                                                                        |
                                                                     ATTC
BX426150.2  6                                   ........................................................................................................................  125
BU902062.1  2                   ...............-........................................................................................................................  136

Query       153   AAATGCGGTGGCATCGACAAAAGAACCATTGAAAAATTTGAGAAGGAGGCTGCTGAGATGGGAAAGGGCTCCTTCAAGTATGCCTGGGTCTTGGATAAACTGAAAGCTGAGCGTGAACGTGGTATCACCATTGATATCTCCTTGTGGAAA  302
DC527884.1  158   .............................................................................................................................T........................  307
BY794942.2  155   ......................................................................................................................................................  304
CF111220.1  151   .....T.....A.......................G.................G...........A........................C....C....................G...........T.....C......C.......G  300
BX426150.2  126   ......................................................................................................................................................  275
BU902062.1  137   ......................................................................................................................................................  286
```

```
Query       303   TTTGAGACCAGCAAGTACTATGTGACTATCATTGATGCCCCAGGACACAGAGACTTTATCAAAAACATGATTACAGGGACATCTCAGGCTGACTGTGCTGTCCTGATTGTTGCTGCTGGTGTTGGTGAATTTGAAGCTGGTATCTCCAAG  452
DC527884.1  308   ........................................................C.............................................................................................  457
BY794942.2  305   ......................................................................................................................................................  454
CF111220.1  301   ........................................................C.....G...........G..C.................C.............................G........................  450
CO001006.1  1464     ....................T..C.............................C....................C.....C.....................................................C............  1318
BX426150.2  276   ......................................................................................................................................................  425
BU902062.1  287   ......................................................................................................................................................  436

Query       453   AATGGGCAGACCCGAGAGCATGCCCTTCTGGCTTACACACTGGGTGTGAAACAACTAATTGTCGGTGTTAACAAAATGGATTCCACTGAGCCACCCTACAGCCAGAAGAGATATGAGGAAATTGTTAAGGAAGTCAGCACTTACATTAAG  602
DC527884.1  458   ......................................................................................................................................................  607
BY794942.2  455   ......................................................................................................................................................  604
CF111220.1  451   ..C...........T........T..............TT.............G........T.....C.................C........A.....T...........C........C.................C.........  600
BX426149.2  1196                                                                           ......-..A....-.A...............G............................................  1122
CO001006.1  1317  ..C...........T.......................C.......................T..C.................T..............T..............C..........................C.........  1168
BX418564.2  1196                                                                      ....-....-......-...-...-...YH.VS..-.R......-W.-..G..W....-.R......M.....K....-.R.  1125
BX426150.2  426   ......................................................................................................................................................  575
AL567209.3  1145                                                                                                 .......S.....-...W.-..........-K.W........R.....-....A.M.  1092
                                                                                                                                                                \
                                                                                                                                                                |
                                                                                                                                                                M
BU902062.1  437   ......................................................................................................................................................  586
BX440470.1  1128                                                                                                                                                      ....  1125

Query       603   AAAATTGGCTACAACCCCGACACAGTAGCATTTGTGCCAATTTCTGGTTGGAATGGTGACAACATGCTGGAGCCAAGTGCTAACATGCCTTGGTTCAAGGGATGGAAAGTCACCCGTAAGGATGGCAATGCCAGTGGAACCACGCTGCTT  752
DC527884.1  608   ......................................................................................................................................................  757
BY794942.2  605   ......................................................................................................................................................  754
CF111220.1  601   .................T.................................................................T.....G..........................C..A.......G.........C...........G  750
BX426149.2  1121  ......................................................................................................................................................  972
CO001006.1  1167  ...............................................C...................................T.....A................................C....................C......  1018
BX418564.2  1124  -......S....-..........-R.....-.................-.....-..........................C......................-.............................................  982
BX426150.2  576   ......................................................................................................................................................  725
AL567209.3  1091  C............C........................................................................................................................................  942
BU902062.1  587   ......................................................................................................................................................  736
BX440470.1  1124  -.....-..-W.-.............-...-....-S.M...Y..-.K......-..............-.....................K..........................................................  984
                                      \
                                      |
                                      C

Query       753   GAGGCTCTGGACTGCATCCTACCACCAACTCGTCCAACTGACAAGCCCTTGCGCCTGCCTCTCCAGGATGTCTACAAAATTGGTGGTATTGGTACTGTTCCTGTTGGCCGAGTGGAGACTGGTGTTCTCAAACCCGGTATGGTGGTCACC  902
DC527884.1  758   ......................................................................................................................................................  907
BY794942.2  755   ......................................................................................................................................................  904
CF111220.1  751   ..A...T..........T..G..G.......................TC....A.....C..............T........C..C.....C.....C.....G...........A.................T...........T...  900
BX426149.2  971   ......................................................................................................................................................  822
CO001006.1  1017  ..A........T........G...........C...........A.........TT..................T.......................A.....G..T..T.......................T..C............  868
BX418564.2  981   ................................................C.....................................................................................................  832
BX426150.2  726   ......................................................................................................................................................  875
AL567209.3  941   .........K............................................................................................................................................  792
BU902062.1  737   ...................................................................................................................................C..G...............  886
BX440470.1  983   .........R............................................................................................................................................  834

Query       903   TTTGCTCCAGTCAACGTTACAACGGAAGTAAAATCTGTCGAAATGCACCATGAAGCTTTGAGTGAAGCTCTTCCTGGGGACAATGTGGGCTTCAATGTCAAGAATGTGTCTGTCAAGGATGTTCGTCGTGGCAACGTTGCTGGTGACAGC  1052
DC527884.1  908   ......................................................................................................................................................  1057
BY794942.2  905   ......................................................................................................................................................  1054
CF111220.1  901   ..............T..A.....T.....C..G.....G................................G..............A........C..A.....C...........A..C..A..A........T........G......  1050
BX426149.2  821   ......................................................................................................................................................  672
CO001006.1  867   .....C........T..A.....T..G..G..C..A................................C...................................C...........A.....C...........T..G............  718
BX418564.2  831   ................................................................................................................C.....................................  682
BX426150.2  876   ....................................................................................................................................-...............-.  1024
                                                                                   \
                                                                                   |
                                                                                   G
AL567209.3  791   ......................................................................................................................................................  642
BU902062.1  887   .............................-..................................................AC...........C...C..........................C..C......................  1036
                                                                                                                                               \
                                                                                                                                               |
                                                                                                                                               G
BX440470.1  833   ......................................................................................................................................................  684

Query       1053  AAAAATGACCCACCAATGGAAGCAGCTGGCTTCACTGCTCAGGTGATTATCCTGAACCATCCAGGCCAAATAAGCGCCGGCTATGCCCCTGTATTGGATTGCCACACGGCTCACATTGCATGCAAGTTTGCTGAGCTGAAGGAAAAGATT  1202
DC527884.1  1058  ......................................................................................................................................................  1207
BY794942.2  1055  ......................................................................................................................................................  1205
                                                                              \
                                                                              |
                                                                              G
CF111220.1  1051  ....................................................................G..C..T..T..............TC....C...........C.....A..............C.....T..A..G.....C  1200
BX426149.2  671   ......................................................................................................................................................  522
CO001006.1  717   ........T.....C........T......C....A..............T....................C..T.GT..A.....A.....GC.......T.....A...........T.......................G......  568
BX418564.2  681   .................................................................................................................................................M....  532
BX426150.2  1025  ..........-.........-.........-...................-..................-.......-.......-................-......T.-.............                          1140
AL567209.3  641   ....-.................................................................................................................................................  493
BU902062.1  1037  ........................C.G......-.........-.........................T....-.....G.......-..A...........C..G..T...-..............                       1163
```

```
                                                        \                          \                \              \
                                                        |                          |                |              |
                                                        C                          G                T              A
BX440470.1  683   ......................................................................................................................................................  533
                                                                                                                                                                     \
                                                                                                                                                                     |
                                                                                                                                                                     C

Query       1203  GATCGCCGTTCTGGTAAAAAGCTGGAAGATGGCCCTAAATTCTTGAAGTCTGGTGATGCTGCCATTGTTGATATGGTTCCTGGCAAGCCCATGTGTGTTGAGAGCTTCTCAGACTATCCACCTTTGGGTCGCTTTGCTGTTCGTGATATG  1352
DC527884.1  1208  ......................................................................................................................................................  1357
BY794942.2  1206  ......................................................................................................................................................  1355
CF111220.1  1201  .....T...........G.................C...................................C.....C.......................A........T.....C..T.AC..T..T.....T..............C...  1350
BX426149.2  521   ......................................................................................................................................................  372
CO001006.1  567   .....T........G................................A........C........C.......................T........C........T..T..T.....T..C.....C..T........G.....C...  418
BX418564.2  531   ......................................................................................................................................................  382
AL567209.3  492   .............................................................................K........................................................................  343
BX440470.1  532   ......................................................................................................................................................  383

Query       1353  AGACAGACAGTTGCGGTGGGTGTCATCAAAGCAGTGGACAAGAAGGCTGCTGGAGCTGGCAAGGTCACCAAGTCTGCCCAGAAAGCTCAGAAGGCTAAATGAATATTATCCCTAATACCTGCCACCCCACTCTTAATCAGTGGTGGAAGA  1502
DC527884.1  1358  .....................A...............................................................................................................C................  1507
BY794942.2  1356  ......................................................................................................................................................  1505
CF111220.1  1351  ..G...........T.................C.................A...........A....................................................C.............G....................  1500
BX426149.2  371   ......................................................................................................................................................  222
CO001006.1  417   ...........C..T................................A................................................................C................G....................  268
BX418564.2  381   ......................................................................................................................................................  232
AL567209.3  342   ........................................................................................M......Y......................................................  192
                                                                                                                                                                \
                                                                                                                                                                |
                                                                                                                                                                A
BX440470.1  382   ......................................................................................................................................................  233

Query       1503  ACGGTCTCAGAACTGTTTGTTTCAATTGGCCATTTAAGTTTAGTAGTAAAAGACTGGTTAATGATAACAATGCATCGTAAAACCTTCAGAAGGAAAGGAGAATgttttgtggaccactttggttttctttttttgcgtgtggcagttttaa  1652
DC527884.1  1508  ......................................................................................................................................................  1657
BY794942.2  1506  ......................................................................................................................................................  1655
CF111220.1  1501  ....................C.....................A....................................................---..................-...--....-..G.----...............  1639
BX426149.2  221   ......................................................................................................................................................  72
CO001006.1  267   ....................C.....................A.........................................................................-.A..-....G.-----.--........-.....  127
                                                                                                                              \
                                                                                                                              |
                                                                                                                              T
BX418564.2  231   ......................................................................................................................................................  82
AL567209.3  191   ......................................................................................................................................................  41
                                                                                                                                    \
                                                                                                                                    |
                                                                                                                                    G
BX440470.1  232   ......................................................................................................................................................  83

Query       1653  gttattagtttttaaaatcagtactttttaATGGAAACAACTTGACCAAAAATTTGTCACAGAATTTGAGACCCATTAAAAAAGTTAAAT  1742
DC527884.1  1658  ..........................................................................................  1748
                                                                                  \
                                                                                  |
                                                                                  T
BY794942.2  1656  ..........................................................................................  1746
                                                                                  \
                                                                                  |
                                                                                  T
CF111220.1  1640  ............C........................................C....................-...........T...  1730
                                                                                     \             \
                                                                                     |             |
                                                                                     T             C
BX426149.2  71    .................................-.......-.....G......G.-..G..........T..                    1
                                                                                     \
                                                                                     |
                                                                                     T
CO001006.1  126   .................................A...................C................................T...  36
                                                                                                 \
                                                                                                 |
                                                                                                 A
BX418564.2  81    ...................................................................M............            1
AL567209.3  40    ............................-....-...-...-..                                              1
BX440470.1  82    ................................................................................            1
                                                                                     \
                                                                                     |
                                                                                     T

Query= N19-16 [organism=Homo sapiens] eef1a1

Length=1492


                                                                Score        E     Max
Sequences producing significant alignments:                    (Bits)     Value  ident
```

```
CR996534.1 CR996534 RZPD no.9017 Homo sapiens cDNA clone RZPDp...  1500       0.0    100%
BX421307.2 BX421307 Homo sapiens B CELLS (RAMOS CELL LINE) Hom...  1500       0.0    100%
CX167214.1 HESC2_49_G11.g1_A035 NIH_MGC_258 Homo sapiens cDNA ...  1500       0.0    100%
BU845975.1 AGENCOURT_10413042 NIH_MGC_109 Homo sapiens cDNA cl...  1500       0.0    100%
BX443032.2 BX443032 Homo sapiens B CELLS (RAMOS CELL LINE) Hom...  1496       0.0    100%
BX412224.1 BX412224 Homo sapiens PLACENTA Homo sapiens cDNA cl...  1496       0.0    100%
BY794942.2 BY794942 Homo sapiens eye Homo sapiens cDNA clone H...  1496       0.0    100%
BX441194.2 BX441194 Homo sapiens FETAL BRAIN Homo sapiens cDNA...  1495       0.0    100%
BX417098.2 BX417098 Homo sapiens PLACENTA Homo sapiens cDNA cl...  1495       0.0    100%
BU844361.1 AGENCOURT_10414634 NIH_MGC_109 Homo sapiens cDNA cl...  1495       0.0    100%

ALIGNMENTS

Query       21    CGCCAGAACACAGGTGTCGTGAAAACTACCCCTAAAAGCCAAAATGGGAAAGGAAAAGACTCATATCAACATTGTCGTCATTGGACACGTAGATTCGGGCAAGTCCACCACTACTGGCCATCTGATCTATAAATGCGGTGGCATCGACAA  170
BY794942.2  25    ......................................................................................................................................................  174

Query       171   AAGAACCATTGAAAAATTTGAGAAGGAGGCTGCTGAGATGGGAAAGGGCTCCTTCAAGTATGCCTGGGTCTTGGATAAACTGAAAGCTGAGCGTGAACGTGGTATCACCATTGATATCTCCTTGTGGAAATTTGAGACCAGCAAGTACTA  320
BY794942.2  175   ......................................................................................................................................................  324

Query       321   TGTGACTATCATTGATGCCCAGGACACAGAGACTTTATCAAAACATGATTACAGGGACATCTCAGGCTGACTGTGCTGTCCTGATTGTTGCTGCTGTGTTGGTGAATTTGAAGCTGGTATCTCCAAGAATGGGCAGACCGAGAGCATGCC  470
BY794942.2  325   ......................................................................................................................................................  479
                                  \                    \                                                     \                                         \        \
                                  |                    |                                                     |                                         |        |
                                  C                    A                                                     G                                         C        C

Query       471   TTCTGGCTTACACACTGGGTGTGAAAAACTAATTGTCGGTGTTAACAAAATGATTCCACTGAGCCACCTACAGCCAGAAGAGATATGAGGAAATTGTAGAGTCACACTTAATTGCTACCAGTCAACGTTACAACGGAAGTAAATCTGTGA  620
BY794942.2  480   ............................................................................................................                                            601
                                           \                       \             \                             \\\\   \    \
                                           |                       |             |                             ||||   |    |
                                           C                       G             C                             T|||   G    C
                                                                                                                |||
                                                                                                                A||
                                                                                                                 ||
                                                                                                                 G|
                                                                                                                  |
                                                                                                                  A

Query       621   AATGCACATGAttttttttttttttttttttttttttttttttttttttttttAGTAAAATCTGTCGAAATGCACCATGAAGCTTTGAGTGAAGCTCTTCCTGGGGACAATGTGGGCTTCAATGTCAAGAATGTGTCTGTCAAGGAT  770
CR996534.1  33                                                          ................................................................................................  126
BX421307.2  87                                                          ................................................................................................  180
CX167214.1  818                                                         ................................................................................................  725
BU845975.1  816                                                         ................................................................................................  723
BX443032.2  113                                                         ...M............................................................................................  206
BX412224.1  818                                                         ................................................................................................  725
BY794942.2  931                                                         ................................................................................................  1024
BX441194.2  125                                                         ................................................................................................  218
BX417098.2  76                                                          ................................................................................................  169
BU844361.1  825                                                         ...........T....................................................................................  732

Query       771   GTTCGTCGTGGCAACGTTGCTGGTGACAGCAAAAATGACCCACCAATGGAAGCAGCTGGCTTCACTGCTCAGGTGATTATCCTGAACCATCCAGGCCAAATAAGCGCCGGCTATGCCCCTGTATTGGATTGCCACACGGCTCACATTGCA  920
CR996534.1  127   ......................................................................................................................................................  276
BX421307.2  181   ......................................................................................................................................................  330
CX167214.1  724   ......................................................................................................................................................  575
BU845975.1  722   ......................................................................................................................................................  573
BX443032.2  207   ......................................................................................................................................................  356
BX412224.1  724   ......................................................................................................................................................  575
BY794942.2  1025  ......................................................................................................................................................  1175
                                                                                                         \
                                                                                                         |
                                                                                                         G
BX441194.2  219   ......................................................................................................................................................  368
BX417098.2  170   ......................................................................................................................................................  319
BU844361.1  731   ......................................................................................................................................................  582

Query       921   TGCAAGTTTGCTGAGCTGAAGGAAAAGATTGATCGCCGTTCTGGTAAAAAGCTGGAAGATGGCCCTAAATTCTTGAAGTCTGGTGATGCTGCCATTGTTGATATGGTTCCTGGCAAGCCCATGTGTGTTGAGAGCTTCTCAGACTATCCA  1070
CR996534.1  277   ......................................................................................................................................................  426
BX421307.2  331   ......................................................................................................................................................  480
CX167214.1  574   ......................................................................................................................................................  425
BU845975.1  572   ......................................................................................................................................................  423
BX443032.2  357   ......................................................................................................................................................  506
BX412224.1  574   ......................................................................................................................................................  425
BY794942.2  1176  ......................................................................................................................................................  1325
BX441194.2  369   ......................................................................................................................................................  518
BX417098.2  320   ......................................................................................................................................................  469
BU844361.1  581   ......................................................................................................................................................  432

Query       1071  CCTTTGGGTCGCTTTGCTGTTCGTGATATGAGACAGACAGTTGCGGTGGGTGTCATCAAAGCAGTGGACAAGAAGGCTGCTGGAGCTGGCAAGGTCACCAAGTCTGCCCAGAAAGCTCAGAAGGCTAAATGAATATTATCCCTAATACCT  1220
CR996534.1  427   ......................................................................................................................................................  576
BX421307.2  481   ......................................................................................................................................................  630
CX167214.1  424   ......................................................................................................................................................  275
BU845975.1  422   ......................................................................................................................................................  273
```

```
BX443032.2  507   ......................................................................................................................................................  656
BX412224.1  424   ......................................................................................................................................................  275
BY794942.2  1326  ......................................................................................................................................................  1475
BX441194.2  519   ......................................................................................................................................................  668
BX417098.2  470   ......................................................................................................................................................  619
BU844361.1  431   ......................................................................................................................................................  282

Query       1221  GCCACCCCACTCTTAATCAGTGGTGGAAGAACGGTCTCAGAACTGTTTGTTTCAATTGGCCATTTAAGTTTAGTAGTAAAAGACTGGTTAATGATAACAATGCATCGTAAAACCTTCAGAAGGAAAGGAGAATgttttgtggaccacttt  1370
CR996534.1  577   ......................................................................................................................................................  726
BX421307.2  631   ......................................................................................................................................................  780
CX167214.1  274   ......................................................................................................................................................  125
BU845975.1  272   ......................................................................................................................................................  123
BX443032.2  657   ......................................................................................................................................................  806
BX412224.1  274   ......................................................................................................................................................  125
BY794942.2  1476  ......................................................................................................................................................  1625
BX441194.2  669   ......................................................................................................................................................  818
BX417098.2  620   ......................................................................................................................................................  769
BU844361.1  281   ......................................................................................................................................................  132

Query       1371  ggttttctttttttgcgtgtggcagttttaagttattagtttttaaaatcagtactttttaATGGAAACAACTTGACCAAAAAATTTGTCACAGAATTTTGAGACCCATTAAAAAAGTTAAAT  1492
CR996534.1  727   .............................................................................-............................................  847
BX421307.2  781   .............................................................................-............................................  901
CX167214.1  124   ..................................................................................-.......................................  4
BU845975.1  122   ..................................................................................-.......................................  2
BX443032.2  807   .............................................................................-............................................  927
BX412224.1  124   ..................................................................................-.......................................  3
                                                                                                                            \
                                                                                                                            |
                                                                                                                            C
BY794942.2  1626  .............................................................................-............................................  1746
BX441194.2  819   .............................................................................-.........................-..................  938
BX417098.2  770   .............................................................................-.........................-..................  889
BU844361.1  131   ..................................................................................-.......................................  11

Query= N20-6 [organism=Homo sapiens] eef1a1

Length=1602


                                                                   Score        E     Max
Sequences producing significant alignments:                       (Bits)     Value  ident

BY794942.2 BY794942 Homo sapiens eye Homo sapiens cDNA clone H...  1873       0.0    100%
DC527884.1 DC527884 chimpanzee skin cDNA library PstA Pan trog...  1866       0.0    100%
BX418589.2 BX418589 Homo sapiens FETAL BRAIN Homo sapiens cDNA...  1853       0.0    100%
BX440470.1 BX440470 Homo sapiens FETAL BRAIN Homo sapiens cDNA...  1853       0.0    100%
BX442182.2 BX442182 Homo sapiens FETAL BRAIN Homo sapiens cDNA...  1849       0.0    100%
AL541984.3 AL541984 Homo sapiens PLACENTA Homo sapiens cDNA cl...  1845       0.0    99%
BX418564.2 BX418564 Homo sapiens FETAL BRAIN Homo sapiens cDNA...  1845       0.0    99%
BX459625.2 BX459625 Homo sapiens FETAL BRAIN Homo sapiens cDNA...  1844       0.0    100%
BX420340.1 BX420340 Homo sapiens FETAL BRAIN Homo sapiens cDNA...  1840       0.0    100%
BX440308.2 BX440308 Homo sapiens FETAL BRAIN Homo sapiens cDNA...  1836       0.0    99%

ALIGNMENTS

Query       2     TTTTTTCGCAACGGGTTTGCCGCCAGAACACAGGTGTCGTGAAAACTACCCCTAAAAGCCAAAATGGGAAAGGAAAAGACTCATATCAACATTGTCGTCATTGGACACGTAGATTCGGGCAAGTCCACCACTACTGGCCATCTGATCTAT  151
BY794942.2  25                        ..................................................................................................................................  154
DC527884.1  8     ......................................................................................................................................................  157

Query       152   AAATGCGGTGGCATCGACAAAAGAACCATTGAAAAATTTGAGAAGGAGGCTGCTGAGATGGGAAAGGGCTCCTTCAAGTATGCCTGGGTCTTGGATAAACTGAAAGCTGAGCGTGAACGTGGTATCACCATTGATATCTCCTTGTGGAAA  301
BY794942.2  155   ......................................................................................................................................................  304
DC527884.1  158   .............................................................................................................................T........................  307

Query       302   TTTGAGACCAGCAAGTACTATGTGACTATCATTGATGCCCCAGGACACAGAGACTTTATCAAAAACATGATTACAGGGACATCTCAGGCTGACTGTGCTGTCCTGATTGTTGCTGCTGGTGTTGGTGAATTTGAAGCTGGTATCTCCAAG  451
BY794942.2  305   ......................................................................................................................................................  454
DC527884.1  308   ........................................................C.............................................................................................  457

Query       452   AATGGGCAGACCCGAGAGCATGCCCTTCTGGCTTACACACTGGGTGTGAAACAACTAATTGTCGGTGTTAACAAAATGGATTCCACTGAGCCACCCTACAGCCAGAAGAGATATGAGGAAATTGTTAAGGATGGCAATGCCAGTGGAACC  601
BY794942.2  721                                                                                                                                ........................  745
BY794942.2  455   ...................................................................................................................................  585
DC527884.1  724                                                                                                                                ........................  748
DC527884.1  458   ...................................................................................................................................  588
BX418589.2  1016                                                                                                                               ........................  992
BX440470.1  1017                                                                                                                               ........................  993
BX442182.2  1009                                                                                                                               ........................  985
AL541984.3  1                                                                                                                                             .............  14
BX418564.2  1015                                                                                                                               ........................  991
BX459625.2  1004                                                                                                                               ........................  980
BX420340.1  1010                                                                                                                               ........................  986
BX440308.2  1014                                                                                                                               .......-..........-K.....  992

Query       602   ACGCTGCTTGAGGCTCTGGACTGCATCCTACCACCAACTCGTCCAACTGACAAGCCCTTGCGCCTGCCTCTCCAGGATGTCTACAAAATTGGTGGTATTGGTACTGTTCCTGTTGGCCGAGTGGAGACTGGTGTTCTCAAACCCGGTATG  751
```

```
BY794942.2  746   ......................................................................................................................................................  895
DC527884.1  749   ......................................................................................................................................................  898
BX418589.2  991   ......................................................................................................................................................  842
BX440470.1  992   ..................R.......................................................................................................................................  843
BX442182.2  984   .............................................................................................................................R........................  835
AL541984.3  15    ......................................................................................................................................................  164
BX418564.2  990   .........................................................C............................................................................................  841
BX459625.2  979   ......................................................................................................................................................  830
BX420340.1  985   ..................A.............................R.....................................................................................................  836
BX440308.2  991   M.................R.............M.....................................................................................................................  842

Query       752   GTGGTCACCTTTGCTCCAGTCAACGTTACAACGGAAGTAAAATCTGTCGAAATGCACCATGAAGCTTTGAGTGAAGCTCTTCCTGGGGACAATGTGGGCTTCAATGTCAAGAATGTGTCTGTCAAGGATGTTCGTCGTGGCAACGTTGCT  901
BY794942.2  896   ......................................................................................................................................................  1045
DC527884.1  899   ......................................................................................................................................................  1048
BX418589.2  841   ......................................................................................................................................................  692
BX440470.1  842   ......................................................................................................................................................  693
BX442182.2  834   ......................................................................................................................................................  685
AL541984.3  165   ......................................................................................................................................................  314
BX418564.2  840   .........................................................................................................................C............................  691
BX459625.2  829   ......................................................................................................................................................  680
BX420340.1  835   ......................................................................................................................................................  686
BX440308.2  841   ......................................................................................................................................................  692

Query       902   GGTGACAGCAAAAATGACCCACCAATGGAAGCAGCTGGCTTCACTGCTCAGGTGATTATCCTGAACCATCCAGGCCAAATAAGCGCCGGCTATGCCCCTGTATTGGATTGCCACACGGCTCACATTGCATGCAAGTTTGCTGAGCTGAAG  1051
BY794942.2  1046  ......................................................................................................................................................  1196
                                                                                       \
                                                                                       |
                                                                                       G
DC527884.1  1049  ......................................................................................................................................................  1198
BX418589.2  691   ......................................................................................................................................................  542
BX440470.1  692   ......................................................................................................................................................  543
BX442182.2  684   ......................................................................................................................................................  535
AL541984.3  315   ......................................................................................................................................................  464
BX418564.2  690   ......................................................................................................................................................  541
BX459625.2  679   ......................................................................................................................................................  530
BX420340.1  685   ......................................................................................................................................................  536
BX440308.2  691   ..............................................................................M.......................................................................  542

Query       1052  GAAAAGATTGATCGCCGTTCTGGTAAAAAGCTGGAAGATGGCCCTAAATTCTTGAAGTCTGGTGATGCTGCCATTGTTGATATGGTTCCTGGCAAGCCCATGTGTGTTGAGAGCTTCTCAGACTATCCACCTTTGGGTCGCTTTGCTGTT  1201
BY794942.2  1197  ......................................................................................................................................................  1346
DC527884.1  1199  ......................................................................................................................................................  1348
BX418589.2  541   ...........Y.S........................................................................................................................................  392
BX440470.1  542   ......................................................................................................................................................  392
                        \
                        |
                        C
BX442182.2  534   ......................................................................................................................................................  385
AL541984.3  465   ......................................................................................................................................................  614
BX418564.2  540   ....M.................................................................................................................................................  391
BX459625.2  529   ......................................................................................................................................................  380
BX420340.1  535   ......................................................................................................................................................  386
BX440308.2  541   ......................................................................................................................................................  392

Query       1202  CGTGATATGAGACAGACAGTTGCGGTGGGTGTCATCAAAGCAGTGGACAAGAAGGCTGCTGGAGCTGGCAAGGTCACCAAGTCTGCCCAGAAAGCTCAGAAGGCTAAATGAATATTATCCCTAATACCTGCCACCCCACTCTTAATCAGT  1351
BY794942.2  1347  ......................................................................................................................................................  1496
DC527884.1  1349  ..............................A...............................................................................................................C.......  1498
BX418589.2  391   ......................................................................................................................................................  242
BX440470.1  391   ......................................................................................................................................................  242
BX442182.2  384   ......................................................................................................................................................  235
AL541984.3  615   ......................................................................................................................................................  764
BX418564.2  390   ......................................................................................................................................................  241
BX459625.2  379   ......................................................................................................................................................  230
BX420340.1  385   ......................................................................................................................................................  236
BX440308.2  391   ......................................................................................................................................................  242

Query       1352  GGTGGAAGAACGGTCTCAGAACTGTTTGTTTCAATTGGCCATTTAAGTTTAGTAGTAAAAGACTGGTTAATGATAACAATGCATCGTAAAACCTTCAGAAGGAAAGGAGAATgttttgtggaccactttggttttctttttttgcgtgtgg  1501
BY794942.2  1497  ......................................................................................................................................................  1646
DC527884.1  1499  ......................................................................................................................................................  1648
BX418589.2  241   ......................................................................................................................................................  92
BX440470.1  241   ......................................................................................................................................................  92
BX442182.2  234   ......................................................................................................................................................  85
AL541984.3  765   ....................................................................................................................................................K.  914
BX418564.2  240   ......................................................................................................................................................  91
BX459625.2  229   ......................................................................................................................................................  80
BX420340.1  235   ......................................................................................................................................................  86
BX440308.2  241   ......................................................................................................................................................  92

Query       1502  cagttttaagttattagtttttaaaatcagtactttttaATGGAAACAACTTGACCAAAAAAATTTGTCACAGAATTTGAGACCCCATTAAAAAAGTTAAAT  1602
BY794942.2  1647  ........................................................-.........................-...................  1746
                                                                                                  \
                                                                                                  |
                                                                                                  T
```

```
DC527884.1  1649  ........................................................-........................-...................  1748
                                                                                             \
                                                                                             |
                                                                                             T
BX418589.2  91    .............................................................-......................-.......           1
                                                                                             \
                                                                                             |
                                                                                             D
BX440470.1  91    .............................................................-......................-.......           1
                                                                                             \
                                                                                             |
                                                                                             T
BX442182.2  84    .............................................................-.......................                  1
                                                                                             \
                                                                                             |
                                                                                             T
AL541984.3  915   S............................................................-...................-...................  1015
                                                                                      \      \
                                                                                      |      |
                                                                                      M      T
BX418564.2  90    .............................................................-...............M......-.......           1
BX459625.2  79    .............................................................-...............                          4
BX420340.1  85    .............................................................-.................R....                   2
                                                                                             \
                                                                                             |
                                                                                             T
BX440308.2  91    .............................................................-......................-........          1
```

Query= N21-3 [organism=Homo sapiens] eef1a1

Length=656

```
                                                                  Score        E     Max
Sequences producing significant alignments:                      (Bits)     Value  ident

BX441194.2 BX441194 Homo sapiens FETAL BRAIN Homo sapiens cDNA...  1205       0.0    100%
BX417098.2 BX417098 Homo sapiens PLACENTA Homo sapiens cDNA cl...  1205       0.0    100%
CA422292.1 UI-H-FG0-bdg-d-06-0-UI.s1 NCI_CGAP_EN1_2 Homo sapie...  1203       0.0    100%
BU628737.1 UI-H-FG0-bdg-k-06-0-UI.s1 NCI_CGAP_EN1_2 Homo sapie...  1203       0.0    100%
BU628664.1 UI-H-FG0-bdf-l-02-0-UI.s1 NCI_CGAP_EN1_2 Homo sapie...  1203       0.0    100%
CT005089.1 CT005089 RZPD no.9017 Homo sapiens cDNA clone RZPDp...  1201       0.0    100%
CR999128.1 CR999128 RZPD no.9017 Homo sapiens cDNA clone RZPDp...  1201       0.0    100%
CR996969.1 CR996969 RZPD no.9017 Homo sapiens cDNA clone RZPDp...  1201       0.0    100%
CR996534.1 CR996534 RZPD no.9017 Homo sapiens cDNA clone RZPDp...  1201       0.0    100%
CR989971.1 CR989971 RZPD no.9017 Homo sapiens cDNA clone RZPDp...  1201       0.0    100%
```

ALIGNMENTS

```
Query       1    TGCTCAGGTGATTATCCTGAACCATCCAGGCCAAATAAGCGCCGGCTATGCCCCTGTATTGGATTGCCACACGGCTCACATTGCATGCAAGTTTGCTGAGCTGAAGGAAAAGATTGATCGCCGTTCTGGTAAAAAGCTGGAAGATGGCCC  150
BX441194.2  284  ......................................................................................................................................................  433
BX417098.2  235  ......................................................................................................................................................  384
CA422292.1  678  ......................................................................................................................................................  529
BU628737.1  678  ......................................................................................................................................................  529
BU628664.1  678  ......................................................................................................................................................  529
CT005089.1  18   ......................................................................................................................................................  167
CR999128.1  90   ......................................................................................................................................................  239
CR996969.1  123  ......................................................................................................................................................  272
CR996534.1  192  ......................................................................................................................................................  341
CR989971.1  48   ......................................................................................................................................................  197

Query       151  TAAATTCTTGAAGTCTGGTGATGCTGCCATTGTTGATATGGTTCCTGGCAAGCCCATGTGTGTTGAGAGCTTCTCAGACTATCCACCTTTGGGTCGCTTTGCTGTTCGTGATATGAGACAGACAGTTGCGGTGGGTGTCATCAAAGCAGT  300
BX441194.2  434  ......................................................................................................................................................  583
BX417098.2  385  ......................................................................................................................................................  534
CA422292.1  528  ......................................................................................................................................................  379
BU628737.1  528  ......................................................................................................................................................  379
BU628664.1  528  ......................................................................................................................................................  379
CT005089.1  168  ......................................................................................................................................................  317
CR999128.1  240  ......................................................................................................................................................  389
CR996969.1  273  ......................................................................................................................................................  422
CR996534.1  342  ......................................................................................................................................................  491
CR989971.1  198  ......................................................................................................................................................  347

Query       301  GGACAAGAAGGCTGCTGGAGCTGGCAAGGTCACCAAGTCTGCCCAGAAAGCTCAGAAGGCTAAATGAATATTATCCCTAATACCTGCCACCCCACTCTTAATCAGTGGTGGAAGAACGGTCTCAGAACTGTTTGTTTCAATTGGCCATTT  450
BX441194.2  584  ......................................................................................................................................................  733
BX417098.2  535  ......................................................................................................................................................  684
CA422292.1  378  ......................................................................................................................................................  229
BU628737.1  378  ......................................................................................................................................................  229
BU628664.1  378  ......................................................................................................................................................  229
CT005089.1  318  ......................................................................................................................................................  467
CR999128.1  390  ......................................................................................................................................................  539
CR996969.1  423  ......................................................................................................................................................  572
```

```
CR996534.1  492  ......................................................................................................................................................  641
CR989971.1  348  ......................................................................................................................................................  497

Query       451  AAGTTTAGTAGTAAAAGACTGGTTAATGATAACAATGCATCGTAAAACCTTCAGAAGGAAAGGAGAATgttttgtggaccactttggttttctttttttgcgtgtggcagttttaagttattagtttttaaaatcagtacttttttaATGGA  600
BX441194.2  734  ......................................................................................................................................................  883
BX417098.2  685  ......................................................................................................................................................  834
CA422292.1  228  ......................................................................................................................................................  79
BU628737.1  228  ......................................................................................................................................................  79
BU628664.1  228  ......................................................................................................................................................  79
CT005089.1  468  ......................................................................................................................................................  617
CR999128.1  540  ......................................................................................................................................................  689
CR996969.1  573  ......................................................................................................................................................  722
CR996534.1  642  ......................................................................................................................................................  791
CR989971.1  498  ......................................................................................................................................................  647

Query       601  AACAACTTGACCAAAAAATTTGTCACAGAATTTTGAGACCATTAAAAAAGTTAAAT  656
BX441194.2  884  ............-...........................................  938
BX417098.2  835  ............-...........................................  889
CA422292.1  78   .............N..........................................  22
                                                         \
                                                         |
                                                         C
BU628737.1  78   .............N..........................................  22
                                                         \
                                                         |
                                                         C
BU628664.1  78   .............N..........................................  22
                                                         \
                                                         |
                                                         C
CT005089.1  618  ............-...........................................  673
                                                       \
                                                       |
                                                       C
CR999128.1  690  ............-...........................................  745
                                                       \
                                                       |
                                                       C
CR996969.1  723  ............-...........................................  778
                                                       \
                                                       |
                                                       C
CR996534.1  792  ............-...........................................  847
                                                       \
                                                       |
                                                       C
CR989971.1  648  ............-...........................................  703
                                                       \
                                                       |
                                                       C
```

Query= N22-5 [organism=Homo sapiens] eef1a1

Length=1719

```
                                                                   Score        E     Max
Sequences producing significant alignments:                       (Bits)     Value  ident

DC527884.1 DC527884 chimpanzee skin cDNA library PstA Pan trog...  3053       0.0    99%
BY794942.2 BY794942 Homo sapiens eye Homo sapiens cDNA clone H...  3037       0.0    99%
BX426149.2 BX426149 Homo sapiens NEUROBLASTOMA Homo sapiens cD...  2012       0.0    97%
BX418564.2 BX418564 Homo sapiens FETAL BRAIN Homo sapiens cDNA...  1932       0.0    96%
BX440470.1 BX440470 Homo sapiens FETAL BRAIN Homo sapiens cDNA...  1929       0.0    100%
BX418589.2 BX418589 Homo sapiens FETAL BRAIN Homo sapiens cDNA...  1921       0.0    100%
BX442182.2 BX442182 Homo sapiens FETAL BRAIN Homo sapiens cDNA...  1919       0.0    100%
BX420340.1 BX420340 Homo sapiens FETAL BRAIN Homo sapiens cDNA...  1917       0.0    100%
BX459625.2 BX459625 Homo sapiens FETAL BRAIN Homo sapiens cDNA...  1910       0.0    100%
BX325185.1 BX325185 Homo sapiens T CELLS (JURKAT CELL LINE) CO...  1892       0.0    99%
```

ALIGNMENTS

```
Query       2     TTTTTCGCAACGGGTTTGCCGCCAGAACACAGGTGTCGTGAAAACTACCCCTAAAAGCCAAAATGGGAAAGGAAAAGACTCATATCAACATTGTCGTCATTGGACACGTAGATTCGGGCAAGTCCACCACTACTGGCCATCTGATCTATA  151
DC527884.1  9     ......................................................................................................................................................  158
BY794942.2  25                       ...................................................................................................................................  155

Query       152   AATGCGGTGGCATCGACAAAAGAACCATTGAAAAATTTGAGAAGGAGGCTGCTGAGATGGGAAAGGGCTCCTTCAAGTATGCCTGGGTCTTGGATAAACTGAAAGCTGAGCGTGAACGTGGTATCACCATTGATATCTCCTTGTGGAAAT  301
DC527884.1  159   ............................................................................................................................T.........................  308
BY794942.2  156   ......................................................................................................................................................  305

Query       302   TTGAGACCAGCAAGTACTATGTGACTATCATTGATGCCCCAGGACACAGAGACTTTATCAAAAACATGATTACAGGGACATCTCAGGCTGACTGTGCTGTCCTGATTGTTGCTGCTGGTGTTGGTGAATTTGAAGCTGGTATCTCCAAGA  451
DC527884.1  309   .......................................................C..............................................................................................  458
```

```
BY794942.2  306   ......................................................................................................................................................  455

Query       452   ATGGGCAGACCCGAGAGCATGCCCTTCTGGCTTACACACTGGGTGTGAAACAACTAATTGTCGGTGTTAACAAAATGGATTCCACTGAGCCACCCTACAGCCAGAAGAGATATGAGGAAATTGTTAAGGAAGTCAGCACTTACATTAAGA  601
DC527884.1  459   ......................................................................................................................................................  608
BY794942.2  456   ......................................................................................................................................................  605
BX426149.2  1196                                                                            .....-..A...-.A................G............................................  1121
BX418564.2  1196                                                                     ....-....-......-...-...-...YH.VS..-.R......-W.-..G..W....-.R......M.....K....-.R.-  1125

Query       602   AAATTGGCTACAACCCCGACACAGTAGCATTTGTGCCAATTTCTGGTTGGAATGGTGACAACATGCCTTGGTTCAAGGGATGGAAAGTCACCCGTAAGGATGGCAATGCCAGTGGAACCACGCTGCTTGAGGCTCTGGACTGCATCCTAC  751
DC527884.1  609   ......................................................................................................................................................  779
                                                                             \
                                                                             |
                                                         AACATGCTGGAGCCAAGTGCT
BY794942.2  606   ......................................................................................................................................................  776
                                                                             \
                                                                             |
                                                         AACATGCTGGAGCCAAGTGCT
BX426149.2  1120  ......................................................................................................................................................  950
                                                                                    \
                                                                                    |
                                                                TGGAGCCAAGTGCTAACATGC
BX418564.2  1124  ......S....-..........-R.....-........................C...........................-...................................................................  960
                                                                 \ \\\    \ \\\
                                                                 | |||    | |||
                                                              GGAA T||    G GC|
                                                                    ||       ||
                                                                  ACA|       A|
                                                                     |        |
                                                                     C   GTGCTC
BX440470.1  1052                                                             ..........K...................................................................R............  962
BX418589.2  1051                                                             ...........K.C.............................................................................  961
BX442182.2  1041                                                                ........................................................................................  954
BX420340.1  1045                                                             ..............................................................................A............  955
BX459625.2  1039                                                             ...........K.A.............................................................................  949
BX325185.1  1050                                                             .....C.Y...................K..M....W.................K.R.M..........K.........A............  960

Query       752   CACCAACTCGTCCAACTGACAAGCCCTTGCGCCTGCCTCTCCAGGATGTCTACAAAATTGGTGGTATTGGTACTGTTCCTGTTGGCCGAGTGGAGACTGGTGTTCTCAAACCCGGTATGGTGGTCACCTTTGCTCCAGTCAACGTTACAA  901
DC527884.1  780   ......................................................................................................................................................  929
BY794942.2  777   ......................................................................................................................................................  926
BX426149.2  949   ......................................................................................................................................................  800
BX418564.2  959   ..........................C...........................................................................................................................  810
BX440470.1  961   ......................................................................................................................................................  812
BX418589.2  960   ......................................................................................................................................................  811
BX442182.2  953   ..............................................................................................R.......................................................  804
BX420340.1  954   .................R....................................................................................................................................  805
BX459625.2  948   ......................................................................................................................................................  799
BX325185.1  959   .................R....................................................................................................................................  810

Query       902   CGGAAGTAAAATCTGTCGAAATGCACCATGAAGCTTTGAGTGAAGCTCTTCCTGGGGACAATGTGGGCTTCAATGTCAAGAATGTGTCTGTCAAGGATGTTCGTCGTGGCAACGTTGCTGGTGACAGCAAAAATGACCCACCAATGGAAG  1051
DC527884.1  930   ......................................................................................................................................................  1079
BY794942.2  927   ......................................................................................................................................................  1076
BX426149.2  799   ......................................................................................................................................................  650
BX418564.2  809   ..........................................................................................C...........................................................  660
BX440470.1  811   ......................................................................................................................................................  662
BX418589.2  810   ......................................................................................................................................................  661
BX442182.2  803   ......................................................................................................................................................  654
BX420340.1  804   ......................................................................................................................................................  655
BX459625.2  798   ......................................................................................................................................................  649
BX325185.1  809   ......................................................................................................................................................  660

Query       1052  CAGCTGGCTTCACTGCTCAGGTGATTATCCTGAACCATCCAGGCCAAATAAGCGCCGGCTATGCCCCTGTATTGGATTGCCACACGGCTCACATTGCATGCAAGTTTGCTGAGCTGAAGGAAAAGATTGATCGCCGTTCTGGTAAAAAGC  1201
DC527884.1  1080  ......................................................................................................................................................  1229
BY794942.2  1077  ......................................................................................................................................................  1227
                                                        \
                                                        |
                                                        G
BX426149.2  649   ......................................................................................................................................................  500
BX418564.2  659   ...........................................................................................................................M..........................  510
BX440470.1  661   ......................................................................................................................................................  511
                                                                                                                                           \
                                                                                                                                           |
                                                                                                                                           C
BX418589.2  660   ..................................................................................................................................Y.S.................  511
BX442182.2  653   ......................................................................................................................................................  504
BX420340.1  654   ......................................................................................................................................................  505
BX459625.2  648   ......................................................................................................................................................  499
BX325185.1  659   ..............................................................................................Y.......................................................  510

Query       1202  TGGAAGATGGCCCTAAATTCTTGAAGTCTGGTGATGCTGCCATTGTTGATATGGTTCCTGGCAAGCCCATGTGTGTTGAGAGCTTCTCAGACTATCCACCTTTGGGTCGCTTTGCTGTTCGTGATATGAGACAGACAGTTGCGGTGGGTG  1351
DC527884.1  1230  .....................................................................................................................................................A  1379
BY794942.2  1228  ......................................................................................................................................................  1377
BX426149.2  499   ......................................................................................................................................................  350
```

```
BX418564.2  509   ......................................................................................................................................................  360
BX440470.1  510   ......................................................................................................................................................  361
BX418589.2  510   ......................................................................................................................................................  361
BX442182.2  503   ......................................................................................................................................................  354
BX420340.1  504   ......................................................................................................................................................  355
BX459625.2  498   ......................................................................................................................................................  349
BX325185.1  509   ......................................................................................................................................................  360

Query       1352  TCATCAAAGCAGTGGACAAGAAGGCTGCTGGAGCTGGCAAGGTCACCAAGTCTGCCCAGAAAGCTCAGAAGGCTAAATGAATATTATCCCTAATACCTGCCACCCCACTCTTAATCAGTGGTGGAAGAACGGTCTCAGAACTGTTTGTTT  1501
DC527884.1  1380  ...............................................................................................................C......................................  1529
BY794942.2  1378  ......................................................................................................................................................  1527
BX426149.2  349   ......................................................................................................................................................  200
BX418564.2  359   ......................................................................................................................................................  210
BX440470.1  360   ......................................................................................................................................................  211
BX418589.2  360   ......................................................................................................................................................  211
BX442182.2  353   ......................................................................................................................................................  204
BX420340.1  354   ......................................................................................................................................................  205
BX459625.2  348   ......................................................................................................................................................  199
BX325185.1  359   ......................................................................................................................................................  210

Query       1502  CAATTGGCCATTTAAGTTTAGTAGTAAAAGACTGGTTAATGATAACAATGCATCGTAAAACCTTCAGAAGGAAAGGAGAATgttttgtggaccactttggttttctttttttgcgtgtggcagttttaagttattagtttttaaaatcagt  1651
DC527884.1  1530  ......................................................................................................................................................  1679
BY794942.2  1528  ......................................................................................................................................................  1677
BX426149.2  199   ......................................................................................................................................................  50
BX418564.2  209   ......................................................................................................................................................  60
BX440470.1  210   ......................................................................................................................................................  61
BX418589.2  210   ......................................................................................................................................................  61
BX442182.2  203   ......................................................................................................................................................  54
BX420340.1  204   ......................................................................................................................................................  55
BX459625.2  198   ......................................................................................................................................................  49
BX325185.1  209   ......................................................................................................................................................  60

Query       1652  actttttaATGGAAACAACTTGACCAAAAATTTGTCACAGAATTTTGAGACCCATTAAAAAAGTTAAA  1719
DC527884.1  1680  ....................................................................  1747
BY794942.2  1678  ....................................................................  1745
BX426149.2  49    ...........-.......-.....G......G.-..G...........T..                  1
BX418564.2  59    .............................................M-.............         1
BX440470.1  60    ............................................................          1
BX418589.2  60    .............................................D..............          1
BX442182.2  53    .....................................................                 1
BX420340.1  54    ................................................R.....                1
BX459625.2  48    ................................................                      1
BX325185.1  59    ...........................................................           1

Query= N23-12 [organism=Homo sapiens] eef1a1

Length=1128


                                                                      Score        E     Max
Sequences producing significant alignments:                          (Bits)     Value  ident

CD684311.1 EST831 human nasopharynx Homo sapiens cDNA, mRNA se...  1136       0.0    98%
CR768893.1 DKFZp468L0412_r1 468 (synonym: phrt1) Pongo abelii ...  1072       0.0    100%
DA037335.1 DA037335 BGGI11 Homo sapiens cDNA clone BGGI1100023...  1072       0.0    100%
DA085397.1 DA085397 BRACE2 Homo sapiens cDNA clone BRACE203976...  1072       0.0    100%
DB247234.1 DB247234 UTERU1 Homo sapiens cDNA clone UTERU100011...  1072       0.0    100%
DB246583.1 DB246583 UMVEN1 Homo sapiens cDNA clone UMVEN100002...  1072       0.0    100%
DB100941.1 DB100941 THYMU1 Homo sapiens cDNA clone THYMU100016...  1072       0.0    100%
DB100909.1 DB100909 THYMU1 Homo sapiens cDNA clone THYMU100013...  1072       0.0    100%
DB194465.1 DB194465 TRACH1 Homo sapiens cDNA clone TRACH100013...  1072       0.0    100%
DB194442.1 DB194442 TRACH1 Homo sapiens cDNA clone TRACH100011...  1072       0.0    100%

ALIGNMENTS

Query       1    CTTTTTTCGCAACGGGTTTGCCGCCAGAACACAGGTGTCGTGAAAACTACCCCTAAAAGCCAAAAATGGGAAAGGAAAAGACTCATATCAACATTGTCGTCATTGGACACGTAGATTCGGGCAAGTCCACCACTACTGGCCATCTGATCT  150
CD684311.1  24     ..............-............................................-........................................................................................  169
CR768893.1  2    .............................................................-........................................................................................  150
DA037335.1  1    .............................................................-........................................................................................  149
DA085397.1  1    .............................................................-........................................................................................  149
DB247234.1  1    .............................................................-........................................................................................  149
DB246583.1  1    .............................................................-........................................................................................  149
DB100941.1  1    .............................................................-........................................................................................  149
DB100909.1  1    .............................................................-........................................................................................  149
DB194465.1  4    .............................................................-........................................................................................  152
DB194442.1  1    .............................................................-........................................................................................  149

Query       151  ATAAATGCGGTGGCATCGACAAAAGAACCATTGAAAAATTTGAGAAGGAGGCTGCTGAGATGGGAAAGGGCTCCTTCAAGTATGCCTGGGTCTTGGATAAACTGAAAGCTGAGCGTGAACGTGGTATCACCATTGATATCTCCTTGTGGA  300
CD684311.1  170  ......................................................................................................................................................  319
CR768893.1  151  ......................................................................................................................................................  300
DA037335.1  150  ......................................................................................................................................................  299
DA085397.1  150  ......................................................................................................................................................  299
```

```
DB247234.1  150  ......................................................................................................................................................  299
DB246583.1  150  ......................................................................................................................................................  299
DB100941.1  150  ......................................................................................................................................................  299
DB100909.1  150  ......................................................................................................................................................  299
DB194465.1  153  ......................................................................................................................................................  302
DB194442.1  150  ......................................................................................................................................................  299

Query       301  AATTTGAGACCAGCAAGTACTATGTGACTATCATTGATGCCCCAGGACACAGAGACTTTATCAAAAACATGATTACAGGGACATCTCAGGCTGACTGTGCTGTCCTGATTGTTGCTGCTGGTGTTGGTGAATTTGAAGCTGGTATCTCCA  450
CD684311.1  320  ................................................................................................................A.....................................  469
CR768893.1  301  ......................................................................................................................................................  450
DA037335.1  300  ......................................................................................................................................................  449
DA085397.1  300  ......................................................................................................................................................  449
DB247234.1  300  ......................................................................................................................................................  449
DB246583.1  300  ......................................................................................................................................................  449
DB100941.1  300  ......................................................................................................................................................  449
DB100909.1  300  ......................................................................................................................................................  449
DB194465.1  303  ......................................................................................................................................................  452
DB194442.1  300  ......................................................................................................................................................  449

Query       451  AGAATGGGCAGACCCGAGAGCATGCCCTTCTGGCTTACACACTGGGTGTGAAACAACTAATTGTCGGTGTTAACAAAATGGATTCCACTGAGCCACCCTACAGCCAGAAGAGATATGAGGAAATTGTTAAGGAAAAGATTGATCGCCGTT  600
CD684311.1  470  .....................................................................................................................------------.....................  607
CR768893.1  451  ......................................................................................................................................                  584
DA037335.1  450  ......................................................................................................................................                  583
DA085397.1  450  ......................................................................................................................................                  583
DB247234.1  450  ......................................................................................................................................                  583
DB246583.1  450  ......................................................................................................................................                  583
DB100941.1  450  ......................................................................................................................................                  583
DB100909.1  450  ......................................................................................................................................                  583
DB194465.1  453  ......................................................................................................................................                  586
DB194442.1  450  ......................................................................................................................................                  583

Query       601  CTGGTAAAAAGCTGGAAGATGGCCCTAAATTCTTGAAGTCTGGTGATGCTGCCATTGTTGATATGGTTTCCTG  673
CD684311.1  608  ....................................................................-....  679
```

Query= N24-22 [organism=Homo sapiens] eef1a1

Length=1117

```
                                                                   Score        E     Max
Sequences producing significant alignments:                       (Bits)     Value  ident

BX421307.2 BX421307 Homo sapiens B CELLS (RAMOS CELL LINE) Hom...  1596       0.0    100%
BX443032.2 BX443032 Homo sapiens B CELLS (RAMOS CELL LINE) Hom...  1592       0.0    100%
BY794942.2 BY794942 Homo sapiens eye Homo sapiens cDNA clone H...  1592       0.0    100%
BQ049312.1 AGENCOURT_6795094 NIH_MGC_85 Homo sapiens cDNA clon...  1592       0.0    100%
BX417098.2 BX417098 Homo sapiens PLACENTA Homo sapiens cDNA cl...  1591       0.0    100%
BX441194.2 BX441194 Homo sapiens FETAL BRAIN Homo sapiens cDNA...  1587       0.0    100%
DC527884.1 DC527884 chimpanzee skin cDNA library PstA Pan trog...  1585       0.0    100%
AL541984.3 AL541984 Homo sapiens PLACENTA Homo sapiens cDNA cl...  1585       0.0    100%
CR984260.1 CR984260 RZPD no.9017 Homo sapiens cDNA clone RZPDp...  1583       0.0    100%
BX440436.2 BX440436 Homo sapiens FETAL BRAIN Homo sapiens cDNA...  1583       0.0    100%
```

ALIGNMENTS

```
Query       1     GAAGTCAGCACTTACATTAAGAAAATTGGCTACAACCCCGACACAGTAGCATTTGTGCCAATTTCTGGTTGGAATGGTGACAACATGCTGGAGCCAAGTGCTAACATGCCTTGGTTCAAGGGATGGAAAGTCACCGTAAGGATGGCAATG  150
BY794942.2  584   ......................................................................................................................................................  734
                                                                                                                                                       \
                                                                                                                                                       |
                                                                                                                                                       C
DC527884.1  587   ......................................................................................................................................................  737
                                                                                                                                                       \
                                                                                                                                                       |
                                                                                                                                                       C
AL541984.3  1                                                                                                                                                    ...  3
BX440436.2  1087                                                               .....-........-...........-.K.........Y......-S.............-K.................-......  1004
                                                                                                                                                       \
                                                                                                                                                       |
                                                                                                                                                       C

Query       151   CCAGTGGAACCACGCTGCTTGAGGCTCTGGACTGCATCCTACCACCAACTCGTCCAACTGACAAGCCTTGCGCCTGCCTCTCCAGGATGTCTACAAAACTTGGTGTTCTCAAACCCGGTATGGTGGTCACCTTTGCTCCAGTCAACGTTA  300
BX421307.2  30                                                                                                        ..................................................  79
BX443032.2  56                                                                                                        ..................................................  105
BY794942.2  874                                                                                                       ..................................................  923
BY794942.2  735   ..................................................................................................-......                                             839
                                                                   \
                                                                   |
                                                                   C
BQ049312.1  28                                                                                                        ..................................................  77
BX417098.2  19                                                                                                        ..................................................  68
BX441194.2  68                                                                                                        .......................W..........................  117
DC527884.1  877                                                                                                       ..................................................  926
```

```
DC527884.1  738   ..................................................................................................-......  842
                                                                                   \
                                                                                   |
                                                                                   C
AL541984.3  143                                                                                                       ..................................................  192
AL541984.3  4     ..................................................................................................-......  108
                                                                                   \
                                                                                   |
                                                                                   C
CR984260.1  46                                                                                                        ..................................................  95
BX440436.2  866                                                                                                       ..................................................  817
BX440436.2  1003  ..............-YK.....-........................................R..................................-......  901
                                                                                     \
                                                                                     |
                                                                                     C

Query       301   CAACGGAAGTAAAATCTGTCGAAATGCACCATGAAGCTTTGAGTGAAGCTCTTCCTGGGGACAATGTGGGCTTCAATGTCAAGAATGTGTCTGTCAAGGATGTTCGTCGTGGCAACGTTGCTGGTGACAGCAAAAATGACCCACCAATGG  450
BX421307.2  80    ......................................................................................................................................................  229
BX443032.2  106   ..........M...........................................................................................................................................  255
BY794942.2  924   ......................................................................................................................................................  1073
BQ049312.1  78    ......................................................................................................................................................  227
BX417098.2  69    ......................................................................................................................................................  218
BX441194.2  118   ......................................................................................................................................................  267
DC527884.1  927   ......................................................................................................................................................  1076
AL541984.3  193   ......................................................................................................................................................  342
CR984260.1  96    ......................................................................................................................................................  245
BX440436.2  816   ......................................................................................................................................................  667

Query       451   AAGCAGCTGGCTTCACTGCTCAGGTGATTATCCTGAACCATCCAGGCCAAATAAGCGCCGGCTATGCCCTGTATTGGATTGCCACACGGCTCACATTGCATGCAAGTTTGCTGAGCTGAAGGAAAAGATTGATCGCCGTTCTGGTAAAAA  600
BX421307.2  230   ......................................................................................................................................................  380
                                                                                    \
                                                                                    |
                                                                                    C
BX443032.2  256   ......................................................................................................................................................  406
                                                                                    \
                                                                                    |
                                                                                    C
BY794942.2  1074  ......................................................................................................................................................  1225
                                                           \                        \
                                                           |                        |
                                                           G                        C
BQ049312.1  228   ......................................................................................................................................................  378
                                                                                    \
                                                                                    |
                                                                                    C
BX417098.2  219   ......................................................................................................................................................  369
                                                                                    \
                                                                                    |
                                                                                    C
BX441194.2  268   ......................................................................................................................................................  418
                                                                                    \
                                                                                    |
                                                                                    C
DC527884.1  1077  ......................................................................................................................................................  1227
                                                                                    \
                                                                                    |
                                                                                    C
AL541984.3  343   ......................................................................................................................................................  493
                                                                                    \
                                                                                    |
                                                                                    C
CR984260.1  246   ......................................................................................................................................................  396
                                                                                    \
                                                                                    |
                                                                                    C
BX440436.2  666   ......................................................................................................................................................  516
                                                                                       \
                                                                                       |
                                                                                       C

Query       601   GCTGGAAGATGGCCCTAAATTCTTGAAGTCTGGTGATGCTGCCATTGTTGATATGGTTCCTGGCAAGCCCATGTGTGTTGAGAGCTTCTCAGACTATCCACCTTTGGGTCGCTTTGCTGTTCGTGATATGAGACAGACAGTTGCGGTGGG  750
BX421307.2  381   ......................................................................................................................................................  530
BX443032.2  407   ......................................................................................................................................................  556
BY794942.2  1226  ......................................................................................................................................................  1375
BQ049312.1  379   ......................................................................................................................................................  528
BX417098.2  370   ......................................................................................................................................................  519
BX441194.2  419   ......................................................................................................................................................  568
DC527884.1  1228  ......................................................................................................................................................  1377
AL541984.3  494   ......................................................................................................................................................  643
CR984260.1  397   ......................................................................................................................................................  546
BX440436.2  515   ......................................................................................................................................................  366
```

```
Query       751   TGTCATCAAAGCAGTGGACAAGAAGGCTGCTGGAGCTGGCAAGGTCACCAAGTCTGCCCAGAAAGCTCAGAAGGCTAAATGAATATTATCCCTAATACCTGCCACCCCACTCTTAATCAGTGGTGGAAGAACGGTCTCAGAACTGTTTGT  900
BX421307.2  531   ......................................................................................................................................................  680
BX443032.2  557   ......................................................................................................................................................  706
BY794942.2  1376  ......................................................................................................................................................  1525
BQ049312.1  529   ......................................................................................................................................................  678
BX417098.2  520   ......................................................................................................................................................  669
BX441194.2  569   ......................................................................................................................................................  718
DC527884.1  1378  .A...............................................................................................................C....................................  1527
AL541984.3  644   ......................................................................................................................................................  793
CR984260.1  547   ......................................................................................................................................................  696
BX440436.2  365   ......................................................................................................................................................  216

Query       901   TTCAATTGGCCATTTAAGTTTAGTAGTAAAAGACTGGTTAATGATAACAATGCATCGTAAAACCTTCAGAAGGAAAGGAGAATgttttgtggaccactttggttttctttttttgcgtgtggcagttttaagttattagtttttaaaatca  1050
BX421307.2  681   ......................................................................................................................................................  830
BX443032.2  707   ......................................................................................................................................................  856
BY794942.2  1526  ......................................................................................................................................................  1675
BQ049312.1  679   ......................................................................................................................................................  828
BX417098.2  670   ......................................................................................................................................................  819
BX441194.2  719   ......................................................................................................................................................  868
DC527884.1  1528  ......................................................................................................................................................  1677
AL541984.3  794   .......................................................................................................................K.S............................  943
CR984260.1  697   .........................................................................................................................................N............  846
BX440436.2  215   ......................................................................................................................................................  66

Query       1051  gtacttttttaATGGAAACAACTTGACCAAAAATTTGTCACAGAATTTTGAGACCCATTAAAAAAGTT  1117
BX421307.2  831   ...................................................................  897
BX443032.2  857   ...................................................................  923
BY794942.2  1676  ...................................................................  1742
BQ049312.1  829   ...................................................................  896
                                                                     \
                                                                     |
                                                                     A
BX417098.2  820   ....................................................-..............  885
BX441194.2  869   ....................................................-..............  934
DC527884.1  1678  ...................................................................  1744
AL541984.3  944   ...................................................................  1011
                                                          \
                                                          |
                                                          M
CR984260.1  847   .........................................N..........-..............  912
BX440436.2  65    .....................................N..........................     1
                                                          \
                                                          |
                                                          N
```

Query= N25-C* [organism=Homo sapiens] eef1a1

Length=1832

Sequences producing significant alignments:	Score (Bits)	E Value	Max ident
BY794942.2 BY794942 Homo sapiens eye Homo sapiens cDNA clone H...	3147	0.0	100%
DC527884.1 DC527884 chimpanzee skin cDNA library PstA Pan trog...	3131	0.0	100%
CF111220.1 Shultzomica04471 Rat lung airway and parenchyma cDN...	2346	0.0	92%
BX426149.2 BX426149 Homo sapiens NEUROBLASTOMA Homo sapiens cD...	2146	0.0	99%
CO001006.1 OC028 pre-oestrum adult sheep ovary library Ovis ar...	2047	0.0	93%
BX418564.2 BX418564 Homo sapiens FETAL BRAIN Homo sapiens cDNA...	2047	0.0	97%
BX426150.2 BX426150 Homo sapiens NEUROBLASTOMA Homo sapiens cD...	2034	0.0	99%
AL567209.3 AL567209 Homo sapiens FETAL BRAIN Homo sapiens cDNA...	2001	0.0	98%
BX440470.1 BX440470 Homo sapiens FETAL BRAIN Homo sapiens cDNA...	1999	0.0	98%
BX420340.1 BX420340 Homo sapiens FETAL BRAIN Homo sapiens cDNA...	1997	0.0	99%

ALIGNMENTS

```
Query       124   CGCAGGTGTCGTGAAAACTACCCCTAAAAGCCAAAATGGGAAAGGAAAAGACTCATATCAACATTGTCGTCATTGGACACGTAGATTCGGGCAAGTCCACCACTACTGGCCATCTGATCTATAAATGCGGTGGCATCGACAAAAGAACCA  273
BY794942.2  35      ....................................................................................................................................................  182
DC527884.1  38      ....................................................................................................................................................  185
CF111220.1  25    .........T......G.C...G........A.......................C........C.....A.................C..............A..C.....C........C.....T.....A................  178
                                            \
                                            |
                                         ATTC
BX426150.2  6       ....................................................................................................................................................  153

Query       274   TTGAAAAATTTGAGAAGGAGGCTGCTGAGATGGGAAAGGGCTCCTTCAAGTATGCCTGGGTCTTGGATAAACTGAAAGCTGAGCGTGAACGTGGTATCACCATTGATATCTCCTTGTGGAAATTTGAGACCAGCAAGTACTATGTGACTA  423
BY794942.2  183   ......................................................................................................................................................  332
DC527884.1  186   .................................................................................................T....................................................  335
CF111220.1  179   .......G.................G...........A........................C....C....................G...........T.....C.....C........G............................  328
CO001006.1  1464                                                                                                                                               ....................T..C.  1440
BX426150.2  154   ......................................................................................................................................................  303
```

```
Query       424   TCATTGATGCCCCAGGACACAGAGACTTTATCAAAAACATGATTACAGGGACATCTCAGGCTGACTGTGCTGTCCTGATTGTTGCTGCTGGTGTTGGTGAATTTGAAGCTGGTATCTCCAAGAATGGGCAGACCCGAGAGCATGCCCTTC  573
BY794942.2  333   ......................................................................................................................................................  482
DC527884.1  336   ............................C.........................................................................................................................  485
CF111220.1  329   ............................C.....G...........G..C.................C.............................G..........................C...........T........T....  478
CO001006.1  1439  ............................C....................C.....C.....................................................C..............C...........T.............  1290
BX426150.2  304   ......................................................................................................................................................  453

Query       574   TGGCTTACACACTGGGTGTGAAACAACTAATTGTCGGTGTTAACAAAATGGATTCCACTGAGCCACCCTACAGCCAGAAGAGATATGAGGAAATTGTTAAGGAAGTCAGCACTTACATTAAGAAAATTGGCTACAACCCCGACACAGTAG  723
BY794942.2  483   ......................................................................................................................................................  632
DC527884.1  486   ......................................................................................................................................................  635
CF111220.1  479   ..........TT.............G........T.....C.................C........A.....T...........C........C.................C..........................T..........  628
BX426149.2  1196                                                ......-..A....-.A...............G........................................................................  1094
CO001006.1  1289  ..........C.......................T..C.................T..............T..............C..........................C.....................................  1140
BX418564.2  1196                                          ....-....-......-...-...-...YH.VS..-.R......-W.-..G..W...-..R......M......K...-.R.-......S....-...........-R..  1100
BX426150.2  454   ......................................................................................................................................................  603
AL567209.3  1145                                                                 .......S.....-...W-...........-K.W........R.....-...A.M.C.............C..............  1064
                                                                                                                                                      \
                                                                                                                                                      |
                                                                                                                                                      M
BX440470.1  1128                                                                                                                        ....-.....-..-W.-.............-.  1101
                                                                                                                                                                \
                                                                                                                                                                |
                                                                                                                                                                C
BX420340.1  1117                                                                                                                                   ...M..M..............  1097

Query       724   CATTTGTGCCAATTTCTGGTTGGAATGGTGACAACATGCTGGAGCCAAGTGCTAACATGCCTTGGTTCAAGGGATGGAAAGTCACCCGTAAGGATGGCAATGCCAGTGGAACCACGCTGCTTGAGGCTCTGGACTGCATCCTACCACCAA  873
BY794942.2  633   ......................................................................................................................................................  782
DC527884.1  636   ......................................................................................................................................................  785
CF111220.1  629   .......................................................T.....G..........................C..A.......G.........C...........G..A...T..........T..G..G....  778
BX426149.2  1093  ......................................................................................................................................................  944
CO001006.1  1139  ...................C...................................T.....A................................C....................C........A........T........G.......  990
BX418564.2  1099  ..-.................-.....-..........................C......................-.........................................................................  954
BX426150.2  604   ......................................................................................................................................................  753
AL567209.3  1063  ...................................................................................................................................K..................  914
BX440470.1  1100  ..-....-S.M...Y..-.K......-..............-.....................K...................................................................R..................  956
BX420340.1  1096  .....K.-..M.W...YT...D....K.-...........T..........Y...............................................................................A..................  949

Query       874   CTCGTCCAACTGACAAGCCCTTGCGCCTGCCTCTCCAGGATGTCTACAAAATTGGTGGTATTGGTACTGTTCCTGTTGGCCGAGTGGAGACTGGTGTTCTCAAACCCGGTATGGTGGTCACCTTTGCTCCAGTCAACGTTACAACGGAAG  1023
BY794942.2  783   ......................................................................................................................................................  932
DC527884.1  786   ......................................................................................................................................................  935
CF111220.1  779   ...................TC....A.....C..............T........C.C...C........C.....G...........A.................T...........T.................T..A.....T....  928
BX426149.2  943   ......................................................................................................................................................  794
CO001006.1  989   ....C...........A........TT...................T.......................A.....G..T..T.......................T..C.................C........T..A.....T..G.  840
BX418564.2  953   ....................C.....................................................................................................................................  804
BX426150.2  754   ......................................................................................................................................................  903
AL567209.3  913   ......................................................................................................................................................  764
BX440470.1  955   ......................................................................................................................................................  806
BX420340.1  948   ...........R..........................................................................................................................................  799

Query       1024  TAAAATCTGTCGAAATGCACCATGAAGCTTTGAGTGAAGCTCTTCCTGGGGACAATGTGGGCTTCAATGTCAAGAATGTGTCTGTCAAGGATGTTCGTCGTGGCAACGTTGCTGGTGACAGCAAAAATGACCCACCAATGGAAGCAGCTG  1173
BY794942.2  933   ......................................................................................................................................................  1082
DC527884.1  936   ......................................................................................................................................................  1085
CF111220.1  929   .C..G.....G................................G..............A........C..A.....C...........A..C...A.A........T........G..................................  1078
BX426149.2  793   ......................................................................................................................................................  644
CO001006.1  839   .G..G..C..A.............................C...................................C...........A.....C...........T..G....................T.....C........T....  690
BX418564.2  803   ....................................................................................C.................................................................  654
BX426150.2  904   ........................................................................................................-...............-...........-.........-.......  1050
                                                                 \
                                                                 |
                                                                 G
AL567209.3  763   ..............................................................................................................................-.......................  615
BX440470.1  805   ......................................................................................................................................................  656
BX420340.1  798   ......................................................................................................................................................  649

Query       1174  GCTTCACTGCTCAGGTGATTATCCTGAACCATCCAGGCCAAATAAGCGCCGGCTATGCCCCTGTATTGGATTGCCACACGGCTCACATTGCATGCAAGTTTGCTGAGCTGAAGGAAAAGATTGATCGCCGTTCTGGTAAAAAGCTGGAAG  1323
BY794942.2  1083  ......................................................................................................................................................  1233
                                                  \
                                                  |
                                                  G
DC527884.1  1086  ......................................................................................................................................................  1235
CF111220.1  1079  ........................................G..C..T..T..............TC....C...........C.....A..............C.....T..A..G.....C.....T...........G..........  1228
BX426149.2  643   ......................................................................................................................................................  494
CO001006.1  689   ..C....A..............T....................C..T.GT..A.....A.....GC.......T.....A...........T.......................G...........T........G.............  540
BX418564.2  653   .....................................................................................................................M................................  504
BX426150.2  1051  ..-...................-..................-.......-.......-................-......T.-.............                                                     1140
AL567209.3  614   ......................................................................................................................................................  465
BX440470.1  655   ......................................................................................................................................................  505
                                                                                                                                             \
                                                                                                                                             |
                                                                                                                                             C
BX420340.1  648   ......................................................................................................................................................  499
```

```
Query       1324  ATGGCCCTAAATTCTTGAAGTCTGGTGATGCTGCCATTGTTGATATGGTTCCTGGCAAGCCCATGTGTGTTGAGAGCTTCTCAGACTATCCACCTTTGGGTCGCTTTGCTGTTCGTGATATGAGACAGACAGTTGCGGTGGGTGTCATCA  1473
BY794942.2  1234  ......................................................................................................................................................  1383
DC527884.1  1236  ...............................................................................................................................................A......  1385
CF111220.1  1229  .......C...................................C.....C.......................A........T.....C..T.AC..T.....T..............C.....G...........T.............  1378
BX426149.2  493   ......................................................................................................................................................  344
CO001006.1  539   ...................A........C........C.......................T........C........T..T..T.....T..C.....C..T........G.....C..............C..T.............  390
BX418564.2  503   ......................................................................................................................................................  354
AL567209.3  464   .................................................K....................................................................................................  315
BX440470.1  504   ......................................................................................................................................................  355
BX420340.1  498   ......................................................................................................................................................  349

Query       1474  AAGCAGTGGACAAGAAGGCTGCTGGAGCTGGCAAGGTCACCAAGTCTGCCCAGAAAGCTCAGAAGGCTAAATGAATATTATCCCTAATACCTGCCACCCCACTCTTAATCAGTGGTGGAAGAACGGTCTCAGAACTGTTTGTTTCAATTG  1623
BY794942.2  1384  ......................................................................................................................................................  1533
DC527884.1  1386  .........................................................................................................C............................................  1535
CF111220.1  1379  ....C.................A...........A....................................................C.............G........................................C.......  1528
BX426149.2  343   ......................................................................................................................................................  194
CO001006.1  389   ...................A................................................................C................G........................................C.......  240
BX418564.2  353   ......................................................................................................................................................  204
AL567209.3  314   ............................................................M......Y..................................................................................  164
                                                                                                                                    \
                                                                                                                                    |
                                                                                                                                    A
BX440470.1  354   ......................................................................................................................................................  205
BX420340.1  348   ......................................................................................................................................................  199

Query       1624  GCCATTTAAGTTTAGTAGTAAAAGACTGGTTAATGATAACAATGCATCGTAAAACCTTCAGAAGGAAAGGAGAATgttttgtggaccactttggttttctttttttgcgtgtggcagttttaagttattagtttttaaaatcagtactttt  1773
BY794942.2  1534  ......................................................................................................................................................  1683
DC527884.1  1536  ......................................................................................................................................................  1685
CF111220.1  1529  ..............A....................................................---..................-...--....-..G.----...........................C...............  1667
BX426149.2  193   ......................................................................................................................................................  44
CO001006.1  239   ..............A.........................................................................-.A..-....G.-----.--.........-................................  99
                                                                                                  \
                                                                                                  |
                                                                                                  T
BX418564.2  203   ......................................................................................................................................................  54
AL567209.3  163   ......................................................................................................................................................  13
                                                                                                        \
                                                                                                        |
                                                                                                        G
BX440470.1  204   ......................................................................................................................................................  55
BX420340.1  198   ......................................................................................................................................................  49

Query       1774  taATGGAAACAACTTGACCAAAAATTTGTCACAGAATTTTGAGACCCATTAAAAAAGTT  1832
BY794942.2  1684  ...........................................................  1742
DC527884.1  1686  ...........................................................  1744
CF111220.1  1668  .........................C....................-............  1726
                                                                        \
                                                                        |
                                                                        C
BX426149.2  43    .....-.......-.....G......G.-..G...........T..               1
CO001006.1  98    .....A...................C.............-...................  40
                                                                          \
                                                                          |
                                                                          A
BX418564.2  53    .......................................M-.............       1
AL567209.3  12    -....-..-...-...                                             1
BX440470.1  54    ......................................................       1
BX420340.1  48    ..........................................R....              1
```

Query= N26-20 [organism=Homo sapiens] eef1a1

Length=1067

```
                                                                   Score        E     Max
Sequences producing significant alignments:                        (Bits)     Value  ident

BX421307.2 BX421307 Homo sapiens B CELLS (RAMOS CELL LINE) Hom...  1592       0.0    100%
BX443032.2 BX443032 Homo sapiens B CELLS (RAMOS CELL LINE) Hom...  1589       0.0    100%
BY794942.2 BY794942 Homo sapiens eye Homo sapiens cDNA clone H...  1589       0.0    100%
BQ049312.1 AGENCOURT_6795094 NIH_MGC_85 Homo sapiens cDNA clon...  1589       0.0    100%
BX417098.2 BX417098 Homo sapiens PLACENTA Homo sapiens cDNA cl...  1587       0.0    100%
BX441194.2 BX441194 Homo sapiens FETAL BRAIN Homo sapiens cDNA...  1583       0.0    100%
BX440436.2 BX440436 Homo sapiens FETAL BRAIN Homo sapiens cDNA...  1583       0.0    100%
BX440470.1 BX440470 Homo sapiens FETAL BRAIN Homo sapiens cDNA...  1583       0.0    100%
DC527884.1 DC527884 chimpanzee skin cDNA library PstA Pan trog...  1581       0.0    100%
AL541984.3 AL541984 Homo sapiens PLACENTA Homo sapiens cDNA cl...  1581       0.0    100%
```

ALIGNMENTS

```
Query       1     GAAAGGAAAAGACTCATATCAACATTGTCGTCATTGGACACGTAGATTCGGGCAAGTCCACCACTACTGGCCATCTGATCTATAAATGCGGTGGCATCGACAAAAGAACCATTGAAAAATTTGAGAAGGAGGCTGCTGAGATGGGAAAGG  150
BY794942.2  72    ......................................................................................................................................................  221
```

```
DC527884.1  75    ......................................................................................................................................................  224

Query       151   GCTCCTTCAAGTATGCCTGGGTCTTGGATAAACTGAAAGCTGAGCGTGAACGTGGTGTTCTCAAACCCGGTATGGTGGTCACCTTTGCTCCAGTCAACGTTACAACGGAAGTAAAATCTGTCGAAATGCACCATGAAGCTTTGAGTGAAG  300
BX421307.2  30                                                        ..................................................................................................  127
BX443032.2  56                                                        ............................................................M.....................................  153
BY794942.2  874                                                       ..................................................................................................  971
BY794942.2  222   ........................................................                                                                                              277
BQ049312.1  28                                                        ..................................................................................................  125
BX417098.2  19                                                        ..................................................................................................  116
BX441194.2  68                                                        .......................W..........................................................................  165
BX440436.2  866                                                       ..................................................................................................  769
BX440470.1  864                                                       ..................................................................................................  767
DC527884.1  877                                                       ..................................................................................................  974
DC527884.1  225   ........................................................                                                                                              280
AL541984.3  143                                                       ..................................................................................................  240

Query       301   CTCTTCCTGGGGACAATGTGGGCTTCAATGTCAAGAATGTGTCTGTCAAGGATGTTCGTCGTGGCAACGTTGCTGGTGACAGCAAAAATGACCCACCAATGGAAGCAGCTGGCTTCACTGCTCAGGTGATTATCCTGAACCATCCAGGCC  450
BX421307.2  128   ......................................................................................................................................................  277
BX443032.2  154   ......................................................................................................................................................  303
BY794942.2  972   ......................................................................................................................................................  1122
                                                                                                                                                                 \
                                                                                                                                                                 |
                                                                                                                                                                 G
BQ049312.1  126   ......................................................................................................................................................  275
BX417098.2  117   ......................................................................................................................................................  266
BX441194.2  166   ......................................................................................................................................................  315
BX440436.2  768   ......................................................................................................................................................  619
BX440470.1  766   ......................................................................................................................................................  617
DC527884.1  975   ......................................................................................................................................................  1124
AL541984.3  241   ......................................................................................................................................................  390

Query       451   AAATAAGCGCCGGCTATGCCCCTGTATTGGATTGCCACACGGCTCACATTGCATGCAAGTTTGCTGAGCTGAAGGAAAAGATTGATCGCCGTTCTGGTAAAAAGCTGGAAGATGGCCCTAAATTCTTGAAGTCTGGTGATGCTGCCATTG  600
BX421307.2  278   ......................................................................................................................................................  427
BX443032.2  304   ......................................................................................................................................................  453
BY794942.2  1123  ......................................................................................................................................................  1272
BQ049312.1  276   ......................................................................................................................................................  425
BX417098.2  267   ......................................................................................................................................................  416
BX441194.2  316   ......................................................................................................................................................  465
BX440436.2  618   ......................................................................................................................................................  469
BX440470.1  616   ......................................................................................................................................................  466
                                                                                                  \
                                                                                                  |
                                                                                                  C
DC527884.1  1125  ......................................................................................................................................................  1274
AL541984.3  391   ......................................................................................................................................................  540

Query       601   TTGATATGGTTCCTGGCAAGCCCATGTGTGTTGAGAGCTTCTCAGACTATCCACCTTTGGGTCGCTTTGCTGTTCGTGATATGAGACAGACAGTTGCGGTGGGTGTCATCAAAGCAGTGGACAAGAAGGCTGCTGGAGCTGGCAAGGTCA  750
BX421307.2  428   ......................................................................................................................................................  577
BX443032.2  454   ......................................................................................................................................................  603
BY794942.2  1273  ......................................................................................................................................................  1422
BQ049312.1  426   ......................................................................................................................................................  575
BX417098.2  417   ......................................................................................................................................................  566
BX441194.2  466   ......................................................................................................................................................  615
BX440436.2  468   ......................................................................................................................................................  319
BX440470.1  465   ......................................................................................................................................................  316
DC527884.1  1275  ........................................................................................................A.............................................  1424
AL541984.3  541   ......................................................................................................................................................  690

Query       751   CCAAGTCTGCCAGAAAGCTCAGAAGGCTAAATGAATATTATCCCTAATACCTGCCACCCCACTCTTAATCAGTGGTGGAAGAACGGTCTCAGAACTGTTTGTTTCAATTGGCCATTTAAGTTTAGTAGTAAAAGACTGGTTAATGATAAC  900
BX421307.2  578   ......................................................................................................................................................  728
                           \
                           |
                           C
BX443032.2  604   ......................................................................................................................................................  754
                           \
                           |
                           C
BY794942.2  1423  ......................................................................................................................................................  1573
                           \
                           |
                           C
BQ049312.1  576   ......................................................................................................................................................  726
                           \
                           |
                           C
BX417098.2  567   ......................................................................................................................................................  717
                           \
                           |
                           C
BX441194.2  616   ......................................................................................................................................................  766
                           \
                           |
```

```
                           C
BX440436.2  318   ......................................................................................................................................................  168
                             \
                             |
                             C
BX440470.1  315   ......................................................................................................................................................  165
                             \
                             |
                             C
DC527884.1  1425  .................................................................C....................................................................................  1575
                           \
                           |
                           C
AL541984.3  691   ......................................................................................................................................................  841
                           \
                           |
                           C

Query       901   AATGCATCGTAAAACCTTCAGAAGGAAAGGAGAATgttttgtggaccactttggttttctttttttgcgtgtggcagttttaagttattagtttttaaaatcagtactttttaATGGAAACAACTTGACCAAAAATTTGTCACAGAATTTT  1050
BX421307.2  729   ......................................................................................................................................................  878
BX443032.2  755   ......................................................................................................................................................  904
BY794942.2  1574  ......................................................................................................................................................  1723
BQ049312.1  727   ......................................................................................................................................................  876
BX417098.2  718   ......................................................................................................................................................  867
BX441194.2  767   ......................................................................................................................................................  916
BX440436.2  167   ...........................................................................................................................................N..........  17
                                                                                                                                                                \
                                                                                                                                                                |
                                                                                                                                                                N
BX440470.1  164   ......................................................................................................................................................  15
DC527884.1  1576  ......................................................................................................................................................  1725
AL541984.3  842   .......................................................................K.S............................................................................  992
                                                                                                                                                                \
                                                                                                                                                                |
                                                                                                                                                                M

Query       1051  GAGACCCATTAAAAAAG  1067
BX421307.2  879   .................  895
BX443032.2  905   .................  921
BY794942.2  1724  .................  1740
BQ049312.1  877   .................  894
                     \
                     |
                     A
BX417098.2  868   ......-..........  883
BX441194.2  917   ......-..........  932
BX440436.2  16    ................   1
BX440470.1  14    ..............     1
DC527884.1  1726  .................  1742
AL541984.3  993   .................  1009
```

Query= N27-C* [organism=Homo sapiens] eef1a1

Length=1807

```
                                                                  Score        E     Max
Sequences producing significant alignments:                      (Bits)     Value  ident

BY794942.2 BY794942 Homo sapiens eye Homo sapiens cDNA clone H...  3134       0.0    100%
DC527884.1 DC527884 chimpanzee skin cDNA library PstA Pan trog...  3116       0.0    100%
CF111220.1 Shultzomica04471 Rat lung airway and parenchyma cDN...  2331       0.0    92%
BX426149.2 BX426149 Homo sapiens NEUROBLASTOMA Homo sapiens cD...  2143       0.0    99%
CO001006.1 OC028 pre-oestrum adult sheep ovary library Ovis ar...  2041       0.0    93%
BX418564.2 BX418564 Homo sapiens FETAL BRAIN Homo sapiens cDNA...  2041       0.0    97%
BX426150.2 BX426150 Homo sapiens NEUROBLASTOMA Homo sapiens cD...  2028       0.0    99%
AL567209.3 AL567209 Homo sapiens FETAL BRAIN Homo sapiens cDNA...  2001       0.0    98%
BX440470.1 BX440470 Homo sapiens FETAL BRAIN Homo sapiens cDNA...  1993       0.0    98%
BX420340.1 BX420340 Homo sapiens FETAL BRAIN Homo sapiens cDNA...  1991       0.0    98%
```

ALIGNMENTS

```
Query       105   GTGTCGTGAAAACTACCCCTAAAAGCCAAAATGGGAAAGGAAAAGACTCATATCAACATTGTCGTCATTGGACACGTAGATTCGGGCAAGTCCACCACTACTGGCCATCTGATCTATAAATGCGGTGGCATCGACAAAAGAACCATTGAA  254
BY794942.2  38    ......................................................................................................................................................  187
DC527884.1  41    ......................................................................................................................................................  190
CF111220.1  55                         .....A.......................C........C.....A.................C..............A..C.....C........C....T.....A......................  183
BX426150.2  9     ......................................................................................................................................................  158

Query       255   AAATTTGAGAAGGAGGCTGCTGAGATGGGAAAGGGCTCCTTCAAGTATGCCTGGGTCTTGGATAAACTGAAAGCTGAGCGTGAACGTGGTATCACCATTGATATCTCCTTGTGGAAATTTGAGACCAGCAAGTACTATGTGACTATCATT  404
BY794942.2  188   ......................................................................................................................................................  337
DC527884.1  191   ............................................................................................T.........................................................  340
CF111220.1  184   ..G.................G...........A........................C...C.....................G...........T.....C......C......G..................................  333
```

```
CO001006.1  1464                                                                                                                          ....................T..C......  1435
BX426150.2  159   ......................................................................................................................................................  308

Query       405   GATGCCCCAGGACACAGAGACTTTATCAAAAACATGATTACAGGGACATCTCAGGCTGACTGTGCTGTCCTGATTGTTGCTGCTGGTGTTGGTGAATTTGAAGCTGGTATCTCCAAGAATGGGCAGACCCGAGAGCATGCCCTTCTGGCT  554
BY794942.2  338   ......................................................................................................................................................  487
DC527884.1  341   .......................C..............................................................................................................................  490
CF111220.1  334   .......................C.....G...........G..C.................C.............................G..........................C...........T........T.........  483
CO001006.1  1434  .......................C....................C.....C.....................................................C..............C...........T..................  1285
BX426150.2  309   ......................................................................................................................................................  458

Query       555   TACACACTGGGTGTGAAACAACTAATTGTCGGTGTTAACAAAATGGATTCCACTGAGCCACCCTACAGCCAGAAGAGATATGAGGAAATTGTTAAGGAAGTCAGCACTTACATTAAGAAAATTGGCTACAACCCCGACACAGTAGCATTT  704
BY794942.2  488   ......................................................................................................................................................  637
DC527884.1  491   ......................................................................................................................................................  640
CF111220.1  484   .....TT.............G........T.....C.................C........A.....T...........C........C.................C..........................T...............  633
BX426149.2  1196                                          ......-..A....-.A...............G.............................................................................  1089
CO001006.1  1284  .....C.......................T..C.................T..............T..............C..........................C..........................................  1135
BX418564.2  1196                                     ....-....-......-...-...-...YH.VS..-.R......-W.-..G..W....-.R......M.....K....-.R.-......S....-..........-R.....-..  1096
BX426150.2  459   ......................................................................................................................................................  608
AL567209.3  1145                                                              .......S.....-...W.-..........-K.W........R.....-....A.M.C............C...................  1059
                                                                                                                                 \
                                                                                                                                 |
                                                                                                                                 M
BX440470.1  1128                                                                                                                   ....-.....-..-W.-.............-...-..  1097
                                                                                                                                         \
                                                                                                                                         |
                                                                                                                                         C
BX420340.1  1117                                                                                                                              ...M..M...................  1092

Query       705   GTGCCAATTTCTGGTTGGAATGGTGACAACATGCTGGAGCCAAGTGCTAACATGCCTTGGTTCAAGGGATGGAAAGTCACCCGTAAGGATGGCAATGCCAGTGGAACCACGCTGCTTGAGGCTCTGGACTGCATCCTACCACCAACTCGT  854
BY794942.2  638   ......................................................................................................................................................  787
DC527884.1  641   ......................................................................................................................................................  790
CF111220.1  634   ..................................................T.....G..........................C..A.......G.........C...........G..A...T..........T..G..G.........  783
BX426149.2  1088  ......................................................................................................................................................  939
CO001006.1  1134  ..............C...................................T.....A................................C....................C........A........T........G...........C  985
BX418564.2  1095  ...............-.....-..........................C......................-..............................................................................  949
BX426150.2  609   ......................................................................................................................................................  758
AL567209.3  1058  ..............................................................................................................................K.......................  909
BX440470.1  1096  ..-S.M...Y..-.K......-..............-.....................K...................................................................R.......................  951
BX420340.1  1091  K.-..M.W...YT...D....K.-...........T..........Y...............................................................................A.......................  944

Query       855   CCAACTGACAAGCCCTTGCGCCTGCCTCTCCAGGATGTCTACAAAATTGGTGGTATTGGTACTGTTCCTGTTGGCCGAGTGGAGACTGGTGTTCTCAAACCCGGTATGGTGGTCACCTTTGCTCCAGTCAACGTTACAACGGAAGTAAAA  1004
BY794942.2  788   ......................................................................................................................................................  937
DC527884.1  791   ......................................................................................................................................................  940
CF111220.1  784   ..............TC....A.....C..............T........C..C.....C.....C.....G...........A.................T...........T.................T..A.....T.....C..G  933
BX426149.2  938   ......................................................................................................................................................  789
CO001006.1  984   ...........A........TT...................T.......................A.....G..T..T.......................T..C.................C........T..A.....T..G..G..G  835
BX418564.2  948   ...............C......................................................................................................................................  799
BX426150.2  759   ......................................................................................................................................................  908
AL567209.3  908   ......................................................................................................................................................  759
BX440470.1  950   ......................................................................................................................................................  801
BX420340.1  943   ......R...............................................................................................................................................  794

Query       1005  TCTGTCGAAATGCACCATGAAGCTTTGAGTGAAGCTCTTCCTGGGGACAATGTGGGCTTCAATGTCAAGAATGTGTCTGTCAAGGATGTTCGTCGTGGCAACGTTGCTGGTGACAGCAAAAATGACCCACCAATGGAAGCAGCTGGCTTC  1154
BY794942.2  938   ......................................................................................................................................................  1087
DC527884.1  941   ......................................................................................................................................................  1090
CF111220.1  934   .....G................................G..............A........C..A.....C...........A..C...A.A........T........G.......................................  1083
BX426149.2  788   ......................................................................................................................................................  639
CO001006.1  834   ..C..A.............................C...................................C...........A.....C...........T..G....................T.....C........T......C..  685
BX418564.2  798   ...............................................................................C......................................................................  649
BX426150.2  909   ...................................................................................................-...............-...........-.........-.........-..  1054
                                                            \
                                                            |
                                                            G
AL567209.3  758   .........................................................................................................................-............................  610
BX440470.1  800   ......................................................................................................................................................  651
BX420340.1  793   ......................................................................................................................................................  644

Query       1155  ACTGCTCAGGTGATTATCCTGAACCATCCAGGCCAAATAAGCGCCGGCTATGCCCCTGTATTGGATTGCCACACGGCTCACATTGCATGCAAGTTTGCTGAGCTGAAGGAAAAGATTGATCGCCGTTCTGGTAAAAAGCTGGAAGATGGC  1304
BY794942.2  1088  ......................................................................................................................................................  1238
                                             \
                                             |
                                             G
DC527884.1  1091  ......................................................................................................................................................  1240
CF111220.1  1084  ...................................G..C..T..T..............TC....C...........C.....A..............C.....T..A..G.....C.....T...........G...............  1233
BX426149.2  638   ......................................................................................................................................................  489
CO001006.1  684   ..A..............T....................C..T.GT..A.....A.....GC.......T.....A...........T.......................G...........T........G..................  535
BX418564.2  648   ................................................................................................................M.....................................  499
BX426150.2  1055  .................-..................-.......-.......-................-......T.-.............                                                          1140
AL567209.3  609   ......................................................................................................................................................  460
BX440470.1  650   ......................................................................................................................................................  500
                                                                                                                                
                                                                                                                                    \
                                                                                                                                    |
```

```
                                                                                                                                    C
BX420340.1  643   ......................................................................................................................................................  494

Query       1305  CCTAAATTCTTGAAGTCTGGTGATGCTGCCATTGTTGATATGGTTCCTGGCAAGCCCATGTGTGTTGAGAGCTTCTCAGACTATCCACCTTTGGGTCGCTTTGCTGTTCGTGATATGAGACAGACAGTTGCGGTGGGTGTCATCAAAGCA  1454
BY794942.2  1239  ......................................................................................................................................................  1388
DC527884.1  1241  ..........................................................................................................................................A...........  1390
CF111220.1  1234  ..C...................................C.....C.......................A........T.....C..T....AC.T...T..............C....G............T.................C  1383
BX426149.2  488   ......................................................................................................................................................  339
CO001006.1  534   ..............A........C........C.......................T........C........T..T..T.....T..C.....C..T........G.....C..............C..T..................  385
BX418564.2  498   ......................................................................................................................................................  349
AL567209.3  459   ............................................K.........................................................................................................  310
BX440470.1  499   ......................................................................................................................................................  350
BX420340.1  493   ......................................................................................................................................................  344

Query       1455  GTGGACAAGAAGGCTGCTGGAGCTGGCAAGGTCACCAAGTCTGCCCAGAAAGCTCAGAAGGCTAAATGAATATTATCCCTAATACCTGCCACCCCACTCTTAATCAGTGGTGGAAGAACGGTCTCAGAACTGTTTGTTTCAATTGGCCAT  1604
BY794942.2  1389  ......................................................................................................................................................  1538
DC527884.1  1391  ....................................................................................................C.................................................  1540
CF111220.1  1384  .................A...........A....................................................C.............G........................................C............  1533
BX426149.2  338   ......................................................................................................................................................  189
CO001006.1  384   ..............A................................................................C................G........................................C............  235
BX418564.2  348   ......................................................................................................................................................  199
AL567209.3  309   .......................................................M......Y.......................................................................................  159
                                                                                                                               \
                                                                                                                               |
                                                                                                                               A
BX440470.1  349   ......................................................................................................................................................  200
BX420340.1  343   ......................................................................................................................................................  194

Query       1605  TTAAGTTTAGTAGTAAAAGACTGGTTAATGATAACAATGCATCGTAAAACCTTCAGAAGGAAAGGAGAATgttttgtggaccactttggttttctttttttgcgtgtggcagttttaagttattagtttttaaaatcagtactttttaATG  1754
BY794942.2  1539  ......................................................................................................................................................  1688
DC527884.1  1541  ......................................................................................................................................................  1690
CF111220.1  1534  .........A....................................................---..................-...--....-..G.----...........................C....................  1672
BX426149.2  188   ......................................................................................................................................................  39
CO001006.1  234   .........A.........................................................................-.A..-....G.-----.--........-......................................  94
                                                                                             \
                                                                                             |
                                                                                             T
BX418564.2  198   ......................................................................................................................................................  49
AL567209.3  158   .................................................................................................................................................-....  9
                                                                                                   \
                                                                                                   |
                                                                                                   G
BX440470.1  199   ......................................................................................................................................................  50
BX420340.1  193   ......................................................................................................................................................  44

Query       1755  GAAACAACTTGACCAAAAATTTGTCACAGAATTTTTGAGACCCATTAAAAAAG  1807
BY794942.2  1689  ...............................-.....................  1740
DC527884.1  1691  ...............................-.....................  1742
CF111220.1  1673  ....................C..............-......-..........  1724
                                                                    \
                                                                    |
                                                                    C
BX426149.2  38    -.......-.....G......G.-..G........-...T..             1
CO001006.1  93    A...................C.............--................   44
BX418564.2  48    ..................................M--.............     1
AL567209.3  8     -..-...-...                                            1
BX440470.1  49    ...................................-..............     1
BX420340.1  43    ...................................-..R.....           1
```

Query= N28-13 [organism=Homo sapiens] eef1a1

Length=1189

```
                                                                  Score        E     Max
Sequences producing significant alignments:                       (Bits)     Value  ident

DC527884.1 DC527884 chimpanzee skin cDNA library PstA Pan trog...  2182       0.0    100%
BY794942.2 BY794942 Homo sapiens eye Homo sapiens cDNA clone H...  2152       0.0    100%
BX426150.2 BX426150 Homo sapiens NEUROBLASTOMA Homo sapiens cD...  2037       0.0    99%
BU902062.1 AGENCOURT_10127553 NIH_MGC_71 Homo sapiens cDNA clo...  1999       0.0    98%
BX402801.2 BX402801 Homo sapiens T CELLS (JURKAT CELL LINE) CO...  1988       0.0    98%
AL513590.3 AL513590 Homo sapiens PLACENTA Homo sapiens cDNA cl...  1977       0.0    97%
BX402793.2 BX402793 Homo sapiens T CELLS (JURKAT CELL LINE) CO...  1973       0.0    98%
BX440468.2 BX440468 Homo sapiens FETAL BRAIN Homo sapiens cDNA...  1971       0.0    98%
AL513664.3 AL513664 Homo sapiens NEUROBLASTOMA Homo sapiens cD...  1962       0.0    98%
DC852462.1 DC852462 macaque spleen cDNA library QspA Macaca fa...  1960       0.0    97%
```

ALIGNMENTS

```
Query       2     CTTTTTCGCAACGGGTTTGCCGCCAGAACACAGGTGTCGTGAAAACTACCCCTAAAGCCAAAATGGGAAAGGAAAAGACTCATATCAACATTGTCGTCATTGGACACGTAGATTCGGGCAAGTCCACCACTACTGGCCATCTGATCTAT  151
DC527884.1  9      .....................................................................................................................................................  157
```

```
BY794942.2 25                        ..................................................................................................................................  154
BX426150.2 1                              ..T..........................................................................................................................  125
BU902062.1 1                ........................................................................................................................................  136
BX402801.2 1             ....A......................................................................................................................................  139
AL513590.3 2                                ........................................................................................................................  122
                                                                                                \
                                                                                                |
                                                                                                C
BX402793.2 6                        .....................................................................................................................................  138
BX440468.2 1                         .................................................................................................................................  129
AL513664.3 1                         .................................................................................................................................  129
DC852462.1 1      .....................................................................................................................................................C  150

Query      152   AAATGCGGTGGCATCGACAAAAGAACCATTGAAAAATTTGAGAAGGAGGCTGCTGAGATGGGAAAGGGCTCCTTCAAGTATGCCTGGGTCTTGGATAAACTGAAAGCTGAGCGTGAACGTGGTATCACCATTGATATCTCCTTGTGGAAA  301
DC527884.1 158   .............................................................................................................................T........................  307
BY794942.2 155   ......................................................................................................................................................  304
BX426150.2 126   ......................................................................................................................................................  275
BU902062.1 137   ......................................................................................................................................................  286
BX402801.2 140   ......................................................................................................................................................  289
AL513590.3 123   ...........................................................................................................B..........................................  272
BX402793.2 139   ......................................................................................................................................................  288
BX440468.2 130   ......................................................................................................................................................  279
AL513664.3 130   .............................................................................................................................Y........................  279
DC852462.1 151   .................................................................................................................G....................................  300

Query      302   TTTGAGACCAGCAAGTACTATGTGACTATCATTGATGCCCCAGGACACAGAGACTTTATCAAAAACATGATTACAGGGACATCTCAGGCTGACTGTGCTGTCCTGATTGTTGCTGCTGGTGTTGGTGAATTTGAAGCTGGTATCTCCAAG  451
DC527884.1 308   ........................................................C.............................................................................................  457
BY794942.2 305   ......................................................................................................................................................  454
BX426150.2 276   ......................................................................................................................................................  425
BU902062.1 287   ......................................................................................................................................................  436
BX402801.2 290   ......................................................................................................................................................  439
AL513590.3 273   ..............................................................................................................K..B..B.....K...........................  422
BX402793.2 289   ......................................................................................................................................................  438
BX440468.2 280   ......................................................................................................................................................  429
AL513664.3 280   ....................................................................................................................Y.................................  429
DC852462.1 301   .................T......................................C.............................................................................................  450

Query      452   AATGGGCAGACCCGAGAGCATGCCCTTCTGGCTTACACACTGGGTGTGAAACAACTAATTGTCGGTGTTAACAAAATGGATTCCACTGAGCCACCCTACAGCCAGAAGAGATATGAGGAAATTGTTAAGGAAGTCAGCACTTACATTAAG  601
DC527884.1 458   ......................................................................................................................................................  607
BY794942.2 455   ......................................................................................................................................................  604
BX426150.2 426   ......................................................................................................................................................  575
BU902062.1 437   ......................................................................................................................................................  586
BX402801.2 440   ......................................................................................................................................................  589
AL513590.3 423   ......................................................................................................................................................  572
BX402793.2 439   ......................................................................................................................................................  588
BX440468.2 430   ......................................................................................................................................................  579
AL513664.3 430   ......................................................................................................................................................  579
DC852462.1 451   .......................T......................................T..................................................C....................................  600

Query      602   AAAATTGGCTACAACCCCGACACAGTAGCATTTGTGCCAATTTCTGGTTGGAATGGTGACAACATGCTGGAGCCAAGTGCTAACATGCCTTGGTTCAAGGGATGGAAAGTCACCCGTAAGGATGGCAATGCCAGTGGAACCACGCTGCTT  751
DC527884.1 608   ......................................................................................................................................................  757
BY794942.2 605   ......................................................................................................................................................  754
BX426150.2 576   ......................................................................................................................................................  725
BU902062.1 587   ......................................................................................................................................................  736
BX402801.2 590   ......................................................................................................................................................  739
AL513590.3 573   ...................................................................Y............Y......................................-...............T..............  721
BX402793.2 589   ......................................................................................................................................................  738
BX440468.2 580   .......................................................................................................................-..............................  728
AL513664.3 580   .......................................................................................................................................K..............  729
DC852462.1 601   ...........................................................................................................G..........................................  750

Query      752   GAGGCTCTGGACTGCATCCTACCACCAACTCGTCCAACTGACAAGCCCTTGCGCCTGCCTCTCCAGGATGTCTACAAAATTGGTGGTATTGGTACTGTTCCTGTTGGCCGAGTGGAGACTGGTGTTCTCAAACCCGGTATGGTGGTCACC  901
DC527884.1 758   ......................................................................................................................................................  907
BY794942.2 755   ......................................................................................................................................................  904
BX426150.2 726   ......................................................................................................................................................  875
BU902062.1 737   ...................................................................................................................................C..G...............  886
BX402801.2 740   ......................................................................................................................................................  889
AL513590.3 722   ........................................................................................................Y.............................................  871
BX402793.2 739   ......................................................................................................................................................  888
BX440468.2 729   ......................................................................................................................................................  878
AL513664.3 730   ......................................................................................................................................................  879
DC852462.1 751   ......................................................................................................................................................  900

Query      902   TTTGCTCCAGTCAACGTTACAACGGAAGTAAAATCTGTCGAAATGCACCATGAAGCTTTGAGTGAAGCTCTTCCTGGGGACAATGTGGGCTTCAATGTCAAGAATGTGTCTGTCAAGGATGTTCGTCGTGGCAACGTTGCTGGTGACAGC  1051
DC527884.1 908   ......................................................................................................................................................  1057
BY794942.2 905   ......................................................................................................................................................  1054
BX426150.2 876   ....................................................................................................................................-...............-.  1024
                                                                                            \
                                                                                            |
                                                                                            G
BU902062.1 887   .............................-..................................................AC...........C...C..........................C..C......................  1036
                                                                                                                                                  \
```

```
                                                                                                                                                   |
                                                                                                                                                   G
BX402801.2  890   .............................W......................................Y.......................Y.....T............K.T..A................M.K............S.  1041
                                                                                                        \                                          \
                                                                                                        |                                          |
                                                                                                        G                                          K
AL513590.3  872   ...........T.................T........Y........................R..................................M............T....................-...............C.  1020
BX402793.2  889   .............................S..................................-.................................TM.A..............................................S.  1037
BX440468.2  879   ...............................................................................................W....................R................M-.............S-  1026
AL513664.3  880   ...........T.................W.................................K..................................A............K.T........G........GC...............S.  1030
                                                                                                                                                            \
                                                                                                                                                            |
                                                                                                                                                            G
DC852462.1  901   .......................T.........................................................-.................-...............-......-......-...................  1045

Query       1052  AAAAATGACCCACCAATGGAAGCAGCTGGCTTCACTGCTCAGGTGATTATCCTGAACCATCCAGGCCAAATAAGCGCCGGCTATGCCCCTGTATTGGATTGCCACACGGCTCACATTGCATGCAAGTTTGCTGAGCTG  1189
DC527884.1  1058  ..........................................................................................................................................  1195
BY794942.2  1055  ..........................................................................................................................................  1193
                                                                              \
                                                                              |
                                                                              G
BX426150.2  1025  ..........-.........-.........-...................-..................-.......-.......-................-......T.-............               1140
BU902062.1  1037  ........................C.G......-.........-.........................T....-.....G.......-..A...........C..G..T...-..............           1163
                                                        \                          \                \              \
                                                        |                          |                |              |
                                                        C                          G                T              A
BX402801.2  1042  ....W-..A.................K..Y...............T..W......................W.....S.S-....-.........K.W.......                                  1144
                                                                                                                        \
                                                                                                                        |
                                                                                                                        M
AL513590.3  1021  ..........................Y.........K.C.........W......M.M.-................................T.......T...M.Y..S.Y.A..................       1153
                                                                                   \
                                                                                   |
                                                                                   C
BX402793.2  1038  ....WKR...M-.....................M....Y.........-......M.M-...M...M-................S...                                                   1121
BX440468.2  1027  ...........M.....................M......M..........-....MWC..R.-.....A..M-..S..-.......Y-.........                                          1120
AL513664.3  1031  -.........................G...........Y.........W.M...................WR....S.SY-......-...T......                                           1127
DC852462.1  1046  ....C-.........-..-.-.........-................-...-...-.-......-.-......T-..T-............G-..A..G.......A...............-.C.-...........  1168
                                                                                                                                      \
                                                                                                                                      |
                                                                                                                                      G

  Database: Database of GenBank+EMBL+DDBJ sequences from EST Divisions
    Posted date:  Feb 24, 2019  1:46 AM
  Number of letters in database: 43,200,890,307
  Number of sequences in database:  77,566,966

Lambda      K        H
   1.33    0.621     1.12
Gapped
Lambda      K        H
   1.28    0.460    0.850
Matrix: blastn matrix:1 -2
Gap Penalties: Existence: 0, Extension: 0
Number of Sequences: 77566966
Number of Hits to DB: 3956718
Number of extensions: 27026
Number of successful extensions: 27026
Number of sequences better than 10: 972
Number of HSP's better than 10 without gapping: 0
Number of HSP's gapped: 23040
Number of HSP's successfully gapped: 22826
Length of database: 43200890307
A: 0
X1: 13 (25.0 bits)
X2: 32 (59.1 bits)
X3: 54 (99.7 bits)
S1: 13 (25.1 bits)
```

```
BLASTN 2.9.0+
Reference: Zheng Zhang, Scott Schwartz, Lukas Wagner, and
Webb Miller (2000), "A greedy algorithm for aligning DNA
sequences", J Comput Biol 2000; 7(1-2):203-14.


RID: 8R733CVH014


Database: Database of GenBank+EMBL+DDBJ sequences from EST Divisions
           77,566,966 sequences; 43,200,890,307 total letters
Query= T1-C [organism=Homo sapiens] anxa1

Length=1480


                                                                  Score        E     Max
Sequences producing significant alignments:                       (Bits)     Value  ident

BU508007.1 AGENCOURT_10128424 NIH_MGC_71 Homo sapiens cDNA clo...  1929       0.0    98%
AL570428.3 AL570428 Homo sapiens PLACENTA COT 25-NORMALIZED Ho...  1910       0.0    98%
AL544231.3 AL544231 Homo sapiens PLACENTA COT 25-NORMALIZED Ho...  1905       0.0    98%
DC631365.1 DC631365 macaque bone marrow cDNA library QbmA Maca...  1879       0.0    94%
DC636772.1 DC636772 macaque bone marrow cDNA library QbmA Maca...  1853       0.0    94%
AL541874.3 AL541874 Homo sapiens PLACENTA Homo sapiens cDNA cl...  1853       0.0    99%
BU902298.1 AGENCOURT_10127317 NIH_MGC_71 Homo sapiens cDNA clo...  1847       0.0    96%
BX438944.2 BX438944 Homo sapiens PLACENTA Homo sapiens cDNA cl...  1844       0.0    99%
AL576223.3 AL576223 Homo sapiens PLACENTA COT 25-NORMALIZED Ho...  1831       0.0    98%
AL570884.3 AL570884 Homo sapiens PLACENTA COT 25-NORMALIZED Ho...  1825       0.0    97%

ALIGNMENTS

Query       87    AGTGTGAAATCTTCAGAGAAGAATTTCTCTTTAGTTCTTTGCAAGAAGGTAGAGATAAAGACACTTTTTCAAAAATGGCAATGGTATCAGAATTCCTCAAGCAGGCCTGGTTTATTGAAAATGAAGAGCAGGAATATGTTCAAACTGTGA  236
BU508007.1  1                         ..................................................................................................................................  131
                                                                                 \
                                                                                 |
                                                                                 N
AL544231.3  19                     ..T..................................................................................................................................  151
DC631365.1  1     ......-....................C.C........................................................................................................................  149
DC636772.1  1     ......-....................C.C........................................................................................................................  149
AL541874.3  1                              .............................................................................................................................  125
BU902298.1  24              ............................................................................................................................................  164
                                                                                 \
                                                                                 |
                                                                                 N
BX438944.2  1                      .....................................................................................................................................  133

Query       237   AGTCATCCAAAGGTGGTCCCGGATCAGCGGTGAGCCCCTATCCTACCTTCAATCCATCCTCGGATGTCGCTGCCTTGCATAAGGCCATAATGGTTAAAGGTGTGGATGAAGCAACCATCATTGACATTCTAACTAAGCGAAACAATGCAC  386
BU508007.1  132   ......................................................................................................................................................  281
AL570428.3  1088                                                                                                                                 .....-.T............-..  1068
AL544231.3  152   ......................................................................................................................................................  301
DC631365.1  150   .A.................T........A......................................T...........C......................................................................  299
DC636772.1  150   .A.................T........A..................................................C......................................................................  299
AL541874.3  126   ......................................................................................................................................................  275
BU902298.1  165   ......................................................................................................................................................  314
BX438944.2  134   ......................................................................................................................................................  283

Query       387   AGCGTCAACAGATCAAAGCAGCATATCTCCAGGAAACAGGAAAGCCCCTGGATGAAACACTGAAGAAAGCCCTTACAGGTCACCTTGAGGAGGTTGTTTTAGCTCTGCTAAAAACTCCAGCGCAATTTGATGCTGATGAACTTCGTGCTG  536
BU508007.1  282   ......................................................................................................................................................  431
AL570428.3  1067  ...T....-.A..-..........W.......-.........GC...........................-Y.............................................................................  922
AL544231.3  302   ......................................................................................................................................................  451
DC631365.1  300   ...........................................................................TG....................................................................G....  449
DC636772.1  300   .........................................................................G.TG....................................................................G....  449
AL541874.3  276   ......................................................................................................................................................  425
BU902298.1  315   ......................................................................................................................................................  464
BX438944.2  284   ......................................................................................................................................................  433
AL576223.3  1050       ......-W......-R...-.Y....-........-........-.R...................-.....-.D.....WR............-.A...-.V..................................-.......  917
AL570884.3  1059                 .........T.....T............C...Y...W...............RS....W.....Y.M.....R...T...K......YY..S.W......C.............................K...S  924
                                    \
                                    |
                                    M

Query       537   CCATGAAGGGCCTTGGAACTGATGAAGATACTCTAATTGAGATTTTGGCATCAAGAACTAACAAAGAAATCAGAGACATTAACAGGGTCTACAGAGAGGAACTGAAGAGAGATCTGGCCAAAGACATAACCTCAGACACATCTGGAGATT  686
BU508007.1  432   ......................................................................................................................................................  581
AL570428.3  921   ................M...............................................................W.....................................................................  772
AL544231.3  452   ......................................................................................................................................................  601
DC631365.1  450   ............................C.....G.............................G.....................................................................................  599
DC636772.1  450   ............................C.....G...................................................................................................................  599
AL541874.3  426   ......................................................................................................................................................  575
BU902298.1  465   ......................................................................................................................................................  614
BX438944.2  434   ......................................................................................................................................................  583
AL576223.3  916   .................................W..AAR...............................................................................................................  767
```

```
AL570884.3  923   ......................................................................................................................................................  774

Query       687   TTCGGAACGCTTTGCTTTCTCTTGCTAAGGGTGACCGATCTGAGGACTTTGGTGTGAATGAAGACTTGGCTGATTCAGATGCCAGGGCCTTGTATGAAGCAGGAGAAAGGAGAAAGGGGACAGACGTAAACGTGTTCAATACCATCCTTA  836
BU508007.1  582   ......................................................................................................................................................  731
AL570428.3  771   ..............................................................R.......................................................N...............................  622
AL544231.3  602   ......................................................................................................................................................  751
DC631365.1  600   ....................................................................................................G.......................T.........................  749
DC636772.1  600   ....................................................................................................G.......................T.........................  749
AL541874.3  576   ......................................................................................................................................................  725
BU902298.1  615   ......................................................................................................................................................  764
BX438944.2  584   ......................................................................................................................................................  733
AL576223.3  766   ......................................................................................................................................................  617
AL570884.3  773   ......................................................................................................................................................  624

Query       837   CCACCAGAAGCTATCCACAACTTCGCAGAGTGTTTCAGAAATACACCAAGTACAGTAAGCATGACATGAACAAAGTTCTGGACCTGGAGTTGAAAGGTGACATTGAGAAATGCCTCACAGCTATCGTGAAGTGCGCCACAAGCAAACCAG  986
BU508007.1  732   ......................................................................................................................................................  881
AL570428.3  621   ..............................................................A.......................................................................................  472
AL544231.3  752   .......................................................................................................................................-..............  900
DC631365.1  750   .....C.............T.................................................................................................................T................  899
DC636772.1  750   .....C.............T.................................................................................................................T................  899
AL541874.3  726   ......................................................................................................................................................  875
BU902298.1  765   ..........................................................................................................A....................TG.....................  914
BX438944.2  734   ......................................................................................................................................................  883
AL576223.3  616   ......................................................................................................................................................  467
AL570884.3  623   ......................................................................................................................................................  474

Query       987   CTTTCTTTGCAGAGAAGCTTCATCAAGCCATGAAAGGTGTTGGAACTCGCCATAAGGCATTGATCAGGATTATGGTTTCCCGTTCTGAAATTGACATGAATGATATCAAAGCATTCTATCAGAAGATGTATGGTATCTCCCTTTGCCAAG  1136
BU508007.1  882   ........................-..........................C.....................................................................-......G....G...............C  1032
                                                                                           \                                             \                  \
                                                                                           |                                             |                  |
                                                                                           G                                             T                  C
AL570428.3  471   ............................................................................................A.........................................T...............  322
AL544231.3  901   ...........................................................................................................................G.W-......WWY-.............  1048
DC631365.1  900   ................A........G.............C.......................................A..........................................-.........................-.  1048
                                                                                           \
                                                                                           |
                                                                                           G
DC636772.1  900   ................A........G.............C..........-............................A..................-...............................................-.-.  1048
                                                                        \                  \                                          \
                                                                        |                  |                                          |
                                                                        T                  G                                          T
AL541874.3  876   ..............................................................................................................-....-.......-.....W..K-......-.......-.  1019
BU902298.1  915   .....C..................................................C...........GA.....G...................T.......A...C....T........A....A.G....A.......C...G...C  1067
                                                                                                                              \                  \                   \
                                                                                                                              |                  |                   |
                                                                                                                              G                  A                   C
BX438944.2  884   .......................................K...R.............S...........................................D.......R-....Y.............W..KD-.Y.......Y...  1030
                                                                                                                    \
                                                                                                                    |
                                                                                                                    A
AL576223.3  466   ......................................................................................................................................................  317
AL570884.3  473   ......................................................................................................................................................  324

Query       1137  CCATCCTGGATGAAACCAAAGGAGATTATGAGAAAATCCTGGTGGCTCTTTGTGGAGGAAACTAAACATTCCCTTGATGGTCTCAAGCTATGATCAGAAGACTTTAATTATATATTTTCATCCTATAAGCTTAAATAGGAAAGTTTCTTC  1286
BU508007.1  1033  ..T...G.........C....GA...T..TA..........................................C......C.....                                                                 1123
                                                               \                 \        \     \
                                                               |                 |        |     |
                                                               G                 A        T     AG
AL570428.3  321   ......................................................................................................................................................  172
AL544231.3  1049  .MWY.-........-.......R...-........-..Y..K-.......K-...R....                                                                                            1102
DC631365.1  1049  ....-..-.....-.-.--......-....-...-..-....-.............-..-....-...-....-....A-...-.......................-........CA...G..-.-..C...-.-..C.-...-..-.  1173
                                                                                                                     \
                                                                                                                     |
                                                                                                                     A
DC636772.1  1049  .-...-..-.....GG-.--......-....-......-.....-...........-..-....-...-.........-.....-....GA...........C-.......-....-....-..-.-...G..-...-.....C-..-.  1174
                                                      \   \
                                                      |   |
                                                      C   G
AL541874.3  1020  .M-..-......                                                                                                                                            1029
BU902298.1  1068  ..C.....T........G...A.......A.......C..G............A....AA.............                                                                               1152
                             \                 \               \\        \         \  \  \
                             |                 |               ||        |         |  |  |
                             G                 G               G|        G         T  A  T
                                                                |
                                                                C
AL576223.3  316   ......................................................................................................................................................  167
AL570884.3  323   ......................................................................................................................................................  174

Query       1287  AACAGGATTACAGTGTAGCTACCTACATGCTGAAAAATATAGCCTTTAAATCATTTTTATATTATAACTCTGTATAATAGAGATAAGTCCATTTTTTAAAAATGTTTTCCCCAAACCATAAAACCCTATACAAGTTGTTCTAGTAACAAT  1436
AL570428.3  171   ...........................................................................T..........................................................................  22
DC631365.1  1174  --......-..C..--.........T-......C...-.....-.A....                                                                                                      1216
                                              \
```

```
                                    |
                                    G
DC636772.1  1175  --.........G...-..A.-...T-........T..GG.--...A....A..........                                                                                      1231
                                    \                          \
                                    |                          |
                                    G                          A
AL576223.3  166   .............................................................................................................B........................................  17
AL570884.3  173   .......................................................................................................RW....V.............................N....N....W  23
                                                                                                                                                \
                                                                                                                                                 |
                                                                                                                                                 N

Query       1437  ACATGAGAAAGATGTCTATGT  1457
AL570428.3  21    .....................  1
AL576223.3  16    ................       1
AL570884.3  22    ...............A.....  1
                    \
                    |
                    N
```

Query= T2-2 [organism=Homo sapiens] anxa1

Length=1065

```
                                                                 Score        E     Max
Sequences producing significant alignments:                      (Bits)     Value  ident

BQ228162.1 AGENCOURT_7258587 NIH_MGC_71 Homo sapiens cDNA clon...  1707       0.0    99%
AL574991.3 AL574991 Homo sapiens PLACENTA COT 25-NORMALIZED Ho...  1676       0.0    100%
AL576223.3 AL576223 Homo sapiens PLACENTA COT 25-NORMALIZED Ho...  1661       0.0    99%
AL570884.3 AL570884 Homo sapiens PLACENTA COT 25-NORMALIZED Ho...  1655       0.0    99%
AL570428.3 AL570428 Homo sapiens PLACENTA COT 25-NORMALIZED Ho...  1655       0.0    99%
AL576603.2 AL576603 Homo sapiens PLACENTA COT 25-NORMALIZED Ho...  1652       0.0    99%
AL575425.3 AL575425 Homo sapiens PLACENTA COT 25-NORMALIZED Ho...  1644       0.0    99%
AL576340.3 AL576340 Homo sapiens PLACENTA COT 25-NORMALIZED Ho...  1642       0.0    99%
BM555842.1 AGENCOURT_6544353 NIH_MGC_88 Homo sapiens cDNA clon...  1639       0.0    99%
AL553095.3 AL553095 Homo sapiens PLACENTA COT 25-NORMALIZED Ho...  1615       0.0    98%
```

ALIGNMENTS

```
Query       123   AAAGGGCCTTGGAACTGATGAAGATACTCTAATTGAGATTTTGGCATCAAGAACTAACAAAGAAATCAGAGACATTAACAGGGTCTACAGAGAGGAACTGAAGAGAGATCTGGCCAAAGACATAACCTCAGACACATCTGGAGATTTTCG  272
BQ228162.1  25     .....................................................................................................................................................  173
AL574991.3  911    .....................................................................................................................................................  763
AL576223.3  911    ............................W..AAR...................................................................................................................  763
AL570884.3  918    .....................................................................................................................................................  770
AL570428.3  916    ...........M...............................................................W.........................................................................  768
AL576603.2  912       ........-...........-WM...............-.........................................................................................................  770
AL575425.3  930   ......................................................................R........................R......................................................  781
AL576340.3  915       ....-.............-RW..................-.......-..................................................................................................  774
BM555842.1  1                       ....................................................................................................................................  132
AL553095.3  917    .....................................................................................................................................................  769

Query       273   GAACGCTTTGCTTTCTCTTGCTAAGGGTGACCGATCTGAGGACTTTGGTGTGAATGAAGACTTGGCTGATTCAGATGCCAGGGCCTTGTATGAAGCAGGAGAAAGGAGAAAGGGGACAGACGTAAACGTGTTCAATACCATCCTTACCAC  422
BQ228162.1  174   ......................................................................................................................................................  323
AL574991.3  762   ......................................................................................................................................................  613
AL576223.3  762   ......................................................................................................................................................  613
AL570884.3  769   ......................................................................................................................................................  620
AL570428.3  767   ..........................................................R.......................................................N...................................  618
AL576603.2  769   ......................................................................................................................................................  620
AL575425.3  780   ........................................R.................B........S..............................................T...................................  631
AL576340.3  773   ......................................................................................................................................................  624
BM555842.1  133   ......................................................................................................................................................  282
AL553095.3  768   ......................................................................................................................................................  619

Query       423   CAGAAGCTATCCACAACTTCGCAGAGTGTTTCAGAAATACACCAAGTACAGTAAGCATGACATGAACAAAGTTCTGGACCTGGAGTTGAAAGGTGACATTGAGAAATGCCTCACAGCTATCGTGAAGTGCGCCACAAGCAAACCAGCTTT  572
BQ228162.1  324   ......................................................................................................................................................  473
AL574991.3  612   ......................................................................................................................................................  463
AL576223.3  612   ......................................................................................................................................................  463
AL570884.3  619   ......................................................................................................................................................  470
AL570428.3  617   ..........................................................A...........................................................................................  468
AL576603.2  619   ......................................................................................................................................................  470
AL575425.3  630   ..........................................................N....................................................N......................................  481
AL576340.3  623   ......................................................................................................................................................  474
BM555842.1  283   ......................................................................................................................................................  432
AL553095.3  618   ....................................................................................................................................................YY  469

Query       573   CTTTGCAGAGAAGCTTCATCAAGCCATGAAAGGTGTTGGAACTCGCCATAAGGCATTGATCAGGATTATGGTTTCCCGTTCTGAAATTGACATGAATGATATCAAAGCATTCTATCAGAAGATGTATGGTATCTCCCTTTGCCAAGCCAT  722
BQ228162.1  474   ......................................................................................................................................................  623
AL574991.3  462   ....................................................................................................................................................W.  313
AL576223.3  462   ......................................................................................................................................................  313
AL570884.3  469   ......................................................................................................................................................  320
```

```
AL570428.3  467   ........................................................................................A.........................................T...................  318
AL576603.2  469   ......................................................................................................................................................  320
AL575425.3  480   ......................................................................................................................................................  331
AL576340.3  473   ......................................................................................................................................................  324
BM555842.1  433   ......................................................................................................................................................  582
AL553095.3  468   ...V...........C.......................V......................................S..S..................RK...R...G...R....................................  319

Query       723   CCTGGATGAAACCAAAGGAGATTATGAGAAAATCCTGGTGGCTCTTTGTGGAGGAAACTAAACATTCCCTTGATGGTCTCAAGCTATGATCAGAAGACTTTAATTATATATTTTCATCCTATAAGCTTAAATAGGAAAGTTTCTTCAACA  872
BQ228162.1  624   ......................................................................................................................................................  774
                                                                                                                                                         \
                                                                                                                                                         |
                                                                                                                                                         A
AL574991.3  312   .............................................................................Y........................................................................  163
AL576223.3  312   ......................................................................................................................................................  163
AL570884.3  319   ......................................................................................................................................................  170
AL570428.3  317   ......................................................................................................................................................  168
AL576603.2  319   ......................................................................................................................................................  170
AL575425.3  330   ......................................................................................................................................................  181
AL576340.3  323   ..........R.......R.......................................................................................................................................  174
BM555842.1  583   ......................................................................................................................................................  732
AL553095.3  318   ......K...G.......R.......................................................................................................................................  169

Query       873   GGATTACAGTGTAGCTACCTACATGCTGAAAAATATAGCCTTTAAATCATTTTTATATTATAACTCTGTATAATAGAGATAAGTCCATTTTTTAAAAATGTTTTCCCCAAACCATAAAACCCTATACAAGTTGTTCTAGTAACAATACAT  1022
BQ228162.1  775   ...........................................................................................................................................G..........  925
                                                                                                                                                   \
                                                                                                                                                   |
                                                                                                                                                   G
AL574991.3  162   ......................................................................................................................................................  13
AL576223.3  162   .........................................................................................................B............................................  13
AL570884.3  169   ...................................................................................................RW....V.............................N....N....W....  18
                                                                                                                                                           \          \
                                                                                                                                                           |          |
                                                                                                                                                           N          N
AL570428.3  167   .......................................................................T..............................................................................  18
AL576603.2  169   .................................................................................................................................................A....  19
                                                                                                                                                  \
                                                                                                                                                  |
                                                                                                                                                  C
AL575425.3  180   ...................N.............................................................................................................................N....  31
AL576340.3  173   ......................................................................................................................................................  23
                                                                                                                                                   \
                                                                                                                                                   |
                                                                                                                                                   N
BM555842.1  733   .....................................................................................................................................G................  888
                                                                                                                    \     \              \              \        \ \
                                                                                                                    |     |              |              |        | |
                                                                                                                    A     C              C              N        C N
AL553095.3  168   ......................................................................................................................................................  18
                                                                                                                                                         \
                                                                                                                                                         |
                                                                                                                                                         N

Query       1023  GAGAAAGATGTCTATGTAGCTGAAAATAAAATGACGTC  1060
BQ228162.1  926   ......................................  965
                    \                   \
                    |                   |
                    G                   A
AL574991.3  12    ............                            1
AL576223.3  12    ............                            1
AL570884.3  17    ...........A.....                       1
AL570428.3  17    .................                       1
AL576603.2  18    ..................                      1
AL575425.3  30    ........G.....                          17
AL576340.3  22    ..................                      5
BM555842.1  889   ...............................         922
                        \     \  \
                        |     |  |
                        A     C  G
AL553095.3  17    ..............G..                       1

Query= T3-20 [organism=Homo sapiens] anxa1

Length=1346


                                                                      Score        E     Max
Sequences producing significant alignments:                          (Bits)     Value  ident

BQ228162.1 AGENCOURT_7258587 NIH_MGC_71 Homo sapiens cDNA clon...  1607       0.0    99%
AL576603.2 AL576603 Homo sapiens PLACENTA COT 25-NORMALIZED Ho...  1583       0.0    100%
AL576340.3 AL576340 Homo sapiens PLACENTA COT 25-NORMALIZED Ho...  1581       0.0    100%
AL576223.3 AL576223 Homo sapiens PLACENTA COT 25-NORMALIZED Ho...  1580       0.0    100%
```

```
AL574991.3 AL574991 Homo sapiens PLACENTA COT 25-NORMALIZED Ho...  1576       0.0    100%
BM555842.1 AGENCOURT_6544353 NIH_MGC_88 Homo sapiens cDNA clon...  1570       0.0    99%
AL570428.3 AL570428 Homo sapiens PLACENTA COT 25-NORMALIZED Ho...  1559       0.0    99%
AL570884.3 AL570884 Homo sapiens PLACENTA COT 25-NORMALIZED Ho...  1555       0.0    99%
AL575425.3 AL575425 Homo sapiens PLACENTA COT 25-NORMALIZED Ho...  1543       0.0    99%
BE614257.1 601504294T1 NIH_MGC_71 Homo sapiens cDNA clone IMAG...  1528       0.0    99%

ALIGNMENTS

Query       324   TCTAACTAAGCGAAACAATGCACAGCGTCAACAGATCAAAGCAGCATATCTCCAGGAAACAGGAAAGCCCCTGGATGAAACACTGAAGAAAGCCCTTACAGGTCACCTTGAGGAGGTTGTTTTAGCTCTGCTAAAAACAAAGAAATCAGA  473
BQ228162.1  79                                                                                                                                           ...............  93
AL576603.2  864                                                                                                                                          ...............  850
AL576340.3  868                                                                                                                                          ...............  854
AL576223.3  857                                                                                                                                          ...............  843
AL574991.3  857                                                                                                                                          ...............  843
BM555842.1  38                                                                                                                                           ...............  52
AL570428.3  862                                                                                                                                          ...............  848
AL570428.3  1088  .....-.T............-.....T....-.A..-..........W.......-.........GC...........................Y.-.........................................              957
AL570884.3  864                                                                                                                                          ...............  850
AL575425.3  875                                                                                                                                          ...............  861
AL575425.3  1037                                                                      ........-..........-........M.......M...................KG.........C..              970
BE614257.1  871                                                                                                                                               ..........  862

Query       474   GACATTAACAGGGTCTACAGAGAGGAACTGAAGAGAGATCTGGCCAAAGACATAACCTCAGACACATCTGGAGATTTTCGGAACGCTTTGCTTTCTCTTGCTAAGGGTGACCGATCTGAGGACTTTGGTGTGAATGAAGACTTGGCTGAT  623
BQ228162.1  94    ......................................................................................................................................................  243
AL576603.2  849   ......................................................................................................................................................  700
AL576340.3  853   ......................................................................................................................................................  704
AL576223.3  842   ......................................................................................................................................................  693
AL574991.3  842   ......................................................................................................................................................  693
BM555842.1  53    ......................................................................................................................................................  202
AL570428.3  847   ......W...................................................................................................................................R...........  698
AL570884.3  849   ......................................................................................................................................................  700
AL575425.3  860   R........................R..............................................................................................R.................B........S..  711
BE614257.1  861   ............-..-........A.T.........A.................C...........................................-...................................................  713
                                                \                                        \
                                                |                                        |
                                                A                                        G

Query       624   TCAGATGCCAGGGCCTTGTATGAAGCAGGAGAAAGGAGAAAGGGGACAGACGTAAACGTGTTCAATACCATCCTTACCACCAGAAGCTATCCACAACTTCGCAGAGTGTTTCAGAAATACACCAAGTACAGTAAGCATGACATGAACAAA  773
BQ228162.1  244   ......................................................................................................................................................  393
AL576603.2  699   ......................................................................................................................................................  550
AL576340.3  703   ......................................................................................................................................................  554
AL576223.3  692   ......................................................................................................................................................  543
AL574991.3  692   ......................................................................................................................................................  543
BM555842.1  203   ......................................................................................................................................................  352
AL570428.3  697   ............................................N.............................................................................................A...........  548
AL570884.3  699   ......................................................................................................................................................  550
AL575425.3  710   ............................................T.............................................................................................N...........  561
BE614257.1  712   ......................................................................................................................................................  563

Query       774   GTTCTGGACCTGGAGTTGAAAGGTGACATTGAGAAATGCCTCACAGCTATCGTGAAGTGCGCCACAAGCAAACCAGCTTTCTTTGCAGAGAAGCTTCATCAAGCCATGAAAGGTGTTGGAACTCGCCATAAGGCATTGATCAGGATTATG  923
BQ228162.1  394   ......................................................................................................................................................  543
AL576603.2  549   ......................................................................................................................................................  400
AL576340.3  553   ......................................................................................................................................................  404
AL576223.3  542   ......................................................................................................................................................  393
AL574991.3  542   ......................................................................................................................................................  393
BM555842.1  353   ......................................................................................................................................................  502
AL570428.3  547   ......................................................................................................................................................  398
AL570884.3  549   ......................................................................................................................................................  400
AL575425.3  560   .........................................N............................................................................................................  411
BE614257.1  562   ......................................................................................................................................................  413

Query       924   GTTTCCCGTTCTGAAATTGACATGAATGATATCAAAGCATTCTATCAGAAGATGTATGGTATCTCCCTTTGCCAAGCCATCCTGGATGAAACCAAAGGAGATTATGAGAAAATCCTGGTGGCTCTTTGTGGAGGAAACTAAACATTCCCT  1073
BQ228162.1  544   ......................................................................................................................................................  693
AL576603.2  399   ......................................................................................................................................................  250
AL576340.3  403   ..........................................................................................R.......R...................................................  254
AL576223.3  392   ......................................................................................................................................................  243
AL574991.3  392   ..............................................................................W.......................................................................  243
BM555842.1  503   ......................................................................................................................................................  652
AL570428.3  397   ..................A.........................................T.........................................................................................  248
AL570884.3  399   ......................................................................................................................................................  250
AL575425.3  410   ......................................................................................................................................................  261
BE614257.1  412   ......................................................................................................................................................  263

Query       1074  TGATGGTCTCAAGCTATGATCAGAAGACTTTAATTATATATTTTCATCCTATAAGCTTAAATAGGAAAGTTTCTTCAACAGGATTACAGTGTAGCTACCTACATGCTGAAAAATATAGCCTTTAAATCATTTTTATATTATAACTCTGTA  1223
BQ228162.1  694   ......................................................................................................................................................  844
                                                                                   \
                                                                                   |
                                                                                   A
AL576603.2  249   ......................................................................................................................................................  100
AL576340.3  253   ......................................................................................................................................................  104
AL576223.3  242   ......................................................................................................................................................  93
```

```
AL574991.3  242   ......Y...............................................................................................................................................  93
BM555842.1  653   ......................................................................................................................................................  802
AL570428.3  247   ......................................................................................................................................................  98
AL570884.3  249   ......................................................................................................................................................  100
AL575425.3  260   ...................................................................................................N..................................................  111
BE614257.1  262   ......................................................................................................................................................  113

Query       1224  TAATAGAGATAAGTCCATTTTTTAAAAATGTTTTCCCCAAACCATAAAACCCTATACAAGTTGTTCTAGTAACAATACATGAGAAAGATGTCTATGTAGCTGAAAATAAAATGACGTC  1341
BQ228162.1  845   .....................................................................G................................................  965
                                                                             \                      \                   \
                                                                             |                      |                   |
                                                                             G                      G                   A
AL576603.2  99    ...........................................................................A......................                      1
                                                                                            \
                                                                                            |
                                                                                            C
AL576340.3  103   ..................................................................................................                      5
                                                                                             \
                                                                                             |
                                                                                             N
AL576223.3  92    ...................................B........................................................                            1
AL574991.3  92    ............................................................................................                            1
BM555842.1  803   ...............................................................G...............................................         922
                                              \     \              \              \        \ \          \     \  \
                                              |     |              |              |        | |          |     |  |
                                              A     C              C              N        C N          A     C  G
AL570428.3  97    .T...............................................................................................                       1
AL570884.3  99    .............................RW....V.............................N....N....W...............A.....                       1
                                                                                     \          \
                                                                                     |          |
                                                                                     N          N
AL575425.3  110   ...........................................................................N............G.....                          17
BE614257.1  112   ......................................................................................T.A-.................            6
                                                                                                                          \
                                                                                                                          |
                                                                                                                          T
```

Query= T4-18 [organism=Homo sapiens] anxa1

Length=1226

```
                                                                  Score        E     Max
Sequences producing significant alignments:                      (Bits)     Value  ident

CB306442.1 UI-CF-EN1-aej-e-15-0-UI.s1 UI-CF-EN1 Homo sapiens c...  1295       0.0    100%
CA447733.1 UI-H-EI0-ayf-a-16-0-UI.s1 NCI_CGAP_EI0 Homo sapiens...  1295       0.0    100%
CA447223.1 UI-H-EI0-ayd-b-19-0-UI.s1 NCI_CGAP_EI0 Homo sapiens...  1295       0.0    100%
CA419844.1 UI-H-FH0-bcm-d-16-0-UI.s1 NCI_CGAP_FH0 Homo sapiens...  1295       0.0    100%
CA417462.1 UI-H-FE0-bbw-c-13-0-UI.s1 NCI_CGAP_FE0 Homo sapiens...  1295       0.0    100%
CA415613.1 UI-H-EZ0-bay-p-10-0-UI.s1 NCI_CGAP_Ch1 Homo sapiens...  1295       0.0    100%
CA414702.1 UI-H-EZ0-bar-e-18-0-UI.s1 NCI_CGAP_Ch1 Homo sapiens...  1295       0.0    100%
BU633206.1 UI-H-FL1-bgt-f-12-0-UI.s1 NCI_CGAP_FL1 Homo sapiens...  1295       0.0    100%
BQ776306.1 UI-H-FH0-bcn-i-12-0-UI.s1 NCI_CGAP_FH0 Homo sapiens...  1295       0.0    100%
BQ775850.1 UI-H-FH0-bcg-g-15-0-UI.s1 NCI_CGAP_FH0 Homo sapiens...  1295       0.0    100%
```

ALIGNMENTS

```
Query       526   AAGCAGGAGAAAGGAGAAAGGGGACAGACGTAAACGTGTTCAATACCATCCTTACCACCAGAAGCTATCCACAACTTCGCAGAGTGTTTCAGAAATACACCAAGTACAGTAAGCATGACATGAACAAAGTTCTGGACCTGGAGTTGAAAG  675
CB306442.1  719   ......................................................................................................................................................  570
CA447733.1  719   ......................................................................................................................................................  570
CA447223.1  719   ......................................................................................................................................................  570
CA419844.1  719   ......................................................................................................................................................  570
CA417462.1  716   ......................................................................................................................................................  567
CA415613.1  721   ......................................................................................................................................................  572
CA414702.1  721   ......................................................................................................................................................  572
BU633206.1  719   ......................................................................................................................................................  570
BQ776306.1  719   ......................................................................................................................................................  570
BQ775850.1  716   ......................................................................................................................................................  567

Query       676   GTGACATTGAGAAATGCCTCACAGCTATCGTGAAGTGCGCCACAAGCAAACCAGCTTTCTTTGCAGAGAAGCTTCATCAAGCCATGAAAGGTGTTGGAACTCGCCATAAGGCATTGATCAGGATTATGGTTTCCCGTTCTGAAATTGACA  825
CB306442.1  569   ......................................................................................................................................................  420
CA447733.1  569   ......................................................................................................................................................  420
CA447223.1  569   ......................................................................................................................................................  420
CA419844.1  569   ......................................................................................................................................................  420
CA417462.1  566   ......................................................................................................................................................  417
CA415613.1  571   ......................................................................................................................................................  422
CA414702.1  571   ......................................................................................................................................................  422
BU633206.1  569   ......................................................................................................................................................  420
BQ776306.1  569   ......................................................................................................................................................  420
BQ775850.1  566   ......................................................................................................................................................  417
```

```
Query       826   TGAATGATATCAAAGCATTCTATCAGAAGATGTATGGTATCTCCCTTTGCCAAGCCATCCTGGATGAAACCAAAGGAGATTATGAGAAAATCCTGGTGGCTCTTTGTGGAGGAAACTAAACATTCCCTTGATGGTCTCAAGCTATGATCA  975
CB306442.1  419   ......................................................................................................................................................  270
CA447733.1  419   ......................................................................................................................................................  270
CA447223.1  419   ......................................................................................................................................................  270
CA419844.1  419   ......................................................................................................................................................  270
CA417462.1  416   ......................................................................................................................................................  267
CA415613.1  421   ......................................................................................................................................................  272
CA414702.1  421   ......................................................................................................................................................  272
BU633206.1  419   ......................................................................................................................................................  270
BQ776306.1  419   ......................................................................................................................................................  270
BQ775850.1  416   ......................................................................................................................................................  267

Query       976   GAAGACTTTAATTATATATTTTCATCCTATAAGCTTAAATAGGAAAGTTTCTTCAACAGGATTACAGTGTAGCTACCTACATGCTGAAAAATATAGCCTTTAAATCATTTTTATATTATAACTCTGTATAATAGAGATAAGTCCATTTTT  1125
CB306442.1  269   ......................................................................................................................................................  120
CA447733.1  269   ......................................................................................................................................................  120
CA447223.1  269   ......................................................................................................................................................  120
CA419844.1  269   ......................................................................................................................................................  120
CA417462.1  266   ......................................................................................................................................................  117
CA415613.1  271   ......................................................................................................................................................  122
CA414702.1  271   ......................................................................................................................................................  122
BU633206.1  269   ......................................................................................................................................................  120
BQ776306.1  269   ......................................................................................................................................................  120
BQ775850.1  266   ......................................................................................................................................................  117

Query       1126  TAAAAATGTTTTCCCCAAACCATAAAACCCTATACAAGTTGTTCTAGTAACAATACATGAGAAAGATGTCTATGTAGCTGAAAATAAAATGACGTCACAAG  1226
CB306442.1  119   .....................................................................................................  19
CA447733.1  119   .....................................................................................................  19
CA447223.1  119   .....................................................................................................  19
CA419844.1  119   .....................................................................................................  19
CA417462.1  116   .....................................................................................................  16
CA415613.1  121   .....................................................................................................  21
CA414702.1  121   .....................................................................................................  21
BU633206.1  119   .....................................................................................................  19
BQ776306.1  119   .....................................................................................................  19
BQ775850.1  116   .....................................................................................................  16

Query= T5-22 [organism=Homo sapiens] anxa1

Length=1238


                                                                   Score        E     Max
Sequences producing significant alignments:                       (Bits)     Value  ident

CA446942.1 UI-H-ED0-aya-g-19-0-UI.s1 NCI_CGAP_ED0 Homo sapiens...  1260       0.0    100%
CA442733.1 UI-H-DP0-avr-a-19-0-UI.s1 NCI_CGAP_Fs1 Homo sapiens...  1260       0.0    100%
CA419750.1 UI-H-FH0-bcm-o-09-0-UI.s1 NCI_CGAP_FH0 Homo sapiens...  1260       0.0    100%
CA418232.1 UI-H-FH0-bcf-o-06-0-UI.s1 NCI_CGAP_FH0 Homo sapiens...  1260       0.0    100%
CA418210.1 UI-H-FH0-bcf-i-22-0-UI.s1 NCI_CGAP_FH0 Homo sapiens...  1260       0.0    100%
BU615320.1 UI-H-FH0-bcj-e-10-0-UI.s1 NCI_CGAP_FH0 Homo sapiens...  1260       0.0    100%
BQ774950.1 UI-H-FH0-bcd-n-16-0-UI.s1 NCI_CGAP_FH0 Homo sapiens...  1260       0.0    100%
BQ774641.1 UI-H-FH0-bcc-l-13-0-UI.s1 NCI_CGAP_FH0 Homo sapiens...  1260       0.0    100%
BQ774017.1 UI-H-FH0-bce-a-18-0-UI.s1 NCI_CGAP_FH0 Homo sapiens...  1260       0.0    100%
BQ773942.1 UI-H-FH0-bce-c-05-0-UI.s1 NCI_CGAP_FH0 Homo sapiens...  1260       0.0    100%

ALIGNMENTS

Query       557   GAGAAAGGGACAGACGTAAACGTGTTCAATACCATCCTTACCACCAGAAGCTATCCACAACTTCGCAGAGTGTTTCAGAAATACACCAAGTACAGTAAGCATGACATGAACAAAGTTCTGGACCTGGAGTTGAAAGGTGACATTGAGAAA  706
CA446942.1  705   ......................................................................................................................................................  556
CA442733.1  705   ......................................................................................................................................................  556
CA419750.1  705   ......................................................................................................................................................  556
CA418232.1  705   ......................................................................................................................................................  556
CA418210.1  702   ......................................................................................................................................................  553
BU615320.1  705   ......................................................................................................................................................  556
BQ774950.1  705   ......................................................................................................................................................  556
BQ774641.1  705   ......................................................................................................................................................  556
BQ774017.1  702   ......................................................................................................................................................  553
BQ773942.1  705   ......................................................................................................................................................  556

Query       707   TGCCTCACAGCTATCGTGAAGTGCGCCACAAGCAAACCAGCTTTCTTTGCAGAGAAGCTTCATCAAGCCATGAAAGGTGTTGGAACTCGCCATAAGGCATTGATCAGGATTATGGTTTCCCGTTCTGAAATTGACATGAATGATATCAAA  856
CA446942.1  555   ......................................................................................................................................................  406
CA442733.1  555   ......................................................................................................................................................  406
CA419750.1  555   ......................................................................................................................................................  406
CA418232.1  555   ......................................................................................................................................................  406
CA418210.1  552   ......................................................................................................................................................  403
BU615320.1  555   ......................................................................................................................................................  406
BQ774950.1  555   ......................................................................................................................................................  406
BQ774641.1  555   ......................................................................................................................................................  406
BQ774017.1  552   ......................................................................................................................................................  403
BQ773942.1  555   ......................................................................................................................................................  406

Query       857   GCATTCTATCAGAAGATGTATGGTATCTCCCTTTGCCAAGCCATCCTGGATGAAACCAAAGGAGATTATGAGAAAATCCTGGTGGCTCTTTGTGGAGGAAACTAAACATTCCCTTGATGGTCTCAAGCTATGATCAGAAGACTTTAATTA  1006
```

```
CA446942.1  405   ......................................................................................................................................................  256
CA442733.1  405   ......................................................................................................................................................  256
CA419750.1  405   ......................................................................................................................................................  256
CA418232.1  405   ......................................................................................................................................................  256
CA418210.1  402   ......................................................................................................................................................  253
BU615320.1  405   ......................................................................................................................................................  256
BQ774950.1  405   ......................................................................................................................................................  256
BQ774641.1  405   ......................................................................................................................................................  256
BQ774017.1  402   ......................................................................................................................................................  253
BQ773942.1  405   ......................................................................................................................................................  256

Query       1007  TATATTTTCATCCTATAAGCTTAAATAGGAAAGTTTCTTCAACAGGATTACAGTGTAGCTACCTACATGCTGAAAAATATAGCCTTTAAATCATTTTTATATTATAACTCTGTATAATAGAGATAAGTCCATTTTTTAAAAATGTTTTCC  1156
CA446942.1  255   ......................................................................................................................................................  106
CA442733.1  255   ......................................................................................................................................................  106
CA419750.1  255   ......................................................................................................................................................  106
CA418232.1  255   ......................................................................................................................................................  106
CA418210.1  252   ......................................................................................................................................................  103
BU615320.1  255   ......................................................................................................................................................  106
BQ774950.1  255   ......................................................................................................................................................  106
BQ774641.1  255   ......................................................................................................................................................  106
BQ774017.1  252   ......................................................................................................................................................  103
BQ773942.1  255   ......................................................................................................................................................  106

Query       1157  CCAAACCATAAAACCCTATACAAGTTGTTCTAGTAACAATACATGAGAAAGATGTCTATGTAGCTGAAAATAAAATGACGTC  1238
CA446942.1  105   ..................................................................................  24
CA442733.1  105   ..................................................................................  24
CA419750.1  105   ..................................................................................  24
CA418232.1  105   ..................................................................................  24
CA418210.1  102   ..................................................................................  21
BU615320.1  105   ..................................................................................  24
BQ774950.1  105   ..................................................................................  24
BQ774641.1  105   ..................................................................................  24
BQ774017.1  102   ..................................................................................  21
BQ773942.1  105   ..................................................................................  24

Query= T6-14 [organism=Homo sapiens] anxa1

Length=2854


                                                                   Score        E     Max
Sequences producing significant alignments:                       (Bits)     Value  ident

AL544959.3 AL544959 Homo sapiens PLACENTA COT 25-NORMALIZED Ho...  1701       0.0    100%
BX438944.2 BX438944 Homo sapiens PLACENTA Homo sapiens cDNA cl...  1698       0.0    100%
AL551427.3 AL551427 Homo sapiens PLACENTA COT 25-NORMALIZED Ho...  1696       0.0    100%
AL540651.3 AL540651 Homo sapiens PLACENTA Homo sapiens cDNA cl...  1692       0.0    100%
AL551980.3 AL551980 Homo sapiens PLACENTA COT 25-NORMALIZED Ho...  1687       0.0    100%
AL544231.3 AL544231 Homo sapiens PLACENTA COT 25-NORMALIZED Ho...  1685       0.0    100%
AL541874.3 AL541874 Homo sapiens PLACENTA Homo sapiens cDNA cl...  1683       0.0    100%
BU902298.1 AGENCOURT_10127317 NIH_MGC_71 Homo sapiens cDNA clo...  1683       0.0    99%
BU508007.1 AGENCOURT_10128424 NIH_MGC_71 Homo sapiens cDNA clo...  1681       0.0    100%
AL553114.3 AL553114 Homo sapiens PLACENTA COT 25-NORMALIZED Ho...  1677       0.0    99%

ALIGNMENTS

Query       1     AGTGTGAAATCTTCAGAGAAGAATTTCTCTTTAGTTCTTTGCAAGAAGGTAGAGATAAAGACACTTTTTCAAAAATGGCAATGGTATCAGAATTCCTCAAGCAGGCCTGGTTTATTGAAAATGAAGAGCAGGAATATGTTCAAACTGTGA  150
AL544959.3  1             ..............................................................................................................................................  142
BX438944.2  1                      .....................................................................................................................................  133
AL551427.3  1                    ..T....................................................................................................................................  135
AL540651.3  19                     ..T..................................................................................................................................  151
AL551980.3  22                       ...................................................................................................................................  152
AL544231.3  19                     ..T..................................................................................................................................  151
AL541874.3  1                              .............................................................................................................................  125
BU902298.1  24             .............................................................................................................................................  164
                                                                                 \
                                                                                 |
                                                                                 N
BU508007.1  1                       ...................................................................................................................................  131
                                                                                 \
                                                                                 |
                                                                                 N
AL553114.3  107   ......................................................................................................................................................  256

Query       151   AGTCATCCAAAGGTGGTCCCGGATCAGCGGTGAGCCCCTATCCTACCTTCAATCCATCCTCGGATGTCGCTGCCTTGCATAAGGCCATAATGGTTAAAGGTGTGGATGAAGCAACCATCATTGACATTCTAACTAAGCGAAACAATGCAC  300
AL544959.3  143   ......................................................................................................................................................  292
BX438944.2  134   ......................................................................................................................................................  283
AL551427.3  136   ......................................................................................................................................................  285
AL540651.3  152   ......................................................................................................................................................  301
AL551980.3  153   ......................................................................................................................................................  302
AL544231.3  152   ......................................................................................................................................................  301
AL541874.3  126   ......................................................................................................................................................  275
```

```
BU902298.1  165   ......................................................................................................................................................  314
BU508007.1  132   ......................................................................................................................................................  281
AL553114.3  257   ......................................................................................................................................................  406

Query       301   AGCGTCAACAGATCAAAGCAGCATATCTCCAGGAAACAGGAAAGCCCCTGGATGAAACACTGAAGAAAGCCCTTACAGGTCACCTTGAGGAGGTTGTTTTAGCTCTGCTAAAAACTCCAGCGCAATTTGATGCTGATGAACTTCGTGCTG  450
AL544959.3  293   ......................................................................................................................................................  442
BX438944.2  284   ......................................................................................................................................................  433
AL551427.3  286   ......................................................................................................................................................  435
AL540651.3  302   ......................................................................................................................................................  451
AL551980.3  303   ......................................................................................................................................................  452
AL544231.3  302   ......................................................................................................................................................  451
AL541874.3  276   ......................................................................................................................................................  425
BU902298.1  315   ......................................................................................................................................................  464
BU508007.1  282   ......................................................................................................................................................  431
AL553114.3  407   ......................................................................................................................................................  556

Query       451   CCATGAAGGGCCTTGGAACTGATGAAGATACTCTAATTGAGATTTTGGCATCAAGAACTAACAAAGAAATCAGAGACATTAACAGGGTCTACAGAGAGGAACTGAAGAGAGATCTGGCCAAAGACATAACCTCAGACACATCTGGAGATT  600
AL544959.3  443   ......................................................................................................................................................  592
BX438944.2  434   ......................................................................................................................................................  583
AL551427.3  436   ......................................................................................................................................................  585
AL540651.3  452   ......................................................................................................................................................  601
AL551980.3  453   ......................................................................................................................................................  602
AL544231.3  452   ......................................................................................................................................................  601
AL541874.3  426   ......................................................................................................................................................  575
BU902298.1  465   ......................................................................................................................................................  614
BU508007.1  432   ......................................................................................................................................................  581
AL553114.3  557   ......................................................................................................................................................  706

Query       601   TTCGGAACGCTTTGCTTTCTCTTGCTAAGGGTGACCGATCTGAGGACTTTGGTGTGAATGAAGACTTGGCTGATTCAGATGCCAGGGCCTTGTATGAAGCAGGAGAAAGGAGAAAGGGGACAGACGTAAACGTGTTCAATACCATCCTTA  750
AL544959.3  593   ......................................................................................................................................................  742
BX438944.2  584   ......................................................................................................................................................  733
AL551427.3  586   ......................................................................................................................................................  735
AL540651.3  602   ......................................................................................................................................................  751
AL551980.3  603   ......................................................................................................................................................  752
AL544231.3  602   ......................................................................................................................................................  751
AL541874.3  576   ......................................................................................................................................................  725
BU902298.1  615   ......................................................................................................................................................  764
BU508007.1  582   ......................................................................................................................................................  731
AL553114.3  707   ......................................................................................................................................................  856

Query       751   CCACCAGAAGCTATCCACAACTTCGCAGAGTGTTTCAGAAATACACCAAGTACAGTAAGCATGACATGAACAAAGTTCTGGACCTGGAGTTGAAAGGTGACATTGAGAAATGCCTCACAGCTATCGTGAAGTGCGCCACAAGCAAACCAG  900
AL544959.3  743   ..........................................................................................................................................M.......M...  893
                                                                                                           \
                                                                                                           |
                                                                                                           T
BX438944.2  734   ......................................................................................................................................................  883
AL551427.3  736   ......................................................................................................................................................  885
AL540651.3  752   ......................................................................................................................................................  901
AL551980.3  753   ........................................................W............................................................M................................  902
AL544231.3  752   .......................................................................................................................................-..............  900
AL541874.3  726   ......................................................................................................................................................  875
BU902298.1  765   ..........................................................................................................A....................TG.....................  914
BU508007.1  732   ......................................................................................................................................................  881
AL553114.3  857   .............................................................................................................W..-...........................-..-......  1003

Query       901   CTTTCTTTGCAGAGAAGCTTCATCAAGCCATGAAAGTATGTACCATTCTACTTATATGTCCTGCTTAGAGGAAGAATTATTTGTAGAAAGAACAGAAAACCTCATGTTGTTTGAAAATCTCACATTTAATATCTTCCCATTAATGAGAAT  1050
AL544959.3  894   ....................................                                                                                                                    929
BX438944.2  884   ....................................                                                                                                                    919
AL551427.3  886   ....................................                                                                                                                    921
AL540651.3  902   ....................................                                                                                                                    937
AL551980.3  903   ....................................                                                                                                                    938
AL544231.3  901   ....................................                                                                                                                    936
AL541874.3  876   ....................................                                                                                                                    911
BU902298.1  915   .....C..............................                                                                                                                    950
BU508007.1  882   .........................-..........                                                                                                                    916
AL553114.3  1004  ..........-....-.........-..M-.......                                                                                                                   1036

Query       1051  CATTGTTCTATTTGATGAAAGAGGTAATACACTACTTCTCAGAATAAACTTCTGTTAGATTCGGTGCtttttttACAGATGAGATTGTAAAGTGAGCTGTAAGTGATTTACGGCTCACTTGTGATTTGCTTAAAACATGTGTCACCACGG  1200

Query       1201  AAGGTGATGTACTTTCTTTTTAACGTGTAATTTTAGTGGCCTACTTCCTCTAAGGTTGTGAGTTTTATAATCTCCCACTTTATGAGTCTGATTTGTTGTTTTATTATTCTGTCTTTTATGCCACAGATTAATAGTTGAAACTTTGAAATG  1350

Query       1351  TCATTCTATGATTAAGGATGTAGAATGATGTATTTGAGATCAACTATGTCACTTTAACTTTACTGTCTGACATGCATGGTATTATACAAGGTACCTCTTCTATTCTGACATTTTGAGCCCACAAATGAATAGTTATGAAGTTATCTTCCT  1500

Query       1501  TTTTCTTTGCAGCTCCAAAATAAGGAGGTTTTCagagagagagagaAAGAAAGATCTAACtatttgaataatatgaaggaatattttgaatatatatgaataatttgaataatatgaaatgataGACATGCTAGGTACTGCTCTGTAGTG  1650

Query       1651  GTTAGCATAGTTGCTGCCCCTTTCTGCCATAGTCTAGTGTGATATTTCGTTGGGTAATGATGAATACCTGGTGCCATGGAAGAGAAAAGTGGACATATACTCCAGACTTTGGAAATCCAGGTAGCATTGCTGTCAGCTAAATAACACCTG  1800

Query       1801  AAGTTTAAATAGAAATGAGCAGATGAGGGCAAGAGTGGAAGAGATGGGATAAAGCAAGGGTGGTCCAAGAGGAAGAAGTAACCCACACACAGGCTTGAAGGCAAAAGGTAACATAACCTTCTCAAGACAGTAAGTATAGAGTGATGGTGC  1950

Query       1951  ACACTGGAAGAGATGGTAGAATTAGGCAGGATATTGTAAGCCTTGAAAAAGAATTAGGCAGGATATCGGAAGCCCTGATTAGATTCTATCCTAAGAGCAACAGAAGATCACTGACAGTGTTTTAATAGATAGACTAGTTTATTAGATTTG  2100

Query       2101  CAGTTTAGAAGTTCCtttttttGTAATTATTGGACAGTGTAGAGACCGGATGGTGAGAGATGAGTTAGGAAGTTGTGACAGCTCTCTATACCTACCGCTAATGTAGAGGATTATTTATTTTCATTTCATTACCATTCGTGTAAGgtgtgt  2250
```

```
Query       2251  gtgtgtgtgtgtgtgtgtgtgtgtgtATAGCTAGTTTCTCTATAGAAATTATATGGAAGAGTAAGAAGGCTTCACTTTAAATTCATGTTTTAATGAGAGTATTTCTGAATATGAGACACTTACCCTCATTTATTTTGGCCAGGGTGTTGG  2400
BX438944.2  919                                                                                                                                                 ....K...  926
AL544231.3  936                                                                                                                                                 ........  943
AL541874.3  911                                                                                                                                                 ........  918
BU508007.1  916                                                                                                                                                 ........  923

Query       2401  AACTCGCCATAAGGCATTGATCAGGATTATGGTTTCCCGTTCTGAAATTGACATGAATGATATCAAAGCATTCTATCAGAAGATGTATGGTATCTCCCTTTGCCAAGCCATCCTGGATGAAACCAAAGGAGATTATGAGAAAATCCTGGT  2550
BX438944.2  927   R.............S...........................................D.......R-....Y.............W..KD-.Y.......Y...                                             1030
                                                                           \
                                                                           |
                                                                           A
AL544231.3  944   ................................................................................G.W-......WWY-..............MWY.-........-.......R...-........-..Y..K-  1086
AL541874.3  919   ..................................................................-....-........-.....W..K-......-.......-..M-.-......                                  1029
BU508007.1  924   ........C.....................................................................-......G....G................C..T...G.........C....GA...T..TA...........  1075
                                                \                                             \                  \
                                                |                                             |                  |
                                                G                                             T                  C

Query       2551  GGCTCTTTGTGGAGGAAACTAAACATTCCCTTGATGGTCTCAA  2593
AL544231.3  1087  .......K-...R....                            1102
BU508007.1  1077  ..............................C......C.....  1123
                  \                 \        \      \
                  |                 |        |      |
                  G                 A        T     AG
```

Query= T7-3 [organism=Homo sapiens] anxa1

Length=2231

Sequences producing significant alignments:	Score (Bits)	E Value	Max ident
AL570428.3 AL570428 Homo sapiens PLACENTA COT 25-NORMALIZED Ho...	1849	0.0	99%
AL574991.3 AL574991 Homo sapiens PLACENTA COT 25-NORMALIZED Ho...	1810	0.0	98%
AL576223.3 AL576223 Homo sapiens PLACENTA COT 25-NORMALIZED Ho...	1807	0.0	98%
AL570884.3 AL570884 Homo sapiens PLACENTA COT 25-NORMALIZED Ho...	1792	0.0	97%
AL575425.3 AL575425 Homo sapiens PLACENTA COT 25-NORMALIZED Ho...	1788	0.0	98%
AL553095.3 AL553095 Homo sapiens PLACENTA COT 25-NORMALIZED Ho...	1762	0.0	97%
BX417009.2 BX417009 Homo sapiens PLACENTA Homo sapiens cDNA cl...	1748	0.0	99%
BQ228162.1 AGENCOURT_7258587 NIH_MGC_71 Homo sapiens cDNA clon...	1731	0.0	99%
AL576340.3 AL576340 Homo sapiens PLACENTA COT 25-NORMALIZED Ho...	1729	0.0	97%
AL576603.2 AL576603 Homo sapiens PLACENTA COT 25-NORMALIZED Ho...	1655	0.0	99%

ALIGNMENTS

```
Query       13    TCAGAGAAGAATTTCTCTTTAGTTCTTTGCAAGAAGGTAGAGATAAAGACACTTTTTCAAAAATGGCAATGGTATCAGAATTCCTCAAGCAGGCCTGGTTTATTGAAAATGAAGAGCAGGAATATGTTCAAACTGTGAAGTCATCCAAAG  162
BX417009.2  1     ......................................................................................................................................................  150

Query       163   GTGGTCCCGGATCAGCGGTGAGCCCCTATCCTACCTTCAATCCATCCTCGGATGTCGCTGCCTTGCATAAGGCCATAATGGTTAAAGGTGTGGATGAAGCAACCATCATTGACATTCTAACTAAGCGAAACAATGCACAGCGTCAACAGA  312
BX417009.2  151   ......................................................................................................................................................  300

Query       313   TCAAAGCAGCATATCTCCAGGAAACAGGAAGGTAAGTTAGAGTGGTAAATTTAGATATTTAATTTCAGCATAGTTATACTTAACCATGGATTCGGAAGCACAGTTACCTAGTTCTTTAAGGTTCTAACCACTGTTTTCTCATTACATCTA  462
BX417009.2  301   ......................................................................................................................................................  451
                                              \
                                              |
                                              A

Query       463   TGATTGGGATTGCAGTGTTTATCCACTTTGTTGCAATTTAATCAATTTTATCAAATTTCCTATTTTTATACATTAGTCATCTTGGTGTATATTGTTTGCAGATGTGGTGCTCTGGGGACAAATTTTTAATTTGAACGTAAACATCGAGAT  612
BX417009.2  452   ......................................................................................................................................................  601

Query       613   TGCTGCTGTCAATAAAGAATATGGTCTGATTGAAATGTACTAATATTTAAACTGAACTGTTTAATGCCTAAATTTTATATATAATTCTACTTTAAAAATAAGCCTTATGTCTTTTATACCAAATATGAGTAGTTTTCTAAAGCAATGAAA  762
BX417009.2  602   ......................................................................................................................................................  751

Query       763   TTAGAAAACTATATAATTTCAGTATCTGATTATACTGCTTGTTATTTGAGAAGTACAAACCTCAAAGATTGGAAACATGAATATATTATTTTAAAAGTAATTTTACTTCTGTTTTCTGTTAGCACACAGTCCTCTGTGTTTTGATCTTTG  912
BX417009.2  752   ......................................................................................................................................................  901

Query       913   TGATTTCTCCCCTGTGTATAAGATTACTAACCCTATGCTTTAAGAAGTTATTGATGAATTCAGTAAATTTTATTTTAATCCCTCTAGATCTGGAAGGGTAAGATAATGTTTCAAAATATTTGGTACACTTTGTATGTGAAGGGAAGAAGC  1062
BX417009.2  902   ........-...............-.......-.......-.......-.W........-..........-....                                                                           969

Query       1063  TCTAAAAGATGGTTGGGTTTAGGATGAGTTGTACGATGACCTAAAGTCAGGTAATATCACATTTTTTAACCTAGCATGTTACTTATTGGAAGTCCTGATTCTAATCtttttttttGTAGCCCCTGGATGAAACACTGAAGAAAGCCCTTA  1212
AL570428.3  1026                                                                                                                          ..........................-Y..  997
AL574991.3  1017                                                                                                                       .......-...-R.....-K...-..RS..-..  990
AL576223.3  1018                                                                                                                       .......-.R...................-...  988
AL570884.3  1030                                                                                                                         .......W...............RS....W.  999
                                                                                                                                              \
                                                                                                                                              |
                                                                                                                                              Y
AL575425.3  1037                                                                                                                          ........-..........-........M.  1010
AL553095.3  1020                                                                                                                          ........-R....-YK.......-.....  994
AL576340.3  1011                                                                                                                          ....-....-......-...-...G..-..  987
```

```
Query       1213  CAGGTCACCTTGAGGAGGTTGTTTTAGCTCTGCTAAAAACTCCAGCGCAATTTGATGCTGATGAACTTCGTGCTGCCATGAAGGGCCTTGGAACTGATGAAGATACTCTAATTGAGATTTTGGCATCAAGAACTAACAAAGAAATCAGAG  1362
AL570428.3  996   ...........................................................................................M..........................................................  847
AL574991.3  989   .CR..............-...K.............................................-..................................................................................  842
AL576223.3  987   ..-.D.....WR............-.A....-V..................................-........................................W..AAR....................................  842
AL570884.3  998   ....Y.M.....R...T...K......YY..S.W......C.............................K...S...........................................................................  849
AL575425.3  1009  ......M...................KG.........C.............................C..C...S....A.....................................................................R  860
AL553095.3  993   ..-..............-...-.....-..........................................................................................................................  848
BQ228162.1  12                                                                       ...................................................................................  94
AL576340.3  986   ..-....-.Y...-..-....-........-...-........-......-................-.....-K.......-....-.............-RW..................-.......-...................  853
AL576603.2  917                                                                                   ....-........-...........-WM..............-.............................  849

Query       1363  ACATTAACAGGGTCTACAGAGAGGAACTGAAGAGAGATCTGGCCAAAGACATAACCTCAGACACATCTGGAGATTTTCGGAACGCTTTGCTTTCTCTTGCTAAGGGTGACCGATCTGAGGACTTTGGTGTGAATGAAGACTTGGCTGATT  1512
AL570428.3  846   .....W...................................................................................................................................R............  697
AL574991.3  841   ......................................................................................................................................................  692
AL576223.3  841   ......................................................................................................................................................  692
AL570884.3  848   ......................................................................................................................................................  699
AL575425.3  859   ........................R..............................................................................................R.................B........S...  710
AL553095.3  847   ......................................................................................................................................................  698
BQ228162.1  95    ......................................................................................................................................................  244
AL576340.3  852   ......................................................................................................................................................  703
AL576603.2  848   ......................................................................................................................................................  699

Query       1513  CAGATGCCAGGGCCTTGTATGAAGCAGGAGAAAGGAGAAAGGGGACAGACGTAAACGTGTTCAATACCATCCTTACCACCAGAAGCTATCCACAACTTCGCAGAGTGTTTCAGAAATACACCAAGTACAGTAAGCATGACATGAACAAAG  1662
AL570428.3  696   ...........................................N.............................................................................................A............  547
AL574991.3  691   ......................................................................................................................................................  542
AL576223.3  691   ......................................................................................................................................................  542
AL570884.3  698   ......................................................................................................................................................  549
AL575425.3  709   ...........................................T.............................................................................................N............  560
AL553095.3  697   ......................................................................................................................................................  548
BQ228162.1  245   ......................................................................................................................................................  394
AL576340.3  702   ......................................................................................................................................................  553
AL576603.2  698   ......................................................................................................................................................  549

Query       1663  TTCTGGACCTGGAGTTGAAAGGTGACATTGAGAAATGCCTCACAGCTATCGTGAAGTGCGCCACAAGCAAACCAGCTTTCTTTGCAGAGAAGCTTCATCAAGCCATGAAAGGTGTTGGAACTCGCCATAAGGCATTGATCAGGATTATGG  1812
AL570428.3  546   ......................................................................................................................................................  397
AL574991.3  541   ......................................................................................................................................................  392
AL576223.3  541   ......................................................................................................................................................  392
AL570884.3  548   ......................................................................................................................................................  399
AL575425.3  559   ........................................N.............................................................................................................  410
AL553095.3  547   .............................................................................YY...V...........C.......................V...............................  398
BQ228162.1  395   ......................................................................................................................................................  544
AL576340.3  552   ......................................................................................................................................................  403
AL576603.2  548   ......................................................................................................................................................  399

Query       1813  TTTCCCGTTCTGAAATTGACATGAATGATATCAAAGCATTCTATCAGAAGATGTATGGTATCTCCCTTTGCCAAGCCATCCTGGATGAAACCAAAGGAGATTATGAGAAAATCCTGGTGGCTCTTTGTGGAGGAAACTAAACATTCCCTT  1962
AL570428.3  396   .................A.........................................T..........................................................................................  247
AL574991.3  391   .............................................................................W........................................................................  242
AL576223.3  391   ......................................................................................................................................................  242
AL570884.3  398   ......................................................................................................................................................  249
AL575425.3  409   ......................................................................................................................................................  260
AL553095.3  397   .......S..S..................RK...R...G...R..........................................K...G.......R....................................................  248
BQ228162.1  545   ......................................................................................................................................................  694
AL576340.3  402   .........................................................................................R.......R....................................................  253
AL576603.2  398   ......................................................................................................................................................  249

Query       1963  GATGGTCTCAAGCTATGATCAGAAGACTTTAATTATATATTTTCATCCTATAAGCTTAAATAGGAAAGTTTCTTCAACAGGATTACAGTGTAGCTACCTACATGCTGAAAAATATAGCCTTTAAATCATTTTTATATTATAACTCTGTAT  2112
AL570428.3  246   ......................................................................................................................................................  97
AL574991.3  241   ......Y...............................................................................................................................................  92
AL576223.3  241   ......................................................................................................................................................  92
AL570884.3  248   ......................................................................................................................................................  99
AL575425.3  259   ..................................................................................................N...................................................  110
AL553095.3  247   ......................................................................................................................................................  98
BQ228162.1  695   ......................................................................................................................................................  845
                                                                                  \
                                                                                  |
                                                                                  A
AL576340.3  252   ......................................................................................................................................................  103
AL576603.2  248   ......................................................................................................................................................  99

Query       2113  AATAGAGATAAGTCCATTTTTTAAAAATGTTTTCCCCAAACCATAAAACCCTATACAAGTTGTTCTAGTAACAATACATGAGAAAGATGTCTATGTAGCTGAAAATAAAATGACGTC  2229
AL570428.3  96    T...............................................................................................                       1
AL574991.3  91    ...........................................................................................                            1
AL576223.3  91    ..................................B........................................................                            1
AL570884.3  98    ............................RW....V.............................N....N....W...............A.....                       1
                                                                                    \          \
                                                                                    |          |
                                                                                    N          N
AL575425.3  109   ...........................................................................N............G.....                          17
AL553095.3  97    .............................................................................................G..                       1
                                                                                  \
                                                                                  |
```

```
                                                                                  N
BQ228162.1  846   ....................................................................G...................................................  965
                                                                            \                      \                   \
                                                                            |                      |                   |
                                                                            G                      G                   A
AL576340.3  102   ..................................................................................................                        5
                                                                                            \
                                                                                            |
                                                                                            N
AL576603.2  98    ..........................................................................A.......................                        1
                                                                                           \
                                                                                           |
                                                                                           C

Query= T9-9 [organism=Homo sapiens] anxa1

Length=2698


                                                                   Score        E     Max
Sequences producing significant alignments:                       (Bits)     Value  ident

AL553114.3 AL553114 Homo sapiens PLACENTA COT 25-NORMALIZED Ho...  1604       0.0    100%
AL544959.3 AL544959 Homo sapiens PLACENTA COT 25-NORMALIZED Ho...  1594       0.0    100%
BM458053.1 AGENCOURT_6411336 NIH_MGC_71 Homo sapiens cDNA clon...  1589       0.0    99%
BX438944.2 BX438944 Homo sapiens PLACENTA Homo sapiens cDNA cl...  1587       0.0    100%
AL551427.3 AL551427 Homo sapiens PLACENTA COT 25-NORMALIZED Ho...  1585       0.0    100%
BU902298.1 AGENCOURT_10127317 NIH_MGC_71 Homo sapiens cDNA clo...  1585       0.0    100%
AL544231.3 AL544231 Homo sapiens PLACENTA COT 25-NORMALIZED Ho...  1581       0.0    100%
AL540651.3 AL540651 Homo sapiens PLACENTA Homo sapiens cDNA cl...  1581       0.0    100%
AL553679.3 AL553679 Homo sapiens PLACENTA COT 25-NORMALIZED Ho...  1578       0.0    99%
AL553164.3 AL553164 Homo sapiens PLACENTA COT 25-NORMALIZED Ho...  1576       0.0    100%

ALIGNMENTS

Query       1     AGTGTGAAATCTTCAGCAGAAGAATTTCTCTTTAGTTCTTTGCAAGAAGGTAGAGATAAAGACACTTTTTCAAAAATGGCAATGGTATCAGAATTCCTCAAGCAGGCCTGGTTTATTGAAAATGAAGAGCAGGAATATGTTCAAACTGTG  150
AL553114.3  107   ................-.....................................................................................................................................  255
AL544959.3  1             ........-.....................................................................................................................................  141
BM458053.1  11    ................-.....................................................................................................................................  159
BX438944.2  1                       ....................................................................................................................................  132
AL551427.3  4                        ...................................................................................................................................  134
BU902298.1  24              ......-.....................................................................................................................................  163
                                                                                  \
                                                                                  |
                                                                                  N
AL544231.3  19                      ..T.................................................................................................................................  150
AL540651.3  19                      ..T.................................................................................................................................  150
AL553679.3  1             ........-.....................................................................................................................................  141
AL553164.3  22                        ..................................................................................................................................  151

Query       151   AAGTCATCCAAAGGTGGTCCCGGATCAGCGGTGAGCCCCTATCCTACCTTCAATCCATCCTCGGATGTCGCTGCCTTGCATAAGGCCATAATGGTTAAAGGTGTGGATGAAGCAACCATCATTGACATTCTAACTAAGCGAAACAATGCA  300
AL553114.3  256   ......................................................................................................................................................  405
AL544959.3  142   ......................................................................................................................................................  291
BM458053.1  160   ......................................................................................................................................................  309
BX438944.2  133   ......................................................................................................................................................  282
AL551427.3  135   ......................................................................................................................................................  284
BU902298.1  164   ......................................................................................................................................................  313
AL544231.3  151   ......................................................................................................................................................  300
AL540651.3  151   ......................................................................................................................................................  300
AL553679.3  142   ......................................................................................................................................................  291
AL553164.3  152   ......................................................................................................................................................  301

Query       301   CAGCGTCAACAGATCAAAGCAGCATATCTCCAGGAAACAGGAAAGCCCCTGGATGAAACACTGAAGAAAGCCCTTACAGGTCACCTTGAGGAGGTTGTTTTAGCTCTGCTAAAAACTCCAGCGCAATTTGATGCTGATGAACTTCGTGCT  450
AL553114.3  406   ......................................................................................................................................................  555
AL544959.3  292   ......................................................................................................................................................  441
BM458053.1  310   ......................................................................................................................................................  459
BX438944.2  283   ......................................................................................................................................................  432
AL551427.3  285   ......................................................................................................................................................  434
BU902298.1  314   ......................................................................................................................................................  463
AL544231.3  301   ......................................................................................................................................................  450
AL540651.3  301   ......................................................................................................................................................  450
AL553679.3  292   ......................................................................-...............................................................................  440
AL553164.3  302   ......................................................................................................................................................  451

Query       451   GCCATGAAGGGCCTTGGAACTGATGAAGATACTCTAATTGAGATTTTGGCATCAAGAACTAACAAAGAAATCAGAGACATTAACAGGGTCTACAGAGAGGAACTGAAGAGAGATCTGGCCAAAGACATAACCTCAGACACATCTGGAGAT  600
AL553114.3  556   ......................................................................................................................................................  705
AL544959.3  442   ......................................................................................................................................................  591
BM458053.1  460   ......................................................................................................................................................  609
BX438944.2  433   ......................................................................................................................................................  582
AL551427.3  435   ......................................................................................................................................................  584
BU902298.1  464   ......................................................................................................................................................  613
AL544231.3  451   ......................................................................................................................................................  600
```

```
AL540651.3  451   ......................................................................................................................................................  600
AL553679.3  441   ......................................................................................................................................................  590
AL553164.3  452   ......................................................................................................................................................  601

Query       601   TTTCGGAACGCTTTGCTTTCTCTTGCTAAGGGTGACCGATCTGAGGACTTTGGTGTGAATGAAGACTTGGCTGATTCAGATGCCAGGGCCTTGTATGAAGCAGGAGAAAGGAGAAAGGGGACAGACGTAAACGTGTTCAATACCATCCTT  750
AL553114.3  706   ......................................................................................................................................................  855
AL544959.3  592   ......................................................................................................................................................  741
BM458053.1  610   .......................................................................................T..............................................................  759
BX438944.2  583   ......................................................................................................................................................  732
AL551427.3  585   ......................................................................................................................................................  734
BU902298.1  614   ......................................................................................................................................................  763
AL544231.3  601   ......................................................................................................................................................  750
AL540651.3  601   ......................................................................................................................................................  750
AL553679.3  591   ......................................................................................................................................................  740
AL553164.3  602   ......................................................................................................................................................  751

Query       751   ACCACCAGAAGCTATCCACAACTTCGCAGAGTGTTTCAGAAATACACCAAGTACAGTAAGCATGACATGAACAAAGTTCTGGACCTGGAGTTGAAAGGTGACATTGAGAAATGCCTCACAGCTATCGGTATGTAGTCCAGCAGTTGAAAG  900
AL553114.3  856   ..............................................................................................................W..-.............                        981
AL544959.3  742   ...............................................................................................................................                        869
                                                                                            \
                                                                                            |
                                                                                            T
BM458053.1  760   ................................................C..............................................................................                        889
                                       \                                                            \                                       \
                                       |                                                            |                                       |
                                       C                                                            A                                       C
BX438944.2  733   ...............................................................................................................................                        859
AL551427.3  735   ...............................................................................................................................                        861
BU902298.1  764   ...........................................................................................................A...................                        890
AL544231.3  751   ...............................................................................................................................                        877
AL540651.3  751   ...............................................................................................................................                        877
AL553679.3  741   ..............................................................................................................T................                        869
                                                                                       \                 \
                                                                                       |                 |
                                                                                       T                 T
AL553164.3  752   .................................................................................................................-.............                        877

Query       901   AGTTTTCTAACTAGAAATTGTATTTTCAAAGCTATCCTATACTTaacaagaacaacagcaacaaaaccaataaaagaaaacaaaaaacaaTCAGAAGTAGTGCATCTGTTTCAATTGAAGCAACGTAAAAGATTGGCAAAGTACTATAAC  1050

Query       1051  CTTAATAACAATAGATATTATTCCCCTTTTCAAAAATAGAGAGAGATAGACTATACTTATTGAGCACCTACTTATATCAAACAATGTACCAGGCTGGGCACACCACAGAGGCAATTCAGAATGGTCACTGAGAGCATGGGATCTGGAATA  1200

Query       1201  AATGTAACATCTGGATAAATGTAACTTCAAGTCCTGGTCTACTGCTTGTTAATTGTACAATCTTGGGCAGGTCCAGGAGAGTCTGATTCAGTGGGTCCGAGTTGGCCTCTAAAAATCGGTACTTTTTAAAGATCTCCCAGGTTATTCAGA  1350

Query       1351  CAACCATCAGGGTTGGGGAACCTATGTTCTCAAGCAGTGTTTCTCATTCATGACTATGCACTTGAATCCCCTGGAAAGCTTGTTAAAATGCAGATTCTGATTCCCCAGGTCTGGGGTGGAGCTTAGATTCTGCTTTTCTAACAAGCTCCC  1500

Query       1501  CATTGATGCCAATGCTGTTGGTCCATAGATCTCGACAAGATGAAGAACACTATGAACTGGAATGTGCTAGTATTATTTGGGTTCTGTTTTATATTTAAGCCCATCCTATCCCAGAGttttttcttcatttattcatttatttttacttat  1650

Query       1651  ttgtttGTGCAACTCTTACTTTAGACACAGAGGTACATGTGCACGTTTGTTCCATGGGTGTTCTGCACTCAGGCAGTGAGCATAGTACCCAATAAGTAGTTTTTTGACCAATGCCCCTTCTCTCCCTGCCCTCTCTAGTAGTCTGCAGTG  1800

Query       1801  TCTGTTGTTCATAGGGTGTTCTTTTTAAATGTTCTTATAGTAACATAAATAAAGTATTTACATAGGCTTGAACACCTAGTGGCTTCTCCACTCCCAAGGAGAATATATGTTGGATAGCGCAAACTACTGAAGTTGCTCTTGAGATGTATT  1950

Query       1951  TAAGGTAATaaaaaaaTTGATCGTCAGGGAAAAGAATATTCAAAACCACCATCTAGAGAGTCACAAATACCTTCTCAGAAGTTAACTGCCCTATATTGGGAGATGAGAGGTGAAGAATGATGATGAGGGATAGAACACTGTTGGCAAAAT  2100

Query       2101  TCTATATTTCCTGTTTCTTTCATATGGATATTTCATTTTTTCATGGTAAATACATCAACTAAAATTTTCTTCTCTAACAGTGAAGTGCGCCACAAGCAAACCAGCTTTCTTTGCAGAGAAGCTTCATCAAGCCATGAAAGGTGTTGGAAC  2250
AL544959.3  869                                                                                  .............M.......M..........................................G......  939
BX438944.2  859                                                                                  ................................................................K...R..  929
AL551427.3  861                                                                                  ....................................................................-..  930
BU902298.1  894                                                                                      ..........................C........................................  960
AL544231.3  877                                                                                  ..........-............................................................  946
AL540651.3  877                                                                                  .............................................................          937
AL553164.3  877                                                                                  ..........-.......-...............................-.-.....-.....        936

Query       2251  TCGCCATAAGGCATTGATCAGGATTATGGTTTCCCGTTCTGAAATTGACATGAATGATATCAAAGCATTCTATCAGAAGATGTATGGTATCTCCCTTTGCCAAGCCATCCTGGATGAAACCAAAGGAGATTATGAGAAAATCCTGGTGGC  2400
AL544959.3  940   ..............KR............-..........R....                                                                                                          983
BX438944.2  930   ...........S.........................................D.......R-....Y.............W..KD-.Y.......Y...                                                  1030
                                                                       \
                                                                       |
                                                                       A
AL551427.3  931   .......................W....-....G............-.W.G....W..-....M..-....M...R-........G.WY...-.....M.S-.-..........                                   1038
BU902298.1  961   .........C..........GA.....G..................T.......A...C....T........A....A.G....A.......C...G...C..C......T.........G...A.......A.......C..G...  1116
                                                                \                  \                  \           \                 \              \
                                                                |                  |                  |           |                 |              |
                                                                G                  A                  C           G                 G              G
AL544231.3  947   .............................................................................G.W-......WWY-..............MWY.-........-.......R...-........-..Y..K-...  1089

Query       2401  TCTTTGTGGAGGAAACTAAACATTCCCTTGA  2431
BU902298.1  1118  .........A....AA.............  1152
                  \        \         \  \  \
                  |        |         |  |  |
                  C        G         T  A  T
AL544231.3  1090  ....K-...R....                 1102
```

Query= T10-C* [organism=Homo sapiens] anxa1

Length=1518

```
                                                                   Score        E     Max
Sequences producing significant alignments:                       (Bits)     Value  ident

AL542692.3 AL542692 Homo sapiens PLACENTA Homo sapiens cDNA cl...  1914       0.0    98%
AL570428.3 AL570428 Homo sapiens PLACENTA COT 25-NORMALIZED Ho...  1910       0.0    98%
BU508007.1 AGENCOURT_10128424 NIH_MGC_71 Homo sapiens cDNA clo...  1858       0.0    98%
AL544231.3 AL544231 Homo sapiens PLACENTA COT 25-NORMALIZED Ho...  1834       0.0    98%
AL576223.3 AL576223 Homo sapiens PLACENTA COT 25-NORMALIZED Ho...  1831       0.0    98%
AL570884.3 AL570884 Homo sapiens PLACENTA COT 25-NORMALIZED Ho...  1825       0.0    97%
AL574991.3 AL574991 Homo sapiens PLACENTA COT 25-NORMALIZED Ho...  1810       0.0    98%
AL541874.3 AL541874 Homo sapiens PLACENTA Homo sapiens cDNA cl...  1792       0.0    99%
DC631365.1 DC631365 macaque bone marrow cDNA library QbmA Maca...  1788       0.0    94%
AL575425.3 AL575425 Homo sapiens PLACENTA COT 25-NORMALIZED Ho...  1788       0.0    98%

ALIGNMENTS

Query       1     AGTGTGAAATCTTCAGAGAAGAATTTCTCTTTAGTTCTTTGCAAGAAGGTAGAGATAAAGAGTCTTGTTATGTCGCCCAGGATGGAGTGTAGTGGTACAATCTCGGCTCACTGCAGCCTCCGCCTCTTCCTGGGTTCAAGTGATTCTCCT  150
AL542692.3  19                     ..T..................................................................................................................................  151
BU508007.1  1                         .........................................                                                                                         41
AL544231.3  19                     ..T.........................................                                                                                         62
AL541874.3  1                              ....................................                                                                                         36
DC631365.1  1     ......-....................C.C...............................                                                                                         60

Query       151   GCCTCAGCCTTCTGAGTAGATGGGACTACAGACACTTTTTCAAAAATGGCAATGGTATCAGAATTCCTCAAGCAGGCCTGGTTTATTGAAAATGAAGAGCAGGAATATGTTCAAACTGTGAAGTCATCCAAAGGTGGTCCCGGATCAGCG  300
AL542692.3  152   ......................................................................................................................................................  301
BU508007.1  36                              ..A.........................................................................................................................  160
                                                  \
                                                  |
                                                  N
AL544231.3  57                              ..A.........................................................................................................................  180
AL541874.3  31                              ..A.........................................................................................................................  154
DC631365.1  55                              ..A.............................................................................................A.................T........A  178

Query       301   GTGAGCCCCTATCCTACCTTCAATCCATCCTCGGATGTCGCTGCCTTGCATAAGGCCATAATGGTTAAAGGTGTGGATGAAGCAACCATCATTGACATTCTAACTAAGCGAAACAATGCACAGCGTCAACAGATCAAAGCAGCATATCTC  450
AL542692.3  302   ......................................................................................................................................................  451
AL570428.3  1088                                                                                                     .....-.T...........-.....T....-.A..-..........W....  1041
BU508007.1  161   ......................................................................................................................................................  310
AL544231.3  181   ......................................................................................................................................................  330
AL576223.3  1050                                                                                                                                ......-W......-R...-.Y..  1030
AL570884.3  1059                                                                                                                                          .........T....  1045
                                                                                                                                                              \
                                                                                                                                                              |
                                                                                                                                                              M
AL541874.3  155   ......................................................................................................................................................  304
DC631365.1  179   ......................................T...........C...................................................................................................  328

Query       451   CAGGAAACAGGAAAGCCCCTGGATGAAACACTGAAGAAAGCCCTTACAGGTCACCTTGAGGAGGTTGTTTTAGCTCTGCTAAAAACTCCAGCGCAATTTGATGCTGATGAACTTCGTGCTGCCATGAAGGGCCTTGGAACTGATGAAGAT  600
AL542692.3  452   ......................................................................................................................................................  601
AL570428.3  1040  ...-.........GC...........................-Y.............................................................................................M............  893
BU508007.1  311   ......................................................................................................................................................  460
AL544231.3  331   ......................................................................................................................................................  480
AL576223.3  1029  ..-........-........-.R...................-.....-.D.....WR............-.A...-V...................................-....................................  888
AL570884.3  1044  .T............C...Y...W...............RS....W.....Y.M.....R...T...K......YY..S.W......C.............................K...S.............................  895
AL574991.3  1017               .......-...-R.....-K...-..RS..-...CR...............-...K............................................-....................................  888
AL541874.3  305   ......................................................................................................................................................  454
DC631365.1  329   ..............................................TG....................................................................G................................C  478
AL575425.3  1037                  ........-..........-........M.......M...................KG.........C.............................C..C..S...A..........................  906

Query       601   ACTCTAATTGAGATTTTGGCATCAAGAACTAACAAAGAAATCAGAGACATTAACAGGGTCTACAGAGAGGAACTGAAGAGAGATCTGGCCAAAGACATAACCTCAGACACATCTGGAGATTTTCGGAACGCTTTGCTTTCTCTTGCTAAG  750
AL542692.3  602   ......................................................................................................................................................  751
AL570428.3  892   ...................................................W..................................................................................................  743
BU508007.1  461   ......................................................................................................................................................  610
AL544231.3  481   ......................................................................................................................................................  630
AL576223.3  887   ....W..AAR............................................................................................................................................  738
AL570884.3  894   ......................................................................................................................................................  745
AL574991.3  887   ......................................................................................................................................................  738
AL541874.3  455   ......................................................................................................................................................  604
DC631365.1  479   .....G.............................G..................................................................................................................  628
AL575425.3  905   .............................................R........................R..............................................................................  756

Query       751   GGTGACCGATCTGAGGACTTTGGTGTGAATGAAGACTTGGCTGATTCAGATGCCAGGGCCTTGTATGAAGCAGGAGAAAGGAGAAAGGGGACAGACGTAAACGTGTTCAATACCATCCTTACCACCAGAAGCTATCCACAACTTCGCAGA  900
AL542692.3  752   ..........................................................................................R...........................................................  901
AL570428.3  742   .................................R.......................................................N............................................................  593
BU508007.1  611   ......................................................................................................................................................  760
AL544231.3  631   ......................................................................................................................................................  780
AL576223.3  737   ......................................................................................................................................................  588
AL570884.3  744   ......................................................................................................................................................  595
```

```
AL574991.3  737   ......................................................................................................................................................  588
AL541874.3  605   ......................................................................................................................................................  754
DC631365.1  629   .......................................................................G.......................T..............................C.............T.........  778
AL575425.3  755   ...............R.................B........S..............................................T............................................................  606

Query       901   GTGTTTCAGAAATACACCAAGTACAGTAAGCATGACATGAACAAAGTTCTGGACCTGGAGTTGAAAGGTGACATTGAGAAATGCCTCACAGCTATCGTGAAGTGCGCCACAAGCAAACCAGCTTTCTTTGCAGAGAAGCTTCATCAAGCC  1050
AL542692.3  902   ....................................................................K...........W......M...........R............C....M.....YY.........................  1052
                                                                          \
                                                                          |
                                                                          G
AL570428.3  592   .................................A......................................................................................................................  443
BU508007.1  761   .................................................................................................................................................-....  909
AL544231.3  781   ..........................................................................................................-...........................................  929
AL576223.3  587   ......................................................................................................................................................  438
AL570884.3  594   ......................................................................................................................................................  445
AL574991.3  587   ......................................................................................................................................................  438
AL541874.3  755   ......................................................................................................................................................  904
DC631365.1  779   ........................................................................................................T................................A........G...  928
AL575425.3  605   .................................N....................................................N...............................................................  456

Query       1051  ATGAAAGGTGTTGGAACTCGCCATAAGGCATTGATCAGGATTATGGTTTCCCGTTCTGAAATTGACATGAATGATATCAAAGCATTCTATCAGAAGATGTATGGTATCTCCCTTTGCCAAGCCATCCTGGATGAAACCAAAGGAGATTAT  1200
AL542692.3  1053  .......G..-..R.MY...M-.....M-.........R........-....S.-...R.....                                                                                        1111
AL570428.3  442   ...............................................................A.........................................T............................................  293
BU508007.1  910   ......................C.....................................................................-......G....G...............C..T...G..........C....GA...T.  1061
                                                              \                                             \                  \
                                                              |                                             |                  |
                                                              G                                             T                  C
AL544231.3  930   ..............................................................................................G.W-......WWY-..............MWY.-........-.......R...-..  1074
AL576223.3  437   ......................................................................................................................................................  288
AL570884.3  444   ......................................................................................................................................................  295
AL574991.3  437   ...........................................................................................................................W..........................  288
AL541874.3  905   ................................................................................-....-........-.....W..K-......-.......-..M-..-......                   1029
DC631365.1  929   ..........C.......................................A..........................................-.........................-......-..-.....-.-.--......-..  1070
                                                              \
                                                              |
                                                              G
AL575425.3  455   ......................................................................................................................................................  306

Query       1201  GAGAAAATCCTGGTGGCTCTTTGTGGAGGAAACTAAACATTCCCTTGATGGTCTCAAGCTATGATCAGAAGACTTTAATTATATATTTTCATCCTATAAGCTTAAATAGGAAAGTTTCTTCAACAGGATTACAGTGTAGCTACCTACATG  1350
AL570428.3  292   ......................................................................................................................................................  143
BU508007.1  1062  .TA.........................................C......C.....                                                                                               1123
                                \                 \        \      \
                                |                 |        |      |
                                G                 A        T     AG
AL544231.3  1075  ......-..Y..K-.......K-...R....                                                                                                                         1102
AL576223.3  287   ......................................................................................................................................................  138
AL570884.3  294   ......................................................................................................................................................  145
AL574991.3  287   ....................................................Y.................................................................................................  138
DC631365.1  1071  ..-...-..-....-.............-..-....-...-....-....A-...-.......................-........CA...G..-.-..C...-.-..C.-...-..-.--......-..C..--.........T-..  1196
                                                                                        \
                                                                                        |
                                                                                        A
AL575425.3  305   ................................................................................................................................................N.....  156

Query       1351  CTGAAAAATATAGCCTTTAAATCATTTTTATATTATAACTCTGTATAATAGAGATAAGTCCATTTTTTAAAAATGTTTTCCCCAAACCATAAAACCCTATACAAGTTGTTCTAGTAACAATACATGAGAAAGATGTCTATGT  1492
AL570428.3  142   ..............................................T...............................................................................................  1
AL576223.3  137   ................................................................................B........................................................       1
AL570884.3  144   ..........................................................................RW....V.............................N....N....W...............A.....  1
                                                                                                                                  \          \
                                                                                                                                  |          |
                                                                                                                                  N          N
AL574991.3  137   .........................................................................................................................................       1
DC631365.1  1197  ....C...-.....-.A....                                                                                                                              1216
                   \
                   |
                   G
AL575425.3  155   ........................................................................................................................N............G.....     17
```

Query= T11-16 [organism=Homo sapiens] anxa1

Length=1927

Sequences producing significant alignments:	Score (Bits)	E Value	Max ident
BX417009.2 BX417009 Homo sapiens PLACENTA Homo sapiens cDNA cl...	1753	0.0	99%
AU134300.1 AU134300 OVARC1 Homo sapiens cDNA clone OVARC100168...	1450	0.0	99%
AU138502.1 AU138502 PLACE1 Homo sapiens cDNA clone PLACE100869...	1315	0.0	98%
CA421424.1 UI-H-EZ1-bbb-c-06-0-UI.s1 NCI_CGAP_Ch2 Homo sapiens...	1284	0.0	100%
AI764962.1 wh57a06.x1 NCI_CGAP_Kid11 Homo sapiens cDNA clone I...	1205	0.0	97%
BF107418.1 601824036F1 NIH_MGC_79 Homo sapiens cDNA clone IMAG...	1064	0.0	98%

```
DB013065.1 DB013065 TESOP2 Homo sapiens cDNA clone TESOP200154...  1040       0.0    100%
DB328492.1 DB328492 PLACE6 Homo sapiens cDNA clone PLACE600972...  948        0.0    100%
BG231632.1 naf37c05.x1 Soares_NPBMC Homo sapiens cDNA clone IM...  942        0.0    100%
BU674442.1 UI-CF-DU0-aab-j-09-0-UI.s1 UI-CF-DU0 Homo sapiens c...  935        0.0    100%

ALIGNMENTS

Query       1     AGTGTGAAATCTTCAGAGAAGAATTTCTCTTTAGTTCTTTGCAAGAAGGTAGAGATAAAGACACTTTTTCAAAAATGGCAATGGTATCAGAATTCCTCAAGCAGGCCTGGTTTATTGAAAATGAAGAGCAGGAATATGTTCAAACTGTGA  150
BX417009.2  1                 ..........................................................................................................................................  138
AU134300.1  1     ......................................................................................................................................................  150
AU138502.1  1     ..................................................................C...................................................................................  150
BF107418.1  1                      .....................................................................................................................................  133

Query       151   AGTCATCCAAAGGTGGTCCCGGATCAGCGGTGAGCCCCTATCCTACCTTCAATCCATCCTCGGATGTCGCTGCCTTGCATAAGGCCATAATGGTTAAAGGTGTGGATGAAGCAACCATCATTGACATTCTAACTAAGCGAAACAATGCAC  300
BX417009.2  139   ......................................................................................................................................................  288
AU134300.1  151   ......................................................................................................................................................  300
AU138502.1  151   ......................................................................................................................................................  300
BF107418.1  134   ......................................................................................................................................................  283

Query       301   AGCGTCAACAGATCAAAGCAGCATATCTCCAGGAAACAGGAAAGGTAAGTTAGAGTGGTAAATTTAGATATTTAATTTCAGCATAGTTATACTTAACCATGGATTCGGAAGCACAGTTACCTAGTTCTTTAAGGTTCTAACCACTGTTTT  450
BX417009.2  289   ......................................................................................................................................................  438
AU134300.1  301   ......................................................................................................................................................  450
AU138502.1  301   ......................................................................................................................................................  450
BF107418.1  284   ......................................................................................................................................................  433

Query       451   CTCATTACATCTATGATTGGGATTGCAGTGTTTATCCACTTTGTTGCAATTTAATCAATTTTATCAAATTTCCTATTTTTATACATTAGTCATCTTGGTGTATATTGTTTGCAGATGTGGTGCTCTGGGGACAAATTTTTAATTTGAACG  600
BX417009.2  439   ......................................................................................................................................................  588
AU134300.1  451   ...........................................................................................................N..........................................  600
AU138502.1  451   ......................................................................................................................................................  600
BF107418.1  434   .................G............................-...................................................-......-.-..........................................  579
DB013065.1  1                                                                                             ..............................................................  62

Query       601   TAAACATCGAGATTGCTGCTGTCAATAAAGAATATGGTCTGATTGAAATGTACTAATATTTAAACTGAACTGTTTAATGCCTAAATTTTATATATAATTCTACTTTAAAAATAAGCCTTATGTCTTTTATACCAAATATGAGTAGTTTTC  750
BX417009.2  589   ......................................................................................................................................................  738
AU134300.1  601   ...................................................-.........................................................................................N...N....  749
AU138502.1  601   .................................................................................................A........................G........N........G....N....  760
                                                 \                                    \                                 \          \  \        \            \\   \
                                                 |                                    |                                 |          |  |        |            ||   |
                                                 G                                    G                                 C          A  A        C            A|  NG
                                                                                                                                                                |
                                                                                                                                                                T

BF107418.1  580   .T..AT...........TG..G....C....                                                                                                                         610
DB013065.1  63    ......................................................................................................................................................  213
                                                                                        \
                                                                                        |
                                                                                        T

Query       751   TAAAGCAATGAAATTAGAAAACTATATAATTTCAGTATCTGATTATACTGCTTGTTATTTGAGAAGTACAAACCTCAAAGATTGGAAACATGAATATATTATTTTAAAAGTAATTTTACTTCTGTTTTCTGTTAGCACACAGTCCTCTGT  900
BX417009.2  739   ......................................................................................................................................................  888
AU134300.1  750   .........................N.................-......                                                                                                      798
AU138502.1  761   C........                                                                                                                                               770
                       \
                       |
                       G
DB013065.1  214   ......................................................................................................................................................  363

Query       901   GTTTTGATCTTTGTGATTTCTCCCCTGTGTATAAGATTACTAACCCTATGCTTTAAGAAGTTATTGATGAATTCAGTAAATTTTATTTTAATCCCTCTAGATCTGGAAGGGTAAGATAATGTTTCAAAATATTTGGTACACTTTGTATGT  1050
BX417009.2  889   .....................-...............-.......-.......-.......-.W........-..........-....                                                                969
DB013065.1  364   ......................................................................................................................................................  513

Query       1051  GAAGGGAAGAAGCTCTAAAAGATGGTTGGGTTTAGGGATGAGTTGTACGATGACCTAAAGTCAGGTAATATCACATTTTTTAACCTAGCATGTTACTTATTGGAAGTCCTGATTCTAATCtttttttttGTAGCCCCTGGATGAAACACT  1200
DB013065.1  514   ......................................................                                                                                                  567

Query       1201  GAAGAAAGCCCTTACAGGTCACCTTGAGGAGGTTGTTTTAGCTCTGCTAAAAACTCCAGCGCAATTTGATGCTGATGAACTTCGTGCTGCCATGAAGGGCCTTGGAACTGATGAAGATACTCTAATTGAGATTTTGGCATCAAGAACTAA  1350
CA421424.1  727                            .....-....-..................................................................................................................  605
AI764962.1  731    ...-...............C....A.......-..................C...-.....................CT..................C...................................G.C.......-...T.  586
                                                                              \
                                                                              |
                                                                              G

Query       1351  CAAAGAAATCAGAGACATTAACAGGGTCTACAGAGAGGAACTGAAGAGAGATCTGGCCAAAGACATAACCTCAGACACATCTGGAGATTTTCGGAACGCTTTGCTTTCTCTTGCTAAGGGTGACCGATCTGAGGACTTTGGTGTGAATGA  1500
CA421424.1  604   ......................................................................................................................................................  455
AI764962.1  585   .C.G...T......C.....C........C.........C...............-..C.......T...................................................................................  437
DB328492.1  528                                                                   ....................................................................................  443
BG231632.1  525                                                                      .................................................................................  443
BU674442.1  536                                                                       ................................................................................  455

Query       1501  AGACTTGGCTGATTCAGATGCCAGGGCCTTGTATGAAGCAGGAGAAAGGAGAAAGGGGACAGACGTAAACGTGTTCAATACCATCCTTACCACCAGAAGCTATCCACAACTTCGCAGAGGTAACAATAAATTTCTTTTTCTGGAATCTGT  1650
CA421424.1  454   ......................................................................................................................................................  305
AI764962.1  436   ......................................................................................................................................................  287
DB328492.1  442   ......................................................................................................................................................  293
BG231632.1  442   ......................................................................................................................................................  293
```

```
BU674442.1  454   .........................C............................................................................................................................  305

Query       1651  TTATGGAAGATGCAATTTTCTTTTTTGATGACAAATAAGAGAAAGTAAAAACAGAACCCTTTTTCAATATCACTCCTGTATCAAACAGATACAGTGTTCCTGACCCATTGTTATTTGTCAATTTGTCCAATTTTGTACAGCCGCTACTTT  1800
CA421424.1  304   ......................................................................................................................................................  155
AI764962.1  286   ......................................................................................................................................................  137
DB328492.1  292   ......................................................................................................................................................  143
BG231632.1  292   ......................................................................................................................................................  143
BU674442.1  304   ......................................................................................................................................................  155

Query       1801  ATCATATTTAATACATTTTGTGACTTGGGCATGTGGAACATAATTTTGTTATTTTCACTGCTTTTACTCCCCTAAAAGATTTGGAATATGTGTGGGAAAAGACATGCATAACAGAACAAATGAAATT  1927
CA421424.1  154   ...............................................................................................................................  28
AI764962.1  136   ...............................................................................................................................  10
DB328492.1  142   ...............................................................................................................................  16
BG231632.1  142   ...............................................................................................................................  16
BU674442.1  154   ...............................................................................................................................  28
```

Query= T12-2 [organism=Homo sapiens] anxa1

Length=1027

```
                                                                   Score        E     Max
Sequences producing significant alignments:                       (Bits)     Value  ident

CB306442.1 UI-CF-EN1-aej-e-15-0-UI.s1 UI-CF-EN1 Homo sapiens c...  1279       0.0    100%
CA447733.1 UI-H-EI0-ayf-a-16-0-UI.s1 NCI_CGAP_EI0 Homo sapiens...  1279       0.0    100%
CA447223.1 UI-H-EI0-ayd-b-19-0-UI.s1 NCI_CGAP_EI0 Homo sapiens...  1279       0.0    100%
CA422748.1 UI-H-FL0-bdr-g-09-0-UI.s1 NCI_CGAP_FL0 Homo sapiens...  1279       0.0    100%
CA419844.1 UI-H-FH0-bcm-d-16-0-UI.s1 NCI_CGAP_FH0 Homo sapiens...  1279       0.0    100%
CA417462.1 UI-H-FE0-bbw-c-13-0-UI.s1 NCI_CGAP_FE0 Homo sapiens...  1279       0.0    100%
CA415613.1 UI-H-EZ0-bay-p-10-0-UI.s1 NCI_CGAP_Ch1 Homo sapiens...  1279       0.0    100%
CA414702.1 UI-H-EZ0-bar-e-18-0-UI.s1 NCI_CGAP_Ch1 Homo sapiens...  1279       0.0    100%
BU633206.1 UI-H-FL1-bgt-f-12-0-UI.s1 NCI_CGAP_FL1 Homo sapiens...  1279       0.0    100%
BQ776306.1 UI-H-FH0-bcn-i-12-0-UI.s1 NCI_CGAP_FH0 Homo sapiens...  1279       0.0    100%
```

ALIGNMENTS

```
Query       330   CAGGAAACAGGAGAAAGGGGACAGACGTAAACGTGTTCAATACCATCCTTACCACCAGAAGCTATCCACAACTTCGCAGAGTGTTTCAGAAATACACCAAGTACAGTAAGCATGACATGAACAAAGTTCTGGACCTGGAGTTGAAAGGTG  479
CB306442.1  716   .....G.A..............................................................................................................................................  567
CA447733.1  716   .....G.A..............................................................................................................................................  567
CA447223.1  716   .....G.A..............................................................................................................................................  567
CA422748.1  718   .....G.A..............................................................................................................................................  569
CA419844.1  716   .....G.A..............................................................................................................................................  567
CA417462.1  713   .....G.A..............................................................................................................................................  564
CA415613.1  718   .....G.A..............................................................................................................................................  569
CA414702.1  718   .....G.A..............................................................................................................................................  569
BU633206.1  716   .....G.A..............................................................................................................................................  567
BQ776306.1  716   .....G.A..............................................................................................................................................  567

Query       480   ACATTGAGAAATGCCTCACAGCTATCGTGAAGTGCGCCACAAGCAAACCAGCTTTCTTTGCAGAGAAGCTTCATCAAGCCATGAAAGGTGTTGGAACTCGCCATAAGGCATTGATCAGGATTATGGTTTCCCGTTCTGAAATTGACATGA  629
CB306442.1  566   ......................................................................................................................................................  417
CA447733.1  566   ......................................................................................................................................................  417
CA447223.1  566   ......................................................................................................................................................  417
CA422748.1  568   ......................................................................................................................................................  419
CA419844.1  566   ......................................................................................................................................................  417
CA417462.1  563   ......................................................................................................................................................  414
CA415613.1  568   ......................................................................................................................................................  419
CA414702.1  568   ......................................................................................................................................................  419
BU633206.1  566   ......................................................................................................................................................  417
BQ776306.1  566   ......................................................................................................................................................  417

Query       630   ATGATATCAAAGCATTCTATCAGAAGATGTATGGTATCTCCCTTTGCCAAGCCATCCTGGATGAAACCAAAGGAGATTATGAGAAAATCCTGGTGGCTCTTTGTGGAGGAAACTAAACATTCCCTTGATGGTCTCAAGCTATGATCAGAA  779
CB306442.1  416   ......................................................................................................................................................  267
CA447733.1  416   ......................................................................................................................................................  267
CA447223.1  416   ......................................................................................................................................................  267
CA422748.1  418   ......................................................................................................................................................  269
CA419844.1  416   ......................................................................................................................................................  267
CA417462.1  413   ......................................................................................................................................................  264
CA415613.1  418   ......................................................................................................................................................  269
CA414702.1  418   ......................................................................................................................................................  269
BU633206.1  416   ......................................................................................................................................................  267
BQ776306.1  416   ......................................................................................................................................................  267

Query       780   GACTTTAATTATATATTTTCATCCTATAAGCTTAAATAGGAAAGTTTCTTCAACAGGATTACAGTGTAGCTACCTACATGCTGAAAAATATAGCCTTTAAATCATTTTTATATTATAACTCTGTATAATAGAGATAAGTCCATTTTTTAA  929
CB306442.1  266   ......................................................................................................................................................  117
CA447733.1  266   ......................................................................................................................................................  117
CA447223.1  266   ......................................................................................................................................................  117
CA422748.1  268   ......................................................................................................................................................  119
CA419844.1  266   ......................................................................................................................................................  117
CA417462.1  263   ......................................................................................................................................................  114
CA415613.1  268   ......................................................................................................................................................  119
CA414702.1  268   ......................................................................................................................................................  119
```

```
BU633206.1  266   ......................................................................................................................................................  117
BQ776306.1  266   ......................................................................................................................................................  117

Query       930   AAATGTTTTCCCCAAACCATAAAACCCTATACAAGTTGTTCTAGTAACAATACATGAGAAAGATGTCTATGTAGCTGAAAATAAAATGACGTCACAAG  1027
CB306442.1  116   ..................................................................................................  19
CA447733.1  116   ..................................................................................................  19
CA447223.1  116   ..................................................................................................  19
CA422748.1  118   ..................................................................................................  21
CA419844.1  116   ..................................................................................................  19
CA417462.1  113   ..................................................................................................  16
CA415613.1  118   ..................................................................................................  21
CA414702.1  118   ..................................................................................................  21
BU633206.1  116   ..................................................................................................  19
BQ776306.1  116   ..................................................................................................  19

Query= T13-10 [organism=Homo sapiens] anxa1

Length=1277


                                                                   Score        E     Max
Sequences producing significant alignments:                       (Bits)     Value  ident

BQ228162.1 AGENCOURT_7258587 NIH_MGC_71 Homo sapiens cDNA clon...  1679       0.0    99%
AL574991.3 AL574991 Homo sapiens PLACENTA COT 25-NORMALIZED Ho...  1648       0.0    100%
BM555842.1 AGENCOURT_6544353 NIH_MGC_88 Homo sapiens cDNA clon...  1639       0.0    99%
AL576603.2 AL576603 Homo sapiens PLACENTA COT 25-NORMALIZED Ho...  1635       0.0    99%
AL576223.3 AL576223 Homo sapiens PLACENTA COT 25-NORMALIZED Ho...  1633       0.0    99%
AL570428.3 AL570428 Homo sapiens PLACENTA COT 25-NORMALIZED Ho...  1631       0.0    99%
AL570884.3 AL570884 Homo sapiens PLACENTA COT 25-NORMALIZED Ho...  1628       0.0    99%
AL576340.3 AL576340 Homo sapiens PLACENTA COT 25-NORMALIZED Ho...  1624       0.0    99%
AL575425.3 AL575425 Homo sapiens PLACENTA COT 25-NORMALIZED Ho...  1615       0.0    99%
AL553095.3 AL553095 Homo sapiens PLACENTA COT 25-NORMALIZED Ho...  1587       0.0    98%

ALIGNMENTS

Query       351   GATGAAGATACTCTAATTGAGATTTTGGCATCAAGAACTAACAAAGAAATCAGAGACATTAACAGGGTCTACAGAGAGGAACTGAAGAGAGATCTGGCCAAAGACATAACCTCAGACACATCTGGAGATTTTCGGAACGCTTTGCTTTCT  500
BQ228162.1  40    ......................................................................................................................................................  189
AL574991.3  896   ......................................................................................................................................................  747
BM555842.1  1       ....................................................................................................................................................  148
AL576603.2  901   ........-WM..............-............................................................................................................................  754
AL576223.3  896   .............W..AAR...................................................................................................................................  747
AL570428.3  901   ............................................................W.........................................................................................  752
AL570884.3  903   ......................................................................................................................................................  754
AL576340.3  896            ..................-.......-..................................................................................................................  758
AL575425.3  914   ......................................................R........................R......................................................................  765
AL553095.3  902   ......................................................................................................................................................  753

Query       501   CTTGCTAAGGGTGACCGATCTGAGGACTTTGGTGTGAATGAAGACTTGGCTGATTCAGATGCCAGGGCCTTGTATGAAGCAGGAGAAAGGAGAAAGGGGACAGACGTAAACGTGTTCAATACCATCCTTACCACCAGAAGCTATCCACAA  650
BQ228162.1  190   ......................................................................................................................................................  339
AL574991.3  746   ......................................................................................................................................................  597
BM555842.1  149   ......................................................................................................................................................  298
AL576603.2  753   ......................................................................................................................................................  604
AL576223.3  746   ......................................................................................................................................................  597
AL570428.3  751   ..........................................R.......................................................N...................................................  602
AL570884.3  753   ......................................................................................................................................................  604
AL576340.3  757   ......................................................................................................................................................  608
AL575425.3  764   ........................R.................B........S..............................................T...................................................  615
AL553095.3  752   ......................................................................................................................................................  603

Query       651   CTTCGCAGAGTGTTTCAGAAATACACCAAGTACAGTAAGCATGACATGAACAAAGTTCTGGACCTGGAGTTGAAAGGTGACATTGAGAAATGCCTCACAGCTATCGTGAAGTGCGCCACAAGCAAACCAGCTTTCTTTGCAGAGAAGCTT  800
BQ228162.1  340   ......................................................................................................................................................  489
AL574991.3  596   ......................................................................................................................................................  447
BM555842.1  299   ......................................................................................................................................................  448
AL576603.2  603   ......................................................................................................................................................  454
AL576223.3  596   ......................................................................................................................................................  447
AL570428.3  601   ..........................................A...........................................................................................................  452
AL570884.3  603   ......................................................................................................................................................  454
AL576340.3  607   ......................................................................................................................................................  458
AL575425.3  614   ..........................................N....................................................N......................................................  465
AL553095.3  602   ....................................................................................................................................YY...V...........C  453

Query       801   CATCAAGCCATGAAAGGTGTTGGAACTCGCCATAAGGCATTGATCAGGATTATGGTTTCCCGTTCTGAAATTGACATGAATGATATCAAAGCATTCTATCAGAAGATGTATGGTATCTCCCTTTGCCAAGCCATCCTGGATGAAACCAAA  950
BQ228162.1  490   ......................................................................................................................................................  639
AL574991.3  446   ....................................................................................................................................W.................  297
BM555842.1  449   ......................................................................................................................................................  598
AL576603.2  453   ......................................................................................................................................................  304
AL576223.3  446   ......................................................................................................................................................  297
AL570428.3  451   ........................................................................A.........................................T...................................  302
AL570884.3  453   ......................................................................................................................................................  304
AL576340.3  457   ................................................................................................................................................R.....  308
AL575425.3  464   ......................................................................................................................................................  315
```

```
AL553095.3  452   .......................V......................................S..S..................RK...R...G...R..........................................K...G.....  303

Query       951   GGAGATTATGAGAAAATCCTGGTGGCTCTTTGTGGAGGAAACTAAACATTCCCTTGATGGTCTCAAGCTATGATCAGAAGACTTTAATTATATATATTTTCATCCTATAAGCTTAAATAGGAAAGTTTCTTCAACAGGATTACAGTGTAGCT  1100
BQ228162.1  640   ......................................................................................................................................................  790
                                                                                                                                         \
                                                                                                                                         |
                                                                                                                                         A
AL574991.3  296   .............................................................Y........................................................................................  147
BM555842.1  599   ......................................................................................................................................................  748
AL576603.2  303   ......................................................................................................................................................  154
AL576223.3  296   ......................................................................................................................................................  147
AL570428.3  301   ......................................................................................................................................................  152
AL570884.3  303   ......................................................................................................................................................  154
AL576340.3  307   ..R...................................................................................................................................................  158
AL575425.3  314   ......................................................................................................................................................  165
AL553095.3  302   ..R...................................................................................................................................................  153

Query       1101  ACCTACATGCTGAAAAATATAGCCTTTAAATCATTTTTATATTATAACTCTGTATAATAGAGATAAGTCCATTTTTTAAAAATGTTTTCCCCAAACCATAAAACCCTATACAAGTTGTTCTAGTAACAATACATGAGAAAGATGTCTATG  1250
BQ228162.1  791   ...........................................................................................................................G..........................  942
                                                                                                                                   \                      \
                                                                                                                                   |                      |
                                                                                                                                   G                      G
AL574991.3  146   ..................................................................................................................................................      1
BM555842.1  749   .....................................................................................................................G................................  907
                                                                                                    \     \              \              \        \ \          \     \  \
                                                                                                    |     |              |              |        | |          |     |  |
                                                                                                    A     C              C              N        C N          A     C  G
AL576603.2  153   ...............................................................................................................................A......................  3
                                                                                                                                             \
                                                                                                                                             |
                                                                                                                                             C
AL576223.3  146   .........................................................................................B........................................................      1
AL570428.3  151   .......................................................T..............................................................................................  2
AL570884.3  153   ...................................................................................RW...V..............................N...N...W.................A....  2
                                                                                                                                       \        \
                                                                                                                                       |        |
                                                                                                                                       N        N
AL576340.3  157   ......................................................................................................................................................  7
                                                                                                                                             \
                                                                                                                                             |
                                                                                                                                             N
AL575425.3  164   ...N...........................................................................................................................N..............G.....    17
AL553095.3  152   ....................................................................................................................................................G.  2
                                                                                                                                     \
                                                                                                                                     |
                                                                                                                                     N

Query       1251  TAGCTGAAAATAAAATGACGTC  1272
BQ228162.1  943   ......................  965
                        \
                        |
                        A
BM555842.1  908   ...............         922
AL576603.2  2     ..                      1
AL570428.3  1     .                       1
AL570884.3  1     .                       1
AL576340.3  6     ..                      5
AL553095.3  1     .                       1
```

Query= T14-11 [organism=Homo sapiens] anxa1

Length=1348

Sequences producing significant alignments:	Score (Bits)	E Value	Max ident
AL570428.3 AL570428 Homo sapiens PLACENTA COT 25-NORMALIZED Ho...	1908	0.0	98%
AL576223.3 AL576223 Homo sapiens PLACENTA COT 25-NORMALIZED Ho...	1831	0.0	98%
AL570884.3 AL570884 Homo sapiens PLACENTA COT 25-NORMALIZED Ho...	1825	0.0	97%
AL574991.3 AL574991 Homo sapiens PLACENTA COT 25-NORMALIZED Ho...	1810	0.0	98%
AL575425.3 AL575425 Homo sapiens PLACENTA COT 25-NORMALIZED Ho...	1788	0.0	98%
AL553095.3 AL553095 Homo sapiens PLACENTA COT 25-NORMALIZED Ho...	1762	0.0	97%
FS581262.1 FS581262 macaque heart cDNA library QhtB Macaca fas...	1742	0.0	96%
BQ228162.1 AGENCOURT_7258587 NIH_MGC_71 Homo sapiens cDNA clon...	1731	0.0	99%
AL576340.3 AL576340 Homo sapiens PLACENTA COT 25-NORMALIZED Ho...	1729	0.0	97%
AL576603.2 AL576603 Homo sapiens PLACENTA COT 25-NORMALIZED Ho...	1655	0.0	99%

ALIGNMENTS

```
Query       1     AGTGTGAAATCTTCAGAGAAGAATTTCTCTTTAGTTCTTTGCAAGAAGGTAGAGATATAAGACACTTTTTCAAAAATGGCAATGGTATCAGAATTCCTCAAGCAGGCCTGGTTTATTGAAAATGAAGAGCAGGAATATGTTCAAACTGTG  150
FS581262.1  1     ......-...................C.C.........................-...............................................................................................  148
```

```
Query       151   AAGTCATCCAAAGGTGGTCCCGGATCAGCGGTGAGCCCCTATCCTACCTTCAAATCCATCATTTGAACATTGATCCTAACTAACTAAGCGAAACAATGCACAGCGTCAACAGATCAAAGCAGCATATCTCCAGGAAACAGGAAAGCCCCT  300
AL570428.3  1087                                                                                 ....-.T............-.....T....-.A..-..........W.......-.........GC.....  1022
AL576223.3  1050                                                                                                            ......-W......-R...-.Y....-........-........  1012
AL570884.3  1059                                                                                                                      .........T.....T............C...Y.  1025
                                                                                                                                         \
                                                                                                                                         |
                                                                                                                                         M
AL574991.3  1017                                                                                                                                                 .......  1011
AL575425.3  1037                                                                                                                                                    ....  1034
AL553095.3  1020                                                                                                                                                    ....  1017
FS581262.1  149   ..A.................T........A.......................-......CAACC.T......-.A.-T.......................................................................  295
AL576340.3  1011                                                                                                                                                    ....  1008

Query       301   GGATGAAACACTGAAGAAAGCCCTTACAGGTCACCTTGAGGAGGTTGTTTTAGCTCTGCTAAAAACTCCAGCGCAATTTGATGCTGATGAACTTCGTGCTGCCATGAAGGGCCTTGGAACTGATGAAGATACTCTAATTGAGATTTTGGC  450
AL570428.3  1021  ......................-Y.............................................................................................M................................  873
AL576223.3  1011  -.R...................-.....-.D.....WR............-.A....-V..................................-........................................W..AAR..........  868
AL570884.3  1024  ..W...............RS....W.....Y.M.....R...T...K......YY..S.W......C.............................K...S.................................................  875
AL574991.3  1010  -...-R.....-K..-..RS..-...CR...............-...K.............................................-........................................................  868
AL575425.3  1033  ....-..........-........M.......M...................KG.........C.............................C..C...S....A............................................  886
AL553095.3  1016  ....-R....-YK.......-.......-..............-...-.....-................................................................................................  874
FS581262.1  296   .......................C..TG....................................................................G................................C.....G..............  445
BQ228162.1  12                                                                                               .........................................................  68
AL576340.3  1007  -....-......-...-...G..-....-....-.Y...-..-....-........-...-........-......-................-....-.K.......-....-.............-RW...................-  878
AL576603.2  917                                                                                                         .....-........-...........-WM..............-...  875

Query       451   ATCAAGAACTAACAAAGAAATCAGAGACATTAACAGGGTCTACAGAGAGGAACTGAAGAGAGATCTGGCCAAAGACATAACCTCAGACACATCTGGAGATTTTCGGAACGCTTTGCTTTCTCTTGCTAAGGGTGACCGATCTGAGGACTT  600
AL570428.3  872   ...............................W......................................................................................................................  723
AL576223.3  867   ......................................................................................................................................................  718
AL570884.3  874   ......................................................................................................................................................  725
AL574991.3  867   ......................................................................................................................................................  718
AL575425.3  885   .........................R........................R..............................................................................................R....  736
AL553095.3  873   ......................................................................................................................................................  724
FS581262.1  446   ......................................................................................................................................................  595
BQ228162.1  69    ......................................................................................................................................................  218
AL576340.3  877   ......-...............................................................................................................................................  729
AL576603.2  874   ......................................................................................................................................................  725

Query       601   TGGTGTGAATGAAGACTTGGCTGATTCAGATGCCAGGGCCTTGTATGAAGCAGGAGAAAGGAGAAAGGGGACAGACGTAAACGTGTTCAATACCATCCTTACCACCAGAAGCTATCCACAACTTCGCAGAGTGTTTCAGAAATACACCAA  750
AL570428.3  722   .............R.......................................................N................................................................................  573
AL576223.3  717   ......................................................................................................................................................  568
AL570884.3  724   ......................................................................................................................................................  575
AL574991.3  717   ......................................................................................................................................................  568
AL575425.3  735   .............B........S..............................................T................................................................................  586
AL553095.3  723   ......................................................................................................................................................  574
FS581262.1  596   ...................................................G.......................T..............................C.............T.............................  745
BQ228162.1  219   ......................................................................................................................................................  368
AL576340.3  728   ......................................................................................................................................................  579
AL576603.2  724   ......................................................................................................................................................  575

Query       751   GTACAGTAAGCATGACATGAACAAAGTTCTGGACCTGGAGTTGAAAGGTGACATTGAGAAATGCCTCACAGCTATCGTGAAGTGCGCCACAAGCAAACCAGCTTTCTTTGCAGAGAAGCTTCATCAAGCCATGAAAGGTGTTGGAACTCG  900
AL570428.3  572   .............A........................................................................................................................................  423
AL576223.3  567   ......................................................................................................................................................  418
AL570884.3  574   ......................................................................................................................................................  425
AL574991.3  567   ......................................................................................................................................................  418
AL575425.3  585   .............N....................................................N...................................................................................  436
AL553095.3  573   .......................................................................................................YY...V...........C.......................V.....  424
FS581262.1  746   ....................................................................................T................................A........G.............C.........  895
BQ228162.1  369   ......................................................................................................................................................  518
AL576340.3  578   ......................................................................................................................................................  429
AL576603.2  574   ......................................................................................................................................................  425

Query       901   CCATAAGGCATTGATCAGGATTATGGTTTCCCGTTCTGAAATTGACATGAATGATATCAAAGCATTCTATCAGAAGATGTATGGTATCTCCCTTTGCCAAGCCATCCTGGATGAAACCAAAGGAGATTATGAGAAAATCCTGGTGGCTCT  1050
AL570428.3  422   ...........................................A.........................................T................................................................  273
AL576223.3  417   ......................................................................................................................................................  268
AL570884.3  424   ......................................................................................................................................................  275
AL574991.3  417   .......................................................................................................W..............................................  268
AL575425.3  435   ......................................................................................................................................................  286
AL553095.3  423   .................................S..S..................RK...R...G...R..........................................K...G.......R..........................  274
FS581262.1  896   ..............................A...........................-..............-.........................................-....-......-....-........-........  1038
BQ228162.1  519   ......................................................................................................................................................  668
AL576340.3  428   ...................................................................................................................R.......R..........................  279
AL576603.2  424   ......................................................................................................................................................  275

Query       1051  TTGTGGAGGAAACTAAACATTCCCTTGATGGTCTCAAGCTATGATCAGAAGACTTTAATTATATATTTTCATCCTATAAGCTTAAATAGGAAAGTTTCTTCAACAGGATTACAGTGTAGCTACCTACATGCTGAAAAATATAGCCTTTAA  1200
AL570428.3  272   ......................................................................................................................................................  123
AL576223.3  267   ......................................................................................................................................................  118
AL570884.3  274   ......................................................................................................................................................  125
AL574991.3  267   ................................Y.....................................................................................................................  118
AL575425.3  285   ............................................................................................................................N.........................  136
AL553095.3  273   ......................................................................................................................................................  124
FS581262.1  1039  .....-..-.......-........-....-.....-...                                                                                                                1072
```

```
BQ228162.1  669   ......................................................................................................................................................  819
                                                                                                                \
                                                                                                                |
                                                                                                                A
AL576340.3  278   ......................................................................................................................................................  129
AL576603.2  274   ......................................................................................................................................................  125

Query       1201  ATCATTTTTATATTATAACTCTGTATAATAGAGATAAGTCCATTTTTTAAAAATGTTTTCCCCAAACCATAAAACCCTATACAAGTTGTTCTAGTAACAATACATGAGAAAGATGTCTATGTAGCTGAAAATAAAATGACGTC  1343
AL570428.3  122   ..........................T...............................................................................................                       1
AL576223.3  117   ............................................................B........................................................                            1
AL570884.3  124   ......................................................RW....V.............................N....N....W...............A.....                       1
                                                                                                                  \          \
                                                                                                                  |          |
                                                                                                                  N          N
AL574991.3  117   .....................................................................................................................                            1
AL575425.3  135   ....................................................................................................N............G.....                          17
AL553095.3  123   .......................................................................................................................G..                       1
                                                                                                                \
                                                                                                                |
                                                                                                                N
BQ228162.1  820   ..............................................................................................G................................................  965
                                                                                                      \                      \                   \
                                                                                                      |                      |                   |
                                                                                                      G                      G                   A
AL576340.3  128   ...........................................................................................................................                      5
                                                                                                                      \
                                                                                                                      |
                                                                                                                      N
AL576603.2  124   ....................................................................................................A......................                      1
                                                                                                                     \
                                                                                                                     |
                                                                                                                     C

Query= T15-5 [organism=Homo sapiens] anxa1

Length=1206


                                                                  Score        E     Max
Sequences producing significant alignments:                      (Bits)     Value  ident

BQ228162.1 AGENCOURT_7258587 NIH_MGC_71 Homo sapiens cDNA clon...  1531       0.0    99%
AL576603.2 AL576603 Homo sapiens PLACENTA COT 25-NORMALIZED Ho...  1507       0.0    100%
AL576340.3 AL576340 Homo sapiens PLACENTA COT 25-NORMALIZED Ho...  1506       0.0    100%
AL576223.3 AL576223 Homo sapiens PLACENTA COT 25-NORMALIZED Ho...  1504       0.0    100%
AL574991.3 AL574991 Homo sapiens PLACENTA COT 25-NORMALIZED Ho...  1500       0.0    100%
BM555842.1 AGENCOURT_6544353 NIH_MGC_88 Homo sapiens cDNA clon...  1495       0.0    99%
AL570428.3 AL570428 Homo sapiens PLACENTA COT 25-NORMALIZED Ho...  1487       0.0    99%
BE614257.1 601504294T1 NIH_MGC_71 Homo sapiens cDNA clone IMAG...  1483       0.0    99%
AL570884.3 AL570884 Homo sapiens PLACENTA COT 25-NORMALIZED Ho...  1480       0.0    99%
BU857810.1 AGENCOURT_10474308 NIH_MGC_107 Homo sapiens cDNA cl...  1478       0.0    100%

ALIGNMENTS

Query       360   ACTGAAGAGAGATCTGGCCAAAGACATAACCTCAGACACATCTGGAGATTTTCGGAACGCTTTGCTTTCTCTTGCTAAGGGTGACCGATCTGAGGACTTTGGTGTGAATGAAGACTTGGCTGATTCAGATGCCAGGGCCTTGTATGAAGC  509
BQ228162.1  120   ......................................................................................................................................................  269
AL576603.2  823   ......................................................................................................................................................  674
AL576340.3  827   ......................................................................................................................................................  678
AL576223.3  816   ......................................................................................................................................................  667
AL574991.3  816   ......................................................................................................................................................  667
BM555842.1  79    ......................................................................................................................................................  228
AL570428.3  821   ................................................................................................................R.....................................  672
BE614257.1  836    .........A.................C...........................................-.............................................................................  687
                      \                                        \
                      |                                        |
                      A                                        G
AL570884.3  823   ......................................................................................................................................................  674
BU857810.1  1                                       ....................................................................................................................  116

Query       510   AGGAGAAAGGAGAAAGGGGACAGACGTAAACGTGTTCAATACCATCCTTACCACCAGAAGCTATCCACAACTTCGCAGAGTGTTTCAGAAATACACCAAGTACAGTAAGCATGACATGAACAAAGTTCTGGACCTGGAGTTGAAAGGTGA  659
BQ228162.1  270   ......................................................................................................................................................  419
AL576603.2  673   ......................................................................................................................................................  524
AL576340.3  677   ......................................................................................................................................................  528
AL576223.3  666   ......................................................................................................................................................  517
AL574991.3  666   ......................................................................................................................................................  517
BM555842.1  229   ......................................................................................................................................................  378
AL570428.3  671   ..................N.............................................................................................A.....................................  522
BE614257.1  686   ......................................................................................................................................................  537
AL570884.3  673   ......................................................................................................................................................  524
BU857810.1  117   ......................................................................................................................................................  266

Query       660   CATTGAGAAATGCCTCACAGCTATCGTGAAGTGCGCCACAAGCAAACCAGCTTTCTTTGCAGAGAAGCTTCATCAAGCCATGAAAGGTGTTGGAACTCGCCATAAGGCATTGATCAGGATTATGGTTTCCCGTTCTGAAATTGACATGAA  809
```

```
BQ228162.1  420   ......................................................................................................................................................  569
AL576603.2  523   ......................................................................................................................................................  374
AL576340.3  527   ......................................................................................................................................................  378
AL576223.3  516   ......................................................................................................................................................  367
AL574991.3  516   ......................................................................................................................................................  367
BM555842.1  379   ......................................................................................................................................................  528
AL570428.3  521   ..............................................................................................................................................A.......  372
BE614257.1  536   ......................................................................................................................................................  387
AL570884.3  523   ......................................................................................................................................................  374
BU857810.1  267   ......................................................................................................................................................  416

Query       810   TGATATCAAAGCATTCTATCAGAAGATGTATGGTATCTCCCTTTGCCAAGCCATCCTGGATGAAACCAAAGGAGATTATGAGAAAATCCTGGTGGCTCTTTGTGGAGGAAACTAAACATTCCCTTGATGGTCTCAAGCTATGATCAGAAG  959
BQ228162.1  570   ......................................................................................................................................................  719
AL576603.2  373   ......................................................................................................................................................  224
AL576340.3  377   ................................................................R.......R.............................................................................  228
AL576223.3  366   ......................................................................................................................................................  217
AL574991.3  366   ....................................................W..............................................................................Y..................  217
BM555842.1  529   ......................................................................................................................................................  678
AL570428.3  371   ..................................T...................................................................................................................  222
BE614257.1  386   ......................................................................................................................................................  237
AL570884.3  373   ......................................................................................................................................................  224
BU857810.1  417   ......................................................................................................................................................  566

Query       960   ACTTTAATTATATATTTTCATCCTATAAGCTTAAATAGGAAAGTTTCTTCAACAGGATTACAGTGTAGCTACCTACATGCTGAAAAATATAGCCTTTAAATCATTTTTATATTATAACTCTGTATAATAGAGATAAGTCCATTTTTTAAA  1109
BQ228162.1  720   ......................................................................................................................................................  870
                                                         \
                                                         |
                                                         A
AL576603.2  223   ......................................................................................................................................................  74
AL576340.3  227   ......................................................................................................................................................  78
AL576223.3  216   ......................................................................................................................................................  67
AL574991.3  216   ......................................................................................................................................................  67
BM555842.1  679   ......................................................................................................................................................  828
AL570428.3  221   .............................................................................................................................T........................  72
BE614257.1  236   ......................................................................................................................................................  87
AL570884.3  223   ......................................................................................................................................................  74
BU857810.1  567   .................................N................................................................N.................................................N.  716

Query       1110  AATGTTTTCCCCAAACCATAAAACCCTATACAAGTTGTTCTAGTAACAATACATGAGAAAGATGTCTATGTAGCTGAAAATAAAATGACGTC  1201
BQ228162.1  871   ...........................................G................................................  965
                                                   \                      \                   \
                                                   |                      |                   |
                                                   G                      G                   A
AL576603.2  73    .................................................A......................                      1
                                                  \
                                                  |
                                                  C
AL576340.3  77    .........................................................................                     5
                                                   \
                                                   |
                                                   N
AL576223.3  66    .........B........................................................                            1
AL574991.3  66    ..................................................................                            1
BM555842.1  829   .....................................G...............................................         922
                    \     \              \              \        \ \          \     \  \
                    |     |              |              |        | |          |     |  |
                    A     C              C              N        C N          A     C  G
AL570428.3  71    .......................................................................                       1
BE614257.1  86    ............................................................T.A-.................             6
                                                                                              \
                                                                                              |
                                                                                              T
AL570884.3  73    ...RW....V.............................N....N....W...............A.....                       1
                                                           \          \
                                                           |          |
                                                           N          N
BU857810.1  717   ...............................................N............................................  808
```

Query= T16-12 [organism=Homo sapiens] anxa1

Length=1094

Sequences producing significant alignments:	Score (Bits)	E Value	Max ident
AL553114.3 AL553114 Homo sapiens PLACENTA COT 25-NORMALIZED Ho...	1441	0.0	100%
CX870542.1 HESC4_46_F03.g1_A037 NIH_MGC_262 Homo sapiens cDNA ...	1441	0.0	100%
BQ953713.1 AGENCOURT_8786826 NIH_MGC_43 Homo sapiens cDNA clon...	1432	0.0	100%
BM458053.1 AGENCOURT_6411336 NIH_MGC_71 Homo sapiens cDNA clon...	1430	0.0	100%
AL544959.3 AL544959 Homo sapiens PLACENTA COT 25-NORMALIZED Ho...	1426	0.0	100%
AU117349.1 AU117349 HEMBA1 Homo sapiens cDNA clone HEMBA100120...	1426	0.0	100%

```
AL553679.3 AL553679 Homo sapiens PLACENTA COT 25-NORMALIZED Ho...  1419       0.0    100%
BQ962441.1 AGENCOURT_10056322 NIH_MGC_71 Homo sapiens cDNA clo...  1419       0.0    100%
BU902298.1 AGENCOURT_10127317 NIH_MGC_71 Homo sapiens cDNA clo...  1417       0.0    100%
EB387180.1 nbj26d10.y1 Human optic nerve. Unnormalized (nbj) H...  1415       0.0    100%

ALIGNMENTS

Query       1    AGTGTGAAATCTTCAGAGAAGAATTTCTCTTTAGTTCTTTGCAAGAAGGTAGAGATAAAGACACTTTTTCAAAAATGGCAATGGTATCAGAATTCCTCAAGCAGGCCTGGTTTATTGAAAATGAAGAGCAGGAATATGTTCAAACTGTGA  150
AL553114.3  107  ......................................................................................................................................................  256
CX870542.1  29   ......................................................................................................................................................  178
BQ953713.1  1         .................................................................................................................................................  145
BM458053.1  11   ......................................................................................................................................................  160
AL544959.3  1            ..............................................................................................................................................  142
AU117349.1  1    ......................................................................................................................................................  150
AL553679.3  1            ..............................................................................................................................................  142
BQ962441.1  1                ..........................................................................................................................................  138
BU902298.1  24            .............................................................................................................................................  164
                                                                                \
                                                                                |
                                                                                N
EB387180.1  1                  ........................................................................................................................................  136

Query       151  AGTCATCCAAAGGTGGTCCCGGATCAGCGGTGAGCCCCTATCCTACCTTCAATCCATCCTCGGATGTCGCTGCCTTGCATAAGGCCATAATGGTTAAAGGTGTGGATGAAGCAACCATCATTGACATTCTAACTAAGCGAAACAATGCAC  300
AL553114.3  257  ......................................................................................................................................................  406
CX870542.1  179  ......................................................................................................................................................  328
BQ953713.1  146  ......................................................................................................................................................  295
BM458053.1  161  ......................................................................................................................................................  310
AL544959.3  143  ......................................................................................................................................................  292
AU117349.1  151  ......................................................................................................................................................  300
AL553679.3  143  ......................................................................................................................................................  292
BQ962441.1  139  ......................................................................................................................................................  288
BU902298.1  165  ......................................................................................................................................................  314
EB387180.1  137  ......................................................................................................................................................  286

Query       301  AGCGTCAACAGATCAAAGCAGCATATCTCCAGGAAACAGGAAAGCCCCTGGATGAAACACTGAAGAAAGCCCTTACAGGTCACCTTGAGGAGGTTGTTTTAGCTCTGCTAAAAACTCCAGCGCAATTTGATGCTGATGAACTTCGTGCTG  450
AL553114.3  407  ......................................................................................................................................................  556
CX870542.1  329  ......................................................................................................................................................  478
BQ953713.1  296  ......................................................................................................................................................  445
BM458053.1  311  ......................................................................................................................................................  460
AL544959.3  293  ......................................................................................................................................................  442
AU117349.1  301  ........................................................................C.............................................................................  450
AL553679.3  293  .....................................................................-................................................................................  441
BQ962441.1  289  ......................................................................................................................................................  438
BU902298.1  315  ......................................................................................................................................................  464
EB387180.1  287  ......................................................................................................................................................  436

Query       451  CCATGAAGGGCCTTGGAACTGATGAAGATACTCTAATTGAGATTTTGGCATCAAGAACTAACAAAGAAATCAGAGACATTAACAGGGTCTACAGAGAGGAACTGAAGAGAGATCTGGCCAAAGACATAACCTCAGACACATCTGGAGATT  600
AL553114.3  557  ......................................................................................................................................................  706
CX870542.1  479  ......................................................................................................................................................  628
BQ953713.1  446  ......................................................................................................................................................  595
BM458053.1  461  ......................................................................................................................................................  610
AL544959.3  443  ......................................................................................................................................................  592
AU117349.1  451  ......................................................................................................................................................  600
AL553679.3  442  ......................................................................................................................................................  591
BQ962441.1  439  ......................................................................................................................................................  588
BU902298.1  465  ......................................................................................................................................................  614
EB387180.1  437  ......................................................................................................................................................  586

Query       601  TTCGGAACGCTTTGCTTTCTCTTGCTAAGGGTGACCGATCTGAGGACTTTGGTGTGAATGAAGACTTGGCTGATTCAGATGCCAGGGCCTTGTATGAAGCAGGAGAAAGGAGAAAGGGGACAGACGTAAACGTGTTCAATACCATCCTTA  750
AL553114.3  707  ......................................................................................................................................................  856
CX870542.1  629  ......................................................................................................................................................  778
BQ953713.1  596  ......................................................................................................................................................  745
BM458053.1  611  ......................................................................................T...............................................................  760
AL544959.3  593  ......................................................................................................................................................  742
AU117349.1  601  .....................................................................................................N................................................  750
AL553679.3  592  ......................................................................................................................................................  741
BQ962441.1  589  ......................................................................................................................................................  738
BU902298.1  615  ......................................................................................................................................................  764
EB387180.1  587  ......................................................................................................................................................  736

Query       751  CCACCAGAAGCTATCCACAACTTCGCAGAG  780
AL553114.3  857  ..............................  886
CX870542.1  779  ..............................  808
BQ953713.1  746  ..............................  775
BM458053.1  761  ..............................  791
                                      \
                                      |
                                      C
AL544959.3  743  ..............................  772
AU117349.1  751  ...........................     777
AL553679.3  742  ..............................  771
BQ962441.1  739  ..............................  768
```

```
BU902298.1  765  ..............................  794
EB387180.1  737  ..............................  766

Query= T17-2 [organism=Homo sapiens] anxa1

Length=1103


                                                                   Score        E     Max
Sequences producing significant alignments:                       (Bits)     Value  ident

BU857810.1 AGENCOURT_10474308 NIH_MGC_107 Homo sapiens cDNA cl...  1386       0.0    99%
CN386790.1 17000599887650 GRN_PRENEU Homo sapiens cDNA 5', mRN...  1378       0.0    100%
BQ228162.1 AGENCOURT_7258587 NIH_MGC_71 Homo sapiens cDNA clon...  1376       0.0    99%
CA447733.1 UI-H-EI0-ayf-a-16-0-UI.s1 NCI_CGAP_EI0 Homo sapiens...  1371       0.0    100%
CA423056.1 UI-H-FL0-bdk-f-10-0-UI.s1 NCI_CGAP_FL0 Homo sapiens...  1365       0.0    100%
BE614257.1 601504294T1 NIH_MGC_71 Homo sapiens cDNA clone IMAG...  1358       0.0    99%
BQ776006.1 UI-H-FH0-bck-h-18-0-UI.s1 NCI_CGAP_FH0 Homo sapiens...  1356       0.0    100%
AL576603.2 AL576603 Homo sapiens PLACENTA COT 25-NORMALIZED Ho...  1352       0.0    100%
AL576340.3 AL576340 Homo sapiens PLACENTA COT 25-NORMALIZED Ho...  1351       0.0    100%
AL576223.3 AL576223 Homo sapiens PLACENTA COT 25-NORMALIZED Ho...  1349       0.0    100%

ALIGNMENTS

Query       346   CCGATCTGAGGACTTTGGTGTGAATGAAGACTTGGCTGATTCAGATGCCAGGGCCTTGTATGAAGCAGGAGAAAGGAGAAAGGGGACAGACGTAAACGTGTTCAATACCATCCTTACCACCAGAAGCTATCCACAACTTCGCAGAGTGTT  495
BU857810.1  51    ......................................................................................................................................................  200
CN386790.1  18    ......................................................................................................................................................  167
BQ228162.1  204   ......................................................................................................................................................  353
CA447733.1  765                   ......................................................................................................................................  632
CA423056.1  767                   ......................................................................................................................................  634
BE614257.1  752   ......................................................................................................................................................  603
BQ776006.1  765                   ..................-..................................................................................................................N  632
                                                         \
                                                         |
                                                         T
AL576603.2  739   ......................................................................................................................................................  590
AL576340.3  743   ......................................................................................................................................................  594
AL576223.3  732   ......................................................................................................................................................  583

Query       496   TCAGAAATACACCAAGTACAGTAAGCATGACATGAACAAAGTTCTGGACCTGGAGTTGAAAGGTGACATTGAGAAATGCCTCACAGCTATCGTGAAGTGCGCCACAAGCAAACCAGCTTTCTTTGCAGAGAAGCTTCATCAAGCCATGAA  645
BU857810.1  201   ......................................................................................................................................................  350
CN386790.1  168   ......................................................................................................................................................  317
BQ228162.1  354   ......................................................................................................................................................  503
CA447733.1  631   ......................................................................................................................................................  482
CA423056.1  633   ......................................................................................................................................................  484
BE614257.1  602   ......................................................................................................................................................  453
BQ776006.1  631   ......................................................................................................................................................  482
AL576603.2  589   ......................................................................................................................................................  440
AL576340.3  593   ......................................................................................................................................................  444
AL576223.3  582   ......................................................................................................................................................  433

Query       646   AGGTGTTGGAACTCGCCATAAGGCATTGATCAGGATTATGGTTTCCCGTTCTGAAATTGACATGAATGATATCAAAGCATTCTATCAGAAGATGTATGGTATCTCCCTTTGCCAAGCCATCCTGGATGAAACCAAAGGAGATTATGAGAA  795
BU857810.1  351   ......................................................................................................................................................  500
CN386790.1  318   ......................................................................................................................................................  467
BQ228162.1  504   ......................................................................................................................................................  653
CA447733.1  481   ......................................................................................................................................................  332
CA423056.1  483   ......................................................................................................................................................  334
BE614257.1  452   ......................................................................................................................................................  303
BQ776006.1  481   ......................................................................................................................................................  332
AL576603.2  439   ......................................................................................................................................................  290
AL576340.3  443   ..................................................................................................................................R.......R...........  294
AL576223.3  432   ......................................................................................................................................................  283

Query       796   AATCCTGGTGGCTCTTTGTGGAGGAAACTAAACATTCCCTTGATGGTCTCAAGCTATGATCAGAAGACTTTAATTATATATTTTCATCCTATAAGCTTAAATAGGAAAGTTTCTTCAACAGGATTACAGTGTAGCTACCTACATGCTGAA  945
BU857810.1  501   ...................................................................................................N..................................................  650
CN386790.1  468   ......................................................................................................................................................  617
BQ228162.1  654   ......................................................................................................................................................  804
                                                                                                                           \
                                                                                                                           |
                                                                                                                           A
CA447733.1  331   ......................................................................................................................................................  182
CA423056.1  333   ......................................................................................................................................................  184
BE614257.1  302   ......................................................................................................................................................  153
BQ776006.1  331   ......................................................................................................................................................  182
AL576603.2  289   ......................................................................................................................................................  140
AL576340.3  293   ......................................................................................................................................................  144
AL576223.3  282   ......................................................................................................................................................  133

Query       946   AAATATAGCCTTTAAATCATTTTTATATTATAACTCTGTATAATAGAGATAAGTCCATTTTTTAAAAATGTTTTCCCCAAACCATAAAACCCTATACAAGTTGTTCTAGTAACAATACATGAGAAAGATGTCTATGTAGCTGAAAATAAA  1095
BU857810.1  651   ..............N.................................................N................................................N....................................  800
CN386790.1  618   ......................................................................G..............................................................................   766
BQ228162.1  805   .............................................................................................................G........................................  957
```

```
                                                                                                                     \                      \                   \
                                                                                                                     |                      |                   |
                                                                                                                     G                      G                   A
CA447733.1  181   ......................................................................................................................................................  32
CA423056.1  183   .................................................................................................................................................G....  34
BE614257.1  152   ..............................................................................................................................T.A-.................     6
                                                                                                                                                                  \
                                                                                                                                                                  |
                                                                                                                                                                  T
BQ776006.1  181   ......................................................................................................................................................  32
AL576603.2  139   ...................................................................................................................A......................              1
                                                                                                                                    \
                                                                                                                                    |
                                                                                                                                    C
AL576340.3  143   ..........................................................................................................................................              5
                                                                                                                                     \
                                                                                                                                     |
                                                                                                                                     N
AL576223.3  132   ...........................................................................B........................................................                    1

Query       1096  ATGACGTC  1103
BU857810.1  801   ........  808
BQ228162.1  958   ........  965
CA447733.1  31    ........  24
CA423056.1  33    ........  26
BQ776006.1  31    ........  24

Query= T18-19 [organism=Homo sapiens] anxa1

Length=1170


                                                                  Score        E     Max
Sequences producing significant alignments:                      (Bits)     Value  ident

CA447733.1 UI-H-EI0-ayf-a-16-0-UI.s1 NCI_CGAP_EI0 Homo sapiens...  1319       0.0    100%
CB306442.1 UI-CF-EN1-aej-e-15-0-UI.s1 UI-CF-EN1 Homo sapiens c...  1315       0.0    100%
CA418062.1 UI-H-FH0-bcc-j-24-0-UI.s1 NCI_CGAP_FH0 Homo sapiens...  1315       0.0    100%
BQ776006.1 UI-H-FH0-bck-h-18-0-UI.s1 NCI_CGAP_FH0 Homo sapiens...  1315       0.0    100%
CA423056.1 UI-H-FL0-bdk-f-10-0-UI.s1 NCI_CGAP_FL0 Homo sapiens...  1314       0.0    100%
CA418210.1 UI-H-FH0-bcf-i-22-0-UI.s1 NCI_CGAP_FH0 Homo sapiens...  1312       0.0    100%
CA443862.1 UI-H-DP0-avg-a-20-0-UI.s1 NCI_CGAP_Fs1 Homo sapiens...  1310       0.0    100%
BQ774641.1 UI-H-FH0-bcc-l-13-0-UI.s1 NCI_CGAP_FH0 Homo sapiens...  1308       0.0    100%
CA447223.1 UI-H-EI0-ayd-b-19-0-UI.s1 NCI_CGAP_EI0 Homo sapiens...  1303       0.0    100%
BQ775843.1 UI-H-FH0-bcg-e-23-0-UI.s1 NCI_CGAP_FH0 Homo sapiens...  1301       0.0    100%

ALIGNMENTS

Query       457   AGGGCCTTGTATGAAGCAGGAGAAAGGAGAAAGGGGACAGACGTAAACGTGTTCAATACCATCCTTACCACCAGAAGCTATCCACAACTTCGCAGAGTGTTTCAGAAATACACCAAGTACAGTAAGCATGACATGAACAAAGTTCTGGAC  606
CA447733.1  732   ......................................................................................................................................................  583
CB306442.1  732   ...N..................................................................................................................................................  583
CA418062.1  732   ...................................N..................................................................................................................  583
BQ776006.1  732   ....................................................................................................N.................................................  583
CA423056.1  734   ......................................................................................................................................................  585
CA418210.1  728   ...................................-..................................................................................................................  580
CA443862.1  732   ......C...........................N...................................................................................................................  583
BQ774641.1  729     .................................-..................................................................................................................  583
CA447223.1  723            .............................................................................................................................................  583
BQ775843.1  721           ..........................N...................................................................................................................  580

Query       607   CTGGAGTTGAAAGGTGACATTGAGAAATGCCTCACAGCTATCGTGAAGTGCGCCACAAGCAAACCAGCTTTCTTTGCAGAGAAGCTTCATCAAGCCATGAAAGGTGTTGGAACTCGCCATAAGGCATTGATCAGGATTATGGTTTCCCGT  756
CA447733.1  582   ......................................................................................................................................................  433
CB306442.1  582   ......................................................................................................................................................  433
CA418062.1  582   ......................................................................................................................................................  433
BQ776006.1  582   ......................................................................................................................................................  433
CA423056.1  584   ......................................................................................................................................................  435
CA418210.1  579   ......................................................................................................................................................  430
CA443862.1  582   ......................................................................................................................................................  433
BQ774641.1  582   ......................................................................................................................................................  433
CA447223.1  582   ......................................................................................................................................................  433
BQ775843.1  579   ......................................................................................................................................................  430

Query       757   TCTGAAATTGACATGAATGATATCAAAGCATTCTATCAGAAGATGTATGGTATCTCCCTTTGCCAAGCCATCCTGGATGAAACCAAAGGAGATTATGAGAAAATCCTGGTGGCTCTTTGTGGAGGAAACTAAACATTCCCTTGATGGTCT  906
CA447733.1  432   ......................................................................................................................................................  283
CB306442.1  432   ......................................................................................................................................................  283
CA418062.1  432   ......................................................................................................................................................  283
BQ776006.1  432   ......................................................................................................................................................  283
CA423056.1  434   ......................................................................................................................................................  285
CA418210.1  429   ......................................................................................................................................................  280
CA443862.1  432   ......................................................................................................................................................  283
BQ774641.1  432   ......................................................................................................................................................  283
CA447223.1  432   ......................................................................................................................................................  283
```

```
BQ775843.1  429   ......................................................................................................................................................  280

Query       907   CAAGCTATGATCAGAAGACTTTAATTATATATTTTCATCCTATAAGCTTAAATAGGAAAGTTTCTTCAACAGGATTACAGTGTAGCTACCTACATGCTGAAAAATATAGCCTTTAAATCATTTTTATATTATAACTCTGTATAATAGAGA  1056
CA447733.1  282   ......................................................................................................................................................  133
CB306442.1  282   ......................................................................................................................................................  133
CA418062.1  282   ......................................................................................................................................................  133
BQ776006.1  282   ......................................................................................................................................................  133
CA423056.1  284   ......................................................................................................................................................  135
CA418210.1  279   ......................................................................................................................................................  130
CA443862.1  282   ......................................................................................................................................................  133
BQ774641.1  282   ......................................................................................................................................................  133
CA447223.1  282   ......................................................................................................................................................  133
BQ775843.1  279   ......................................................................................................................................................  130

Query       1057  TAAGTCCATTTTTTAAAAATGTTTTCCCCAAACCATAAAACCCTATACAAGTTGTTCTAGTAACAATACATGAGAAAGATGTCTATGTAGCTGAAAATAAAATGACGTCACAAG  1170
CA447733.1  132   ..................................................................................................................  19
CB306442.1  132   ..................................................................................................................  19
CA418062.1  132   ..................................................................................................................  19
BQ776006.1  132   ..................................................................................................................  19
CA423056.1  134   ................................................................................................G.................  21
CA418210.1  129   ..................................................................................................................  16
CA443862.1  132   ..................................................................................................................  19
BQ774641.1  132   ..................................................................................................................  19
CA447223.1  132   ..................................................................................................................  19
BQ775843.1  129   ..................................................................................................................  16
```

Query= T19-6 [organism=Homo sapiens] anxa1

Length=1210

```
                                                                   Score        E     Max
Sequences producing significant alignments:                       (Bits)     Value  ident

CA447733.1 UI-H-EI0-ayf-a-16-0-UI.s1 NCI_CGAP_EI0 Homo sapiens...  1360       0.0    100%
CA423056.1 UI-H-FL0-bdk-f-10-0-UI.s1 NCI_CGAP_FL0 Homo sapiens...  1354       0.0    100%
BQ776006.1 UI-H-FH0-bck-h-18-0-UI.s1 NCI_CGAP_FH0 Homo sapiens...  1345       0.0    99%
BU857810.1 AGENCOURT_10474308 NIH_MGC_107 Homo sapiens cDNA cl...  1336       0.0    99%
CB306442.1 UI-CF-EN1-aej-e-15-0-UI.s1 UI-CF-EN1 Homo sapiens c...  1330       0.0    100%
CA418062.1 UI-H-FH0-bcc-j-24-0-UI.s1 NCI_CGAP_FH0 Homo sapiens...  1330       0.0    100%
BM981135.1 UI-CF-EN1-adg-a-11-0-UI.s1 UI-CF-EN1 Homo sapiens c...  1330       0.0    99%
CN386790.1 17000599887650 GRN_PRENEU Homo sapiens cDNA 5', mRN...  1328       0.0    100%
CA418210.1 UI-H-FH0-bcf-i-22-0-UI.s1 NCI_CGAP_FH0 Homo sapiens...  1328       0.0    100%
CA443862.1 UI-H-DP0-avg-a-20-0-UI.s1 NCI_CGAP_Fs1 Homo sapiens...  1327       0.0    99%
```

ALIGNMENTS

```
Query       472   ATGAAGACTTGGCTGATTCAGATGCCAGGGCCTTGTATGAAGCAGGAGAAAGGAGAAAGGGGACAGACGTAAACGTGTTCAATACCATCCTTACCACCAGAAGCTATCCACAACTTCGCAGAGTGTTTCAGAAATACACCAAGTACAGTA  621
CA447733.1  758   ......................................................................................................................................................  609
CA423056.1  760   ......................................................................................................................................................  611
BQ776006.1  758   ..........-...............................................................................................................N.......................  609
                                  \
                                  |
                                  T
BU857810.1  74    ......................................................................................................................................................  223
CB306442.1  744                 ...............N........................................................................................................................  609
CA418062.1  744                 ...............................................N........................................................................................  609
BM981135.1  754   .......-.....................-..N............................-........................................-...............................................  609
CN386790.1  41    ......................................................................................................................................................  190
CA418210.1  740                 ...............................................-........................................................................................  606
CA443862.1  748              .....................C...........................N.........................................................................................  609
                                  \
                                  |
                                  T

Query       622   AGCATGACATGAACAAAGTTCTGGACCTGGAGTTGAAAGGTGACATTGAGAAATGCCTCACAGCTATCGTGAAGTGCGCCACAAGCAAACCAGCTTTCTTTGCAGAGAAGCTTCATCAAGCCATGAAAGGTGTTGGAACTCGCCATAAGG  771
CA447733.1  608   ......................................................................................................................................................  459
CA423056.1  610   ......................................................................................................................................................  461
BQ776006.1  608   ......................................................................................................................................................  459
BU857810.1  224   ......................................................................................................................................................  373
CB306442.1  608   ......................................................................................................................................................  459
CA418062.1  608   ......................................................................................................................................................  459
BM981135.1  608   ......................................................................................................................................................  459
CN386790.1  191   ......................................................................................................................................................  340
CA418210.1  605   ......................................................................................................................................................  456
CA443862.1  608   ......................................................................................................................................................  459

Query       772   CATTGATCAGGATTATGGTTTCCCGTTCTGAAATTGACATGAATGATATCAAAGCATTCTATCAGAAGATGTATGGTATCTCCCTTTGCCAAGCCATCCTGGATGAAACCAAAGGAGATTATGAGAAAATCCTGGTGGCTCTTTGTGGAG  921
CA447733.1  458   ......................................................................................................................................................  309
CA423056.1  460   ......................................................................................................................................................  311
BQ776006.1  458   ......................................................................................................................................................  309
BU857810.1  374   ......................................................................................................................................................  523
```

```
CB306442.1  458   ......................................................................................................................................................  309
CA418062.1  458   ......................................................................................................................................................  309
BM981135.1  458   ......................................................................................................................................................  309
CN386790.1  341   ......................................................................................................................................................  490
CA418210.1  455   ......................................................................................................................................................  306
CA443862.1  458   ......................................................................................................................................................  309

Query       922   GAAACTAAACATTCCCTTGATGGTCTCAAGCTATGATCAGAAGACTTTAATTATATATTTTCATCCTATAAGCTTAAATAGGAAAGTTTCTTCAACAGGATTACAGTGTAGCTACCTACATGCTGAAAAATATAGCTTTAAATCATTTTT  1071
CA447733.1  308   ......................................................................................................................................................  158
                                                                                                                                                          \
                                                                                                                                                          |
                                                                                                                                                          C
CA423056.1  310   ......................................................................................................................................................  160
                                                                                                                                                          \
                                                                                                                                                          |
                                                                                                                                                          C
BQ776006.1  308   ......................................................................................................................................................  158
                                                                                                                                                          \
                                                                                                                                                          |
                                                                                                                                                          C
BU857810.1  524   ............................................................................N...............................................................N.........  674
                                                                                                                                                         \
                                                                                                                                                         |
                                                                                                                                                         C
CB306442.1  308   ......................................................................................................................................................  158
                                                                                                                                                          \
                                                                                                                                                          |
                                                                                                                                                          C
CA418062.1  308   ......................................................................................................................................................  158
                                                                                                                                                          \
                                                                                                                                                          |
                                                                                                                                                          C
BM981135.1  308   ......................................................................................................................................................  158
                                                                                                                                                          \
                                                                                                                                                          |
                                                                                                                                                          C
CN386790.1  491   ......................................................................................................................................................  641
                                                                                                                                                         \
                                                                                                                                                         |
                                                                                                                                                         C
CA418210.1  305   ......................................................................................................................................................  155
                                                                                                                                                          \
                                                                                                                                                          |
                                                                                                                                                          C
CA443862.1  308   ......................................................................................................................................................  158
                                                                                                                                                          \
                                                                                                                                                          |
                                                                                                                                                          C

Query       1072  ATATTATAACTCTGTATAATAGAGATAAGTCCATTTTTTAAAAATGTTTTCCCCAAACCATAAAACCCTATACAAGTTGTTCTAGTAACAATACATGAGAAAGATGTCTATGTAGCTGAAAATAAAATGACGTCACAAG  1210
CA447733.1  157   ...........................................................................................................................................  19
CA423056.1  159   .........................................................................................................................G.................  21
BQ776006.1  157   ...........................................................................................................................................  19
BU857810.1  675   .........................................N................................................N...........................................       808
CB306442.1  157   ...........................................................................................................................................  19
CA418062.1  157   ...........................................................................................................................................  19
BM981135.1  157   ...........................................................................................................................................  19
CN386790.1  642   ...............................................G.............................................................................                766
CA418210.1  154   ...........................................................................................................................................  16
CA443862.1  157   ...........................................................................................................................................  19

Query= T21-11 [organism=Homo sapiens] anxa1

Length=989


                                                                    Score        E     Max
Sequences producing significant alignments:                        (Bits)     Value  ident

CA447733.1 UI-H-EI0-ayf-a-16-0-UI.s1 NCI_CGAP_EI0 Homo sapiens...  1369       0.0    100%
CA423056.1 UI-H-FL0-bdk-f-10-0-UI.s1 NCI_CGAP_FL0 Homo sapiens...  1363       0.0    100%
BU857810.1 AGENCOURT_10474308 NIH_MGC_107 Homo sapiens cDNA cl...  1354       0.0    99%
BQ776006.1 UI-H-FH0-bck-h-18-0-UI.s1 NCI_CGAP_FH0 Homo sapiens...  1354       0.0    100%
CN386790.1 17000599887650 GRN_PRENEU Homo sapiens cDNA 5', mRN...  1349       0.0    100%
BQ228162.1 AGENCOURT_7258587 NIH_MGC_71 Homo sapiens cDNA clon...  1345       0.0    99%
BM981135.1 UI-CF-EN1-adg-a-11-0-UI.s1 UI-CF-EN1 Homo sapiens c...  1338       0.0    99%
BE614257.1 601504294T1 NIH_MGC_71 Homo sapiens cDNA clone IMAG...  1328       0.0    99%
CB306442.1 UI-CF-EN1-aej-e-15-0-UI.s1 UI-CF-EN1 Homo sapiens c...  1327       0.0    100%
CA418062.1 UI-H-FH0-bcc-j-24-0-UI.s1 NCI_CGAP_FH0 Homo sapiens...  1327       0.0    100%

ALIGNMENTS
```

```
Query       249  GGTGTGAATGAAGACTTGGCTGATTCAGATGCCAGGGCCTTGTATGAAGCAGGAGAAAGGAGAAAGGGGACAGACGTAAACGTGTTCAATACCATCCTTACCACCAGAAGCTATCCACAACTTCGCAGAGTGTTTCAGAAATACACCAAG  398
CA447733.1  765  ......................................................................................................................................................  616
CA423056.1  767  ......................................................................................................................................................  618
BU857810.1  67   ......................................................................................................................................................  216
BQ776006.1  765  ..................-..................................................................................................................N................  616
                                        \
                                        |
                                        T
CN386790.1  34   ......................................................................................................................................................  183
BQ228162.1  220  ......................................................................................................................................................  369
BM981135.1  760   .............-.....................-..N............................-........................................-........................................  616
BE614257.1  736  ......................................................................................................................................................  587
CB306442.1  744                       ...............N.................................................................................................................  616
CA418062.1  744                       ...............................................N.................................................................................  616

Query       399  TACAGTAAGCATGACATGAACAAAGTTCTGGACCTGGAGTTGAAAGGTGACATTGAGAAATGCCTCACAGCTATCGTGAAGTGCGCCACAAGCAAACCAGCTTTCTTTGCAGAGAAGCTTCATCAAGCCATGAAAGGTGTTGGAACTCGC  548
CA447733.1  615  ......................................................................................................................................................  466
CA423056.1  617  ......................................................................................................................................................  468
BU857810.1  217  ......................................................................................................................................................  366
BQ776006.1  615  ......................................................................................................................................................  466
CN386790.1  184  ......................................................................................................................................................  333
BQ228162.1  370  ......................................................................................................................................................  519
BM981135.1  615  ......................................................................................................................................................  466
BE614257.1  586  ......................................................................................................................................................  437
CB306442.1  615  ......................................................................................................................................................  466
CA418062.1  615  ......................................................................................................................................................  466

Query       549  CATAAGGCATTGATCAGGATTATGGTTTCCCGTTCTGAAATTGACATGAATGATATCAAAGCATTCTATCAGAAGATGTATGGTATCTCCCTTTGCCAAGCCATCCTGGATGAAACCAAAGGAGATTATGAGAAAATCCTGGTGGCTCTT  698
CA447733.1  465  ......................................................................................................................................................  316
CA423056.1  467  ......................................................................................................................................................  318
BU857810.1  367  ......................................................................................................................................................  516
BQ776006.1  465  ......................................................................................................................................................  316
CN386790.1  334  ......................................................................................................................................................  483
BQ228162.1  520  ......................................................................................................................................................  669
BM981135.1  465  ......................................................................................................................................................  316
BE614257.1  436  ......................................................................................................................................................  287
CB306442.1  465  ......................................................................................................................................................  316
CA418062.1  465  ......................................................................................................................................................  316

Query       699  TGTGGAGGAAACTAAACATTCCCTTGATGGTCTCAAGCTATGATCAGAAGACTTTAATTATATATTTTCATCCTATAAGCTTAAATAGGAAAGTTTCTTCAACAGGATTACAGTGTAGCTACCTACATGCTGAAAAATATAGCCTTTAAA  848
CA447733.1  315  ......................................................................................................................................................  166
CA423056.1  317  ......................................................................................................................................................  168
BU857810.1  517  ...................................................................................N................................................................N.  666
BQ776006.1  315  ......................................................................................................................................................  166
CN386790.1  484  ......................................................................................................................................................  633
BQ228162.1  670  ......................................................................................................................................................  820
                                                                                                          \
                                                                                                          |
                                                                                                          A
BM981135.1  315  ......................................................................................................................................................  166
BE614257.1  286  ......................................................................................................................................................  137
CB306442.1  315  ......................................................................................................................................................  166
CA418062.1  315  ......................................................................................................................................................  166

Query       849  TCATTTTTATATTATAACTCTGTATAATAGAGATAAGTCCATTTTTTAAAAATGTTTTCCCCAAACCATAAAACCCTATACAAGTTGTTCTAGTAACAATACATGAGAAAGATGTCTATGTAGCTGAAAATAAAATGACGT  989
CA447733.1  165  .............................................................................................................................................  25
CA423056.1  167  .................................................................................................................................G...........  27
BU857810.1  667  ................................................N................................................N...........................................  807
BQ776006.1  165  .............................................................................................................................................  25
CN386790.1  634  ......................................................G..............................................................................         766
BQ228162.1  821  .............................................................................................G...............................................  964
                                                                                                    \                      \                       \
                                                                                                    |                      |                       |
                                                                                                    G                      G                       A
BM981135.1  165  .............................................................................................................................................  25
BE614257.1  136  ..............................................................................................................T.A-.................           6
                                                                                                                                             \
                                                                                                                                             |
                                                                                                                                             T
CB306442.1  165  .............................................................................................................................................  25
CA418062.1  165  .............................................................................................................................................  25
```

Query= T22-21 [organism=Homo sapiens] anxa1

Length=1084

```
                                                              Score        E     Max
Sequences producing significant alignments:                  (Bits)     Value  ident

CB306442.1 UI-CF-EN1-aej-e-15-0-UI.s1 UI-CF-EN1 Homo sapiens c...  1288       0.0    100%
CA447733.1 UI-H-EI0-ayf-a-16-0-UI.s1 NCI_CGAP_EI0 Homo sapiens...  1288       0.0    100%
```

```
CA447223.1 UI-H-EI0-ayd-b-19-0-UI.s1 NCI_CGAP_EI0 Homo sapiens...  1288       0.0    100%
CA422748.1 UI-H-FL0-bdr-g-09-0-UI.s1 NCI_CGAP_FL0 Homo sapiens...  1288       0.0    100%
CA419844.1 UI-H-FH0-bcm-d-16-0-UI.s1 NCI_CGAP_FH0 Homo sapiens...  1288       0.0    100%
CA417462.1 UI-H-FE0-bbw-c-13-0-UI.s1 NCI_CGAP_FE0 Homo sapiens...  1288       0.0    100%
CA415613.1 UI-H-EZ0-bay-p-10-0-UI.s1 NCI_CGAP_Ch1 Homo sapiens...  1288       0.0    100%
CA414702.1 UI-H-EZ0-bar-e-18-0-UI.s1 NCI_CGAP_Ch1 Homo sapiens...  1288       0.0    100%
BU633206.1 UI-H-FL1-bgt-f-12-0-UI.s1 NCI_CGAP_FL1 Homo sapiens...  1288       0.0    100%
BQ776306.1 UI-H-FH0-bcn-i-12-0-UI.s1 NCI_CGAP_FH0 Homo sapiens...  1288       0.0    100%

ALIGNMENTS

Query       388   AGGAGAAAGGAGAAAGGGGACAGACGTAAACGTGTTCAATACCATCCTTACCACCAGAAGCTATCCACAACTTCGCAGAGTGTTTCAGAAATACACCAAGTACAGTAAGCATGACATGAACAAAGTTCTGGACCTGGAGTTGAAAGGTGA  537
CB306442.1  715   ......................................................................................................................................................  566
CA447733.1  715   ......................................................................................................................................................  566
CA447223.1  715   ......................................................................................................................................................  566
CA422748.1  717   ......................................................................................................................................................  568
CA419844.1  715   ......................................................................................................................................................  566
CA417462.1  712   ......................................................................................................................................................  563
CA415613.1  717   ......................................................................................................................................................  568
CA414702.1  717   ......................................................................................................................................................  568
BU633206.1  715   ......................................................................................................................................................  566
BQ776306.1  715   ......................................................................................................................................................  566

Query       538   CATTGAGAAATGCCTCACAGCTATCGTGAAGTGCGCCACAAGCAAACCAGCTTTCTTTGCAGAGAAGCTTCATCAAGCCATGAAAGGTGTTGGAACTCGCCATAAGGCATTGATCAGGATTATGGTTTCCCGTTCTGAAATTGACATGAA  687
CB306442.1  565   ......................................................................................................................................................  416
CA447733.1  565   ......................................................................................................................................................  416
CA447223.1  565   ......................................................................................................................................................  416
CA422748.1  567   ......................................................................................................................................................  418
CA419844.1  565   ......................................................................................................................................................  416
CA417462.1  562   ......................................................................................................................................................  413
CA415613.1  567   ......................................................................................................................................................  418
CA414702.1  567   ......................................................................................................................................................  418
BU633206.1  565   ......................................................................................................................................................  416
BQ776306.1  565   ......................................................................................................................................................  416

Query       688   TGATATCAAAGCATTCTATCAGAAGATGTATGGTATCTCCCTTTGCCAAGCCATCCTGGATGAAACCAAAGGAGATTATGAGAAAATCCTGGTGGCTCTTTGTGGAGGAAACTAAACATTCCCTTGATGGTCTCAAGCTATGATCAGAAG  837
CB306442.1  415   ......................................................................................................................................................  266
CA447733.1  415   ......................................................................................................................................................  266
CA447223.1  415   ......................................................................................................................................................  266
CA422748.1  417   ......................................................................................................................................................  268
CA419844.1  415   ......................................................................................................................................................  266
CA417462.1  412   ......................................................................................................................................................  263
CA415613.1  417   ......................................................................................................................................................  268
CA414702.1  417   ......................................................................................................................................................  268
BU633206.1  415   ......................................................................................................................................................  266
BQ776306.1  415   ......................................................................................................................................................  266

Query       838   ACTTTAATTATATATTTTCATCCTATAAGCTTAAATAGGAAAGTTTCTTCAACAGGATTACAGTGTAGCTACCTACATGCTGAAAAATATAGCCTTTAAATCATTTTTATATTATAACTCTGTATAATAGAGATAAGTCCATTTTTTAAA  987
CB306442.1  265   ......................................................................................................................................................  116
CA447733.1  265   ......................................................................................................................................................  116
CA447223.1  265   ......................................................................................................................................................  116
CA422748.1  267   ......................................................................................................................................................  118
CA419844.1  265   ......................................................................................................................................................  116
CA417462.1  262   ......................................................................................................................................................  113
CA415613.1  267   ......................................................................................................................................................  118
CA414702.1  267   ......................................................................................................................................................  118
BU633206.1  265   ......................................................................................................................................................  116
BQ776306.1  265   ......................................................................................................................................................  116

Query       988   AATGTTTTCCCCAAACCATAAAACCCTATACAAGTTGTTCTAGTAACAATACATGAGAAAGATGTCTATGTAGCTGAAAATAAAATGACGTCACAAG  1084
CB306442.1  115   .................................................................................................  19
CA447733.1  115   .................................................................................................  19
CA447223.1  115   .................................................................................................  19
CA422748.1  117   .................................................................................................  21
CA419844.1  115   .................................................................................................  19
CA417462.1  112   .................................................................................................  16
CA415613.1  117   .................................................................................................  21
CA414702.1  117   .................................................................................................  21
BU633206.1  115   .................................................................................................  19
BQ776306.1  115   .................................................................................................  19

  Database: Database of GenBank+EMBL+DDBJ sequences from EST Divisions
    Posted date:  Feb 24, 2019  1:46 AM
  Number of letters in database: 43,200,890,307
  Number of sequences in database:  77,566,966

Lambda      K        H
    1.33    0.621     1.12
Gapped
Lambda      K        H
    1.28    0.460    0.850
Matrix: blastn matrix:1 -2
```

Gap Penalties: Existence: 0, Extension: 0
Number of Sequences: 77566966
Number of Hits to DB: 3240899
Number of extensions: 1347
Number of successful extensions: 1347
Number of sequences better than 10: 64
Number of HSP's better than 10 without gapping: 0
Number of HSP's gapped: 1343
Number of HSP's successfully gapped: 1343
Length of database: 43200890307
A: 0
X1: 13 (25.0 bits)
X2: 32 (59.1 bits)
X3: 54 (99.7 bits)
S1: 13 (25.1 bits)
BLASTN 2.9.0+
Reference: Zheng Zhang, Scott Schwartz, Lukas Wagner, and
Webb Miller (2000), "A greedy algorithm for aligning DNA
sequences", J Comput Biol 2000; 7(1-2):203-14.

RID: 8RBRB7KC015

Database: Database of GenBank+EMBL+DDBJ sequences from EST Divisions
77,566,966 sequences; 43,200,890,307 total letters
Query= N1-C [organism=Homo sapiens] anxa1

Length=1485

Sequences producing significant alignments:	Score (Bits)	E Value	Max ident
BU508007.1 AGENCOURT_10128424 NIH_MGC_71 Homo sapiens cDNA clo...	1929	0.0	98%
AL570428.3 AL570428 Homo sapiens PLACENTA COT 25-NORMALIZED Ho...	1910	0.0	98%
AL544231.3 AL544231 Homo sapiens PLACENTA COT 25-NORMALIZED Ho...	1905	0.0	98%
DC631365.1 DC631365 macaque bone marrow cDNA library QbmA Maca...	1879	0.0	94%
DC636772.1 DC636772 macaque bone marrow cDNA library QbmA Maca...	1853	0.0	94%
AL541874.3 AL541874 Homo sapiens PLACENTA Homo sapiens cDNA cl...	1853	0.0	99%
BU902298.1 AGENCOURT_10127317 NIH_MGC_71 Homo sapiens cDNA clo...	1847	0.0	96%
BX438944.2 BX438944 Homo sapiens PLACENTA Homo sapiens cDNA cl...	1844	0.0	99%
AL553114.3 AL553114 Homo sapiens PLACENTA COT 25-NORMALIZED Ho...	1842	0.0	99%
AL576223.3 AL576223 Homo sapiens PLACENTA COT 25-NORMALIZED Ho...	1831	0.0	98%

ALIGNMENTS

```
Query       2     AAACAGAAGGCAGCCAATTACTAACTTCTGGTTGCTAGGTGTGGCTTCCTTTAAAATCCTATAAAATCAGAAGCCCAAGTCTCCACTGCCAGTGTGAAATCTTCAGAGAAGAATTTCTCTTTAGTTCTTTGCAAGAAGGTAGAGATAAAG  151
BU508007.1  1                                                                                                                   ........................................  40
AL544231.3  19                                                                                                             ..T........................................  61
DC631365.1  1                                                                                               ......-....................C.C..............................  59
DC636772.1  1                                                                                               ......-....................C.C..............................  59
AL541874.3  1                                                                                                                        ...................................  35
BU902298.1  24                                                                                                    ..................................................  73
BX438944.2  1                                                                                                              ...........................................  43
AL553114.3  17    ......................................................................................................................................................  166

Query       152   ACACTTTTTCAAAAATGGCAATGGTATCAGAATTCCTCAAGCAGGCCTGGTTTATTGAAAATGAAGAGCAGGAATATGTTCAAACTGTGAAGTCATCCAAAGGTGGTCCCGGATCAGCGGTGAGCCCCTATCCTACCTTCAATCCATCCT  301
BU508007.1  41    ......................................................................................................................................................  191
                     \
                     |
                     N
AL544231.3  62    ......................................................................................................................................................  211
DC631365.1  60    .................................................................................................A...............T.......A............................  209
DC636772.1  60    .................................................................................................A...............T.......A............................  209
AL541874.3  36    ......................................................................................................................................................  185
BU902298.1  74    ......................................................................................................................................................  224
                     \
                     |
                     N
BX438944.2  44    ......................................................................................................................................................  193
AL553114.3  167   ......................................................................................................................................................  316

Query       302   CGGATGTCGCTGCCTTGCATAAGGCCATAATGGTTAAAGGTGTGGATGAAGCAACCATCATTGACATTCTAACTAAGCGAAACAATGCACAGCGTCAACAGATCAAAGCAGCATATCTCCAGGAAACAGGAAAGCCCCTGGATGAAACAC  451
BU508007.1  192   ......................................................................................................................................................  341
AL570428.3  1088                                                                     .....-.T............-.....T....-.A..-..........W.......-........GC.................  1011
AL544231.3  212   ......................................................................................................................................................  361
DC631365.1  210   .......T...........C..................................................................................................................................  359
DC636772.1  210   ...................C..................................................................................................................................  359
AL541874.3  186   ......................................................................................................................................................  335
BU902298.1  225   ......................................................................................................................................................  374
BX438944.2  194   ......................................................................................................................................................  343
```

```
AL553114.3  317   ......................................................................................................................................................  466
AL576223.3  1050                                                                                                 ......-W......-R..-.Y...-.......-........-.R...................  1002

Query       452   TGAAGAAAGCCCTTACAGGTCACCTTGAGGAGGTTGTTTTAGCTCTGCTAAAAACTCCAGCGCAATTTGATGCTGATGAACTTCGTGCTGCCATGAAGGGCCTTGGAACTGATGAAGATACTCTAATTGAGATTTTGGCATCAAGAACTA  601
BU508007.1  342   ......................................................................................................................................................  491
AL570428.3  1010  ...........-Y.............................................................................................M...........................................  862
AL544231.3  362   ......................................................................................................................................................  511
DC631365.1  360   ...............TG....................................................................G................................C....G..........................  509
DC636772.1  360   .............G.TG....................................................................G................................C....G..........................  509
AL541874.3  336   ......................................................................................................................................................  485
BU902298.1  375   ......................................................................................................................................................  524
BX438944.2  344   ......................................................................................................................................................  493
AL553114.3  467   ......................................................................................................................................................  616
AL576223.3  1001  ...........-.....-.D.....WR............-.A....-V..................................-........................................W..AAR.....................  857

Query       602   ACAAAGAAATCAGAGACATTAACAGGGTCTACAGAGAGGAACTGAAGAGAGATCTGGCCAAAGACATAACCTCAGACACATCTGGAGATTTTCGGAACGCTTTGCTTTCTCTTGCTAAGGGTGACCGATCTGAGGACTTTGGTGTGAATG  751
BU508007.1  492   ......................................................................................................................................................  641
AL570428.3  861   ....................W.................................................................................................................................  712
AL544231.3  512   ......................................................................................................................................................  661
DC631365.1  510   ....G.................................................................................................................................................  659
DC636772.1  510   ......................................................................................................................................................  659
AL541874.3  486   ......................................................................................................................................................  635
BU902298.1  525   ......................................................................................................................................................  674
BX438944.2  494   ......................................................................................................................................................  643
AL553114.3  617   ......................................................................................................................................................  766
AL576223.3  856   ......................................................................................................................................................  707

Query       752   AAGACTTGGCTGATTCAGATGCCAGGGCCTTGTATGAAGCAGGAGAAAGGAGAAAGGGGACAGACGTAAACGTGTTCAATACCATCCTTACCACCAGAAGCTATCCACAACTTCGCAGAGTGTTTCAGAAATACACCAAGTACAGTAAGC  901
BU508007.1  642   ......................................................................................................................................................  791
AL570428.3  711   ..R.......................................................N...........................................................................................  562
AL544231.3  662   ......................................................................................................................................................  811
DC631365.1  660   ........................................G.......................T...............................C.............T.......................................  809
DC636772.1  660   ........................................G.......................T...............................C.............T.......................................  809
AL541874.3  636   ......................................................................................................................................................  785
BU902298.1  675   ......................................................................................................................................................  824
BX438944.2  644   ......................................................................................................................................................  793
AL553114.3  767   ......................................................................................................................................................  916
AL576223.3  706   ......................................................................................................................................................  557

Query       902   ATGACATGAACAAAGTTCTGGACCTGGAGTTGAAAGGTGACATTGAGAAATGCCTCACAGCTATCGTGAAGTGCGCCACAAGCAAACCAGCTTTCTTTGCAGAGAAGCTTCATCAAGCCATGAAAGGTGTTGGAACTCGCCATAAGGCAT  1051
BU508007.1  792   ..................................................................................................................-...........................C.......  940
AL570428.3  561   ..A...................................................................................................................................................  412
AL544231.3  812   ...........................................................................-..........................................................................  960
DC631365.1  810   .........................................................................T................................A........G.............C....................  959
DC636772.1  810   .........................................................................T................................A........G.............C..........-.........  959
                                                                                                                                                                 \
                                                                                                                                                                 |
                                                                                                                                                                 T
AL541874.3  786   ......................................................................................................................................................  935
BU902298.1  825   ..............................................A.....................TG..........................C..................................................C..  974
BX438944.2  794   ..................................................................................................................................K..R.............S..  943
AL553114.3  917   .................................................W..-...........................-..-................-....-.........-..M-......                          1035
AL576223.3  556   ......................................................................................................................................................  407

Query       1052  TGATCAGGATTATGGTTTCCCGTTCTGAAATTGACATGAATGATATCAAAGCATTCTATCAGAAGATGTATGGTATCTCCCTTTGCCAAGCCATCCTGGATGAAACCAAAGGAGATTATGAGAAAATCCTGGTGGCTCTTTGTGGAGGAA  1201
BU508007.1  941   .............................................................-......G....G...............C..T...G..........C...GA..T..TA..............................  1093
                               \                                             \                  \                                                      \
                               |                                             |                  |                                                      |
                               G                                             T                  C                                                      G
AL570428.3  411   ................................A.........................................T...........................................................................  262
AL544231.3  961   ...............................................................G.W-......WWY-..............MWY.-........-.......R...-........-..Y..K-.......K-...R....  1102
DC631365.1  960   ...................A..........................................-.........................-......-..-.....-.-.--......-....-...-..-....-.............-..  1096
                               \
                               |
                               G
DC636772.1  960   ...................A..................-...............................................-.-..-...-..-.....GG-.--......-....-......-.....-............-..  1099
                               \                                          \                                                                                 \   \
                               |                                          |                                                                                 |   |
                               G                                          T                                                                                 C   G
AL541874.3  936   .................................................-....-........-.....W..K-......-.......-..M-..-......                                                1029
BU902298.1  975   ........GA.....G...................T.......A...C....T........A....A.G....A.......C...G...C..C......T..........G..A.......A.......C..G.............A...  1132
                                                                    \                  \                  \            \                 \               \\        \
                                                                    |                  |                  |            |                 |               ||        |
                                                                    G                  A                  C            G                 G               G|        G
                                                                                                                                                          |
                                                                                                                                                          C
BX438944.2  944   .........................................D.......R-....Y.............W..KD-.Y.......Y...                                                              1030
                                                          \
                                                          |
                                                          A
AL576223.3  406   ......................................................................................................................................................  257
```

```
Query       1202  ACTAAACATTCCCTTGATGGTCTCAAGCTATGATCAGAAGACTTTAATTATATATTTTCATCCTATAAGCTTAAATAGGAAAGTTTCTTCAACAGGATTACAGTGTAGCTACCTACATGCTGAAAAATATAGCCTTTAAATCATTTTTAT  1351
BU508007.1  1094  .............C......C.....                                                                                                                              1123
                   \        \      \
                   |        |      |
                   A        T     AG
AL570428.3  261   ......................................................................................................................................................  112
DC631365.1  1097  -....-...-....-....A-...-.....................-........CA...G..-.-..C...-.-..C.-...-..-.--......-..C..--.........T-......C...-.....-.A....            1216
                                                         \                                                                                |
                                                         |                                                                                |
                                                         A                                                                                G
DC636772.1  1100  -....-...-.........-.....-....GA...........C-.......-....-....-..-.-...G..-...-.....C-..-.--.........G...-..A.-...T-........T..GG.--...A....A.........  1229
                                                                                                                                          \                           \
                                                                                                                                          |                           |
                                                                                                                                          G                           A
BU902298.1  1133  .AA..............                                                                                                                                       1152
                       \  \  \
                       |  |  |
                       T  A  T
AL576223.3  256   ......................................................................................................................................................  107

Query       1352  ATTATAACTCTGTATAATAGAGATAAGTCCATTTTTTAAAAATGTTTTCCCCAAACCATAAAACCCTATACAAGTTGTTCTAGTAACAATACATGAGAAAGATGTCTATGT  1462
AL570428.3  111   ...............T...............................................................................................  1
DC636772.1  1230  ..                                                                                                               1231
AL576223.3  106   .................................................B........................................................       1

Query= N2-26 [organism=Homo sapiens] anxa1

Length=988


                                                                     Score        E     Max
Sequences producing significant alignments:                         (Bits)     Value  ident

CA447733.1 UI-H-EI0-ayf-a-16-0-UI.s1 NCI_CGAP_EI0 Homo sapiens...  1349       0.0    99%
CA423056.1 UI-H-FL0-bdk-f-10-0-UI.s1 NCI_CGAP_FL0 Homo sapiens...  1343       0.0    99%
BQ776006.1 UI-H-FH0-bck-h-18-0-UI.s1 NCI_CGAP_FH0 Homo sapiens...  1334       0.0    99%
BU857810.1 AGENCOURT_10474308 NIH_MGC_107 Homo sapiens cDNA cl...  1330       0.0    99%
CN386790.1 17000599887650 GRN_PRENEU Homo sapiens cDNA 5', mRN...  1323       0.0    99%
BQ228162.1 AGENCOURT_7258587 NIH_MGC_71 Homo sapiens cDNA clon...  1321       0.0    99%
BM981135.1 UI-CF-EN1-adg-a-11-0-UI.s1 UI-CF-EN1 Homo sapiens c...  1317       0.0    99%
CB306442.1 UI-CF-EN1-aej-e-15-0-UI.s1 UI-CF-EN1 Homo sapiens c...  1306       0.0    99%
CA418062.1 UI-H-FH0-bcc-j-24-0-UI.s1 NCI_CGAP_FH0 Homo sapiens...  1306       0.0    99%
CA418210.1 UI-H-FH0-bcf-i-22-0-UI.s1 NCI_CGAP_FH0 Homo sapiens...  1303       0.0    99%

ALIGNMENTS

Query       249  GGTGTGAATGAAGACTTGGCTGATTCAGATGCCAGGGCCTTGTATGAAGCAGGAGAAAGGAGAAAGGGGACAGACGTAAACGTGTTCAATACCATCCTTACCACCAGAAGCTATCCACAACTTCGCAGAGTGTTTCAGAAATACCAAG  398
CA447733.1  765  ......................................................................................................................................................  616
CA423056.1  767  ......................................................................................................................................................  618
BQ776006.1  765  ..................-..................................................................................................................N................  616
                                         \
                                         |
                                         T
BU857810.1  67   ......................................................................................................................................................  216
CN386790.1  34   ......................................................................................................................................................  183
BQ228162.1  220  ......................................................................................................................................................  369
BM981135.1  760   ............-....................-..N..........................-.....................................-......................................  616
CB306442.1  744                       ...............N.....................................................................................................................  616
CA418062.1  744                       ...............................................N...................................................................................  616
CA418210.1  740                       ...............................................-...................................................................................  613

Query       399  TACAGTAAGCATGACATGAACAAAGTTCTGGACCTGGAGTTGAAAGGTGACATTGAGAAATGCCTCACAGCTATCGTGAAGTGCGCCACAAGCAAACCAGCTTTCTTTGCAGAGAAGCTTCATCAAGCCATGAAAGGTGTTGGAACTCGC  548
CA447733.1  615  ......................................................................................................................................................  466
CA423056.1  617  ......................................................................................................................................................  468
BQ776006.1  615  ......................................................................................................................................................  466
BU857810.1  217  ......................................................................................................................................................  366
CN386790.1  184  ......................................................................................................................................................  333
BQ228162.1  370  ......................................................................................................................................................  519
BM981135.1  615  ......................................................................................................................................................  466
CB306442.1  615  ......................................................................................................................................................  466
CA418062.1  615  ......................................................................................................................................................  466
CA418210.1  612  ......................................................................................................................................................  463

Query       549  CATAAGGCATTGATCAGGATTATGGTTTCCCGTTCTGAAATTGACATGAATGATATCAAAGCATTCTATCAGAAGATGTATGGTATCTCCTTTGCCAAGCCATCCTGGATGAAACCAAAGGAGATTATGAGAAAATCCTGGTGGCTCTTT  698
CA447733.1  465  ......................................................................................................................................................  315
                                                                                            \
                                                                                            |
                                                                                            C
CA423056.1  467  ......................................................................................................................................................  317
                                                                                            \
                                                                                            |
                                                                                            C
```

```
BQ776006.1  465  ......................................................................................................................................................  315
                                                                                                           \
                                                                                                           |
                                                                                                           C
BU857810.1  367  ......................................................................................................................................................  517
                                                                                                         \
                                                                                                         |
                                                                                                         C
CN386790.1  334  ......................................................................................................................................................  484
                                                                                                         \
                                                                                                         |
                                                                                                         C
BQ228162.1  520  ......................................................................................................................................................  670
                                                                                                         \
                                                                                                         |
                                                                                                         C
BM981135.1  465  ......................................................................................................................................................  315
                                                                                                           \
                                                                                                           |
                                                                                                           C
CB306442.1  465  ......................................................................................................................................................  315
                                                                                                           \
                                                                                                           |
                                                                                                           C
CA418062.1  465  ......................................................................................................................................................  315
                                                                                                           \
                                                                                                           |
                                                                                                           C
CA418210.1  462  ......................................................................................................................................................  312
                                                                                                           \
                                                                                                           |
                                                                                                           C

Query       699  GTGGAGGAAACTAAACATTCCTTGATGGTCTCAAGCTATGATCAGAAGACTTTAATTATATATTTTCATCCTATAAGCTTAAATAGGAAAGTTTCTTCAACAGGATTACAGTGTAGCTACCTACATGCTGAAAAATATAGCCTTTAAATC  848
CA447733.1  314  ......................................................................................................................................................  164
                                      \
                                      |
                                      C
CA423056.1  316  ......................................................................................................................................................  166
                                      \
                                      |
                                      C
BQ776006.1  314  ......................................................................................................................................................  164
                                      \
                                      |
                                      C
BU857810.1  518  .................................................................................N................................................................N...  668
                                    \
                                    |
                                    C
CN386790.1  485  ......................................................................................................................................................  635
                                    \
                                    |
                                    C
BQ228162.1  671  ......................................................................................................................................................  822
                                    \                                                                   \
                                    |                                                                   |
                                    C                                                                   A
BM981135.1  314  ......................................................................................................................................................  164
                                      \
                                      |
                                      C
CB306442.1  314  ......................................................................................................................................................  164
                                      \
                                      |
                                      C
CA418062.1  314  ......................................................................................................................................................  164
                                      \
                                      |
                                      C
CA418210.1  311  ......................................................................................................................................................  161
                                      \
                                      |
                                      C

Query       849  ATTTTTATATTATAACTCTGTATAATAGAGATAAGTCCATTTTTTAAAAATGTTTTCCCAAACCATAAAACCTATACAAGTTGTTCTAGTAACAATACATGAGAAAGATGTCTATGTAGCTGAAAATAAAATGACGTCAC  988
CA447733.1  163  ............................................................................................................................................  22
                                                                            \          \
                                                                            |          |
                                                                            C          C
CA423056.1  165  .............................................................................................................................G..............  24
                                                                            \          \
```

```
                                                                            |          |
                                                                            C          C
BQ776006.1  163  ............................................................................................................................................  22
                                                                            \          \
                                                                            |          |
                                                                            C          C
BU857810.1  669  ..............................................N...............................................N............................................    808
                                                                         \             \
                                                                         |             |
                                                                         C             C
CN386790.1  636  ....................................................G............................................................................             766
                                                                         \             \
                                                                         |             |
                                                                         C             C
BQ228162.1  823  .........................................................................................G................................................    965
                                                                         \                \     \                      \                   \
                                                                         |                |     |                      |                   |
                                                                         C                C     G                      G                   A
BM981135.1  163  ............................................................................................................................................  22
                                                                            \          \
                                                                            |          |
                                                                            C          C
CB306442.1  163  ............................................................................................................................................  22
                                                                            \          \
                                                                            |          |
                                                                            C          C
CA418062.1  163  ............................................................................................................................................  22
                                                                            \          \
                                                                            |          |
                                                                            C          C
CA418210.1  160  ............................................................................................................................................  19
                                                                            \          \
                                                                            |          |
                                                                            C          C

Query= N3-26 [organism=Homo sapiens] anxa1

Length=1311


                                                                  Score        E     Max
Sequences producing significant alignments:                      (Bits)     Value  ident

BE614257.1 601504294T1 NIH_MGC_71 Homo sapiens cDNA clone IMAG...  1303       0.0    100%
AL576223.3 AL576223 Homo sapiens PLACENTA COT 25-NORMALIZED Ho...  1299       0.0    100%
CN386790.1 17000599887650 GRN_PRENEU Homo sapiens cDNA 5', mRN...  1297       0.0    100%
CA447733.1 UI-H-EI0-ayf-a-16-0-UI.s1 NCI_CGAP_EI0 Homo sapiens...  1297       0.0    100%
CA423056.1 UI-H-FL0-bdk-f-10-0-UI.s1 NCI_CGAP_FL0 Homo sapiens...  1297       0.0    100%
AL574991.3 AL574991 Homo sapiens PLACENTA COT 25-NORMALIZED Ho...  1295       0.0    100%
AL576603.2 AL576603 Homo sapiens PLACENTA COT 25-NORMALIZED Ho...  1295       0.0    100%
AL576340.3 AL576340 Homo sapiens PLACENTA COT 25-NORMALIZED Ho...  1290       0.0    100%
BE620646.1 601483620T1 NIH_MGC_69 Homo sapiens cDNA clone IMAG...  1290       0.0    99%
BU857810.1 AGENCOURT_10474308 NIH_MGC_107 Homo sapiens cDNA cl...  1288       0.0    99%

ALIGNMENTS

Query       306   CAACAGATCAAAGCAGCATATCTCCAGGAAACAGGAAAGCCCTGGATGAAACACTGAAGAAAGCCCTTACAGGTCACCTTGAGGAGGTTGTTTTAGCTCTGCTAAAAACTCCAGCGCAATTTGATGCTGATGAACTTCGTGCTGCCATGA  455
AL576223.3  1050  ......-W......-R...-.Y....-........-......CT.R..................-.....-.D.....WR...........-.A....-V...................................-.............  911
AL574991.3  986                                                                            ..............-...K...........................................-..............  911
AL576603.2  917                                                                                                                                                     ....  914
AL576340.3  1011                                         ....-....-......-...-...G..-....-....-.Y...-..-....-........-...-........-......-................-.....-K.....  917

Query       456   AGGGCCTTGGAACTGATGAAGATACTCTAATTGAGATTTTGGCATCAAGAACTAACAAAGAAATCAGAGACATTAACAGGGTCTACAGAGAGGAACTGAAGAGAGATCTGGCCAAAGACATAACCTCAGATCGGAGGCTAGTGCATCTGA  605
AL576223.3  910   ...........................W..AAR..................................................................................................                     781
CN386790.1  28                                                                                                                                                        ..  29
AL574991.3  910   ..................................................................................................................................                     781
AL576603.2  913   .-........-...........-WM..............-..........................................................................................                     788
AL576340.3  916   .-....-.............-RW..................-.......-................................................................................                     792
BE620646.1  748                                                                                                                                                 ......  743
BU857810.1  61                                                                                                                                                        ..  62

Query       606   GTTTGGTGTGAATGAAGACTTGGCTGATTCAGATGCCAGGGCCTTGTATGAAGCAGGAGAAAGGAGAAAGGGGACAGACGTAAACGTGTTCAATACCATCCTTACCACCAGAAGCTATCCACAACTTCGCAGAGTGTTTCAGAAATACAC  755
BE614257.1  739    .....................................................................................................................................................  591
AL576223.3  719    .....................................................................................................................................................  571
CN386790.1  30    C.....................................................................................................................................................  179
CA447733.1  765       ..................................................................................................................................................  620
CA423056.1  767       ..................................................................................................................................................  622
AL574991.3  719    .....................................................................................................................................................  571
AL576603.2  726    .....................................................................................................................................................  578
AL576340.3  730    .....................................................................................................................................................  582
BE620646.1  742   ......................................................................................................................................................  591
                   \
```

```
                   |
                  AC
BU857810.1  63    C.....................................................................................................................................................  212

Query       756   CAAGTACAGTAAGCATGACATGAACAAAGTTCTGGACCTGGAGTTGAAAGGTGACATTGAGAAATGCCTCACAGCTATCGTGAAGTGCGCCACAAGCAAACCAGCTTTCTTTGCAGAGAAGCTTCATCAAGCCATGAAAGGTGTTGGAAC  905
BE614257.1  590   ......................................................................................................................................................  441
AL576223.3  570   ......................................................................................................................................................  421
CN386790.1  180   ......................................................................................................................................................  329
CA447733.1  619   ......................................................................................................................................................  470
CA423056.1  621   ......................................................................................................................................................  472
AL574991.3  570   ......................................................................................................................................................  421
AL576603.2  577   ......................................................................................................................................................  428
AL576340.3  581   ......................................................................................................................................................  432
BE620646.1  590   ......................................................................................................................................................  441
BU857810.1  213   ......................................................................................................................................................  362

Query       906   TCGCCATAAGGCATTGATCAGGATTATGGTTTCCCGTTCTGAAATTGACATGAATGATATCAAAGCATTCTATCAGAAGATGTATGGTATCTCCCTTTGCCAAGCCATCCTGGATGAAACCAAAGGAGATTATGAGAAAATCCTGGTGGC  1055
BE614257.1  440   ......................................................................................................................................................  291
AL576223.3  420   ......................................................................................................................................................  271
CN386790.1  330   ......................................................................................................................................................  479
CA447733.1  469   ......................................................................................................................................................  320
CA423056.1  471   ......................................................................................................................................................  322
AL574991.3  420   ..........................................................................................................W...........................................  271
AL576603.2  427   ......................................................................................................................................................  278
AL576340.3  431   ......................................................................................................................R.......R.......................  282
BE620646.1  440   ......................................................................................................................................................  291
BU857810.1  363   ......................................................................................................................................................  512

Query       1056  TCTTTGTGGAGGAAACTAAACATTCCCTTGATGGTCTCAAGCTATGATCAGAAGACTTTAATTATATATTTTCATCCTATAAGCTTAAATAGGAAAGTTTCTTCAACAGGATTACAGTGTAGCTACCTACATGCTGAAAAATATAGCCTT  1205
BE614257.1  290   ......................................................................................................................................................  141
AL576223.3  270   ......................................................................................................................................................  121
CN386790.1  480   ......................................................................................................................................................  629
CA447733.1  319   ......................................................................................................................................................  170
CA423056.1  321   ......................................................................................................................................................  172
AL574991.3  270   ...................................Y..................................................................................................................  121
AL576603.2  277   ......................................................................................................................................................  128
AL576340.3  281   ......................................................................................................................................................  132
BE620646.1  290   .........................................................................................................-............................................  140
                                                                          \                              \
                                                                          |                              |
                                                                          A                              C
BU857810.1  513   .......................................................................................N..............................................................  662

Query       1206  TAAATCATTTTTATATTATAACTCTGTATAATAGAGATAAGTCCATTTTTTAAAAATGTTTTCCCCAAACCATAAAACCCTATACAAGTTGTTCTAGTAACAATAC  1311
BE614257.1  140   ..........................................................................................................  35
AL576223.3  120   ...............................................................B..........................................  15
CN386790.1  630   ..........................................................G...............................................  735
CA447733.1  169   ..........................................................................................................  64
CA423056.1  171   ..........................................................................................................  66
AL574991.3  120   ..........................................................................................................  15
AL576603.2  127   ......................................................................................................      26
AL576340.3  131   ..........................................................................................................  25
                                                                                                                         \
                                                                                                                         |
                                                                                                                         N
BE620646.1  139   ..........................................................................................................  34
BU857810.1  663   ..N.................................................N................................................N....  768
```

Query= N4-23 [organism=Homo sapiens] anxa1

Length=1119

```
                                                               Score        E     Max
Sequences producing significant alignments:                   (Bits)     Value  ident

BU857810.1 AGENCOURT_10474308 NIH_MGC_107 Homo sapiens cDNA cl...  1387       0.0    99%
CN386790.1 17000599887650 GRN_PRENEU Homo sapiens cDNA 5', mRN...  1384       0.0    100%
BQ228162.1 AGENCOURT_7258587 NIH_MGC_71 Homo sapiens cDNA clon...  1378       0.0    99%
CA447733.1 UI-H-EI0-ayf-a-16-0-UI.s1 NCI_CGAP_EI0 Homo sapiens...  1367       0.0    100%
BE614257.1 601504294T1 NIH_MGC_71 Homo sapiens cDNA clone IMAG...  1363       0.0    99%
CA423056.1 UI-H-FL0-bdk-f-10-0-UI.s1 NCI_CGAP_FL0 Homo sapiens...  1362       0.0    100%
AL576603.2 AL576603 Homo sapiens PLACENTA COT 25-NORMALIZED Ho...  1358       0.0    100%
AL576340.3 AL576340 Homo sapiens PLACENTA COT 25-NORMALIZED Ho...  1356       0.0    100%
AL576223.3 AL576223 Homo sapiens PLACENTA COT 25-NORMALIZED Ho...  1354       0.0    100%
BQ776006.1 UI-H-FH0-bck-h-18-0-UI.s1 NCI_CGAP_FH0 Homo sapiens...  1352       0.0    100%
```

ALIGNMENTS

```
Query       361   TGACCGATCTGAGGACTTTGGTGTGAATGAAGACTTGGCTGATTCAGATGCCAGGGCCTTGTATGAAGCAGGAGAAAGGAGAAAGGGGACAGACGTAAACGTGTTCAATACCATCCTTACCACCAGAAGCTATCCACAACTTCGCAGAGT  510
BU857810.1  48    ......................................................................................................................................................  197
CN386790.1  15    ......................................................................................................................................................  164
```

```
BQ228162.1  201   ......................................................................................................................................................  350
CA447733.1  765                      ...................................................................................................................................  635
BE614257.1  755   ......................................................................................................................................................  606
CA423056.1  767                      ...................................................................................................................................  637
AL576603.2  742   ......................................................................................................................................................  593
AL576340.3  746   ......................................................................................................................................................  597
AL576223.3  735   ......................................................................................................................................................  586
BQ776006.1  765                      ..................-................................................................................................................  635
                                                            \
                                                            |
                                                            T

Query       511   GTTTCAGAAATACACCAAGTACAGTAAGCATGACATGAACAAAGTTCTGGACCTGGAGTTGAAAGGTGACATTGAGAAATGCCTCACAGCTATCGTGAAGTGCGCCACAAGCAAACCAGCTTTCTTTGCAGAGAAGCTTCATCAAGCCAT  660
BU857810.1  198   ......................................................................................................................................................  347
CN386790.1  165   ......................................................................................................................................................  314
BQ228162.1  351   ......................................................................................................................................................  500
CA447733.1  634   ......................................................................................................................................................  485
BE614257.1  605   ......................................................................................................................................................  456
CA423056.1  636   ......................................................................................................................................................  487
AL576603.2  592   ......................................................................................................................................................  443
AL576340.3  596   ......................................................................................................................................................  447
AL576223.3  585   ......................................................................................................................................................  436
BQ776006.1  634   ..N...................................................................................................................................................  485

Query       661   GAAAGGTGTTGGAACTCGCCATAAGGCATTGATCAGGATTATGGTTTCCCGTTCTGAAATTGACATGAATGATATCAAAGCATTCTATCAGAAGATGTATGGTATCTCCCTTTGCCAAGCCATCCTGGATGAAACCAAAGGAGATTATGA  810
BU857810.1  348   ......................................................................................................................................................  497
CN386790.1  315   ......................................................................................................................................................  464
BQ228162.1  501   ......................................................................................................................................................  650
CA447733.1  484   ......................................................................................................................................................  335
BE614257.1  455   ......................................................................................................................................................  306
CA423056.1  486   ......................................................................................................................................................  337
AL576603.2  442   ......................................................................................................................................................  293
AL576340.3  446   ......................................................................................................................................R......R........  297
AL576223.3  435   ......................................................................................................................................................  286
BQ776006.1  484   ......................................................................................................................................................  335

Query       811   GAAAATCCTGGTGGCTCTTTGTGGAGGAAACTAAACATTCCCTTGATGGTCTCAAGCTATGATCAGAAGACTTTAATTATATATTTTCATCCTATAAGCTTAAATAGGAAAGTTTCTTCAACAGGATTACAGTGTAGCTACCTACATGCT  960
BU857810.1  498   ......................................................................................................N...............................................  647
CN386790.1  465   ......................................................................................................................................................  614
BQ228162.1  651   ......................................................................................................................................................  801
                                                                                                                          \
                                                                                                                          |
                                                                                                                          A
CA447733.1  334   ......................................................................................................................................................  185
BE614257.1  305   ......................................................................................................................................................  156
CA423056.1  336   ......................................................................................................................................................  187
AL576603.2  292   ......................................................................................................................................................  143
AL576340.3  296   ......................................................................................................................................................  147
AL576223.3  285   ......................................................................................................................................................  136
BQ776006.1  334   ......................................................................................................................................................  185

Query       961   GAAAAATATAGCCTTTAAATCATTTTATATTATAACTCTGTATAATAGAGATAAGTCCATTTTTTAAAAATGTTTTCCCCAAACCATAAAACCCTATACAAGTTGTTCTAGTAACAATACATGAGAAAGATGTCTATGTAGCTGAAAAT  1110
BU857810.1  648   .................N.................................................N................................................N.................................  797
CN386790.1  615   .........................................................................G............................................................................  764
BQ228162.1  802   ................................................................................................................G.....................................  954
                                                                                                                    \                      \                       \
                                                                                                                    |                      |                       |
                                                                                                                    G                      G                       A
CA447733.1  184   ......................................................................................................................................................  35
BE614257.1  155   .................................................................................................................................T.A-.................  6
                                                                                                                                                                 \
                                                                                                                                                                 |
                                                                                                                                                                 T
CA423056.1  186   ....................................................................................................................................................G.  37
AL576603.2  142   ......................................................................................................................A......................            1
                                                                                                                                   \
                                                                                                                                   |
                                                                                                                                   C
AL576340.3  146   .............................................................................................................................................            5
                                                                                                                                    \
                                                                                                                                    |
                                                                                                                                    N
AL576223.3  135   ..............................................................................B........................................................                1
BQ776006.1  184   ......................................................................................................................................................  35

Query       1111  AAAATGACG  1119
BU857810.1  798   .........  806
CN386790.1  765   ..         766
BQ228162.1  955   .........  963
CA447733.1  34    .........  26
CA423056.1  36    .........  28
BQ776006.1  34    .........  26
```

```
Query= N5-24 [organism=Homo sapiens] anxa1

Length=1006


                                                                  Score        E     Max
Sequences producing significant alignments:                      (Bits)     Value  ident

CB306442.1 UI-CF-EN1-aej-e-15-0-UI.s1 UI-CF-EN1 Homo sapiens c...  1279       0.0    100%
CA447733.1 UI-H-EI0-ayf-a-16-0-UI.s1 NCI_CGAP_EI0 Homo sapiens...  1279       0.0    100%
CA447223.1 UI-H-EI0-ayd-b-19-0-UI.s1 NCI_CGAP_EI0 Homo sapiens...  1279       0.0    100%
CA422748.1 UI-H-FL0-bdr-g-09-0-UI.s1 NCI_CGAP_FL0 Homo sapiens...  1279       0.0    100%
CA419844.1 UI-H-FH0-bcm-d-16-0-UI.s1 NCI_CGAP_FH0 Homo sapiens...  1279       0.0    100%
CA417462.1 UI-H-FE0-bbw-c-13-0-UI.s1 NCI_CGAP_FE0 Homo sapiens...  1279       0.0    100%
CA415613.1 UI-H-EZ0-bay-p-10-0-UI.s1 NCI_CGAP_Ch1 Homo sapiens...  1279       0.0    100%
CA414702.1 UI-H-EZ0-bar-e-18-0-UI.s1 NCI_CGAP_Ch1 Homo sapiens...  1279       0.0    100%
BU676462.1 UI-CF-DU1-aai-b-16-0-UI.s1 UI-CF-DU1 Homo sapiens c...  1279       0.0    100%
BU633206.1 UI-H-FL1-bgt-f-12-0-UI.s1 NCI_CGAP_FL1 Homo sapiens...  1279       0.0    100%

ALIGNMENTS

Query       315   AAAGGAGAAAGGGGACAGACGTAAACGTGTTCAATACCATCCTTACCACCAGAAGCTATCCACAACTTCGCAGAGTGTTTCAGAAATACACCAAGTACAGTAAGCATGACATGAACAAAGTTCTGGACCTGGAGTTGAAAGGTGACATTG  464
CB306442.1  710   ......................................................................................................................................................  561
CA447733.1  710   ......................................................................................................................................................  561
CA447223.1  710   ......................................................................................................................................................  561
CA422748.1  712   ......................................................................................................................................................  563
CA419844.1  710   ......................................................................................................................................................  561
CA417462.1  707   ......................................................................................................................................................  558
CA415613.1  712   ......................................................................................................................................................  563
CA414702.1  712   ......................................................................................................................................................  563
BU676462.1  707   ......................................................................................................................................................  558
BU633206.1  710   ......................................................................................................................................................  561

Query       465   AGAAATGCCTCACAGCTATCGTGAAGTGCGCCACAAGCAAACCAGCTTTCTTTGCAGAGAAGCTTCATCAAGCCATGAAAGGTGTTGGAACTCGCCATAAGGCATTGATCAGGATTATGGTTTCCCGTTCTGAAATTGACATGAATGATA  614
CB306442.1  560   ......................................................................................................................................................  411
CA447733.1  560   ......................................................................................................................................................  411
CA447223.1  560   ......................................................................................................................................................  411
CA422748.1  562   ......................................................................................................................................................  413
CA419844.1  560   ......................................................................................................................................................  411
CA417462.1  557   ......................................................................................................................................................  408
CA415613.1  562   ......................................................................................................................................................  413
CA414702.1  562   ......................................................................................................................................................  413
BU676462.1  557   ......................................................................................................................................................  408
BU633206.1  560   ......................................................................................................................................................  411

Query       615   TCAAAGCATTCTATCAGAAGATGTATGGTATCTCCCTTTGCCAAGCCATCCTGGATGAAACCAAAGGAGATTATGAGAAAATCCTGGTGGCTCTTTGTGGAGGAAACTAAACATTCCCTTGATGGTCTCAAGCTATGATCAGAAGACTTT  764
CB306442.1  410   ......................................................................................................................................................  261
CA447733.1  410   ......................................................................................................................................................  261
CA447223.1  410   ......................................................................................................................................................  261
CA422748.1  412   ......................................................................................................................................................  263
CA419844.1  410   ......................................................................................................................................................  261
CA417462.1  407   ......................................................................................................................................................  258
CA415613.1  412   ......................................................................................................................................................  263
CA414702.1  412   ......................................................................................................................................................  263
BU676462.1  407   ......................................................................................................................................................  258
BU633206.1  410   ......................................................................................................................................................  261

Query       765   AATTATATATTTTCATCCTATAAGCTTAAATAGGAAAGTTTCTTCAACAGGATTACAGTGTAGCTACCTACATGCTGAAAAATATAGCCTTTAAATCATTTTTATATTATAACTCTGTATAATAGAGATAAGTCCATTTTTTAAAAATGT  914
CB306442.1  260   ......................................................................................................................................................  111
CA447733.1  260   ......................................................................................................................................................  111
CA447223.1  260   ......................................................................................................................................................  111
CA422748.1  262   ......................................................................................................................................................  113
CA419844.1  260   ......................................................................................................................................................  111
CA417462.1  257   ......................................................................................................................................................  108
CA415613.1  262   ......................................................................................................................................................  113
CA414702.1  262   ......................................................................................................................................................  113
BU676462.1  257   ......................................................................................................................................................  108
BU633206.1  260   ......................................................................................................................................................  111

Query       915   TTTCCCCAAACCATAAAACCCTATACAAGTTGTTCTAGTAACAATACATGAGAAAGATGTCTATGTAGCTGAAAATAAAATGACGTCACAAG  1006
CB306442.1  110   ............................................................................................  19
CA447733.1  110   ............................................................................................  19
CA447223.1  110   ............................................................................................  19
CA422748.1  112   ............................................................................................  21
CA419844.1  110   ............................................................................................  19
CA417462.1  107   ............................................................................................  16
CA415613.1  112   ............................................................................................  21
CA414702.1  112   ............................................................................................  21
BU676462.1  107   ............................................................................................  16
BU633206.1  110   ............................................................................................  19
```

```
Query= N6-25 [organism=Homo sapiens] anxa1

Length=1042


                                                                 Score        E     Max
Sequences producing significant alignments:                      (Bits)     Value  ident

AL576223.3 AL576223 Homo sapiens PLACENTA COT 25-NORMALIZED Ho...  1363       0.0    100%
AL574991.3 AL574991 Homo sapiens PLACENTA COT 25-NORMALIZED Ho...  1360       0.0    99%
AL576603.2 AL576603 Homo sapiens PLACENTA COT 25-NORMALIZED Ho...  1360       0.0    100%
BE614257.1 601504294T1 NIH_MGC_71 Homo sapiens cDNA clone IMAG...  1360       0.0    100%
AL576340.3 AL576340 Homo sapiens PLACENTA COT 25-NORMALIZED Ho...  1354       0.0    99%
BU857810.1 AGENCOURT_10474308 NIH_MGC_107 Homo sapiens cDNA cl...  1352       0.0    99%
BQ228162.1 AGENCOURT_7258587 NIH_MGC_71 Homo sapiens cDNA clon...  1352       0.0    99%
AL570884.3 AL570884 Homo sapiens PLACENTA COT 25-NORMALIZED Ho...  1339       0.0    99%
BU596129.1 AGENCOURT_8906716 NIH_MGC_142 Homo sapiens cDNA clo...  1339       0.0    99%
BQ954100.1 AGENCOURT_8869366 NIH_MGC_71 Homo sapiens cDNA clon...  1339       0.0    99%

ALIGNMENTS

Query       298   AACGCTTTGCTTTCTCTTGCTAAGGGTGACCGATCTGAGGACTTTGGTGTGAATGAAGACTTGGCTGATTCAGATGCCAGGGCCTTGTATGAAGCAGGAGAAAGGAGAAAGGGGACAGACGTAAACGTGTTCAATACCATCCTTACCACC  447
AL576223.3  761   ......................................................................................................................................................  612
AL574991.3  761   ......................................................................................................................................................  612
AL576603.2  768   ......................................................................................................................................................  619
BE614257.1  780   ................-.....................................................................................................................................  632
AL576340.3  772   ......................................................................................................................................................  623
BU857810.1  22    ......................................................................................................................................................  171
BQ228162.1  175   ......................................................................................................................................................  324
AL570884.3  768   ......................................................................................................................................................  619
BU596129.1  6     ......................................................................................................................................................  155
BQ954100.1  24    ......................................................................................................................................................  173

Query       448   AGAAGCTATCCACAACTTCGCAGAGTGTTTCAGAAATACACCAAGTACAGTAAGCATGACATGAACAAAGTTCTGGACCTGGAGTTGAAAGGTGACATTGAGAAATGCCTCACAGCTATCGTGAAGTGCGCCACAAGCAAACCAGCTTTC  597
AL576223.3  611   ......................................................................................................................................................  462
AL574991.3  611   ......................................................................................................................................................  462
AL576603.2  618   ......................................................................................................................................................  469
BE614257.1  631   ......................................................................................................................................................  482
AL576340.3  622   ......................................................................................................................................................  473
BU857810.1  172   ......................................................................................................................................................  321
BQ228162.1  325   ......................................................................................................................................................  474
AL570884.3  618   ......................................................................................................................................................  469
BU596129.1  156   ......................................................................................................................................................  305
BQ954100.1  174   ......................................................................................................................................................  323

Query       598   TTTGCAGAGAAGCTTCATCAAGCCATGAAAGGTGTTGGAACTCGCCATAAGGCATTGATCAGGATTATGGTTTCCCGTTCTGAAATTGACATGAATGATATCAAAGCATTCTATCAGAAGATGTATGGTATCTCCTTTGCCAAGCCATCC  747
AL576223.3  461   ......................................................................................................................................................  311
                                                                                                                                                     \
                                                                                                                                                     |
                                                                                                                                                     C

AL574991.3  461   ..................................................................................................................................................W...  311
                                                                                                                                                     \
                                                                                                                                                     |
                                                                                                                                                     C

AL576603.2  468   ......................................................................................................................................................  318
                                                                                                                                                     \
                                                                                                                                                     |
                                                                                                                                                     C

BE614257.1  481   ......................................................................................................................................................  331
                                                                                                                                                     \
                                                                                                                                                     |
                                                                                                                                                     C

AL576340.3  472   ......................................................................................................................................................  322
                                                                                                                                                     \
                                                                                                                                                     |
                                                                                                                                                     C

BU857810.1  322   ......................................................................................................................................................  472
                                                                                                                                                   \
                                                                                                                                                   |
                                                                                                                                                   C

BQ228162.1  475   ......................................................................................................................................................  625
                                                                                                                                                   \
                                                                                                                                                   |
                                                                                                                                                   C

AL570884.3  468   ......................................................................................................................................................  318
                                                                                                                                                     \
                                                                                                                                                     |
                                                                                                                                                     C

BU596129.1  306   ......................................................................................................................................................  456
                                                                                                                                                   \
                                                                                                                                                   |
                                                                                                                                                   C
```

```
BQ954100.1 324   ......................................................................................................................................................  474
                                                                                                                                                                      \
                                                                                                                                                                      |
                                                                                                                                                                      C

Query      748   TGGATGAAACCAAAGGAGATTATGAGAAAATCCTGGTGGCTCTTTGTGGAGGAAACTAAACATTCCCTTGATGGTCTCAAGCTATGATCAGAAGACTTTAATTATATATTTCATCCTATAAGCTTAAATAGGAAAGTTTCTTCAACAGG  897
AL576223.3 310   ......................................................................................................................................................  161
AL574991.3 310   ...........................................................................Y..........................................................................  161
AL576603.2 317   ......................................................................................................................................................  168
BE614257.1 330   ......................................................................................................................................................  181
AL576340.3 321   ........R.......R.....................................................................................................................................  172
BU857810.1 473   ...............................................................................................................................N......................  622
BQ228162.1 626   ......................................................................................................................................................  776
                                                                                                                                                                      \
                                                                                                                                                                      |
                                                                                                                                                                      A

AL570884.3 317   ......................................................................................................................................................  168
BU596129.1 457   ......................................................................................................................................................  606
BQ954100.1 475   ......................................................................................................................................................  624

Query      898   ATTACAGTGTAGCTACCTACATGCTGAAAAATATAGCCTTTAAATCATTTTTATATTATAACTCTGTATAATAGAGATAAGTCCATTTTTTAAAAATGTTTTCCCCAAACCATAAACCCTATACAAGTTGTTCTAGTAACAATAC  1042
AL576223.3 160   .......................................................................................................B.........................................  15
                                                                                                                                     \
                                                                                                                                     |
                                                                                                                                     A

AL574991.3 160   .................................................................................................................................................  15
                                                                                                                                     \
                                                                                                                                     |
                                                                                                                                     A

AL576603.2 167   .............................................................................................................................................      26
                                                                                                                                     \
                                                                                                                                     |
                                                                                                                                     A

BE614257.1 180   .................................................................................................................................................  35
                                                                                                                                     \
                                                                                                                                     |
                                                                                                                                     A

AL576340.3 171   .................................................................................................................................................  25
                                                                                                                                     \                         \
                                                                                                                                     |                         |
                                                                                                                                     A                         N

BU857810.1 623   ..........................................N.................................................N...............................................N....  768
                                                                                                                                  \
                                                                                                                                  |
                                                                                                                                  A

BQ228162.1 777   .........................................................................................................................................G.......  923
                                                                                                                                     \         \
                                                                                                                                     |         |
                                                                                                                                     A         G

AL570884.3 167   .................................................................................................RW....V............................N....N....W..  21
                                                                                                                                     \                 \
                                                                                                                                     |                 |
                                                                                                                                     A                 N

BU596129.1 607   ...........................................................................................T...............CC.....................G..............  753
                                                                                                                                     \
                                                                                                                                     |
                                                                                                                                    AC

BQ954100.1 625   .........................................T........................................................G.........................C.....G..............  771
                                                                                                                                     \         \
                                                                                                                                     |         |
                                                                                                                                     A         G
```

Query= N7-12 [organism=Homo sapiens] anxa1

Length=1094

```
                                                                      Score        E     Max
Sequences producing significant alignments:                          (Bits)     Value  ident

AL553114.3 AL553114 Homo sapiens PLACENTA COT 25-NORMALIZED Ho...  1441       0.0    100%
CX870542.1 HESC4_46_F03.g1_A037 NIH_MGC_262 Homo sapiens cDNA ...  1441       0.0    100%
BQ953713.1 AGENCOURT_8786826 NIH_MGC_43 Homo sapiens cDNA clon...  1432       0.0    100%
BM458053.1 AGENCOURT_6411336 NIH_MGC_71 Homo sapiens cDNA clon...  1430       0.0    100%
AL544959.3 AL544959 Homo sapiens PLACENTA COT 25-NORMALIZED Ho...  1426       0.0    100%
AU117349.1 AU117349 HEMBA1 Homo sapiens cDNA clone HEMBA100120...  1426       0.0    100%
AL553679.3 AL553679 Homo sapiens PLACENTA COT 25-NORMALIZED Ho...  1419       0.0    100%
BQ962441.1 AGENCOURT_10056322 NIH_MGC_71 Homo sapiens cDNA clo...  1419       0.0    100%
BU902298.1 AGENCOURT_10127317 NIH_MGC_71 Homo sapiens cDNA clo...  1417       0.0    100%
EB387180.1 nbj26d10.y1 Human optic nerve. Unnormalized (nbj) H...  1415       0.0    100%
```

ALIGNMENTS

```
Query       1    AGTGTGAAATCTTCAGAGAAGAATTTCTCTTTAGTTCTTTGCAAGAAGGTAGAGATAAAGACACTTTTTCAAAAATGGCAATGGTATCAGAATTCCTCAAGCAGGCCTGGTTTATTGAAAATGAAGAGCAGGAATATGTTCAAACTGTGA  150
AL553114.3  107  ......................................................................................................................................................  256
CX870542.1  29   ......................................................................................................................................................  178
BQ953713.1  1         .................................................................................................................................................  145
BM458053.1  11   ......................................................................................................................................................  160
AL544959.3  1            ..............................................................................................................................................  142
AU117349.1  1    ......................................................................................................................................................  150
AL553679.3  1            ..............................................................................................................................................  142
BQ962441.1  1                ..........................................................................................................................................  138
BU902298.1  24             ............................................................................................................................................  164
                                                                                \
                                                                                |
                                                                                N
EB387180.1  1                  ........................................................................................................................................  136

Query       151  AGTCATCCAAAGGTGGTCCCGGATCAGCGGTGAGCCCCTATCCTACCTTCAATCCATCCTCGGATGTCGCTGCCTTGCATAAGGCCATAATGGTTAAAGGTGTGGATGAAGCAACCATCATTGACATTCTAACTAAGCGAAACAATGCAC  300
AL553114.3  257  ......................................................................................................................................................  406
CX870542.1  179  ......................................................................................................................................................  328
BQ953713.1  146  ......................................................................................................................................................  295
BM458053.1  161  ......................................................................................................................................................  310
AL544959.3  143  ......................................................................................................................................................  292
AU117349.1  151  ......................................................................................................................................................  300
AL553679.3  143  ......................................................................................................................................................  292
BQ962441.1  139  ......................................................................................................................................................  288
BU902298.1  165  ......................................................................................................................................................  314
EB387180.1  137  ......................................................................................................................................................  286

Query       301  AGCGTCAACAGATCAAAGCAGCATATCTCCAGGAAACAGGAAAGCCCCTGGATGAAACACTGAAGAAAGCCCTTACAGGTCACCTTGAGGAGGTTGTTTTAGCTCTGCTAAAAACTCCAGCGCAATTTGATGCTGATGAACTTCGTGCTG  450
AL553114.3  407  ......................................................................................................................................................  556
CX870542.1  329  ......................................................................................................................................................  478
BQ953713.1  296  ......................................................................................................................................................  445
BM458053.1  311  ......................................................................................................................................................  460
AL544959.3  293  ......................................................................................................................................................  442
AU117349.1  301  ........................................................................C.............................................................................  450
AL553679.3  293  .....................................................................-................................................................................  441
BQ962441.1  289  ......................................................................................................................................................  438
BU902298.1  315  ......................................................................................................................................................  464
EB387180.1  287  ......................................................................................................................................................  436

Query       451  CCATGAAGGGCCTTGGAACTGATGAAGATACTCTAATTGAGATTTTGGCATCAAGAACTAACAAAGAAATCAGAGACATTAACAGGGTCTACAGAGAGGAACTGAAGAGAGATCTGGCCAAAGACATAACCTCAGACACATCTGGAGATT  600
AL553114.3  557  ......................................................................................................................................................  706
CX870542.1  479  ......................................................................................................................................................  628
BQ953713.1  446  ......................................................................................................................................................  595
BM458053.1  461  ......................................................................................................................................................  610
AL544959.3  443  ......................................................................................................................................................  592
AU117349.1  451  ......................................................................................................................................................  600
AL553679.3  442  ......................................................................................................................................................  591
BQ962441.1  439  ......................................................................................................................................................  588
BU902298.1  465  ......................................................................................................................................................  614
EB387180.1  437  ......................................................................................................................................................  586

Query       601  TTCGGAACGCTTTGCTTTCTCTTGCTAAGGGTGACCGATCTGAGGACTTTGGTGTGAATGAAGACTTGGCTGATTCAGATGCCAGGGCCTTGTATGAAGCAGGAGAAAGGAGAAAGGGGACAGACGTAAACGTGTTCAATACCATCCTTA  750
AL553114.3  707  ......................................................................................................................................................  856
CX870542.1  629  ......................................................................................................................................................  778
BQ953713.1  596  ......................................................................................................................................................  745
BM458053.1  611  ......................................................................................T...............................................................  760
AL544959.3  593  ......................................................................................................................................................  742
AU117349.1  601  .....................................................................................................N................................................  750
AL553679.3  592  ......................................................................................................................................................  741
BQ962441.1  589  ......................................................................................................................................................  738
BU902298.1  615  ......................................................................................................................................................  764
EB387180.1  587  ......................................................................................................................................................  736

Query       751  CCACCAGAAGCTATCCACAACTTCGCAGAG  780
AL553114.3  857  ..............................  886
CX870542.1  779  ..............................  808
BQ953713.1  746  ..............................  775
BM458053.1  761  ..............................  791
                                      \
                                      |
                                      C
AL544959.3  743  ..............................  772
AU117349.1  751  ...........................     777
AL553679.3  742  ..............................  771
BQ962441.1  739  ..............................  768
BU902298.1  765  ..............................  794
EB387180.1  737  ..............................  766
```

Query= N8-23 [organism=Homo sapiens] anxa1

Length=1377

```
                                                                     Score        E     Max
Sequences producing significant alignments:                         (Bits)     Value  ident

BU508007.1 AGENCOURT_10128424 NIH_MGC_71 Homo sapiens cDNA clo...  1820       0.0    96%
AL570428.3 AL570428 Homo sapiens PLACENTA COT 25-NORMALIZED Ho...  1803       0.0    97%
AL544231.3 AL544231 Homo sapiens PLACENTA COT 25-NORMALIZED Ho...  1796       0.0    96%
DC631365.1 DC631365 macaque bone marrow cDNA library QbmA Maca...  1768       0.0    93%
DC636772.1 DC636772 macaque bone marrow cDNA library QbmA Maca...  1744       0.0    92%
AL541874.3 AL541874 Homo sapiens PLACENTA Homo sapiens cDNA cl...  1742       0.0    97%
BU902298.1 AGENCOURT_10127317 NIH_MGC_71 Homo sapiens cDNA clo...  1736       0.0    95%
BX438944.2 BX438944 Homo sapiens PLACENTA Homo sapiens cDNA cl...  1735       0.0    97%
AL576223.3 AL576223 Homo sapiens PLACENTA COT 25-NORMALIZED Ho...  1722       0.0    96%
AL570884.3 AL570884 Homo sapiens PLACENTA COT 25-NORMALIZED Ho...  1720       0.0    96%

ALIGNMENTS

Query       1     AGTGTGAAATCTTCAGAGAAGAATTTCTCTTTAGTTCTTTGCAAGAAGGTAGAGATAAAGACACTTTTTCAAAAATGGCAATGGTATCAGAATTCCTCAAGCAGGCCTGGTTTATTGAAAATGAAGAGCAGGAATATGTTCAAACTGTGA  150
BU508007.1  1                         ..................................................................................................................................  131
                                                                                 \
                                                                                 |
                                                                                 N
AL544231.3  19                     ..T..................................................................................................................................  151
DC631365.1  1     ......-....................C.C........................................................................................................................  149
DC636772.1  1     ......-....................C.C........................................................................................................................  149
AL541874.3  1                              .............................................................................................................................  125
BU902298.1  24              ............................................................................................................................................  164
                                                                                 \
                                                                                 |
                                                                                 N
BX438944.2  1                      .....................................................................................................................................  133

Query       151   AGTCATCCAAAGGTGGTCCCGGATCAGCGGTGAGCCCCTATCCTACCTTCAATCCATCCTCGGATGTCGCTGCCTTGCATAAGGCCATAATGGTTAAAGGTGTGGATGAAGCAACCATCATTGACATTCTAACTAAGCGAAACAATGCAC  300
BU508007.1  132   ......................................................................................................................................................  281
AL570428.3  1088                                                                                                                                 .....-.T............-..  1068
AL544231.3  152   ......................................................................................................................................................  301
DC631365.1  150   .A.................T........A......................................T...........C......................................................................  299
DC636772.1  150   .A.................T........A..................................................C......................................................................  299
AL541874.3  126   ......................................................................................................................................................  275
BU902298.1  165   ......................................................................................................................................................  314
BX438944.2  134   ......................................................................................................................................................  283

Query       301   AGCGTCAACAGATCAAAGCAGCATATCTCCAGGAAACAGGAAAGCCCTGGATGAAACACTGAAGAAAGCCCTTACAGGTCACCTTGAGGAGGTTGTTTTAGCTCTGCTAAAAACTCCAGCGCAATTTGATGCTGATGAACTTCGTGCTGC  450
BU508007.1  282   ......................................................................................................................................................  432
                                                              \
                                                              |
                                                              C
AL570428.3  1067  ...T....-.A..-..........W.......-.........GC..........................-Y..............................................................................  921
                                                              \
                                                              |
                                                              C
AL544231.3  302   ......................................................................................................................................................  452
                                                              \
                                                              |
                                                              C
DC631365.1  300   ..........................................................................TG....................................................................G.....  450
                                                              \
                                                              |
                                                              C
DC636772.1  300   ........................................................................G.TG....................................................................G.....  450
                                                              \
                                                              |
                                                              C
AL541874.3  276   ......................................................................................................................................................  426
                                                              \
                                                              |
                                                              C
BU902298.1  315   ......................................................................................................................................................  465
                                                              \
                                                              |
                                                              C
BX438944.2  284   ......................................................................................................................................................  434
                                                              \
                                                              |
                                                              C
AL576223.3  1050       ......-W......-R...-.Y....-........-......CT.R...................-.....-.D.....WR............-.A....-V...................................-.......  916
AL570884.3  1059                 .........T.....T............C......W...............RS....W.....Y.M.....R...T...K......YY..S.W......C.............................K...S.  923
                                    \                            \
                                    |                            |
                                    M                            Y
```

```
Query      451  CATGAAGGCCTTGGAACTGATGAAGATACTCTAATTGAGATTTTGGCATCAAGAACTAACAAAGAAATCAGAGACATTAACAGGGTCTACAGAGAGGAACTGAAGAGAGATCTGGCCAAAGACATAACCTCAGACACATCTGGAGATTT  600
BU508007.1 433  ......................................................................................................................................................  582
AL570428.3 920  ...............M...............................................................W......................................................................  771
AL544231.3 453  ......................................................................................................................................................  602
DC631365.1 451  ...........................C.....G.............................G......................................................................................  600
DC636772.1 451  ...........................C.....G....................................................................................................................  600
AL541874.3 427  ......................................................................................................................................................  576
BU902298.1 466  ......................................................................................................................................................  615
BX438944.2 435  ......................................................................................................................................................  584
AL576223.3 915  ................................W..AAR................................................................................................................  766
AL570884.3 922  ......................................................................................................................................................  773

Query      601  TCGGAACGCTTTGCTTTCTCTTGCTAAGGGTGACCGATCTGAGGACTTTGGTGTGAATGAAGACTTGTGATTCAGATCAGGCTTGAAGCAGAGAAAGGAGGGACAGACGTAAACGTGTTCAATACCATCCTTACCACCAGAAGCTATCCA  750
BU508007.1 583  ......................................................................................................................................................  748
                                                                                   \         \\  \\  \\\   \       \
                                                                                   |         ||  ||  |||   |       |
                                                                                  GC         G|  G|  T||   G   GAAAG
                                                                                              |   |   ||
                                                                                              C   C  TG|
                                                                                                       |
                                                                                                       A
AL570428.3 770  .............................................................R........................................................................................  605
                                                                                   \         \ \ \\\      \     \\\   \
                                                                                   |         | | |||      |     |||   |
                                                                                  GC        GC G C||      G     G||   N
                                                                                                  ||             ||
                                                                                                TTG|             A|
                                                                                                   |              |
                                                                                                   A             AA
AL544231.3 603  ......................................................................................................................................................  768
                                                                                   \         \\  \\  \\\   \       \
                                                                                   |         ||  ||  |||   |       |
                                                                                  GC         G|  G|  T||   G   GAAAG
                                                                                              |   |   ||
                                                                                              C   C  TG|
                                                                                                       |
                                                                                                       A
DC631365.1 601  .........................................................................................G.................T..............................C...........  766
                                                                                   \         \\  \\  \\\   \       \
                                                                                   |         ||  ||  |||   |       |
                                                                                  GC         G|  G|  T||   G   GAAAG
                                                                                              |   |   ||
                                                                                              C   C  TG|
                                                                                                       |
                                                                                                       A
DC636772.1 601  .........................................................................................G.................T..............................C...........  766
                                                                                   \         \\  \\  \\\   \       \
                                                                                   |         ||  ||  |||   |       |
                                                                                  GC         G|  G|  T||   G   GAAAG
                                                                                              |   |   ||
                                                                                              C   C  TG|
                                                                                                       |
                                                                                                       A
AL541874.3 577  ......................................................................................................................................................  742
                                                                                   \         \\  \\  \\\   \       \
                                                                                   |         ||  ||  |||   |       |
                                                                                  GC         G|  G|  T||   G   GAAAG
                                                                                              |   |   ||
                                                                                              C   C  TG|
                                                                                                       |
                                                                                                       A
BU902298.1 616  ......................................................................................................................................................  781
                                                                                   \         \\  \\  \\\   \       \
                                                                                   |         ||  ||  |||   |       |
                                                                                  GC         G|  G|  T||   G   GAAAG
                                                                                              |   |   ||
                                                                                              C   C  TG|
                                                                                                       |
                                                                                                       A
BX438944.2 585  ......................................................................................................................................................  750
                                                                                   \         \\  \\  \\\   \       \
                                                                                   |         ||  ||  |||   |       |
                                                                                  GC         G|  G|  T||   G   GAAAG
                                                                                              |   |   ||
                                                                                              C   C  TG|
                                                                                                       |
                                                                                                       A
AL576223.3 765  ......................................................................................................................................................  600
                                                                                   \         \ \ \\\      \     \\\\
                                                                                   |         | | |||      |     ||||
                                                                                  GC        GC G C||      G     G|||
                                                                                                  ||             |||
```

```
                                                                                                  TTG|             A||
                                                                                                     |              ||
                                                                                                     A             AA|
                                                                                                                     |
                                                                                                                     G
AL570884.3  772   ......................................................................................................................................................  607
                                                                                     \         \ \ \\\      \     \\\\
                                                                                     |         | | |||      |     ||||
                                                                                    GC        GC G C||      G     G|||
                                                                                                    ||             |||
                                                                                                  TTG|             A||
                                                                                                     |              ||
                                                                                                     A             AA|
                                                                                                                     |
                                                                                                                     G

Query       751   CAACTTCGCAGAGTGTTTCAGAAATACACCAAGTACAGTAAGCATGACATGAACAAAGTTCTGGACCTGGAGTTGAAAGGTGACATTGAGAAATGCCTCACAGCTATCGTGAAGTGCGCCACAAGCAAACCAGCTTTCTTTGCAGAGAAG  900
BU508007.1  749   ......................................................................................................................................................  898
AL570428.3  604   .............................................A........................................................................................................  455
AL544231.3  769   ......................................................................................................................-...............................  917
DC631365.1  767   ..T.................................................................................................................T................................A  916
DC636772.1  767   ..T.................................................................................................................T................................A  916
AL541874.3  743   ......................................................................................................................................................  892
BU902298.1  782   .........................................................................................A....................TG..........................C...........  931
BX438944.2  751   ......................................................................................................................................................  900
AL576223.3  599   ......................................................................................................................................................  450
AL570884.3  606   ......................................................................................................................................................  457

Query       901   CTTCATCAAGCCATGAAAGGTGTTGGAACTCGCCATAAGGCATTGATCAGGATTATGGTTTCCCGTTCTGAAATTGACATGAATGATATCAAAGCATTCTATCAGAAGATGTATGGTATCTCCCTTTGCCAAGCCATCCTGGATGAAACC  1050
BU508007.1  899   .......-..........................C.....................................................................-......G....G...............C..T...G..........  1049
                                                                          \                                             \                  \
                                                                          |                                             |                  |
                                                                          G                                             T                  C
AL570428.3  454   ...........................................................................A.........................................T................................  305
AL544231.3  918   ..........................................................................................................G.W-......WWY-.............MWY.-.........-..  1063
DC631365.1  917   ........G.............C.......................................A..........................................-.........................-......-..-.....-.-  1061
                                                                          \
                                                                          |
                                                                          G
DC636772.1  917   ........G.............C..........-............................A..................-...............................................-.-..-...-..-.....GG-  1061
                                                        \                 \                                          \
                                                        |                 |                                          |
                                                        T                 G                                          T
AL541874.3  893   ............................................................................................-....-........-.....W..K-......-.......-..M-..-......       1029
BU902298.1  932   .......................................C...........GA.....G...................T.......A...C....T........A....A.G....A.......C...G...C..C......T.......  1085
                                                                                                                 \                  \                  \            \
                                                                                                                 |                  |                  |            |
                                                                                                                 G                  A                  C            G
BX438944.2  901   ......................K...R.............S...........................................D.......R-....Y.............W..KD-.Y.......Y...                   1030
                                                                                                     \
                                                                                                     |
                                                                                                     A
AL576223.3  449   ......................................................................................................................................................  300
AL570884.3  456   ......................................................................................................................................................  307

Query       1051  AAAGGAGATTATGAGAAAATCCTGGTGGCTCTTTGTGGAGGAAACTAAACATTCCCTTGATGGTCTCAAGCTATGATCAGAAGACTTTAATTATATATTTTCATCCTATAAGCTTAAATAGGAAAGTTTCTTCAACAGGATTACAGTGTA  1200
BU508007.1  1050  C....GA...T..TA.........................................C......C.....                                                                                 1123
                                            \                 \        \      \
                                            |                 |        |      |
                                            G                 A        T     AG
AL570428.3  304   ......................................................................................................................................................  155
AL544231.3  1064  .....R...-........-..Y..K-.......K-...R....                                                                                                           1102
DC631365.1  1062  .--.....-....-...-..-.-....-............-..-....-...-....-....A-...-.......................-........CA...G..-.-..C...-.-..C.-...-..-.--......-..C..--.  1185
                                                                                                    \
                                                                                                    |
                                                                                                    A
DC636772.1  1062  .--.....-....-......-.....-............-..-....-...-.........-.....-....GA...........C-.......-....-....-..-.-...G..-...-.....C-..-.--..........G...-.  1188
                                                 \   \
                                                 |   |
                                                 C   G
BU902298.1  1086  ..G...A.......A.......C..G.............A....AA..............                                                                                          1152
                              \               \\        \         \  \  \
                              |               ||        |         |  |  |
                              G               G|        G         T  A  T
                                               |
                                               C
AL576223.3  299   ......................................................................................................................................................  150
AL570884.3  306   ......................................................................................................................................................  157

Query       1201  GCTACCTACATGCTGAAAAATATAGCCTTTAAATCATTTTTATATTATAACTCTGTATAATAGAGATAAGTCCATTTTTTAAAAATGTTTTCCCCAAACCATAAAACCCTATACAAGTTGTTCTAGTAACAATACATGAGAAAGATGTCT  1350
AL570428.3  154   ..........................................................T...........................................................................................  5
DC631365.1  1186  ........T-......C...-.....-.A....                                                                                                                     1216
```

```
                               \
                               |
                               G
DC636772.1  1189  .A.-...T-........T..GG.--...A....A...........                                                                                                         1231
                               \                          \
                               |                          |
                               G                          A
AL576223.3  149   ............................................................................................B........................................................   1
AL570884.3  156   ......................................................................................RW....V.............................N....N....W...............A.  5
                                                                                                                                              \          \
                                                                                                                                              |          |
                                                                                                                                              N          N

Query       1351  ATGT  1354
AL570428.3  4     ....  1
AL570884.3  4     ....  1

Query= N9-8 [organism=Homo sapiens] anxa1

Length=1237


                                                                  Score        E     Max
Sequences producing significant alignments:                      (Bits)     Value  ident

BQ228162.1 AGENCOURT_7258587 NIH_MGC_71 Homo sapiens cDNA clon...  1541       0.0    99%
AL576603.2 AL576603 Homo sapiens PLACENTA COT 25-NORMALIZED Ho...  1517       0.0    100%
AL576340.3 AL576340 Homo sapiens PLACENTA COT 25-NORMALIZED Ho...  1515       0.0    100%
AL576223.3 AL576223 Homo sapiens PLACENTA COT 25-NORMALIZED Ho...  1513       0.0    100%
AL574991.3 AL574991 Homo sapiens PLACENTA COT 25-NORMALIZED Ho...  1509       0.0    100%
BM555842.1 AGENCOURT_6544353 NIH_MGC_88 Homo sapiens cDNA clon...  1504       0.0    99%
AL570428.3 AL570428 Homo sapiens PLACENTA COT 25-NORMALIZED Ho...  1496       0.0    99%
AL570884.3 AL570884 Homo sapiens PLACENTA COT 25-NORMALIZED Ho...  1489       0.0    99%
BE614257.1 601504294T1 NIH_MGC_71 Homo sapiens cDNA clone IMAG...  1487       0.0    98%
AL575425.3 AL575425 Homo sapiens PLACENTA COT 25-NORMALIZED Ho...  1480       0.0    99%

ALIGNMENTS

Query       376   ACAGGTCACCTTGAGGAGGAACTGAAGAGAGATCTGGCCAAAGACATAACCTCAGACACATCTGGAGATTTTCGGAACGCTTTGCTTTCTCTTGCTAAGGGTGACCGATCTGAGGACTTTGGTGTGAATGAAGACTTGGCTGATTCAGAT  525
BQ228162.1  115                  .......................................................................................................................................  249
AL576603.2  828                  .......................................................................................................................................  694
AL576340.3  832                  .......................................................................................................................................  698
AL576223.3  821                  .......................................................................................................................................  687
AL574991.3  821                  .......................................................................................................................................  687
BM555842.1  74                   .......................................................................................................................................  208
AL570428.3  826                  .....................................................................................................................R.................  692
AL570884.3  828                  .......................................................................................................................................  694
BE614257.1  854   ........-.-A..-...A.T.........A.................C...........................................-.........................................................  707
                                          \                                        \
                                          |                                        |
                                          A                                        G
AL575425.3  839                  ....R.............................................................................................R..................B........S........  705

Query       526   GCCAGGGCCTTGTATGAAGCAGGAGAAAGGAGAAAGGGGACAGACGTAAACGTGTTCAATACCATCCTTACCACCAGAAGCTATCCACAACTTCGCAGAGTGTTTCAGAAATACACCAAGTACAGTAAGCATGACATGAACAAAGTTCTG  675
BQ228162.1  250   ......................................................................................................................................................  399
AL576603.2  693   ......................................................................................................................................................  544
AL576340.3  697   ......................................................................................................................................................  548
AL576223.3  686   ......................................................................................................................................................  537
AL574991.3  686   ......................................................................................................................................................  537
BM555842.1  209   ......................................................................................................................................................  358
AL570428.3  691   ......................................N.............................................................................................A.................  542
AL570884.3  693   ......................................................................................................................................................  544
BE614257.1  706   ......................................................................................................................................................  557
AL575425.3  704   ......................................T.............................................................................................N.................  555

Query       676   GACCTGGAGTTGAAAGGTGACATTGAGAAATGCCTCACAGCTATCGTGAAGTGCGCCACAAGCAAACCAGCTTTCTTTGCAGAGAAGCTTCATCAAGCCATGAAAGGTGTTGGAACTCGCCATAAGGCATTGATCAGGATTATGGTTTCC  825
BQ228162.1  400   ......................................................................................................................................................  549
AL576603.2  543   ......................................................................................................................................................  394
AL576340.3  547   ......................................................................................................................................................  398
AL576223.3  536   ......................................................................................................................................................  387
AL574991.3  536   ......................................................................................................................................................  387
BM555842.1  359   ......................................................................................................................................................  508
AL570428.3  541   ......................................................................................................................................................  392
AL570884.3  543   ......................................................................................................................................................  394
BE614257.1  556   ......................................................................................................................................................  407
AL575425.3  554   ...................................N..................................................................................................................  405

Query       826   CGTTCTGAAATTGACATGAATGATATCAAAGCATTCTATCAGAAGATGTATGGTATCTCCCTTTGCCAAGCCATCCTGGATGAAACCAAAGGAGATTATGAGAAAATCCTGGTGGCTCTTTGTGGAGGAAACTAAACATTCCCTTGATGG  975
BQ228162.1  550   ......................................................................................................................................................  699
AL576603.2  393   ......................................................................................................................................................  244
AL576340.3  397   ....................................................................................R.......R.........................................................  248
AL576223.3  386   ......................................................................................................................................................  237
```

```
AL574991.3  386   ........................................................................W.............................................................................  237
BM555842.1  509   ......................................................................................................................................................  658
AL570428.3  391   ............A.........................................T...............................................................................................  242
AL570884.3  393   ......................................................................................................................................................  244
BE614257.1  406   ......................................................................................................................................................  257
AL575425.3  404   ......................................................................................................................................................  255

Query       976   TCTCAAGCTATGATCAGAAGACTTTAATTATATATTTTCATCCTATAAGCTTAAATAGGAAAGTTTCTTCAACAGGATTACAGTGTAGCTACCTACATGCTGAAAAATATAGCCTTTAAATCATTTTTATATTATAACTCTGTATAATAG  1125
BQ228162.1  700   ......................................................................................................................................................  850
                                                                             \
                                                                             |
                                                                             A
AL576603.2  243   ......................................................................................................................................................  94
AL576340.3  247   ......................................................................................................................................................  98
AL576223.3  236   ......................................................................................................................................................  87
AL574991.3  236   .Y....................................................................................................................................................  87
BM555842.1  659   ......................................................................................................................................................  808
AL570428.3  241   .................................................................................................................................................T....  92
AL570884.3  243   ......................................................................................................................................................  94
BE614257.1  256   ......................................................................................................................................................  107
AL575425.3  254   .............................................................................................N........................................................  105

Query       1126  AGATAAGTCCATTTTTTAAAAATGTTTTCCCCAAACCATAAAACCCTATACAAGTTGTTCTAGTAACAATACATGAGAAAGATGTCTATGTAGCTGAAAATAAAATGACGTC  1237
BQ228162.1  851   ...............................................................G................................................  965
                                                                       \                      \                   \
                                                                       |                      |                   |
                                                                       G                      G                   A
AL576603.2  93    .....................................................................A......................                      1
                                                                                      \
                                                                                      |
                                                                                      C
AL576340.3  97    ............................................................................................                      5
                                                                                       \
                                                                                       |
                                                                                       N
AL576223.3  86    .............................B........................................................                            1
AL574991.3  86    ......................................................................................                            1
BM555842.1  809   .........................................................G...............................................         922
                                        \     \              \              \        \ \          \     \  \
                                        |     |              |              |        | |          |     |  |
                                        A     C              C              N        C N          A     C  G
AL570428.3  91    ...........................................................................................                       1
AL570884.3  93    .......................RW....V.............................N....N....W...............A.....                       1
                                                                               \          \
                                                                               |          |
                                                                               N          N
BE614257.1  106   ................................................................................T.A-.................             6
                                                                                                                    \
                                                                                                                    |
                                                                                                                    T
AL575425.3  104   .....................................................................N............G.....                          17
```

Query= N10-2 [organism=Homo sapiens] anxa1

Length=927

```
                                                             Score        E     Max
Sequences producing significant alignments:                  (Bits)     Value  ident

BQ228162.1 AGENCOURT_7258587 NIH_MGC_71 Homo sapiens cDNA clon...  1594       0.0    99%
AL576603.2 AL576603 Homo sapiens PLACENTA COT 25-NORMALIZED Ho...  1576       0.0    100%
AL576340.3 AL576340 Homo sapiens PLACENTA COT 25-NORMALIZED Ho...  1574       0.0    100%
AL576223.3 AL576223 Homo sapiens PLACENTA COT 25-NORMALIZED Ho...  1572       0.0    100%
AL574991.3 AL574991 Homo sapiens PLACENTA COT 25-NORMALIZED Ho...  1568       0.0    100%
BM555842.1 AGENCOURT_6544353 NIH_MGC_88 Homo sapiens cDNA clon...  1568       0.0    99%
AL570428.3 AL570428 Homo sapiens PLACENTA COT 25-NORMALIZED Ho...  1552       0.0    99%
AL570884.3 AL570884 Homo sapiens PLACENTA COT 25-NORMALIZED Ho...  1548       0.0    99%
AL575425.3 AL575425 Homo sapiens PLACENTA COT 25-NORMALIZED Ho...  1535       0.0    99%
BE614257.1 601504294T1 NIH_MGC_71 Homo sapiens cDNA clone IMAG...  1528       0.0    99%
```

ALIGNMENTS

```
Query       43   AAGAAATCAGAGACATTAACAGGGTCTACAGAGAGGAACTGAAGAGAGATCTGGCCAAAGACATAACCTCAGACACATCTGGAGATTTTCGGAACGCTTTGCTTTCTCTTGCTAAGGGTGACCGATCTGAGGACTTTGGTGTGAATGAAG  192
BQ228162.1  83   ......................................................................................................................................................  232
AL576603.2  860  ......................................................................................................................................................  711
AL576340.3  864  ......................................................................................................................................................  715
AL576223.3  853  ......................................................................................................................................................  704
AL574991.3  853  ......................................................................................................................................................  704
BM555842.1  42   ......................................................................................................................................................  191
AL570428.3  858  .................W...................................................................................................................................R  709
AL570884.3  860  ......................................................................................................................................................  711
```

```
AL575425.3  871  ...........R........................R..............................................................................................R.................B  722
BE614257.1  871   ......................-..-........A.T.........A.................C...........................................-........................................  724
                                                          \                                        \
                                                          |                                        |
                                                          A                                        G

Query       193  ACTTGGCTGATTCAGATGCCAGGGCCTTGTATGAAGCAGGAGAAAGGAGAAAGGGGACAGACGTAAACGTGTTCAATACCATCCTTACCACCAGAAGCTATCCACAACTTCGCAGAGTGTTTCAGAAATACACCAAGTACAGTAAGCATG  342
BQ228162.1  233  ......................................................................................................................................................  382
AL576603.2  710  ......................................................................................................................................................  561
AL576340.3  714  ......................................................................................................................................................  565
AL576223.3  703  ......................................................................................................................................................  554
AL574991.3  703  ......................................................................................................................................................  554
BM555842.1  192  ......................................................................................................................................................  341
AL570428.3  708  .......................................................N.............................................................................................A  559
AL570884.3  710  ......................................................................................................................................................  561
AL575425.3  721  ........S..............................................T.............................................................................................N  572
BE614257.1  723  ......................................................................................................................................................  574

Query       343  ACATGAACAAAGTTCTGGACCTGGAGTTGAAAGGTGACATTGAGAAATGCCTCACAGCTATCGTGAAGTGCGCCACAAGCAAACCAGCTTTCTTTGCAGAGAAGCTTCATCAAGCCATGAAAGGTGTTGGAACTCGCCATAAGGCATTGA  492
BQ228162.1  383  ......................................................................................................................................................  532
AL576603.2  560  ......................................................................................................................................................  411
AL576340.3  564  ......................................................................................................................................................  415
AL576223.3  553  ......................................................................................................................................................  404
AL574991.3  553  ......................................................................................................................................................  404
BM555842.1  342  ......................................................................................................................................................  491
AL570428.3  558  ......................................................................................................................................................  409
AL570884.3  560  ......................................................................................................................................................  411
AL575425.3  571  ....................................................N.................................................................................................  422
BE614257.1  573  ......................................................................................................................................................  424

Query       493  TCAGGATTATGGTTTCCCGTTCTGAAATTGACATGAATGATATCAAAGCATTCTATCAGAAGATGTATGGTATCTCCCTTTGCCAAGCCATCCTGGATGAAACCAAAGGAGATTATGAGAAAATCCTGGTGGCTCTTTGTGGAGGAAACT  642
BQ228162.1  533  ......................................................................................................................................................  682
AL576603.2  410  ......................................................................................................................................................  261
AL576340.3  414  .....................................................................................................R.......R........................................  265
AL576223.3  403  ......................................................................................................................................................  254
AL574991.3  403  .........................................................................................W............................................................  254
BM555842.1  492  ......................................................................................................................................................  641
AL570428.3  408  .............................A.........................................T..............................................................................  259
AL570884.3  410  ......................................................................................................................................................  261
AL575425.3  421  ......................................................................................................................................................  272
BE614257.1  423  ......................................................................................................................................................  274

Query       643  AAACATTCCCTTGATGGTCTCAAGCTATGATCAGAAGACTTTAATTATATATTTCATCCTATAAGCTTAAATAGGAAAGTTTCTTCAACAGGATTACAGTGTAGCTACCTACATGCTGAAAAATATAGCCTTTAAATCATTTTTATATT  792
BQ228162.1  683  ......................................................................................................................................................  833
                                                                                             \
                                                                                             |
                                                                                             A
AL576603.2  260  ......................................................................................................................................................  111
AL576340.3  264  ......................................................................................................................................................  115
AL576223.3  253  ......................................................................................................................................................  104
AL574991.3  253  ..................Y...................................................................................................................................  104
BM555842.1  642  ......................................................................................................................................................  791
AL570428.3  258  ......................................................................................................................................................  109
AL570884.3  260  ......................................................................................................................................................  111
AL575425.3  271  ..............................................................................................................N.......................................  122
BE614257.1  273  ......................................................................................................................................................  124

Query       793  ATAACTCTGTATAATAGAGATAAGTCCATTTTTTAAAAATGTTTTCCCCAAACCATAAAACCCTATACAAGTTGTTCTAGTAACAATACATGAGAAAGATGTCTATGTAGCTGAAAATAAAAATGACGTC  922
BQ228162.1  834  ................................................................................G....................................AT...........  965
                                                                                       \                      \
                                                                                       |                      |
                                                                                       G                      G
AL576603.2  110  ......................................................................................A......................                       1
                                                                                                      \
                                                                                                      |
                                                                                                      C
AL576340.3  114  .............................................................................................................                       5
                                                                                                       \
                                                                                                       |
                                                                                                       N
AL576223.3  103  ..............................................B........................................................                             1
AL574991.3  103  .......................................................................................................                             1
BM555842.1  792  ..........................................................................G..................................................       925
                                                        \     \              \              \        \ \          \     \  \
                                                        |     |              |              |        | |          |     |  |
                                                        A     C              C              N        C N          A     C  G
AL570428.3  108  ............T...............................................................................................                        1
AL570884.3  110  ........................................RW....V.............................N....N....W...............A.....                        1
                                                                                               \          \
                                                                                               |          |
                                                                                               N          N
AL575425.3  121  ......................................................................................N............G.....                           17
```

```
BE614257.1  123   ............................................................................................T.A-.................             6
                                                                                                                              \
                                                                                                                              |
                                                                                                                              T

Query= N11-16 [organism=Homo sapiens] anxa1

Length=1930


                                                                   Score        E     Max
Sequences producing significant alignments:                       (Bits)     Value  ident

BX417009.2 BX417009 Homo sapiens PLACENTA Homo sapiens cDNA cl...  1748       0.0    99%
AU134300.1 AU134300 OVARC1 Homo sapiens cDNA clone OVARC100168...  1443       0.0    99%
AU138502.1 AU138502 PLACE1 Homo sapiens cDNA clone PLACE100869...  1310       0.0    98%
CA421424.1 UI-H-EZ1-bbb-c-06-0-UI.s1 NCI_CGAP_Ch2 Homo sapiens...  1286       0.0    100%
AI764962.1 wh57a06.x1 NCI_CGAP_Kid11 Homo sapiens cDNA clone I...  1206       0.0    97%
BF107418.1 601824036F1 NIH_MGC_79 Homo sapiens cDNA clone IMAG...  1057       0.0    98%
DB013065.1 DB013065 TESOP2 Homo sapiens cDNA clone TESOP200154...  1040       0.0    100%
DB328492.1 DB328492 PLACE6 Homo sapiens cDNA clone PLACE600972...  950        0.0    100%
BG231632.1 naf37c05.x1 Soares_NPBMC Homo sapiens cDNA clone IM...  944        0.0    100%
BU674442.1 UI-CF-DU0-aab-j-09-0-UI.s1 UI-CF-DU0 Homo sapiens c...  937        0.0    100%

ALIGNMENTS

Query       1     AGTGTGAAATCTTCAGAGAAGAATTTCTCTTTAGTTCTTTGCAAAAGGTAGAGATAAAGACACTTTTTCAAAAATGGCAATGGTATCAGAATTCCTCAAGCAGGCCTGGTTTATTGAAAATGAAGAGCAGGAATATGTTCAAACTGTGAA  150
BX417009.2  1                 ..........................................................................................................................................  139
                                                              \
                                                              |
                                                              G
AU134300.1  1     ......................................................................................................................................................  151
                                                              \
                                                              |
                                                              G
AU138502.1  1     .................................................................C....................................................................................  151
                                                              \
                                                              |
                                                              G
BF107418.1  1                      .....................................................................................................................................  134
                                                              \
                                                              |
                                                              G

Query       151   GTCATCCAAAGGTGGTCCCGGATCAGCGGTGAGCCCCTATCCTACCTTCAATCCATCCTCGGATGTCGCTGCCTTGCATAAGGCCATAATGGTTAAAGGTGTGGATGAAGCAACCATCATTGACATTCTAACTAAGCGAAACAATGCACA  300
BX417009.2  140   ......................................................................................................................................................  289
AU134300.1  152   ......................................................................................................................................................  301
AU138502.1  152   ......................................................................................................................................................  301
BF107418.1  135   ......................................................................................................................................................  284

Query       301   GCGTCAACAGATCAAAGCAGCATATCTCCAGGAAACAGGAAAGGTAAGTTAGAGTGGTAAATTTAGATATTTAATTTCAGCATAGTTATACTTAACCATGGATTCGGAAGCACAGTTACCTAGTTCTTTAAGGTTCTAACCACTGTTTTC  450
BX417009.2  290   ......................................................................................................................................................  439
AU134300.1  302   ......................................................................................................................................................  451
AU138502.1  302   ......................................................................................................................................................  451
BF107418.1  285   ......................................................................................................................................................  434

Query       451   TCATTACATCTATGATTGGGATTGCAGTGTTTATCCACTTTGTTGCAATTTAATCAATTTTATCAAATTTCCTATTTTTATACATTAGTCATCTTGGTGTATATTGTTTGCAGATGTGGTGCTCTGGGGACAAATTTTTAATTTGAACGT  600
BX417009.2  440   ......................................................................................................................................................  589
AU134300.1  452   ..........................................................................................................N...........................................  601
AU138502.1  452   ......................................................................................................................................................  601
BF107418.1  435   ................G............................-...................................................-......-.-...........................................  580
DB013065.1  1                                                                                            ...............................................................  63

Query       601   AAACATCGAGATTGCTGCTGTCAATAAAGAATATGGTCTGATTGAAATGTACTAATATTTAAACTGAACTGTTTAATGCCTAAATTTTATATATAATTCTACTTTAAAAATAAGCCTTATGTCTTTTATACCAAATATGAGTAGTTTTCT  750
BX417009.2  590   ......................................................................................................................................................  739
AU134300.1  602   ..................................................-.........................................................................................N...N.....  750
AU138502.1  602   ................................................................................................A........................G........N........G....N....C  761
                                              \                                    \                                 \          \  \        \            \\   \
                                              |                                    |                                 |          |  |        |            ||   |
                                              G                                    G                                 C          A  A        C            A|  NG
                                                                                                                                                          |
                                                                                                                                                          T

BF107418.1  581   T..AT...........TG..G....C....                                                                                                                        610
DB013065.1  64    ......................................................................................................................................................  214
                                                                                       \
                                                                                       |
                                                                                       T

Query       751   AAAGCAATGAAATTAGAAAACTATATAATTTCAGTATCTGATTATACTGCTTGTTATTTGAGAAGTACAAACCTCAAAGATTGGAAACATGAATATATTATTTTAAAAGTAATTTTACTTCTGTTTTCTGTTAGCACACAGTCCTCTGTG  900
BX417009.2  740   ......................................................................................................................................................  889
AU134300.1  751   ........................N.................-......                                                                                                     798
AU138502.1  762   .......                                                                                                                                               770
```

```
                      \
                      |
                      G
DB013065.1  215   ......................................................................................................................................................  364

Query       901   TTTTGATCTTTGTGATTTCTCCCCTGTGTATAAGATTACTAACCCTATGCTTTAAGAAGTTATTGATGAATTCAGTAAATTTTATTTTAATCCCTCTAGATCTGGAAGGGTAAGATAATGTTTCAAAATATTTGGTACACTTTGTATGTG  1050
BX417009.2  890   ....................-...............-.......-.......-.......-.W.........-.........-....                                                                969
DB013065.1  365   ......................................................................................................................................................  514

Query       1051  AAGGGAAGAAGCTCTAAAAGATGGTTGGGTTTAGGGATGAGTTGTACGATGACCTAAAGTCAGGTAATATCACATTTTTTAACCTAGCATGTTACTTATTGGAAGTCCTGATTCTAATCtttttttttGTAGCCCCTGGATGAAACACTG  1200
DB013065.1  515   .....................................................                                                                                                  567

Query       1201  AAGAAAGCCCTTACAGGTCACCTTGAGGAGGTTGTTTTAGCTCTGCTAAAAACTCCAGCGCAATTTGATGCTGATGAACTTCGTGCTGCCATGAAGGGCCTTGGAACTGATGAAGATACTCTAATTGAGATTTTGGCATCAAGAACTAAC  1350
CA421424.1  727                           .....-....-...................................................................................................................  604
AI764962.1  731   ...-...............C....A.......-..................C...-.....................CT..................C......C.............................G.C......-...T..  585
                                                                             \
                                                                             |
                                                                             G

Query       1351  AAAGAAATCAGAGACATTAACAGGGTCTACAGAGAGGAACTGAAGAGAGATCTGGCCAAAGACATAACCTCAGACACATCTGGAGATTTCGGAACGCTTTGCTTTCTCTTGCTAAGGGTGACCGATCTGAGGACTTTGGTGTGAATGAA  1500
CA421424.1  603   ......................................................................................................................................................  454
AI764962.1  584   C.G...T......C.....C........C.........C...............-..C.......T....................................................................................  436
DB328492.1  528                                                                  .......................................................................................  442
BG231632.1  525                                                                     ....................................................................................  442
BU674442.1  536                                                                      ...................................................................................  454

Query       1501  GACTTGGCTGATTCAGATGCCAGGGCCTTGTATGAAGCAGGAGAAAGGAGAAAGGGGACAGACGTAAACGTGTTCAATACCATCCTTACCACCAGAAGCTATCCACAACTTCGCAGAGGTAACAATAAATTTCTTTTTCTGGAATCTGTT  1650
CA421424.1  453   ......................................................................................................................................................  304
AI764962.1  435   ......................................................................................................................................................  286
DB328492.1  441   ......................................................................................................................................................  292
BG231632.1  441   ......................................................................................................................................................  292
BU674442.1  453   ........................C.............................................................................................................................  304

Query       1651  TATGGAAGATGCAATTTTCTTTTTGATGACAAATAAGAGAAAGTAAAAACAGAACCCTTTTTCAATATCACTCCTGTATCAAACAGATACAGTGTTCCTGACCCATTGTTATTTGTCAATTTGTCCAATTTTGTACAGCCGCTACTTTA  1800
CA421424.1  303   ......................................................................................................................................................  154
AI764962.1  285   ......................................................................................................................................................  136
DB328492.1  291   ......................................................................................................................................................  142
BG231632.1  291   ......................................................................................................................................................  142
BU674442.1  303   ......................................................................................................................................................  154

Query       1801  TCATATTTAATACATTTTGTGACTTGGGCATGTGGAACATAATTTTGTTATTTTCACTGCTTTTACTCCCCTAAAAGATTTGGAATATGTGTGGGAAAAGACTGCATAACAGAACAAATGAAATTAGAAT  1930
CA421424.1  153   ..................................................................................................................................  23
                                                                                                                        \
                                                                                                                        |
                                                                                                                        A
AI764962.1  135   ..................................................................................................................................  5
                                                                                                                        \
                                                                                                                        |
                                                                                                                        A
DB328492.1  141   ..................................................................................................................................  11
                                                                                                                        \
                                                                                                                        |
                                                                                                                        A
BG231632.1  141   ..................................................................................................................................  11
                                                                                                                        \
                                                                                                                        |
                                                                                                                        A
BU674442.1  153   ..................................................................................................................................  23
                                                                                                                        \
                                                                                                                        |
                                                                                                                        A
```

Query= N12-C* [organism=Homo sapiens] anxa1

Length=1542

```
                                                                   Score        E     Max
Sequences producing significant alignments:                       (Bits)     Value  ident

AL570428.3 AL570428 Homo sapiens PLACENTA COT 25-NORMALIZED Ho...  1910       0.0    98%
BU508007.1 AGENCOURT_10128424 NIH_MGC_71 Homo sapiens cDNA clo...  1855       0.0    98%
AL576223.3 AL576223 Homo sapiens PLACENTA COT 25-NORMALIZED Ho...  1831       0.0    98%
AL544231.3 AL544231 Homo sapiens PLACENTA COT 25-NORMALIZED Ho...  1831       0.0    98%
AL570884.3 AL570884 Homo sapiens PLACENTA COT 25-NORMALIZED Ho...  1825       0.0    97%
AL574991.3 AL574991 Homo sapiens PLACENTA COT 25-NORMALIZED Ho...  1810       0.0    98%
AL575425.3 AL575425 Homo sapiens PLACENTA COT 25-NORMALIZED Ho...  1788       0.0    98%
AL541874.3 AL541874 Homo sapiens PLACENTA Homo sapiens cDNA cl...  1788       0.0    99%
DC631365.1 DC631365 macaque bone marrow cDNA library QbmA Maca...  1784       0.0    94%
AL553129.3 AL553129 Homo sapiens PLACENTA COT 25-NORMALIZED Ho...  1770       0.0    98%
```

ALIGNMENTS

```
Query       1     AGTGTGAAATCTTCAGAGAAGAATTTCTCTTTAGTTCTTTGCAAGAAGGTAGAGATAAAGGGAAGGTGTGGGAAGGACTTGTGAAATACATATTCGAGGAAAAACTATGCACAAGGCCGTGCATTTAAAAATAAACTCCCTAAGGCTGGG  150
BU508007.1  1                         ........................................  40
AL544231.3  19                     ..T........................................  61
AL541874.3  1                              ...................................  35
DC631365.1  1     ......-....................C.C..............................  59

Query       151   GTGAAACCTGCTACGGTCTGCGCAAGTTGACTGTTAATGAATTTGATTCTCAGGTACACTTTTTCAAAAATGGCAATGGTATCAGAATTCCTCAAGCAGGCCTGGTTTATTGAAAATGAAGAGCAGGAATATGTTCAAACTGTGAAGTCA  300
BU508007.1  41                                                           ...............................................................................................  136
                                                                            \
                                                                            |
                                                                            N
AL544231.3  62                                                           ...............................................................................................  156
AL541874.3  36                                                           ...............................................................................................  130
DC631365.1  60                                                           ...........................................................................................A...  154
AL553129.3  3                                                            ...........................................................................................A...  97

Query       301   TCCAAAGGTGGTCCCGGATCAGCGGTGAGCCCCTATCCTACCTTCAATCCATCCTCGGATGTCGCTGCCTTGCATAAGGCCATAATGGTTAAAGGTGTGGATGAAGCAACCATCATTGACATTCTAACTAAGCGAAACAATGCACAGCGT  450
AL570428.3  1088                                                                                                                                                .....-.T............-.....T.  1063
BU508007.1  137   ......................................................................................................................................................  286
AL544231.3  157   ......................................................................................................................................................  306
AL541874.3  131   ......................................................................................................................................................  280
DC631365.1  155   ..............T........A......................................T...........C...........................................................................  304
AL553129.3  98    ......................................................................................................................................................  248
                             \
                             |
                             A

Query       451   CAACAGATCAAAGCAGCATATCTCCAGGAAACAGGAAAGCCCCTGGATGAAACACTGAAGAAAGCCCTTACAGGTCACCTTGAGGAGGTTGTTTTAGCTCTGCTAAAAACTCCAGCGCAATTTGATGCTGATGAACTTCGTGCTGCCATG  600
AL570428.3  1062  ...-.A..-..........W.......-.........GC...........................-Y..................................................................................  917
BU508007.1  287   ......................................................................................................................................................  436
AL576223.3  1050  ......-W......-R...-.Y....-........-........-.R...................-.....-.D.....WR............-.A....-V..................................-............  912
AL544231.3  307   ......................................................................................................................................................  456
AL570884.3  1059            .........T.....T............C...Y...W...............RS....W.....Y.M.....R...T...K......YY..S.W......C.............................K...S.....  919
                               \
                               |
                               M
AL574991.3  1017                                       .......-...-R.....-K...-..RS..-...CR..............-...K.............................................-............  912
AL575425.3  1037                                          ........-..........-........M.......M...................KG.........C.............................C..C...S....A  930
AL541874.3  281   ......................................................................................................................................................  430
DC631365.1  305   ......................................................................TG....................................................................G.........  454
AL553129.3  249   ......................................................................................................................................................  398

Query       601   AAGGGCCTTGGAACTGATGAAGATACTCTAATTGAGATTTTGGCATCAAGAACTAACAAAGAAATCAGAGACATTAACAGGGTCTACAGAGAGGAACTGAAGAGAGATCTGGCCAAAGACATAACCTCAGACACATCTGGAGATTTTCGG  750
AL570428.3  916   ...........M...............................................................W..........................................................................  767
BU508007.1  437   ......................................................................................................................................................  586
AL576223.3  911   ............................W..AAR....................................................................................................................  762
AL544231.3  457   ......................................................................................................................................................  606
AL570884.3  918   ......................................................................................................................................................  769
AL574991.3  911   ......................................................................................................................................................  762
AL575425.3  929   .....................................................................R........................R.......................................................  780
AL541874.3  431   ......................................................................................................................................................  580
DC631365.1  455   .......................C.....G.............................G..........................................................................................  604
AL553129.3  399   .....................................................................R................................................................................  548

Query       751   AACGCTTTGCTTTCTCTTGCTAAGGGTGACCGATCTGAGGACTTTGGTGTGAATGAAGACTTGGCTGATTCAGATGCCAGGGCCTTGTATGAAGCAGGAGAAAGGAGAAAGGGGACAGACGTAAACGTGTTCAATACCATCCTTACCACC  900
AL570428.3  766   .........................................................R.......................................................N....................................  617
BU508007.1  587   ......................................................................................................................................................  736
AL576223.3  761   ......................................................................................................................................................  612
AL544231.3  607   ......................................................................................................................................................  756
AL570884.3  768   ......................................................................................................................................................  619
AL574991.3  761   ......................................................................................................................................................  612
AL575425.3  779   .......................................R.................B........S..............................................T....................................  630
AL541874.3  581   ......................................................................................................................................................  730
DC631365.1  605   ...............................................................................................G.......................T..............................  754
AL553129.3  549   ......................................................................................................................................................  698

Query       901   AGAAGCTATCCACAACTTCGCAGAGTGTTTCAGAAATACACCAAGTACAGTAAGCATGACATGAACAAAGTTCTGGACCTGGAGTTGAAAGGTGACATTGAGAAATGCCTCACAGCTATCGTGAAGTGCGCCACAAGCAAACCAGCTTTC  1050
AL570428.3  616   .........................................................A............................................................................................  467
BU508007.1  737   ......................................................................................................................................................  886
AL576223.3  611   ......................................................................................................................................................  462
AL544231.3  757   ..................................................................................................................................-...................  905
AL570884.3  618   ......................................................................................................................................................  469
AL574991.3  611   ......................................................................................................................................................  462
AL575425.3  629   .........................................................N....................................................N.......................................  480
AL541874.3  731   ......................................................................................................................................................  880
DC631365.1  755   C.............T.................................................................................................................T.....................  904
AL553129.3  699   ...............................................................................................................WY.....................................  848

Query       1051  TTTGCAGAGAAGCTTCATCAAGCCATGAAAGGTGTTGGAACTCGCCATAAGGCATTGATCAGGATTATGGTTTCCCGTTCTGAAATTGACATGAATGATATCAAAGCATTCTATCAGAAGATGTATGGTATCTCCCTTTGCCAAGCCATC  1200
AL570428.3  466   .......................................................................................A.........................................T....................  317
BU508007.1  887   ...................-..........................C.....................................................................-......G....G...............C..T..  1037
```

```
                                                                                      \                                             \                  \
                                                                                      |                                             |                  |
                                                                                      G                                             T                  C
AL576223.3  461   ......................................................................................................................................................  312
AL544231.3  906   ......................................................................................................................G.W-......WWY-..............MWY.  1053
AL570884.3  468   ......................................................................................................................................................  319
AL574991.3  461   ...................................................................................................................................................W..  312
AL575425.3  479   ......................................................................................................................................................  330
AL541874.3  881   .........................................................................................................-....-.......-.....W..K-......-.......-..M-..  1023
DC631365.1  905   ...........A........G.............C.......................................A..........................................-.........................-......  1053
                                                                                      \
                                                                                      |
                                                                                      G
AL553129.3  849   .......................................................................................R-...............-....-.......-............Y....-.....M.-..-...  991

Query       1201  CTGGATGAAACCAAAGGAGATTATGAGAAAATCCTGGTGGCTCTTTGTGGAGGAAACTAAACATTCCCTTGATGGTCTCAAGCTATGATCAGAAGACTTTAATTATATATTTTCATCCTATAAGCTTAAATAGGAAAGTTTCTTCAACAG  1350
AL570428.3  316   ......................................................................................................................................................  167
BU508007.1  1038  .G..........C....GA...T..TA..........................................C......C....                                                                       1123
                                                        \                 \        \      \
                                                        |                 |        |      |
                                                        G                 A        T     AG
AL576223.3  311   ......................................................................................................................................................  162
AL544231.3  1054  -........-.......R...-........-..Y..K-.......K-...R....                                                                                                 1102
AL570884.3  318   ......................................................................................................................................................  169
AL574991.3  311   ............................................................................Y.........................................................................  162
AL575425.3  329   ......................................................................................................................................................  180
AL541874.3  1024  -......                                                                                                                                                 1029
DC631365.1  1054  -..-.....-.-.--......-....-...-..-....-.............-..-....-...-....-....A-...-.......................-........CA...G..-.-..C...-.-..C.-...-..-.--...  1176
                                                                                                                \
                                                                                                                |
                                                                                                                A
AL553129.3  992   -......W.-....                                                                                                                                          1003

Query       1351  GATTACAGTGTAGCTACCTACATGCTGAAAAATATAGCCTTTAAATCATTTTTATATTATAACTCTGTATAATAGAGATAAGTCCATTTTTTAAAAATGTTTTCCCCAAACCATAAAACCCTATACAAGTTGTTCTAGTAACAATACATG  1500
AL570428.3  166   ......................................................................T...............................................................................  17
AL576223.3  161   ........................................................................................................B.............................................  12
AL570884.3  168   ..................................................................................................RW....V.............................N....N....W.....  17
                                                                                                                                                          \          \
                                                                                                                                                          |          |
                                                                                                                                                          N          N
AL574991.3  161   ......................................................................................................................................................  12
AL575425.3  179   ..................N.............................................................................................................................N.....  30
DC631365.1  1177  ...-..C..--.........T-......C...-.....-.A....                                                                                                           1216
                                           \
                                           |
                                           G

Query       1501  AGAAAGATGTCTATGT  1516
AL570428.3  16    ................  1
AL576223.3  11    ...........       1
AL570884.3  16    ..........A.....  1
AL574991.3  11    ...........       1
AL575425.3  29    .......G.....     17
```

Query= N13-17 [organism=Homo sapiens] anxa1

Length=1234

Sequences producing significant alignments:	Score (Bits)	E Value	Max ident
BQ228162.1 AGENCOURT_7258587 NIH_MGC_71 Homo sapiens cDNA clon...	1679	0.0	99%
AL574991.3 AL574991 Homo sapiens PLACENTA COT 25-NORMALIZED Ho...	1648	0.0	100%
AL576603.2 AL576603 Homo sapiens PLACENTA COT 25-NORMALIZED Ho...	1635	0.0	99%
AL576223.3 AL576223 Homo sapiens PLACENTA COT 25-NORMALIZED Ho...	1633	0.0	99%
BM555842.1 AGENCOURT_6544353 NIH_MGC_88 Homo sapiens cDNA clon...	1633	0.0	99%
AL570428.3 AL570428 Homo sapiens PLACENTA COT 25-NORMALIZED Ho...	1629	0.0	99%
AL576340.3 AL576340 Homo sapiens PLACENTA COT 25-NORMALIZED Ho...	1628	0.0	99%
AL570884.3 AL570884 Homo sapiens PLACENTA COT 25-NORMALIZED Ho...	1628	0.0	99%
AL575425.3 AL575425 Homo sapiens PLACENTA COT 25-NORMALIZED Ho...	1615	0.0	99%
AL553095.3 AL553095 Homo sapiens PLACENTA COT 25-NORMALIZED Ho...	1589	0.0	98%

ALIGNMENTS

```
Query       308   AACTGATGAAGATACTCTAATTGAGATTTTGGCATCAAGAACTAACAAAGAAATCAGAGACATTAACAGGGTCTACAGAGAGGAACTGAAGAGAGATCTGGCCAAAGACATAACTCAGACACATCTGGAGATTTTCGGAACGCTTTGCTT  457
BQ228162.1  36    ......................................................................................................................................................  186
                                                                                                                                   \
                                                                                                                                   |
                                                                                                                                   C
AL574991.3  900   ......................................................................................................................................................  750
                                                                                                                                    \
```

```
                                                                                                                                    |
                                                                                                                                    C
AL576603.2  904    ...........-WM..............-........................................................................................................................  757
                                                                                                                                    \
                                                                                                                                    |
                                                                                                                                    C
AL576223.3  900   .................W..AAR...............................................................................................................................  750
                                                                                                                                    \
                                                                                                                                    |
                                                                                                                                    C
BM555842.1  1           ................................................................................................................................................  145
                                                                                                                                   \
                                                                                                                                   |
                                                                                                                                   C
AL570428.3  904    ...............................................................W.....................................................................................  755
                                                                                                                                    \
                                                                                                                                    |
                                                                                                                                    C
AL576340.3  908   ..........-RW..................-.......-..............................................................................................................  761
                                                                                                                                    \
                                                                                                                                    |
                                                                                                                                    C
AL570884.3  907   ......................................................................................................................................................  757
                                                                                                                                    \
                                                                                                                                    |
                                                                                                                                    C
AL575425.3  918   ..........................................................R........................R..................................................................  768
                                                                                                                                    \
                                                                                                                                    |
                                                                                                                                    C
AL553095.3  906   ......................................................................................................................................................  756
                                                                                                                                    \
                                                                                                                                    |
                                                                                                                                    C

Query       458   TCTCTTGCTAAGGGTGACCGATCTGAGGACTTTGGTGTGAATGAAGACTTGGCTGATTCAGATGCCAGGGCCTTGTATGAAGCAGGAGAAAGGAGAAAGGGGACAGACGTAAACGTGTTCAATACCATCCTTACCACCAGAAGCTATCCA  607
BQ228162.1  187   ......................................................................................................................................................  336
AL574991.3  749   ......................................................................................................................................................  600
AL576603.2  756   ......................................................................................................................................................  607
AL576223.3  749   ......................................................................................................................................................  600
BM555842.1  146   ......................................................................................................................................................  295
AL570428.3  754   .............................................R.......................................................N................................................  605
AL576340.3  760   ......................................................................................................................................................  611
AL570884.3  756   ......................................................................................................................................................  607
AL575425.3  767   ...........................R.................B........S..............................................T................................................  618
AL553095.3  755   ......................................................................................................................................................  606

Query       608   CAACTTCGCAGAGTGTTTCAGAAATACACCAAGTACAGTAAGCATGACATGAACAAAGTTCTGGACCTGGAGTTGAAAGGTGACATTGAGAAATGCCTCACAGCTATCGTGAAGTGCGCCACAAGCAAACCAGCTTTCTTTGCAGAGAAG  757
BQ228162.1  337   ......................................................................................................................................................  486
AL574991.3  599   ......................................................................................................................................................  450
AL576603.2  606   ......................................................................................................................................................  457
AL576223.3  599   ......................................................................................................................................................  450
BM555842.1  296   ......................................................................................................................................................  445
AL570428.3  604   .............................................A........................................................................................................  455
AL576340.3  610   ......................................................................................................................................................  461
AL570884.3  606   ......................................................................................................................................................  457
AL575425.3  617   .............................................N....................................................N...................................................  468
AL553095.3  605   .......................................................................................................................................YY...V.........  456

Query       758   CTTCATCAAGCCATGAAAGGTGTTGGAACTCGCCATAAGGCATTGATCAGGATTATGGTTTCCCGTTCTGAAATTGACATGAATGATATCAAAGCATTCTATCAGAAGATGTATGGTATCTCCCTTTGCCAAGCCATCCTGGATGAAACC  907
BQ228162.1  487   ......................................................................................................................................................  636
AL574991.3  449   .......................................................................................................................................W..............  300
AL576603.2  456   ......................................................................................................................................................  307
AL576223.3  449   ......................................................................................................................................................  300
BM555842.1  446   ......................................................................................................................................................  595
AL570428.3  454   ...........................................................................A.........................................T................................  305
AL576340.3  460   ...................................................................................................................................................R..  311
AL570884.3  456   ......................................................................................................................................................  307
AL575425.3  467   ......................................................................................................................................................  318
AL553095.3  455   ..C.......................V......................................S..S..................RK...R...G...R..........................................K...G..  306

Query       908   AAAGGAGATTATGAGAAAATCCTGGTGGCTCTTTGTGGAGGAAACTAAACATTCCCTTGATGGTCTCAAGCTATGATCAGAAGACTTTAATTATATATTTTCATCCTATAAGCTTAAATAGGAAAGTTTCTTCAACAGGATTACAGTGTA  1057
BQ228162.1  637   ......................................................................................................................................................  787
                                                                                                                                            \
                                                                                                                                            |
                                                                                                                                            A
AL574991.3  299   ................................................................Y.....................................................................................  150
AL576603.2  306   ......................................................................................................................................................  157
AL576223.3  299   ......................................................................................................................................................  150
BM555842.1  596   ......................................................................................................................................................  745
AL570428.3  304   ......................................................................................................................................................  155
AL576340.3  310   .....R................................................................................................................................................  161
```

```
AL570884.3  306   ......................................................................................................................................................  157
AL575425.3  317   ......................................................................................................................................................  168
AL553095.3  305   .....R................................................................................................................................................  156

Query       1058  GCTACCTACATGCTGAAAAATATAGCCTTTAAATCATTTTTATATTATAACTCTGTATAATAGAGATAAGTCCATTTTTTAAAAATGTTTTCCCCAAACCATAAAACCCTATACAAGTTGTTCTAGTAACAATACATGAGAAAGATGTCT  1207
BQ228162.1  788   ..............................................................................................................................G.......................  939
                                                                                                                                      \                      \
                                                                                                                                      |                      |
                                                                                                                                      G                      G
AL574991.3  149   .....................................................................................................................................................   1
AL576603.2  156   ....................................................................................................................................A.................  6
                                                                                                                                                   \
                                                                                                                                                   |
                                                                                                                                                   C
AL576223.3  149   ............................................................................................B........................................................   1
BM555842.1  746   ........................................................................................................................G.............................  903
                                                                                                       \     \              \              \        \ \          \     \
                                                                                                       |     |              |              |        | |          |     |
                                                                                                       A     C              C              N        C N          A     C
AL570428.3  154   ..........................................................T...........................................................................................  5
AL576340.3  160   ......................................................................................................................................................  10
                                                                                                                                                    \
                                                                                                                                                    |
                                                                                                                                                    N
AL570884.3  156   ......................................................................................RW....V.............................N....N....W...............A.  5
                                                                                                                                              \          \
                                                                                                                                              |          |
                                                                                                                                              N          N
AL575425.3  167   ......N.............................................................................................................................N............G....  18
AL553095.3  155   ......................................................................................................................................................  5
                                                                                                                                            \
                                                                                                                                            |
                                                                                                                                            N

Query       1208  ATGTAGCTGAAAATAAAATGACGTC  1232
BQ228162.1  940   .........................  965
                           \
                           |
                           A
AL576603.2  5     .....                      1
BM555842.1  904   ..................         922
                    \
                    |
                    G
AL570428.3  4     ....                       1
AL576340.3  9     .....                      5
AL570884.3  4     ....                       1
AL575425.3  17    .                          17
AL553095.3  4     .G..                       1
```

Query= N14-15 [organism=Homo sapiens] anxa1

Length=3707

```
                                                                 Score        E     Max
Sequences producing significant alignments:                      (Bits)     Value  ident

BX417009.2 BX417009 Homo sapiens PLACENTA Homo sapiens cDNA cl...  1748       0.0    99%
CX869128.1 HESC4_32_H02.g1_A037 NIH_MGC_262 Homo sapiens cDNA ...  1644       0.0    100%
BM556687.1 AGENCOURT_6540720 NIH_MGC_88 Homo sapiens cDNA clon...  1491       0.0    96%
BQ723044.1 AGENCOURT_8232565 Lupski_sympathetic_trunk Homo sap...  1483       0.0    98%
AU134300.1 AU134300 OVARC1 Homo sapiens cDNA clone OVARC100168...  1443       0.0    99%
DR422934.1 nav19c11.y1 Human pterygium. Unnormalized (nav) Hom...  1310       0.0    100%
AU138502.1 AU138502 PLACE1 Homo sapiens cDNA clone PLACE100869...  1310       0.0    98%
CA414418.1 UI-H-EZ0-bas-f-10-0-UI.s1 NCI_CGAP_Ch1 Homo sapiens...  1293       0.0    99%
CB321626.1 UI-CF-EN1-aeg-d-16-0-UI.s1 UI-CF-EN1 Homo sapiens c...  1266       0.0    99%
BQ721913.1 AGENCOURT_8101100 Lupski_sympathetic_trunk Homo sap...  1236       0.0    96%
```

ALIGNMENTS

```
Query       1     AGTGTGAAATCTTCAGAGAAGAATTTCTCTTTAGTTCTTTGCAAGAAGGTAGAGATAAGACACTTTTTCAAAAATGGCAATGGTATCAGAATTCCTCAAGCAGGCCTGGTTTATTGAAAATGAAGAGCAGGAATATGTTCAAACTGTGAA  150
BX417009.2  1                 ..........................................................................................................................................  139
                                                                          \
                                                                          |
                                                                          A
AU134300.1  1     ......................................................................................................................................................  151
                                                                          \
                                                                          |
                                                                          A
AU138502.1  1     .................................................................C....................................................................................  151
                                                                          \
```

```
                                                                           |
                                                                           A

Query        151   GTCATCCAAAGGTGGTCCCGGATCAGCGGTGAGCCCCTATCCTACCTTCAATCCATCCTCGGATGTCGCTGCCTTGCATAAGGCCATAATGGTTAAAGGTGTGGATGAAGCAACCATCATTGACATTCTAACTAAGCGAAACAATGCACA  300
BX417009.2   140   ......................................................................................................................................................  289
AU134300.1   152   ......................................................................................................................................................  301
AU138502.1   152   ......................................................................................................................................................  301

Query        301   GCGTCAACAGATCAAAGCAGCATATCTCCAGGAAACAGGAAAGGTAAGTTAGAGTGGTAAATTTAGATATTTAATTTCAGCATAGTTATACTTAACCATGGATTCGGAAGCACAGTTACCTAGTTCTTTAAGGTTCTAACCACTGTTTTC  450
BX417009.2   290   ......................................................................................................................................................  439
AU134300.1   302   ......................................................................................................................................................  451
AU138502.1   302   ......................................................................................................................................................  451

Query        451   TCATTACATCTATGATTGGGATTGCAGTGTTTATCCACTTTGTTGCAATTTAATCAATTTTATCAAATTTCCTATTTTTATACATTAGTCATCTTGGTGTATATTGTTTGCAGATGTGGTGCTCTGGGGACAAATTTTTAATTTGAACGT  600
BX417009.2   440   ......................................................................................................................................................  589
AU134300.1   452   ..........................................................................................................N...........................................  601
AU138502.1   452   ......................................................................................................................................................  601

Query        601   AAACATCGAGATTGCTGCTGTCAATAAAGAATATGGTCTGATTGAAATGTACTAATATTTAAACTGAACTGTTTAATGCCTAAATTTTATATATAATTCTACTTTAAAAATAAGCCTTATGTCTTTTATACCAAATATGAGTAGTTTTCT  750
BX417009.2   590   ......................................................................................................................................................  739
AU134300.1   602   ..................................................-.........................................................................................N...N.....  750
AU138502.1   602   ................................................................................................A........................G........N........G....N....C  761
                                               \                                    \                                 \          \  \        \            \\   \
                                               |                                    |                                 |          |  |        |            ||   |
                                               G                                    G                                 C          A  A        C            A|   NG
                                                                                                                                                           |
                                                                                                                                                           T

Query        751   AAAGCAATGAAATTAGAAAACTATATAATTTCAGTATCTGATTATACTGCTTGTTATTTGAGAAGTACAAACCTCAAAGATTGGAAACATGAATATATTATTTTAAAAGTAATTTTACTTCTGTTTTCTGTTAGCACACAGTCCTCTGTG  900
BX417009.2   740   ......................................................................................................................................................  889
AU134300.1   751   ........................N.................-......                                                                                                      798
AU138502.1   762   ........                                                                                                                                                770
                       \
                       |
                       G

Query        901   TTTTGATCTTTGTGATTTCTCCCCTGTGTATAAGATTACTAACCCTATGCTTTAAGAAGTTATTGATGAATTCAGTAAATTTTATTTTAATCCCTCTAGATCTGGAAGGGTAAGATAATGTTTCAAAATATTTGGTACACTTTGTATGTG  1050
BX417009.2   890   ....................-...............-.......-.......-.......-.W........-..........-....                                                                969

Query        1051  AAGGGAAGAAGCTCTAAAAGATGGTTGGGTTTAGGGATGAGTTGTACGATGACCTAAAGTCAGGTAATATCACATTTTTTAACCTAGCATGTTACTTATTGGAAGTCCTGATTCTAATCtttttttttGTAGCCCCTGGATGAAACACTG  1200
BQ723044.1   6                                                                                                                                       ....................  25

Query        1201  AAGAAAGCCCTTACAGGTCACCTTGAGGAGGTTGTTTTAGCTCTGCTAAAAACTCCAGCGCAATTTGATGCTGATGAACTTCGTGCTGCCATGAAGGGCCTTGGAACTGATGAAGATACTCTAATTGAGATTTTGGCATCAAGAACTAAC  1350
BQ723044.1   26    ......................................G...............................................................................................................  175

Query        1351  AAAGAAATCAGAGACATTAACAGGGTCTACAGAGAGGAACTGAAGAGAGATCTGGCCAAAGACATAACCTCAGACACATCTGGAGATTTTCGGAACGCTTTGCTTTCTCTTGCTAAGGGTGACCGATCTGAGGACTTTGGTGTGAATGAA  1500
CX869128.1   1                              .....-.......................................................................................................................  124
BQ723044.1   176   ......................................................................................................................................................  325

Query        1501  GACTTGGCTGATTCAGATGCCAGGGCCTTGTATGAAGCAGGAGAAAGGAGAAAGGGGACAGACGTAAACGTGTTCAATACCATCCTTACCACCAGAAGCTATCCACAACTTCGCAGAGTGTTTCAGAAATACACCAAGTACAGTAAGCAT  1650
CX869128.1   125   ......................................................................................................................................................  274
BQ723044.1   326   ......................................................................................................................................................  475

Query        1651  GACATGAACAAAGTTCTGGACCTGGAGTTGAAAGGTGACATTGAGAAATGCCTCACAGCTATCGTGAAGTGCGCCACAAGCAAACCAGCTTTCTTTGCAGAGAAGCTTCATCAAGCCATGAAAGTATGTACCATTCTACTTATATGTCCT  1800
CX869128.1   275   ......................................................................................................................................................  424
BQ723044.1   476   ......................................................................................................................................................  625

Query        1801  GCTTAGAGGAAGAATTATTTGTAGAAAGAACAGAAAACCTCATGTTGTTTGAAAATCTCACATTTAATATCTTCCCATTAATGAGAATCATTGTTCTATTTGATGAAAGAGGTAATACACTACCTTCTCAGAATAAACTTCTGTTAGATT  1950
CX869128.1   425   ...........A..........................................................................................................................................  574
BQ723044.1   626   .......C........................................................................................................G.................A...................  775

Query        1951  CGGTGCtttttttACAGATGAGATTGTAAAGTGAGCCTGTAAGTGATTTACGGCTCACTTGTGATTTGCTTAAAACATGTGTCACCACGGGAAGGGTGATGTACTTTCTTTTTTAACGTGTAATTTTAGTGGCCTACTTCCTCTAAGGT  2100
CX869128.1   575   ......C...............................................................................................................................................  724
BQ723044.1   776   ................C.......................C...G.....................................                                                                     868
                                          \          \   \    \   \      \    \  \     \\      \
                                          |          |   |    |   |      |    |  |     ||      |
                                          T          N   C    A   A      G    C  G     T|      A
                                                                                        |
                                                                                        G

Query        2101  TGTGAGTTTTATAATCTCCCACTTTATGAGTCTGATTTGTTGTTTTTATTATTCTGTCTTTTATGCCACAGATTAATAGTTGAAACTTTTGAAATGTCATTCTATGATTAAGGATGTAGAATGATGTATTTGAGATCAACTATGTCACTT  2250
CX869128.1   725   ......................................................................................................................................................  874

Query        2251  TAACTTTACTGTCTGACATGCATGGTATTATACAAGGTACCTCTTCTATTCTGACATTTTGAGCCCACAAATGAATAGTTATGAAGTTATCTTCCTTTTTTCTTTGCAGCTCCAAAATAAGGAGGTTTTCagagagagagagaAAGAAAG  2400
CX869128.1   875   .........................                                                                                                                               899
DR422934.1   1                                                                                                                                 ..........................  26

Query        2401  ATCTAACtatttgaataatatgaaggaatattttgaatatatatgaataatttgaataatatgaaatgataGACATGCTAGGTACTGCTCTGTAGTGGTTAGCATAGTTGCTGCCCCCTTTCTGCCATAGTCTAGTGTGATATTTCGTTG  2550
BM556687.1   1                                                                                        ...................................................................  67
                                                                                                                                                                        \
                                                                                                                                                                        |
                                                                                                                                                                        G
```

```
DR422934.1  27    ......................................................................................................................................................  177
                                                                                                                                                                       \
                                                                                                                                                                       |
                                                                                                                                                                       G

Query       2551  GTAATGATGAATACCTGGTGCCATGGAAGAGAAAAGTGGGACATATACTCCAGACTTTGGAAATCCAGGTAGCATTGCTGTCAGCTAAATTAACACCTGAAGTTTAAATAGAAATGAGCCAGATGAGGGCAAGAGTGGAAGAGATGGGAT  2700
BM556687.1  68    ......................................................................................................................................................  218
                                                                                                                                                                   \
                                                                                                                                                                   |
                                                                                                                                                                   G
DR422934.1  178   ......................................................................................................................................................  328
                                                                                                                                                                   \
                                                                                                                                                                   |
                                                                                                                                                                   G
BQ721913.1  3                                                                        ...................................................................................  86
                                                                                                                                                                   \
                                                                                                                                                                   |
                                                                                                                                                                   G

Query       2701  AAAGCAAGGGTGGTCCAAGAGGAAGAAGTAACCCACACACAGGCTTGAAGGCAAAAGGTAACATAACCTTCTCAAGACAGTAAGTATAGAGTGATGGTGCACACTGGAAGAGATGGTAGAATTAGGCAGGATATTGTAAGCCTTGAAAAA  2850
BM556687.1  219   ......................................................................................................................................................  368
DR422934.1  329   ......................................................................................................................................................  478
BQ721913.1  87    ......................................................................................................................................................  236

Query       2851  GAATTAGGCAGGATATCGGAAGCCCTGATTAGATTCTATCCTAAGAGCAACAGAAGATCACTGACAGTGTTTTAAATAGATAGACTAGTTTATTAGATTTGCAGTTTAGAAGTTCCCtttttttGTAATTATTGGACAGTGTAGAGACCG  3000
BM556687.1  369   ......................................................................................................................................................  518
DR422934.1  479   ......................................................................................................................................................  628
CA414418.1  753                                                                                                                                         ................  738
BQ721913.1  237   ......................................................................................................................................................  386

Query       3001  GATGGTGAGAGATGAGTTAGGAAGTTGTGACAGCTCTCTATACCTACCGCTAATGTAGAGGATTATTTATTTTCATTTCATTACCATTCGTGTAAGgtgtgtgtgtgtgtgtgtgtgtgtgtgtgtgtATAGCTAGTTTCTCTATAGA  3150
BM556687.1  519   ...................N.....................................................................N............................................................  672
                                                                                                                  \
                                                                                                                  |
                                                                                                               GTGT
DR422934.1  629   ........................................................................................                                                              716
CA414418.1  737   ......................................................................................................................................................  582
                                                                                                                                                    \
                                                                                                                                                    |
                                                                                                                                               GTGTGT
CB321626.1  720          ..........................................................................................................................--.N.A................  580
BQ721913.1  387   ....................................................................................................................................................A.  540
                                                                                                                  \
                                                                                                                  |
                                                                                                               GTGT

Query       3151  AATTATATGGAAGAGTAAGAAGGCTTCACTTTAAAATTCATGTTTTTAATGAGAGTATTTTCTGAATATGAGACACTTACCCTCATTTATTTTGGCCAGGGTGTTGGAACTCGCCATAAGGCATTGATCAGGATTATGGTTTCCCGTTCT  3300
BM556687.1  673   ..........................................G.............................................G....-........T.........C.....................................  825
                                                              \                                  \                                                           \      \
                                                              |                                  |                                                           |      |
                                                              G                                  C                                                           G      G
CA414418.1  581   ........................................................................................G.............................................................  432
CB321626.1  579   ......................................................................................................................................................  430
BQ721913.1  541   G.........G............................................................A.............................C.............C.......................G.........C  695
                                                                                                                    \      \                 \                     \\
                                                                                                                    |      |                 |                     ||
                                                                                                                    A      G                 T                     C|
                                                                                                                                                                    |
                                                                                                                                                                    T

Query       3301  GAAATTGACATGAATGATATCAAAGCATTCTATCAGAAGATGTATGGTATCTCCCTTTGCCAAGCCATCCTGGATGAAACCAAGGAGATTATGAGAAAATCCTGGTGGCTCTTTGTGGAGGAAACTAAACATTCCCTTGATGGTCTCAAG  3450
BM556687.1  826   ................G....C............C.......G..........................................                                                                 925
                      \         \              \         \    \  \   \   \  \    \   \     \  \    \
                      |         |              |         |    |  |   |   |  |    |   |     |  |    |
                      A         T              T         G    A  G   C   C  T    A   C    GG  G    C
CA414418.1  431   ......................................................................................................................................................  281
                                                                                                     \
                                                                                                     |
                                                                                                     A
CB321626.1  429   ......................................................................................................................................................  279
                                                                                                     \
                                                                                                     |
                                                                                                     A
BQ721913.1  696   .....................CC...........G...A...C....G....................                                                                                  769
                                    \     \              \        \      \   \
                                    |     |              |        |      |   |
                                    T     G              C        C      C   G

Query       3451  CTATGATCAGAAGACTTTAATTATATATTTCATCCTATAAGCTTAAATAGGAAAGTTTCTTCAACAGGATTACAGTGTAGCTACCTACATGCTGAAAAATATAGCCTTTAAATCATTTTTATATTATAACTCTGTATAATAGAGATAAG  3600
CA414418.1  280   ......................................................................................................................................................  131
CB321626.1  278   ......................................................................................................................................................  129
```

```
Query       3601  TCCATTTTTTAAAAATGTTTTCCCCAAACCATAAAACCCTATACAAGTTGTTCTAGTAACAATACATGAGAAAGATGTCTATGTAGCTGAAAATAAAATGACGTCAC  3707
CA414418.1  130   ....................................................N......................................................  24
CB321626.1  128   ...........................................................................................................  22

Query= N15-2 [organism=Homo sapiens] anxa1

Length=961


                                                                 Score        E     Max
Sequences producing significant alignments:                      (Bits)     Value  ident

BQ228162.1 AGENCOURT_7258587 NIH_MGC_71 Homo sapiens cDNA clon...  1591       0.0    99%
AL576603.2 AL576603 Homo sapiens PLACENTA COT 25-NORMALIZED Ho...  1567       0.0    100%
AL576340.3 AL576340 Homo sapiens PLACENTA COT 25-NORMALIZED Ho...  1565       0.0    100%
AL576223.3 AL576223 Homo sapiens PLACENTA COT 25-NORMALIZED Ho...  1563       0.0    100%
AL574991.3 AL574991 Homo sapiens PLACENTA COT 25-NORMALIZED Ho...  1559       0.0    100%
BM555842.1 AGENCOURT_6544353 NIH_MGC_88 Homo sapiens cDNA clon...  1554       0.0    99%
AL570428.3 AL570428 Homo sapiens PLACENTA COT 25-NORMALIZED Ho...  1543       0.0    99%
AL570884.3 AL570884 Homo sapiens PLACENTA COT 25-NORMALIZED Ho...  1539       0.0    99%
AL575425.3 AL575425 Homo sapiens PLACENTA COT 25-NORMALIZED Ho...  1526       0.0    99%
BE614257.1 601504294T1 NIH_MGC_71 Homo sapiens cDNA clone IMAG...  1520       0.0    98%

ALIGNMENTS

Query       86   ATCAGAGACATTAACAGGGTCTACAGAGAGGAACTGAAGAGAGATCTGGCCAAAGACATAACCTCAGACACATCTGGAGATTTTCGGAACGCTTTGCTTTCTCTTGCTAAGGGTGACCGATCTGAGGACTTTGGTGTGAATGAAGACTTG  235
BQ228162.1  88   ......................................................................................................................................................  237
AL576603.2  855  ......................................................................................................................................................  706
AL576340.3  859  ......................................................................................................................................................  710
AL576223.3  848  ......................................................................................................................................................  699
AL574991.3  848  ......................................................................................................................................................  699
BM555842.1  47   ......................................................................................................................................................  196
AL570428.3  853  ............W...................................................................................................................................R.....  704
AL570884.3  855  ......................................................................................................................................................  706
AL575425.3  866  ......R........................R..............................................................................................R.................B.....  717
BE614257.1  867  ..................-..-........A.T.........A.................C...........................................-.............................................  719
                                                     \                                        \
                                                     |                                        |
                                                     A                                        G

Query       236  GCTGATTCAGATGCCAGGGCCTTGTATGAAGCAGGAGAAAGGAGAAAGGGGACAGACGTAAACGTGTTCAATACCATCCTTACCACCAGAAGCTATCCACAACTTCGCAGAGTGTTTCAGAAATACACCAAGTACAGTAAGCATGACATG  385
BQ228162.1  238  ......................................................................................................................................................  387
AL576603.2  705  ......................................................................................................................................................  556
AL576340.3  709  ......................................................................................................................................................  560
AL576223.3  698  ......................................................................................................................................................  549
AL574991.3  698  ......................................................................................................................................................  549
BM555842.1  197  ......................................................................................................................................................  346
AL570428.3  703  ..................................................N.............................................................................................A.....  554
AL570884.3  705  ......................................................................................................................................................  556
AL575425.3  716  ...S..............................................T.............................................................................................N.....  567
BE614257.1  718  ......................................................................................................................................................  569

Query       386  AACAAAGTTCTGGACCTGGAGTTGAAAGGTGACATTGAGAAATGCCTCACAGCTATCGTGAAGTGCGCCACAAGCAAACCAGCTTTCTTTGCAGAGAAGCTTCATCAAGCCATGAAAGGTGTTGGAACTCGCCATAAGGCATTGATCAGG  535
BQ228162.1  388  ......................................................................................................................................................  537
AL576603.2  555  ......................................................................................................................................................  406
AL576340.3  559  ......................................................................................................................................................  410
AL576223.3  548  ......................................................................................................................................................  399
AL574991.3  548  ......................................................................................................................................................  399
BM555842.1  347  ......................................................................................................................................................  496
AL570428.3  553  ......................................................................................................................................................  404
AL570884.3  555  ......................................................................................................................................................  406
AL575425.3  566  ...............................................N......................................................................................................  417
BE614257.1  568  ......................................................................................................................................................  419

Query       536  ATTATGGTTTCCCGTTCTGAAATTGACATGAATGATATCAAAGCATTCTATCAGAAGATGTATGGTATCTCCCTTTGCCAAGCCATCCTGGATGAAACCAAAGGAGATTATGAGAAAATCCTGGTGGCTCTTTGTGGAGGAAACTAAACA  685
BQ228162.1  538  ......................................................................................................................................................  687
AL576603.2  405  ......................................................................................................................................................  256
AL576340.3  409  ................................................................................................R.......R.............................................  260
AL576223.3  398  ......................................................................................................................................................  249
AL574991.3  398  ....................................................................................W.................................................................  249
BM555842.1  497  ......................................................................................................................................................  646
AL570428.3  403  ........................A.........................................T...................................................................................  254
AL570884.3  405  ......................................................................................................................................................  256
AL575425.3  416  ......................................................................................................................................................  267
BE614257.1  418  ......................................................................................................................................................  269

Query       686  TTCCCTTGATGGTCTCAAGCTATGATCAGAAGACTTTAATTATATATTTTCATCCTATAAGCTTAAATAGGAAAGTTTCTTCAACAGGATTACAGTGTAGCTACCTACATGCTGAAAAATATAGCCTTTAAATCATTTTTATATTATAAC  835
BQ228162.1  688  ......................................................................................................................................................  838
                                                                        \
                                                                        |
                                                                        A
AL576603.2  255  ......................................................................................................................................................  106
```

```
AL576340.3  259  ......................................................................................................................................................  110
AL576223.3  248  ......................................................................................................................................................  99
AL574991.3  248  ............Y.........................................................................................................................................  99
BM555842.1  647  ......................................................................................................................................................  796
AL570428.3  253  ......................................................................................................................................................  104
AL570884.3  255  ......................................................................................................................................................  106
AL575425.3  266  .........................................................................................................N............................................  117
BE614257.1  268  ......................................................................................................................................................  119

Query       836  TCTGTATAATAGAGATAAGTCCATTTTTAAAAATGTTTTCCCCAAACCATAAAACCCTATACAAGTTGTTCTAGTAACAATACATGAGAAAGATGTCTATGTAGCTGAAAATAAAATGACGTC  959
BQ228162.1  839  ...........................................................................G................................................  965
                                                                                  \                      \                   \
                                                                                  |                      |                   |
                                                                                  G                      G                   A
AL576603.2  105  .................................................................................A......................                      1
                                                                                \
                                                                                |
                                                                                C
AL576340.3  109  ........................................................................................................                      5
                                                                                 \
                                                                                 |
                                                                                 N
AL576223.3  98   .........................................B........................................................                            1
AL574991.3  98   ..................................................................................................                            1
BM555842.1  797  .....................................................................G...............................................       922
                                                   \     \              \              \        \ \          \     \  \
                                                   |     |              |              |        | |          |     |  |
                                                   A     C              C              N        C N          A     C  G
AL570428.3  103  .......T...............................................................................................                       1
AL570884.3  105  ...................................RW....V.............................N....N....W...............A.....                       1
                                                                                          \          \
                                                                                          |          |
                                                                                          N          N
AL575425.3  116  .................................................................................N............G.....                          17
BE614257.1  118  ............................................................................................T.A-.................           6
                                                                                                                              \
                                                                                                                              |
                                                                                                                              T
```

Query= N16-14 [organism=Homo sapiens] anxa1

Length=2873

```
                                                                    Score        E     Max
Sequences producing significant alignments:                        (Bits)     Value  ident

AL544959.3 AL544959 Homo sapiens PLACENTA COT 25-NORMALIZED Ho...  1701       0.0    100%
BX438944.2 BX438944 Homo sapiens PLACENTA Homo sapiens cDNA cl...  1698       0.0    100%
AL551427.3 AL551427 Homo sapiens PLACENTA COT 25-NORMALIZED Ho...  1696       0.0    100%
AL540651.3 AL540651 Homo sapiens PLACENTA Homo sapiens cDNA cl...  1692       0.0    100%
AL551980.3 AL551980 Homo sapiens PLACENTA COT 25-NORMALIZED Ho...  1687       0.0    100%
AL544231.3 AL544231 Homo sapiens PLACENTA COT 25-NORMALIZED Ho...  1685       0.0    100%
AL541874.3 AL541874 Homo sapiens PLACENTA Homo sapiens cDNA cl...  1683       0.0    100%
BU902298.1 AGENCOURT_10127317 NIH_MGC_71 Homo sapiens cDNA clo...  1683       0.0    99%
BU508007.1 AGENCOURT_10128424 NIH_MGC_71 Homo sapiens cDNA clo...  1681       0.0    100%
AL553114.3 AL553114 Homo sapiens PLACENTA COT 25-NORMALIZED Ho...  1674       0.0    99%
```

ALIGNMENTS

```
Query       1     TGTGAAATCTTCAGAGAAGAATTTCTCTTTAGTTCTTTGCAAGAAGGTAGAGATAAAGACACTTTTTCAAAAATGGCAATGGTATCAGAATTCCTCAAGCAGGCCTGGTTTATTGAAAATGAAGAGCAGGAATATGTTCAAACTGTGAAG  150
AL544959.3  1           ................................................................................................................................................  144
BX438944.2  1                    .......................................................................................................................................  135
AL551427.3  1                  ..T......................................................................................................................................  137
AL540651.3  19                 ..T....................................................................................................................................  153
AL551980.3  22                   .....................................................................................................................................  154
AL544231.3  19                 ..T....................................................................................................................................  153
AL541874.3  1                        ...............................................................................................................................  127
BU902298.1  24           ...............................................................................................................................................  166
                                                                               \
                                                                               |
                                                                               N
BU508007.1  1                    ...................................................................................................................................  133
                                                                               \
                                                                               |
                                                                               N
AL553114.3  109   ......................................................................................................................................................  258

Query       151   TCATCCAAAGGTGGTCCCGGATCAGCGGTGAGCCCCTATCCTACCTTCAATCCATCCTCGGATGTCGCTGCCTTGCATAAGGCCATAATGGTTAAAGGTGTGGATGAAGCAACCATCATTGACATTCTAACTAAGCGAAACAATGCACAG  300
AL544959.3  145   ......................................................................................................................................................  294
BX438944.2  136   ......................................................................................................................................................  285
AL551427.3  138   ......................................................................................................................................................  287
```

```
AL540651.3  154   ......................................................................................................................................................  303
AL551980.3  155   ......................................................................................................................................................  304
AL544231.3  154   ......................................................................................................................................................  303
AL541874.3  128   ......................................................................................................................................................  277
BU902298.1  167   ......................................................................................................................................................  316
BU508007.1  134   ......................................................................................................................................................  283
AL553114.3  259   ......................................................................................................................................................  408

Query       301   CGTCAACAGATCAAAGCAGCATATCTCCAGGAAACAGGAAAGCCCCTGGATGAAACACTGAAGAAAGCCCTTACAGGTCACCTTGAGGAGGTTGTTTTAGCTCTGCTAAAAACTCCAGCGCAATTTGATGCTGATGAACTTCGTGCTGCC  450
AL544959.3  295   ......................................................................................................................................................  444
BX438944.2  286   ......................................................................................................................................................  435
AL551427.3  288   ......................................................................................................................................................  437
AL540651.3  304   ......................................................................................................................................................  453
AL551980.3  305   ......................................................................................................................................................  454
AL544231.3  304   ......................................................................................................................................................  453
AL541874.3  278   ......................................................................................................................................................  427
BU902298.1  317   ......................................................................................................................................................  466
BU508007.1  284   ......................................................................................................................................................  433
AL553114.3  409   ......................................................................................................................................................  558

Query       451   ATGAAGGGCCTTGGAACTGATGAAGATACTCTAATTGAGATTTTGGCATCAAGAACTAACAAAGAAATCAGAGACATTAACAGGGTCTACAGAGAGGAACTGAAGAGAGATCTGGCCAAAGACATAACCTCAGACACATCTGGAGATTTT  600
AL544959.3  445   ......................................................................................................................................................  594
BX438944.2  436   ......................................................................................................................................................  585
AL551427.3  438   ......................................................................................................................................................  587
AL540651.3  454   ......................................................................................................................................................  603
AL551980.3  455   ......................................................................................................................................................  604
AL544231.3  454   ......................................................................................................................................................  603
AL541874.3  428   ......................................................................................................................................................  577
BU902298.1  467   ......................................................................................................................................................  616
BU508007.1  434   ......................................................................................................................................................  583
AL553114.3  559   ......................................................................................................................................................  708

Query       601   CGGAACGCTTTGCTTTCTCTTGCTAAGGGTGACCGATCTGAGGACTTTGGTGTGAATGAAGACTTGGCTGATTCAGATGCCAGGGCCTTGTATGAAGCAGGAGAAAGGAGAAAGGGGACAGACGTAAACGTGTTCAATACCATCCTTACC  750
AL544959.3  595   ......................................................................................................................................................  744
BX438944.2  586   ......................................................................................................................................................  735
AL551427.3  588   ......................................................................................................................................................  737
AL540651.3  604   ......................................................................................................................................................  753
AL551980.3  605   ......................................................................................................................................................  754
AL544231.3  604   ......................................................................................................................................................  753
AL541874.3  578   ......................................................................................................................................................  727
BU902298.1  617   ......................................................................................................................................................  766
BU508007.1  584   ......................................................................................................................................................  733
AL553114.3  709   ......................................................................................................................................................  858

Query       751   ACCAGAAGCTATCCACAACTTCGCAGAGTGTTTCAGAAATACACCAAGTACAGTAAGCATGACATGAACAAAGTTCTGGACCTGGAGTTGAAAGGTGACATTGAGAAATGCCTCACAGCTATCGTGAAGTGCGCCACAAGCAAACCAGCT  900
AL544959.3  745   ........................................................................................................................................M.......M.....  895
                                                                                                         \
                                                                                                         |
                                                                                                         T
BX438944.2  736   ......................................................................................................................................................  885
AL551427.3  738   ......................................................................................................................................................  887
AL540651.3  754   ......................................................................................................................................................  903
AL551980.3  755   ......................................................W............................................................M..................................  904
AL544231.3  754   .....................................................................................................................................-................  902
AL541874.3  728   ......................................................................................................................................................  877
BU902298.1  767   ........................................................................................................A....................TG.......................  916
BU508007.1  734   ......................................................................................................................................................  883
AL553114.3  859   ...........................................................................................................W..-...........................-..-........  1005

Query       901   TTCTTTGCAGAGAAGCTTCATCAAGCCATGAAAGTATGTACCATTCTACTTATATGTCCTGCTTAGAGGAAGAATTATTTGTAGAAAGAACAGAAAACCTCATGTTGTTTGAAAATCTCACATTTAATATCTTCCCATTAATGAGAATCA  1050
AL544959.3  896   ..................................                                                                                                                      929
BX438944.2  886   ..................................                                                                                                                      919
AL551427.3  888   ..................................                                                                                                                      921
AL540651.3  904   ..................................                                                                                                                      937
AL551980.3  905   ..................................                                                                                                                      938
AL544231.3  903   ..................................                                                                                                                      936
AL541874.3  878   ..................................                                                                                                                      911
BU902298.1  917   ...C..............................                                                                                                                      950
BU508007.1  884   .......................-..........                                                                                                                      916
AL553114.3  1006  ........-....-.........-..M-.......                                                                                                                     1036

Query       1051  TTGTTCTATTTGATGAAAGAGGTAATACACTACCTTCTCAGAATAAACTTCTGTTAGATTCGGTGCttttttttACAGATGAGATTGTAAAGTGAGCCTGTAAGTGATTTACGGCTCACTTGTGATTTGCTTAAAACATGTGTCACCACG  1200

Query       1201  GGAAGGGTGATGTACTTTCTTTTTTAACGTGTAATTTTAGTGGCCTACTTCCTCTAAGGTTGTGAGTTTTATAATCTCCCACTTTATGAGTCTGATTTGTTGTTTTTATTATTCTGTCTTTTATGCCACAGATTAATAGTTGAAACTTTT  1350

Query       1351  GAAATGTCATTCTATGATTAAGGATGTAGAATGATGTATTTGAGATCAACTATGTCACTTTAACTTTACTGTCTGACATGCATGGTATTATACAAGGTACCTCTTCTATTCTGACATTTTGAGCCCACAAATGAATAGTTATGAAGTTAT  1500

Query       1501  CTTCCTTTTTTCTTTGCAGCTCCAAAATAAGGAGGTTTTCagagagagagagaAAGAAAGATCTAACtatttgaataatatgaaggaatattttgaatatatatgaataatttgaataatatgaaatgataGACATGCTAGGTACTGCTC  1650

Query       1651  TGTAGTGGTTAGCATAGTTGCTGCCCCCTTTCTGCCATAGTCTAGTGTGATATTTCGTTGGGTAATGATGAATACCTGGTGCCATGGAAGAGAAAAGTGGGACATATACTCCAGACTTTGGAAATCCAGGTAGCATTGCTGTCAGCTAAA  1800

Query       1801  TTAACACCTGAAGTTTAAATAGAAATGAGCCAGATGAGGGCAAGAGTGGAAGAGATGGGGATAAAGCAAGGGTGGTCCAAGAGGAAGAAGTAACCCACACACAGGCTTGAAGGCAAAAGGTAACATAACCTTCTCAAGACAGTAAGTATA  1950
```

```
Query       1951  GAGTGATGGTGCACACTGGAAGAGATGGTAGAATTAGGCAGGATATTGTAAGCCTTGAAAAAGAATTAGGCAGGATATCGGAAGCCCTGATTAGATTCTATCCTAAGAGCAACAGAAGATCACTGACAGTGTTTTAAATAGATAGACTAG  2100

Query       2101  TTTATTAGATTTGCAGTTTAGAAGTTCCCtttttttGTAATTATTGGACAGTGTAGAGACCGGATGGTGAGAGATGAGTTAGGAAGTTGTGACAGCTCTCTATACCTACCGCTAATGTAGAGGATTATTTATTTTCATTTCATTACCATT  2250

Query       2251  CGTGTAAGgtgtgtgtgtgtgtgtgtgtgtgtgtgtgtATAGCTAGTTTCTCTATAGAAATTATATGGAAGAGTAAGAAGGCTTCACTTTAAAATTCATGTTTTTAATGAGAGTATTTTCTGAATATGAGACACTTACCCTCATTT  2400

Query       2401  ATTTTGGCCAGGGTGTTGGAACTCGCCATAAGGCATTGATCAGGATTATGGTTTCCCGTTCTGAAATTGACATGAATGATATCAAAGCATTCTATCAGAAGATGTATGGTATCTCCCTTTGCCAAGCCATCCTGGATGAAACCAAAGGAG  2550
BX438944.2  919              ....K...R.............S...........................................D.......R-....Y.............W..KD-.Y.......Y...                             1030
                                                                                               \
                                                                                               |
                                                                                               A

AL544231.3  936              ........................................................................................G.W-......WWY-..............MWY.-........-.......R.  1070
AL541874.3  911              ..........................................................................-....-........-.....W..K-......-.......-..M-..-......             1029
BU508007.1  916              ................C.....................................................................-......G...G................C..T...G..........C....GA  1056
                                                                   \                                             \                  \
                                                                   |                                             |                  |
                                                                   G                                             T                  C

Query       2551  ATTATGAGAAAATCCTGGTGGCTCTTTGTGGAGGAAACTAAACATTCCCTTGATGGTCTCAA  2612
AL544231.3  1071  ..-........-..Y..K-.......K-...R....                            1102
BU508007.1  1057  ...T..TA.........................................C......C.....  1123
                                     \                 \        \      \
                                     |                 |        |      |
                                     G                 A        T     AG

Query= N17-2 [organism=Homo sapiens] anxa1

Length=988


                                                                Score        E     Max
Sequences producing significant alignments:                    (Bits)     Value  ident

BQ228162.1 AGENCOURT_7258587 NIH_MGC_71 Homo sapiens cDNA clon...  1624       0.0    99%
AL576603.2 AL576603 Homo sapiens PLACENTA COT 25-NORMALIZED Ho...  1600       0.0    100%
AL576223.3 AL576223 Homo sapiens PLACENTA COT 25-NORMALIZED Ho...  1596       0.0    100%
AL576340.3 AL576340 Homo sapiens PLACENTA COT 25-NORMALIZED Ho...  1592       0.0    100%
AL574991.3 AL574991 Homo sapiens PLACENTA COT 25-NORMALIZED Ho...  1592       0.0    100%
BM555842.1 AGENCOURT_6544353 NIH_MGC_88 Homo sapiens cDNA clon...  1587       0.0    99%
AL570428.3 AL570428 Homo sapiens PLACENTA COT 25-NORMALIZED Ho...  1576       0.0    99%
AL570884.3 AL570884 Homo sapiens PLACENTA COT 25-NORMALIZED Ho...  1572       0.0    99%
AL575425.3 AL575425 Homo sapiens PLACENTA COT 25-NORMALIZED Ho...  1559       0.0    99%
BU838395.1 AGENCOURT_8123206 Lupski_dorsal_root_ganglion Homo ...  1535       0.0    98%

ALIGNMENTS

Query       95    TCAAGAACTAACAAAGAAATCAGAGACATTAACAGGGTCTACAGAGAGGAACTGAAGAGAGATCTGGCCAAAGACATAACCTCAGACACATCTGGAGATTTTCGGAACGCTTTGCTTTCTCTTGCTAAGGGTGACCGATCTGAGGACTTT  244
BQ228162.1  70    ......................................................................................................................................................  219
AL576603.2  873   ......................................................................................................................................................  724
AL576223.3  866   ......................................................................................................................................................  717
AL576340.3  876   ....-.................................................................................................................................................  728
AL574991.3  866   ......................................................................................................................................................  717
BM555842.1  29    ......................................................................................................................................................  178
AL570428.3  871   ..............................W.......................................................................................................................  722
AL570884.3  873   ......................................................................................................................................................  724
AL575425.3  884   ........................R........................R..............................................................................................R.....  735
BU838395.1  27    ......................................................................................................................................................  176

Query       245   GGTGTGAATGAAGACTTGGCTGATTCAGATGCCAGGGCCTTGTATGAAGCAGGAGAAAGGAGAAAGGGGACAGACGTAAACGTGTTCAATACCATCCTTACCACCAGAAGCTATCCACAACTTCGCAGAGTGTTTCAGAAATACACCAAG  394
BQ228162.1  220   ......................................................................................................................................................  369
AL576603.2  723   ......................................................................................................................................................  574
AL576223.3  716   ......................................................................................................................................................  567
AL576340.3  727   ......................................................................................................................................................  578
AL574991.3  716   ......................................................................................................................................................  567
BM555842.1  179   ......................................................................................................................................................  328
AL570428.3  721   ............R.......................................................N.................................................................................  572
AL570884.3  723   ......................................................................................................................................................  574
AL575425.3  734   ............B........S..............................................T.................................................................................  585
BU838395.1  177   ......................................................................................................................................................  326

Query       395   TACAGTAAGCATGACATGAACAAAGTTCTGGACCTGGAGTTGAAAGGTGACATTGAGAAATGCCTCACAGCTATCGTGAAGTGCGCCACAAGCAAACCAGCTTTCTTTGCAGAGAAGCTTCATCAAGCCATGAAAGGTGTTGGAACTCGC  544
BQ228162.1  370   ......................................................................................................................................................  519
AL576603.2  573   ......................................................................................................................................................  424
AL576223.3  566   ......................................................................................................................................................  417
AL576340.3  577   ......................................................................................................................................................  428
AL574991.3  566   ......................................................................................................................................................  417
BM555842.1  329   ......................................................................................................................................................  478
AL570428.3  571   ............A.........................................................................................................................................  422
AL570884.3  573   ......................................................................................................................................................  424
AL575425.3  584   ............N....................................................N....................................................................................  435
BU838395.1  327   ......................................................................................................................................................  476
```

```
Query       545  CATAAGGCATTGATCAGGATTATGGTTTCCCGTTCTGAAATTGACATGAATGATATCAAAGCATTCTATCAGAAGATGTATGGTATCTCCCTTTGCCAAGCCATCCTGGATGAAACCAAAGGAGATTATGAGAAAATCCTGGTGGCTCTT  694
BQ228162.1  520  ......................................................................................................................................................  669
AL576603.2  423  ......................................................................................................................................................  274
AL576223.3  416  ......................................................................................................................................................  267
AL576340.3  427  ..................................................................................................................R.......R...........................  278
AL574991.3  416  ......................................................................................................W...............................................  267
BM555842.1  479  ......................................................................................................................................................  628
AL570428.3  421  ..........................................A.........................................T.................................................................  272
AL570884.3  423  ......................................................................................................................................................  274
AL575425.3  434  ......................................................................................................................................................  285
BU838395.1  477  ..........................................................N......................................N..............N..-................N.................  625

Query       695  TGTGGAGGAAACTAAACATTCCCTTGATGGTCTCAAGCTATGATCAGAAGACTTTAATTATATATTTTCATCCTATAAGCTTAAATAGGAAAGTTTCTTCAACAGGATTACAGTGTAGCTACCTACATGCTGAAAAATATAGCCTTTAAA  844
BQ228162.1  670  ......................................................................................................................................................  820
                                                                                                          \
                                                                                                          |
                                                                                                          A
AL576603.2  273  ......................................................................................................................................................  124
AL576223.3  266  ......................................................................................................................................................  117
AL576340.3  277  ......................................................................................................................................................  128
AL574991.3  266  ...............................Y......................................................................................................................  117
BM555842.1  629  ......................................................................................................................................................  778
AL570428.3  271  ......................................................................................................................................................  122
AL570884.3  273  ......................................................................................................................................................  124
AL575425.3  284  ...........................................................................................................................N..........................  135
BU838395.1  626  ..................................................................................T.................-...............................-...........C.-..  772

Query       845  TCATTTTTATATTATAACTCTGTATAATAGAGATAAGTCCATTTTTTAAAAATGTTTTCCCCAAACCATAAAACCCTATACAAGTTGTTCTAGTAACAATACATGAGAAAGATGTCTATGTAGCTGAAAATAAAATGACGTC  986
BQ228162.1  821  .............................................................................................G................................................  965
                                                                                                    \                      \                   \
                                                                                                    |                      |                   |
                                                                                                    G                      G                   A
AL576603.2  123  ...................................................................................................A......................                      1
                                                                                                                   \
                                                                                                                   |
                                                                                                                   C
AL576223.3  116  ...........................................................B........................................................                            1
AL576340.3  127  ..........................................................................................................................                      5
                                                                                                                    \
                                                                                                                    |
                                                                                                                    N
AL574991.3  116  ....................................................................................................................                            1
BM555842.1  779  .......................................................................................G...............................................         922
                                                                     \     \              \              \        \ \          \     \  \
                                                                     |     |              |              |        | |          |     |  |
                                                                     A     C              C              N        C N          A     C  G
AL570428.3  121  .........................T...............................................................................................                       1
AL570884.3  123  .....................................................RW....V.............................N....N....W...............A.....                       1
                                                                                                            \          \
                                                                                                            |          |
                                                                                                            N          N
AL575425.3  134  ...................................................................................................N............G....                           17
BU838395.1  773  ........................................-......-................C.......-.......NC..N..N.........-.........G..................N.............    908

  Database: Database of GenBank+EMBL+DDBJ sequences from EST Divisions
    Posted date:  Feb 24, 2019  1:46 AM
  Number of letters in database: 43,200,890,307
  Number of sequences in database:  77,566,966

Lambda      K        H
    1.33    0.621     1.12
Gapped
Lambda      K        H
    1.28    0.460    0.850
Matrix: blastn matrix:1 -2
Gap Penalties: Existence: 0, Extension: 0
Number of Sequences: 77566966
Number of Hits to DB: 2901464
Number of extensions: 648
Number of successful extensions: 648
Number of sequences better than 10: 24
Number of HSP's better than 10 without gapping: 0
Number of HSP's gapped: 514
Number of HSP's successfully gapped: 514
Length of database: 43200890307
A: 0
X1: 13 (25.0 bits)
X2: 32 (59.1 bits)
X3: 54 (99.7 bits)
S1: 13 (25.1 bits)
```
